# Continuous Flow Sodiation of Substituted Acrylonitriles, Alkenyl Sulfides and Acrylates

**DOI:** 10.1002/anie.202012085

**Published:** 2020-11-03

**Authors:** Johannes H. Harenberg, Niels Weidmann, Konstantin Karaghiosoff, Paul Knochel

**Affiliations:** ^1^ Department Chemie Ludwig-Maximilians-Universität München Butenandtstrasse 5–13, Haus F 81377 München Germany

**Keywords:** acrylonitriles, alkenyl sulfides, Barbier-type reactions, flow chemistry, sodiation

## Abstract

The sodiation of substituted acrylonitriles and alkenyl sulfides in a continuous flow set‐up using NaDA (sodium diisopropylamide) in EtNMe_2_ or NaTMP (sodium 2,2,6,6‐tetramethylpiperidide)⋅TMEDA in n‐hexane provides sodiated acrylonitriles and alkenyl sulfides, which are subsequently trapped in batch with various electrophiles such as aldehydes, ketones, disulfides and allylic bromides affording functionalized acrylonitriles and alkenyl sulfides. This flow‐procedure was successfully extended to other acrylates by using Barbier‐type conditions.

## Graphical abstract

Sodium in Flow: A sodiation of substituted acrylonitriles and alkenyl sulfides with NaDA or NaTMP⋅TMEDA was performed using a commercial continuous flow set‐up leading to the corresponding organosodiums. Trapping with various electrophiles afforded functionalized acrylonitriles and alkenyl sulfides. This flow‐procedure was successfully extended to other acrylates by using Barbier‐type conditions.

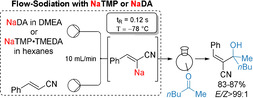

The metalation of unsaturated nitriles and sulfides is an important synthetic procedure.^[1]^ After quenching with various electrophiles, highly functionalized unsaturated products are obtained, which may be useful building blocks for biologically active heterocycles and natural products.^[2]^ The batch‐metalation of alkenyl nitriles or sulfides with lithium bases is often complicated due to competitive allylic lithiations.^[3]^ The use of stronger, more polar bases like sodium or potassium amides may avoid such limitations. However, the sodiation of such unsaturated compounds is much less explored.^[4]^ Moreover, the use of sodium organometallics is of high interest due to the low price, high abundancy and low toxicity of sodium salts.^[5]^ Recently, arylsodium compounds have been prepared by Collum using NaDA (sodium diisopropylamide) as deprotonating agent[^4e^, ^6^] and by Asako and Takai, who have investigated the utility of arylsodiums in catalytic cross‐couplings.^[7]^ Yoshida, Ley, Organ and others have demonstrated a high functional group tolerance performing challenging metalations in a continuous flow set‐up.^[8]^ Based on these studies, we have extended the Collum procedure to the preparation of sodiated aryl and heteroaryl derivatives which are difficult to generate otherwise and decompose upon batch‐sodiation.^[9]^ KDA⋅TMEDA (potassium diisopropylamide⋅*N*,*N*,*N*′,*N*′‐tetramethylethylenediamine) in *n*‐hexane was used in continuous flow for similar metalations.^[10]^ Herein, we wish to report that NaDA and NaTMP (TMPH=2,2,6,6‐tetramethylpiperidine) were efficient bases for the regioselective flow‐metalation of various substituted acrylonitriles and alkenyl sulfides.^[11]^ In first experiments, we have optimized the sodiation of cinnamonitrile (**1 a**) and have found that metalation with NaDA (0.24 m in DMEA (dimethylethylamine), 1.2 equiv) at −78 °C using a combined flow‐rate of 10 mL min^−1^ and a 0.02 mL reactor proceeded best with a residence time of 0.12 s affording organosodium **2 a**. Subsequent trapping with electrophiles of type **3** such as aldehydes, ketones, disulfides and allylic bromides afforded 2‐substituted cinnamonitriles of type **4** with usually high *E*/*Z* ratios (Table 1, entries 1–10). Thus, for a quenching with aromatic aldehydes, we obtained the *Z*‐product of type **4** as major product, whereas for more sterically hindered ketones the *E*‐product was formed.

**Table 1:** Sodiation of cinnamonitrile (**1 a**) using a microflow reactor and subsequent batch quench of the intermediate sodium organometallic **2 a** with various electrophiles of type **3** leading to functionalized cinnamonitriles of type **4**. 
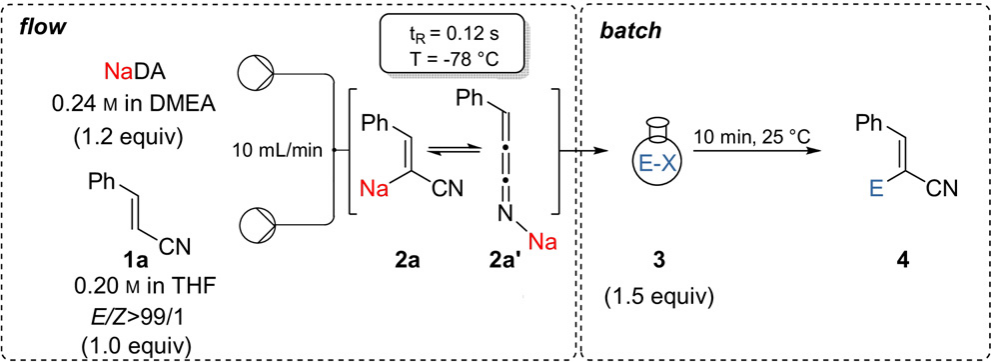

#	electrophile	product^[a]^	#	electrophile	product^[a]^
	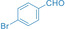	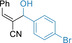		*n*Bu_2_S_2_	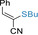
1	**3 a**	**4 aa**: 95 %, *Z*/*E*>99/1^[b]^	6	**3 f**	**4 af**: 93 %, *Z*/*E=*54/46
	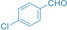	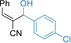		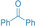	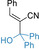
2	**3 b**	**4 ab**: 92 %, *Z*/*E*>99/1^[b]^	7	**3 g**	**4 ag**: 82 %, *E*/*Z*>99/1^[c]^
	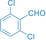	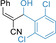		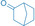	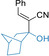
3	**3 c**	**4 ac**: 74 %, *Z*/*E=*89/11^[b]^	8	**3 h**	**4 ah**: 82 %, *E*/*Z*>99/1 *d.r*.>99/1^[c]^
	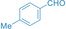	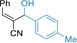		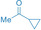	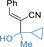
4	**3 d**	**4 ad**: 93 %, *Z*/*E>*99/1^[c]^	9	**3 i**	**4 ai**: 78 %, *E*/*Z*>99/1^[b]^
	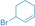	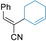		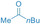	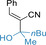
5	**3 e** ^[d]^	**4 ae**: 93 %, *E*/*Z=*9/1^[b]^	10	**3 j**	**4 aj**: 87 %, *E*/*Z*>99/1^[b]^

[a] Yield of analytically pure product. [b] The *E*‐ or *Z*‐ diastereoselectivity was assigned in analogy to related products, for which X‐ray data were obtained. [c] The diastereoselectivity was determined by crystal structure analyses, see Supporting Information. [d] 10 mol % CuCN⋅2 LiCl.

The diastereoselectivity of products of type **4** obtained after the addition to a carbonyl electrophile was tentatively explained by assuming that the sodiated nitrile **2 a** reacted fast with an aldehyde (RCHO) according to pathway A leading to the allylic alcohol ***Z*** **‐4**. In contrast, by using ketones, an equilibration to the cummulene form **2 a′** may occur and the cyclic transition state **A** would be disfavoured due to steric hindrance. *E*/*Z* isomerization of the cummulene structure **2 a′** occurred affording the ***E*** **‐4** product via transition state **B** (Scheme 1).

**Scheme 1:**
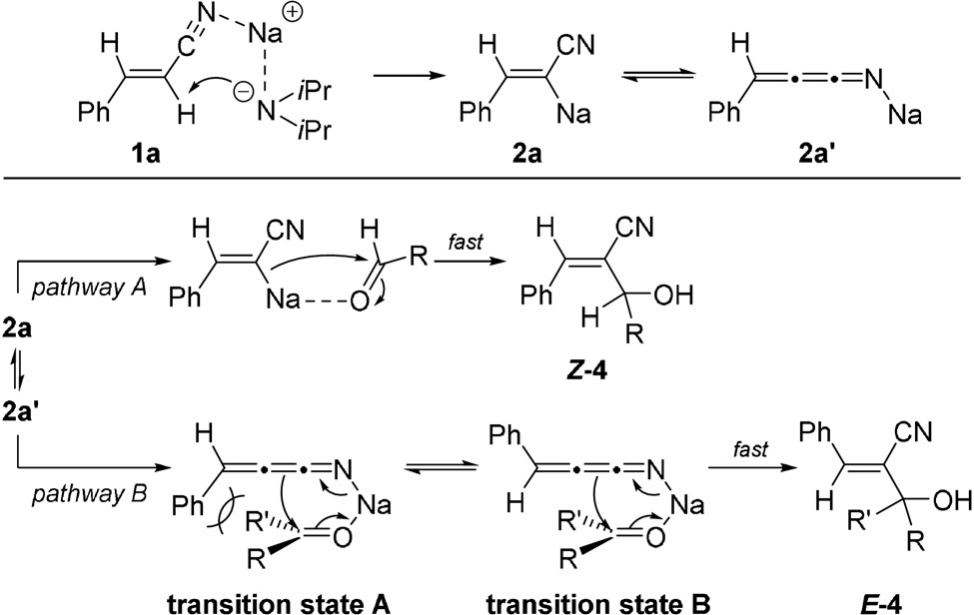
Tentative mechanism for the stereoselective addition of sodiated phenylacrylonitrile **2a** or **2 a′** to aldehydes or ketones.

We have then extended this flow procedure to various functionalized arylacrylonitriles of type **5**. Electron‐rich cinnamonitrile derivatives (**5 a**–**5 d**) were selectively metalated in 2‐position using NaDA in a continuous flow set‐up within 0.12 s at −78 °C. The resulting organosodiums (**6 a**–**d**) were trapped in batch with various carbonyl electrophiles, such as *m*‐anisaldehyde (**3 k**), cyclohexanecarboxaldehyde (**3 l**) or cyclohexanone (**3 m**), and with 3‐bromocyclohexene (**3 e**) using 10 mol % CuCN⋅2LiCl as catalyst, affording the desired alcohols (**7 ak, 7 bk**, **7 cl** and **7 dm**) and an allylated cinnamonitrile derivative (**7 de**) in 57–97 % yield with diastereomeric ratios up to >99/1 (Table 2, entries 1–5). Similarly, regioselective sodiation of electron‐deficient 3‐(4‐(trifluoromethyl)phenyl)acrylonitrile (**5 e**) followed by copper‐catalyzed allylation with 3‐bromocyclohexene (**3 e**) led to the functionalized phenylacrylonitrile (**7 ee**) in 66 % yield with an *E*/*Z* ratio >99/1. Furthermore, an extension to methoxy‐ and ethoxyacrylonitriles **5 f** and **5 g** was possible resulting in secondary alcohols (**7 fn**, **7 fd**, **7 gc** and **7 gl**) after batch‐quench with aromatic aldehydes (**3 c**, **3 d** and **3 n**), and aliphatic aldehyde (**3 l**) in 91–98 % and *Z*/*E* ratios >99/1 (entries 7–10). An alkenyl sulfide such as phenyl(styryl)sulfane (**5 h**) provided the sodium derivative (**6 h)** upon metalation with NaDA, which after trapping with sterically demanding ketones such as adamantanone (**3 o**) and benzophenone (**3 g**) gave tertiary alcohols (**7 ho** and **7 hg**) in 85–95 % yield and comparable *E*/*Z* ratios to the starting material **5 h** (entries 11–12).

**Table 2:** Sodiation of substituted acrylonitriles and alkenyl sulfides of type **5** using a microflow reactor and subsequent batch quench of the intermediate sodium organometallics of type **6** with various electrophiles of type **3** leading to functionalized phenylacrylonitriles and alkenyl sulfides of type **7**. 
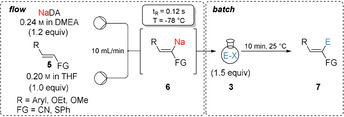

#	SM	product^[a]^	#	SM	product^[a]^
	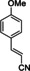	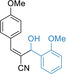		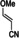	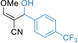
1	**5 a** *E*/*Z=* 76/24	**7 ak**: 97 %^[b]^ *Z*/*E=*89/11	7	**5 f** *E*/*Z=* 83/17	**7 fn**: 93 %^[c]^ *Z*/*E*>99/1
	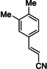	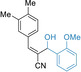		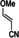	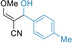
2	**5 b** *E*/*Z=* 79/21	**7 bk**: 84 %^[c]^ *Z*/*E=*90/10	8	**5 f** *E*/*Z=* 83/17	**7 fd**: 98 %^[c]^ *Z*/*E*>99/1
	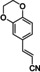	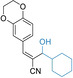		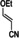	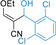
3	**5 c** *E*/*Z=* 83/17	**7 cl**: 74 %^[b]^ *Z*/*E*>99/1	9	**5 g** *E*/*Z=* 68/32	**7 gc**: 95 %^[c]^ *Z*/*E*>99/1
	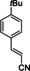	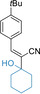		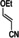	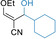
4	**5 d** *E*/*Z=* 79/21	**7 dm**: 67 %^[c]^ *E*/*Z*>99/1	10	**5 g** *E*/*Z=* 68/32	**7 gl**:91 %^[b]^ *Z*/*E*>99/1
	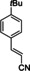	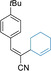		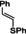	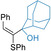
5	**5 d** ^[d]^ *E*/*Z=* 79/21	**7 de**: 57 %^[b]^ *E*/*Z*>99/1	11	**5 h** *E*/*Z=* 71/29	**7 ho**: 95 %^[c]^ *E*/*Z=*77/23
	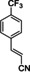	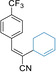		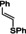	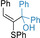
6	**5 e** ^[d]^ *E*/*Z=* 78/22	**7** ***ee***: 66 %^[c]^ *E*/*Z*>99/1	12	**5 h** *E*/*Z=* 71/29	**7 hg**: 85 %^[b]^ *E*/*Z=*68/32

[a] Yield of analytically pure product. [b] The *E*‐ or *Z*‐ diastereoselectivity was assigned in analogy to related products, for which X‐ray data were obtained. [c] The diastereoselectivity was determined by crystal structure analyses, see Supporting Information. [d] 10 mol % CuCN⋅2 LiCl.

Extension to alkyl‐substituted acrylonitriles such as geranylnitrile (**8 a**, *E*/*Z=*50/50) and the related nitrile **8 b** (*E*/*Z=*65/35) was possible under the standard sodiation conditions providing after electrophilic quench the desired functionalized nitriles (**10 ap**, **10 al**, **10 aq**, **10 br**) in 60–98 % yield as *E*/*Z* mixtures (Table 3, entries 1–4). Interestingly, starting from the diastereomerically pure acrylonitrile **8 c** (*E*/*Z*>99/1) the desired product **10 cq** was obtained in 67 % yield (*Z*/*E*=58/42) after quench with α‐tetralone (**3 q**) (entry 5) showing the prevalence of the cumulene structure of the sodiated nitriles (see **2 a’** in Table 1). However, the methoxy‐substituted acrylonitrile **8 d** (*E*/*Z=*80/20) afforded after continuous flow sodiation and quenching with *o*‐anisaldehyde (**3 k**) the allylic alcohol **10 dk** as single diastereoisomer in 58 % yield (*Z*/*E*>99/1) showing the importance of the methoxy group for controlling the stereochemistry of the intermediate sodiated nitrile (entry 6). Also, the dienylnitrile **8 e** was sodiated in flow and trapping with an allylic bromide (**3 e**) or an aldehyde (**3 k**) furnished the functionalized dienylnitriles (**10 ee** and **10 ek**) in 74–82 % yield (entries 7–8).

**Table 3:** Sodiation of alkyl‐ and alkenyl‐substituted acrylonitriles of type **8** using a microflow reactor and subsequent batch quench of the intermediate sodium organometallics of type **9** with various electrophiles of type **3** leading to functionalized alkyl‐ and alkenyl‐substituted acrylonitriles of type **10**.

#	substrate	electrophile	product^[a]^
	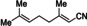	I_2_	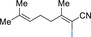
1	**8 a**, *E*/*Z=*50/50	**3 p**	**10 ap**: 75 %, *Z*/*E=*68/32
	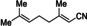	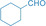	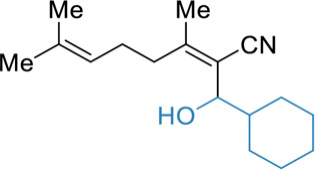
2	**8 a**, *E*/*Z=*50/50	**3 l**	**10 al**: 60 %, *Z*/*E=*64/36
	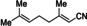	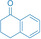	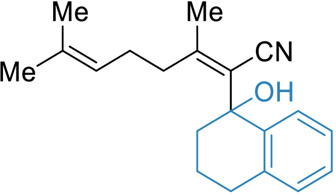
3	**8 a**, *E*/*Z=*50/50	**3 q**	**10 aq**: 98 %, *Z*/*E=*53/47
	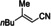	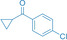	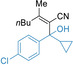
4	**8 b**, *E*/*Z=*65/35	**3 r**	**10 br**: 85 %, *Z*/*E=*55/45
	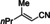	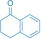	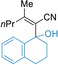
5	**8 c**, *E*/*Z*>99/1	**3 q**	**10 cq**: 67 %, *Z*/*E=*58/42
	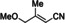	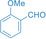	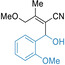
6	**8 d**, *E*/*Z=*80/20	**3 k**	**10 dk**: 58 %, *Z*/*E*>99/1
	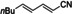	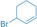	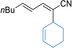
7	**8 e**, *2E/2Z*=69/31	**3 e** ^[b]^	**10** ***ee***: 74 %, *2E/2Z=*77/23
	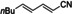	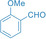	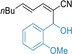
8	**8 e**, *2E/2Z*=69/31	**3 k**	**10 ek**: 82 %. *2Z/2E*=76/24

[a] Yield of analytically pure product. [b] 10 mol % CuCN⋅2 LiCl.

Recently, Takai and Asako published a straightforward synthesis of lithium‐free sodium 2,2,6,6‐tetramethylpiperidide (NaTMP) in *n*‐hexane by using sodium dispersion, TMPH, TMEDA and isoprene.^[12]^ This method would allow us to avoid the use of the amine DMEA as solvent and therefore making our method more practical. Using the Takai procedure, we have prepared hexane‐soluble NaTMP⋅TMEDA^[13]^ and have performed an efficient continuous flow sodiation of cinnamonitrile (**1 a**) selectively in 2‐position within 0.12 s at −78 °C. A subsequent batch trapping of **2 a′** with various ketones of type **3** afforded the desired tertiary alcohols of type **4** in 58–83 % yield as single regioisomers (Scheme 2). Similarly, ethoxyacrylonitrile **5 g** gave, after batch quench with *o*‐anisaldehyde (**3 k**) and benzophenone (**3 g**), the allylic alcohols (**7 gk** and **7 gg**) in 65–78 % yield (*Z*/*E*>99/1). Further, geranylnitrile (**8 a**) provided the organosodium **9 a** upon metalation with NaTMP⋅TMEDA, which after a copper‐catalyzed allylation using 3‐bromocyclohexene (**3 e**) led to the desired product (**10 ae**) in 54 % yield with a *E*/*Z* ratio of 52/48.

**Scheme 2:**
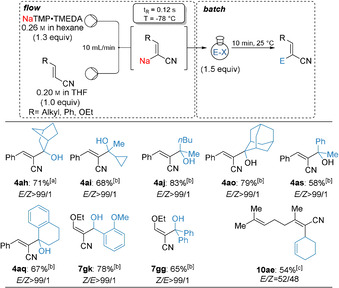
General set‐up for the sodiation of functionalized acrylonitriles with NaTMP⋅TMEDA in a microflow reactor and subsequent batch quench of the intermediate sodium organometallics with various electrophiles leading to functionalized acrylonitriles. [a] The diastereoselectivity was determined by crystal structure analyses, see Supporting Information. [b] The *E*‐ or *Z*‐ diastereoselectivity was assigned in analogy to related products, for which X‐ray data were obtained. [c] 10 mol % CuCN⋅2 LiCl.

However, the sodiation of other acrylates still remained challenging. Applying our standard sodiation method to ethyl cinnamate (**11 a**) afforded solely the condensation product **13 a** showing that the sodiation of **11 a** was possible, but difficult to control. Thus, the intermediate organosodium **12 a** reacted instantaneously with another molecule of **11 a** before the desired electrophile quench proceeded (Scheme 3 a). To prevent this self‐condensation reaction, sterically hindered *tert*‐butyl cinnamate (**11 b**) was used affording organosodium **12 b** after continuous flow sodiation. A copper‐catalyzed batch allylation with 3‐bromocyclohexene (**3 e**) gave the desired product **13 be** in 61 % yield with an *E*/*Z* ratio >99/1 (Scheme 3 b). To overcome the need of sterically hindered esters, we envisioned a Barbier‐type in situ trapping^[14]^ of the highly reactive organosodiums of type **12**. Interestingly, ethyl cinnamate (**11 a**), which underwent self‐condensation side reactions applying our standard flow conditions (Scheme 3 a), was sodiated at −78 °C under Barbier‐conditions and afforded organosodium **12 a**, which was instantaneously trapped by adamantanone (**3 o**), outcompeting self‐condensation and resulting in the tertiary alcohol **13 ao** in 66 % yield (*E*/*Z*>99/1). Similarly, methyl‐3‐methoxyacrylate (**11 c**) was sodiated in 3‐position in the presence of adamantanone (**3 o**) using NaDA (1.2 equiv) affording the spirolactone **13 co** in 58 % yield (Scheme 3 c).

**Scheme 3:**
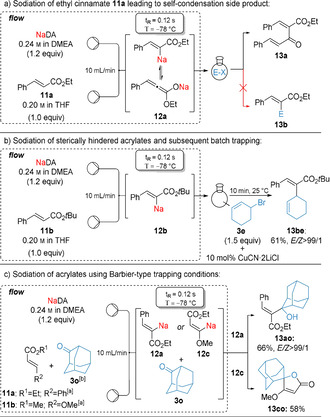
Sodiation of substituted acrylates of type **11** using a microflow reactor under standard‐flow conditions and Barbier conditions. *In situ* quench of the intermediate sodium organometallics of type **12** with adamantanone (**3 o**) afforded functionalized acrylates of type **13**. [a] 0.20 m in THF, 1.0 equiv [b] 0.30 m in THF, 1.5 equiv.

In summary, we have reported the sodiation of substituted acrylonitriles and alkenyl sulfides in a continuous flow set‐up using NaDA (sodium diisopropylamide) in EtNMe_2_ (DMEA) and NaTMP (sodium 2,2,6,6‐tetramethylpiperidide)⋅TMEDA in *n*‐hexane. The resulting sodiated acrylonitriles and alkenyl sulfides were subsequently trapped in batch with various electrophiles such as aldehydes, ketones, disulfides and allylic bromides affording functionalized acrylonitriles and alkenyl sulfides. This flow‐procedure was successfully extended to other acrylates by using Barbier‐type conditions.

## Conflict of interest

The authors declare no conflict of interest.

## Supporting information

As a service to our authors and readers, this journal provides supporting information supplied by the authors. Such materials are peer reviewed and may be re‐organized for online delivery, but are not copy‐edited or typeset. Technical support issues arising from supporting information (other than missing files) should be addressed to the authors.

Supplementary

Supplementary
