# Supplementary material for: Continuous Flow Sodiation of Substituted Acrylonitriles, Alkenyl Sulfides and Acrylates
Source: Angew Chem Int Ed Engl. 2020 Nov 3;60(2):731–5. doi: 10.1002/anie.202012085 (PMC7821005; doi:10.1002/anie.202012085)
Supplement: Supplementary file 2 — Supplementary [file ANIE-60-731-s002.pdf]

Angewandte Chemie
Eine Zeitschrift der Gesellschaft Deutscher Chemiker

Supporting Information

Continuous Flow Sodiation of Substituted Acrylonitriles, Alkenyl Sulfides and Acrylates

*Johannes H. Harenberg[+], Niels Weidmann[+], Konstantin Karaghiosoff, and Paul Knochel**

anie_202012085_sm_miscellaneous_information.pdf
anie_202012085_sm_cif.zip

Table of Contents

General information 2

Solvents 2

Reagents 2

Chromatography... 2

Analytical data.. 3

Typical procedure... 3

Screenig table 9

Preparation of the products... 10

Preparation of the starting materials 50

NMR data 63

Single crystall X-ray diffraction studies 117

General information

Solvents

THF was continuously refluxed and freshly distilled from sodium benzophenone ketyl under nitrogen and stored over molecular sieves.

DMEA was continuously refluxed and freshly distilled from sodium benzophenone ketyl under argon and stored under argon.

Solvents for column chromatography were distilled prior to use.

Reagents

All reagents were obtained from commercial sources and used without further purification unless otherwise stated.

NaDA solution (ca. 1.0 M in DMEA) was prepared according to a slightly modified procedure reported by Collum.[1] Optimizated reaction conditions are as follows: Sodium dispersion (5 mL, 58.3 mmol, 30 wt% in toluene, <0.1 mm particle size) was washed with dry DMEA (3×2 mL). Then, dry DMEA (14.4 mL) and dry di*iso*propyl amine (4.2 mL, 29.8 mmol, 1.0 equiv) were added. After cooling the solution to 0 °C, isoprene (1.52 mL, 15.0 mmol, 0.5 equiv) was added dropwise and the solution was allowed to warm to 25 °C over 2 h. The concentration of the resulting yellow NaDA solution was determined by titration with diphenyl acetic acid.

TMPNa solution (ca. 1.0 M in hexane) was prepared according to a slightly modified procedure reported by Takai.[2] Sodium dispersion (1.5 mL, 17.5 mmol, 1.4 equiv, 30 wt% in toluene, <0.1 mm particle size) was washed with dry hexane (3×2 mL). Then, dry hexane (5 mL) and 2,2,6,6-tetramethylpiperidine (TMPH, 2.1 mL, 12.5 mmol, 1.0 equiv) were added. After cooling the solution to 0 °C, isoprene (1.25 mL, 12.5 mmol, 1.0 equiv) was added dropwise and the solution was allowed to warm to 25 °C over 2 h. The concentration of the resulting black TMPNa solution was determined by titration with diphenyl acetic acid.

CuCN·2LiCl[3] solution (1.0 M in THF) was prepared by drying CuCN (8.96 g, 100 mmol) and LiCl (8.48 g, 200 mmol) in a Schlenk flask under vacuum for 5 h at 150 °C. After cooling to 25 °C, dry THF (100 mL) was added and stirred until the salts were dissolved.

Chromatography

Flash column chromatography was performed using SiO_2 60 (0.040-0.063 mm, 230-400 mesh ASTM) from Merck. Thin layer chromatography (TLC) was performed using aluminum plates covered with SiO_2 (Merck 60, F-254). Spots were visualized under UV light.

---

[1] Y. Ma, R. F. Algera, D. B. Collum, *J. Org. Chem.* **2016**, *81*, 11312.
[2] S. Asako, M. Kodera, H. Nakajima, K. Takai, *Adv. Synth. Catal.* **2019**, *361*, 3120.
[3] P. Knochel, M. C. P. Yeh, S. C. Berk, J. Talbert, *J. Org. Chem.* **1988**, *53*, 2390.

Analytical data

Yields refer to isolated yields of compounds estimated to be >95% pure as determined by ^{1}H-NMR (25 °C) and capillary GC-analyses. NMR spectra were recorded on Bruker ARX 200, AC 300, WH 400 or AMX 600 instruments. Chemical shifts are reported as δ-values in ppm relative to the deuterated solvent peak: $CDCl_3$ (δH: 7.26; δC: 77.16). For the observation of the observed signal multiplicities, the following abbreviations were used: s (singlet), d (doublet), dd (doublet of doublets), t (triplet), q (quartet), quint (quintet), sext (sextet), sept (septet) and m (multiplet). Melting points are uncorrected and were measured on a Büchi B.540 apparatus. Infrared spectra were recorded from 4000-400 cm^{-1} on a Nicolet 510 FT-IR or a Perkin-Elmer 281 IR spectrometer. Samples were measured at a Smiths Detection DuraSampl IR II Diamond ATR. The absorption bands are reported in wavenumbers (cm^{-1}). Gas chromatography (GC)-analysis was performed with instruments of the type Hewlett-Packard 6890 or 5890 Series II, using a column of the type HP 5 (Hewlett-Packard, 5% phenylmethylpolysiloxane; length: 10 m, diameter: 0.25 mm, film thickness: 0.25 μm). The detection was accomplished using a flame ionization detector. Mass spectra (MS) and high resolution mass spectra (HRMS) were recorded on a Finnigan MAT95Q or Finnigan MAT90 instrument for electron impact ionization (EI) and electrospray ionization (ESI). For the combination of gas chromatography with mass spectroscopic detection, a GC-MS of the type Hewlett-Packard 6890 / MSD 5793 networking was used (column: HP 5-MS, Hewlett-Packard; 5% phenylmethylpolysiloxane; length: 15 m, diameter 0.25 mm; film thickness: 0.25 μm).

Typical procedure

General remarks on flow and subsequent batch quenching reactions

Tetradecane ($n\mathrm{C}_{14}\mathrm{H}_{30}$), tridecane ($n\mathrm{C}_{13}\mathrm{H}_{28}$), dodecane ($n\mathrm{C}_{12}\mathrm{H}_{26}$) or undecane ($n\mathrm{C}_{11}\mathrm{H}_{24}$) were used as internal standards. All flasks were heat gun dried (650 °C) under vacuum and backfilled with argon after cooling. Syringes, which were used to transfer reagents and solvents, were purged with argon three times prior to use. Batch quenching reactions were carried out with magnetic stirring. Flow reactions were performed on the commercially available flow system FlowSyn from Uniqsis. Carrier solvents as well as reactant solutions were stored under argon and injected to carrier solvent streams. All reactions were performed in coiled tube reactors. Coiled reactors were made from PFA or PTFE Teflon (I.D. = 0.8 mm or 0.25 mm, O.D. = 1.6 mm) tubing and T-pieces (I.D. = 0.5 mm) were used as mixers. Prior to performing reactions, the systems were dried by flushing with dry THF (flow-rate of all pumps: 1.0 mL·min^{-1}; run-time: 20 min).

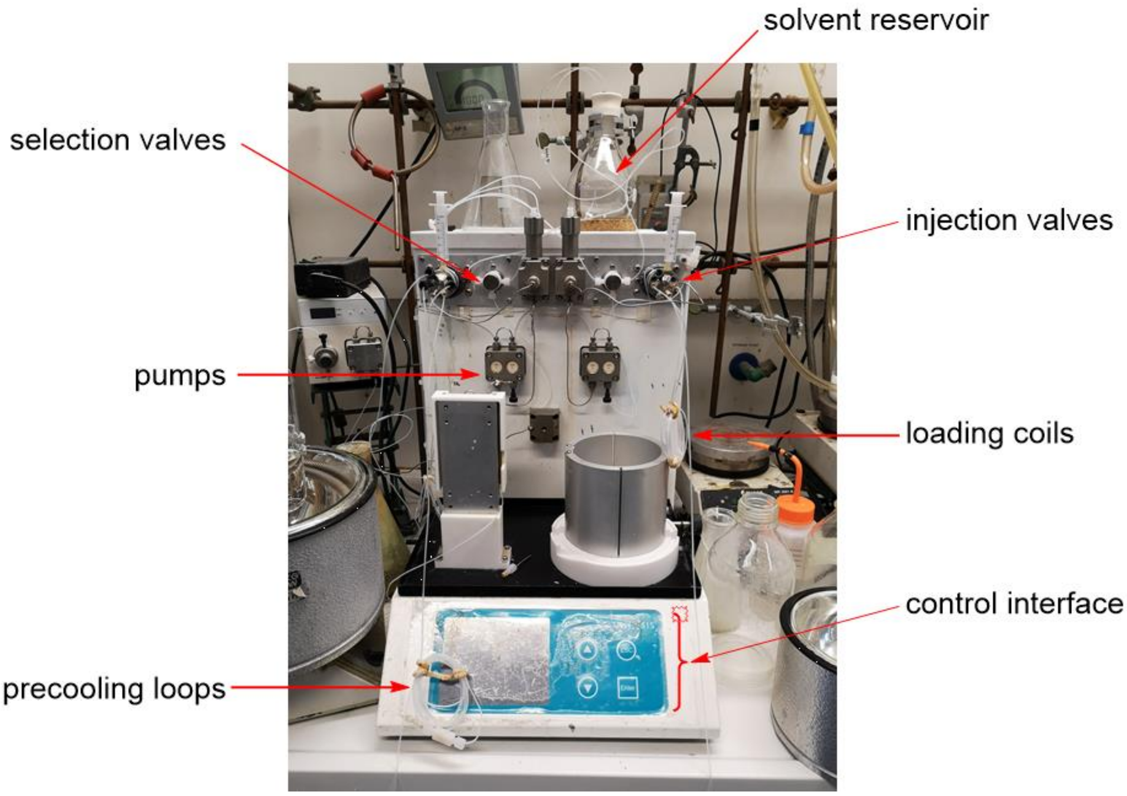

Graphic 1: Picture of the standard continuous flow reaction set-up.

Typical procedure 1 using a Uniqsis flow setup (Scheme SI 1): Sodiation of (substituted) acrylonitriles and alkenes using NaDA and subsequent batch quench with various electrophiles leading to functionalized acrylonitriles and alkenes.

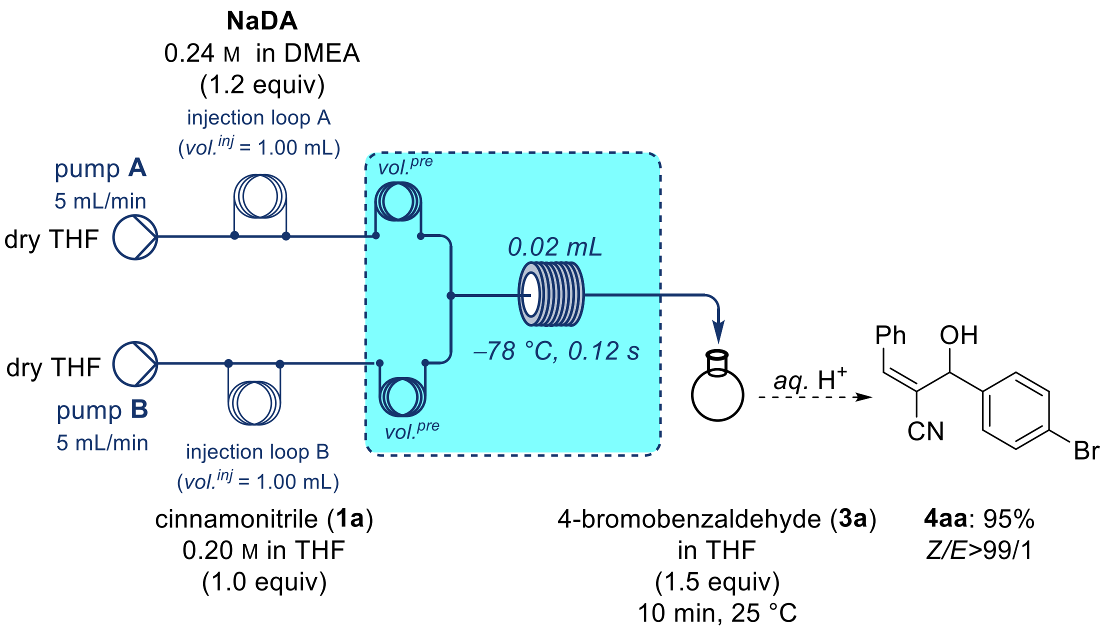

Scheme SI 1: Uniqsis flow setup for the sodiation of cinnamonitrile (**1a**) using a microflow reactor and subsequent batch quench of the intermediate organosodium **2a** with 4-bromobenzaldehyde (**3a**) leading to secondary alcohol **4aa**.

A NaDa solution (0.24 M, 1.2 equiv) in DMEA and a solution of cinnamonitrile (**1a**, 0.20 M, 26 mg, 1.0 equiv) in THF were prepared. Injection loop A (vol^{inj} =1.0 mL) was loaded with the NaDA solution and injection loop B (vol^{inj} =1.0 mL) was loaded with the solution of cinnamonitrile (**1a**). The solutions were simultaneously injected into separate streams of THF (flow-rates: 5 mL·min^{-1}), which each passed a pre-cooling loop (vol^{pre} = 1.0 mL, T^1 = –78 °C, residence time: 12 s), before they were mixed in a T-mixer (PTFE, I.D. = 0.5 mm). The combined stream passed a PTFE reactor tube (vol^R = 0.02 mL; residence time: t^1 = 0.12 s, T^1 = –78 °C) and was subsequently injected in a flask containing a stirred, solution of 4-bromobenzaldehyde (**3a**, 56 mg, 0.30 mmol, 1.5 equiv) in THF. The reaction mixture was further stirred for 10 minutes at 25 °C and quenched with a *sat. aq.* NH_4Cl solution. The aqueous phase was extracted with EtOAc and the organic phases were dried and filtrated. After removal of the solvent *in vacuo*, flash chromatographical purification (silica gel, isohexane:EtOAc = 9:1) afforded the title compound **4aa** as colorless crystals (64 mg, 0.19 mmol, 95% yield; *Z/E* > 99/1).

Typical procedure 2 using a Uniqsis flow setup (Scheme SI 2): Sodiation of (substituted) acrylonitriles and alkenes using NaTMP and subsequent batch quench with various electrophiles leading to functionalized acrylonitriles and alkenes.

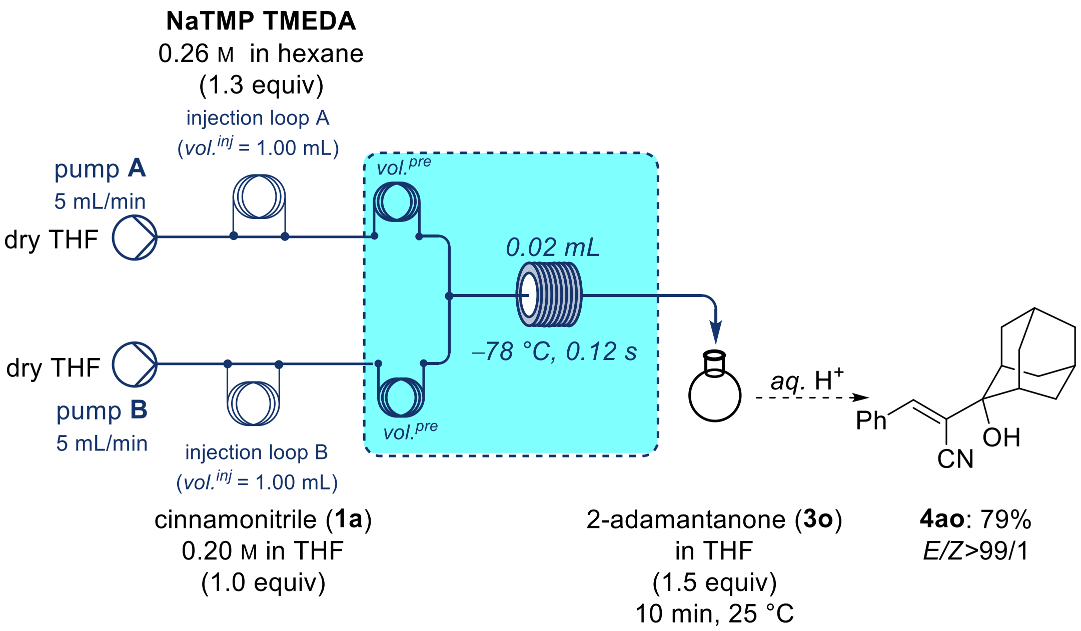

Scheme SI 2: Uniqsis flow setup for the sodiation of cinnamonitrile (**1a**) using a microflow reactor and subsequent batch quench of the intermediate organosodium **2a** with 2-adamantanone (**3o**) leading to tertiary alcohol **4ao**.

A NaTMP solution (0.26 M, 1.2 equiv) in hexane and a solution of cinnamonitrile (**1a**, 0.20 M, 26 mg, 1.0 equiv; *E/Z* > 99/1) in THF were prepared. Injection loop A (vol^{inj} =1.0 mL) was loaded with the NaTMP solution and injection loop B (vol^{inj} =1.0 mL) was loaded with the solution of cinnamonitrile (**1a**). The solutions were simultaneously injected into separate streams of THF (flow-rates: 5 $mL \cdot min^{-1}$), which each passed a pre-cooling loop (vol^{pre} = 1.0 mL, T^1 = –78 °C, residence time: 12 s), before they were mixed in a T-mixer (PTFE, I.D. = 0.5 mm). The combined stream passed a PTFE reactor tube (vol^R = 0.02 mL; residence time: t^1 = 0.12 s, T^1 = –78 °C) and was subsequently injected in a flask containing a stirred, solution of 2-adamantanone (**3o**, 45 mg, 0.30 mmol, 1.5 equiv) in THF. The reaction mixture was further stirred for 10 minutes at 25 °C and quenched with a *sat. aq.* NH_4Cl solution. The aqueous phase was extracted with EtOAc and the organic phases were dried and filtrated. After removal of the solvent *in vacuo*, flash chromatographical purification (silica gel, isohexane:EtOAc = 9:1) afforded the title compound **4ao** as colorless oil (44 mg, 0.16 mmol, 79% yield; *E/Z* > 99/1).

Typical procedure 3 using a Uniqsis flow setup (Scheme SI 3): Sodiation of (substituted) acrylonitriles and alkenes using NaDA and under Barbier-type condtitions with various electrophiles leading to functionalized acrylonitriles and alkenes.

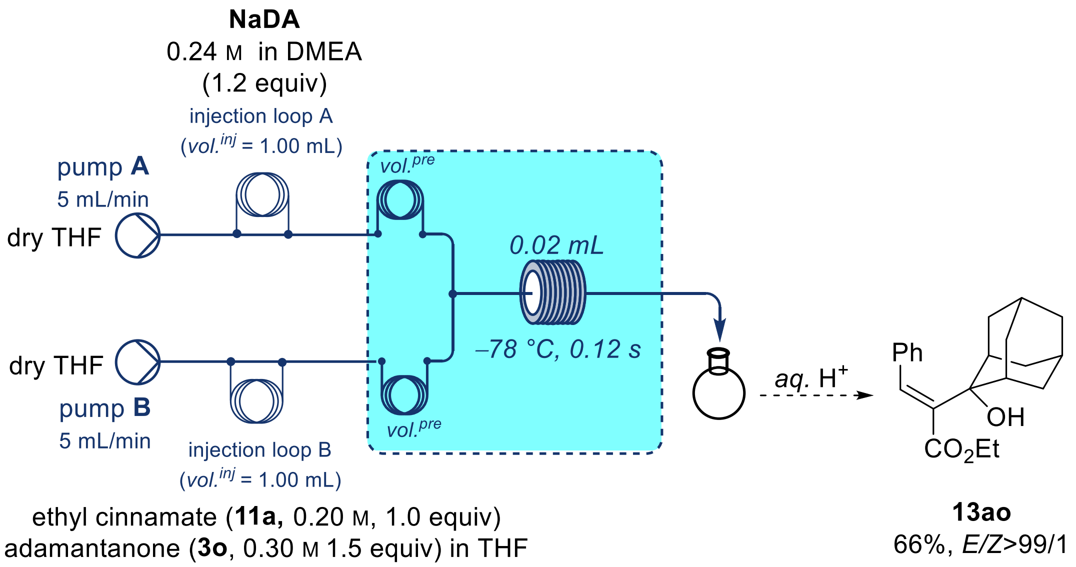

Scheme SI 3: Uniqsis flow setup for the sodiation of ethyl cinnamate (**11a**) using a microflow reactor under Barbier-type conditions with adamantanone (**3o**) leading to tertiary alcohol **13ao**.

A solution of ethyl cinnamate (**11a**, 0.20 M, 35 mg, 0.20 mmol, 1.0 equiv; *E*/*Z* > 99/1) and adamantanone (**3o**, 45 mg, 0.30 mmol, 1.5 equiv) in THF (total volume: 1 mL) and a solution of NaDA (0.24 M in DMEA, 0.24 mmol, 1.2 equiv) were prepared. Injection loop A (vol^{inj} =1.0 mL) was loaded with the NaDA solution and injection loop B (vol^{inj} =1.0 mL) was loaded with the solution of ethyl cinnamate (**11a**) and adamantanone (**3o**). The solutions were simultaneously injected into separate streams of THF (flow-rates: 5 mL·min^{-1}), which each passed a pre-cooling loop (vol^{pre} = 1.0 mL, T^1 = –78 °C, residence time: 12 s), before they were mixed in a T-mixer (PTFE, I.D. = 0.5 mm). The combined stream passed a PTFE reactor tube (vol^R = 0.02 mL; residence time: t^1 = 0.12 s, T^1 = –78 °C) and was subsequently injected in a flask containing a stirrbar. The reaction was stirred for 30 min at −78 °C and quenched by the addition of *sat. aq.* NH_4Cl. The aqueous phase was extracted three times with EtOAc (3×10 mL) and the combined organic phases were dried over anhydrous $MgSO_4$ and filtrated. After removal of the solvent *in vacuo*, flash chromatographical purification (silica gel) afforded the title compound **13ao** as white solid (43 mg, 0.13 mmol, 66% yield; *E*/*Z* > 99/1).

Typical procedure 4 using batch conditions: Sodiation of (substituted) acrylonitriles and alkenes using NaDA and under batch condtitions with various electrophiles leading to functionalized acrylonitriles and alkenes.

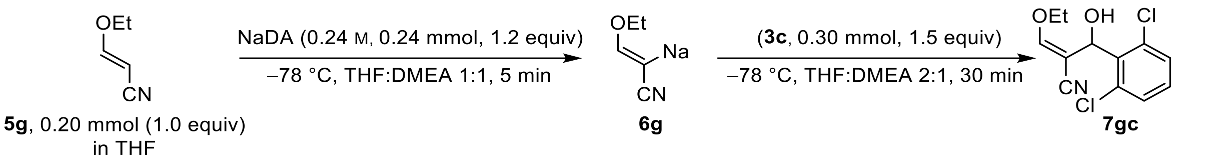

Scheme SI 4: Sodiation of cinnamonitrile (**1a**) under batch conditions subsequent quench with 4-chlorobenzaldehyde (**3b**) leading to tertiary alcohol **4ab**.

To a solution of (*E*)-ethoxyacrylonitrile (**5g**, 0.20 M, 0.20 mmol, 1.0 equiv; *E*/*Z* > 99/1) in THF (total volume: 1 mL) at −78 °C was added a solution of NaDA (0.24 M in DMEA, 0.24 mmol, 1.2 equiv). The mixture was stirred for 5 min at −78 °C. After 5 min a solution of 2,6-dichlorobenzaldehyde (**3c**, 53 mg, 0.30 mmol, 1.5 equiv) in THF (1.0 mL) was added at the same temperature. After stirring for 30 min at −78 °C the mixture was quenched by the addition of *sat. aq.* NH_4Cl. The aqueous phases were dried over anhydrous $MgSO_4$ and filtrated. After removal of the solvent *in vacuo*, flash chromatographical purification (silica gel) afforded the title compound **7gc** as a colorless solid (38 mg, 0.14 mmol, 72% yield; *Z/E* > 99/1).

Screenig table

Table SI 1: Screening of flow conditions for the preparation of **4ab**.

Entry	T [°C]	Combined flow-rate [mL/min]	Reactor volume [mL]	Residence time [s]	Conversion [%]	GC yield[a] [%]
1	0	2	0.02	0.6	100	19
2	0	2	1	30	100	37
3	0	10	0.02	0.12	100	29
4	0	10	1	6	100	30
5	0	10	4	24	100	39
6	-40	2	0.02	0.6	100	17
7	-40	2	1	30	100	14
8	-40	2	4	120	100	12
9	-40	10	0.02	0.12	100	74
10	-40	10	1	6	100	65
11	-40	10	4	24	100	44
12	-78	2	0.02	0.6	100	60
13	-78	2	1	30	100	26
14	-78	2	4	120	100	25
15	-78	10	0.02	0.12	100	95[b]
16	-78	10	1	6	100	50
17	-78	10	4	24	100	54

a) GC-yield normalized to isolated yield. b) isolated yield.

Preparation of the products

(*Z*)-2-((4-Bromophenyl)(hydroxy)methyl)-3-phenylacrylonitrile (4aa)

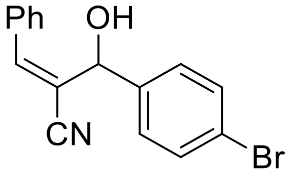

According to the **TP1**, a solution of cinnamonitrile (**1a**, 0.20 M, 26 mg, 0.20 mmol, 1.0 equiv; *E/Z* > 99/1) in THF (total volume: 1 mL) and a solution of NaDA (0.24 M in DMEA, 0.24 mmol, 1.2 equiv) were prepared. The precooled solutions were mixed with an overall 10 $mL\cdot min^{-1}$ flow-rate in a T-mixer. The combined stream passed a 0.02 mL reactor tube (0.12 s, –78 °C) and was subsequently injected in a flask containing a stirred solution of 4-bromobenzaldehyde (**3a**, 56 mg, 0.30 mmol, 1.5 equiv) in THF. The reaction was instantaneously quenched by the addition of *sat. aq.* NH_4Cl. The aqueous phase was extracted three times with EtOAc (3×10 mL) and the combined organic phases were dried over anhydrous $MgSO_4$ and filtrated. After removal of the solvent *in vacuo*, flash chromatographical purification (silica gel, isohexane:EtOAc = 9:1) afforded the title compound **4aa** as colorless crystals (64 mg, 0.19 mmol, 95% yield; *Z/E* > 99/1).

^{1}H-NMR (400 MHz, $CDCl_3$): δ / ppm = 7.57 – 7.53 (m, 2H), 7.48 (d, *J* = 0.8 Hz, 1H), 7.45 – 7.41 (m, 3H), 7.38 – 7.32 (m, 4H), 5.75 (s, 1H), 2.37 (s, 1H).

^{13}C-NMR (100 MHz, $CDCl_3$): δ / ppm = 146.2, 138.9, 133.1, 132.3 (2C), 130.4, 129.3 (2C), 129.2 (2C), 128.1 (2C), 123.1, 118.2, 118.1, 69.2.

IR (Diamond-ATR, neat): $\tilde{\nu}$ / cm^{-1} = 3480, 2213, 1616, 1591, 1574, 1488, 1447, 1399, 1391, 1361, 1292, 1283, 1248, 1218, 1194, 1159, 1136, 1108, 1074, 1030, 1012, 977, 946, 934, 906, 866, 847, 827, 778, 759, 699, 657.

MS (EI, 70 eV): *m/z* (%) = 315 (11), 313 (11), 187 (28) 185 (50), 183 (31), 157 (10), 140 (11), 130 (100), 129 (33), 105 (13), 102 (24), 78 (30), 77 (61), 76 (12), 75 (11), 51 (16), 43 (32).

HRMS (EI): *m/z* calc. for [$C_{16}H_{12}ONBr$]: 313.0102; found: 313.0087.

m.p. (°C): 134.3 – 136.3.

(*Z*)-2-((4-Chlorophenyl)(hydroxy)methyl)-3-phenylacrylonitrile (4ab)

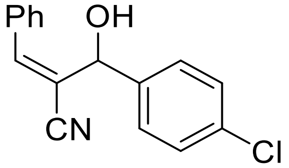

According to the **TP1**, a solution of cinnamonitrile (**1a**, 0.18 M, 23 mg, 0.18 mmol, 1.0 equiv; *E/Z* > 99/1) in THF (total volume: 1 mL) and a solution of NaDA (0.21 M in DMEA, 0.21 mmol, 1.2 equiv) were prepared. The precooled solutions were mixed with an overall 10 mL·min^{-1} flow-rate in a T-mixer. The combined stream passed a 0.02 mL reactor tube (0.12 s, –78 °C) and was subsequently injected in a flask containing a stirred solution of 4-chlorobenzaldehyde (**3b**, 42 mg, 0.30 mmol, 1.7 equiv) in THF. The reaction was instantaneously quenched by the addition of *sat. aq.* NH_4Cl. The aqueous phase was extracted three times with EtOAc (3×10 mL) and the combined organic phases were dried over anhydrous $MgSO_4$ and filtrated. After removal of the solvent *in vacuo*, flash chromatographical purification (silica gel, isohexane:EtOAc = 9:1) afforded the title compound **4ab** as colorless crystals (45 mg, 0.17 mmol, 92% yield; *Z/E* > 99/1).

^{1}H-NMR (400 MHz, $CDCl_3$): δ / ppm = 7.49 (s, 1H), 7.46 – 7.42 (m, 3H), 7.41 (d, *J* = 4.1 Hz, 3H), 7.33 (dt, *J* = 7.9, 3.5 Hz, 2H), 5.77 (s, 1H), 4.68 (s, 1H), 2.42 – 2.23 (m, 1H).

^{13}C-NMR (100 MHz, $CDCl_3$): δ / ppm = 146.2, 138.3, 130.4, 129.4 (2C), 129.3 (2C), 129.2 (2C), 128.8 (2C), 128.4, 127.8, 118.2, 69.1, 29.9.

IR (Diamond-ATR, neat): $\tilde{\nu}$ / cm^{-1} = 3398, 2922, 2852, 2219, 1616, 1596, 1575, 1490, 1464, 1456, 1447, 1404, 1091, 1042, 1013, 836, 799, 777, 755, 728, 697.

MS (EI, 70 eV): *m/z* (%) = 253 (16), 251 (50), 217 (17), 216 (100), 214 (27), 189 (32), 141 (13), 139 (36), 130 (26), 77 (17).

HRMS (EI): *m/z* calc. for [$C_{16}H_{12}NOCl$]: 269.0607; found: 251.0498 [M – H_2O]

m.p. (°C): 124.2 – 128.3.

2-((4-Chlorophenyl)(hydroxy)methyl)-3-phenylacrylonitrile (4ac)

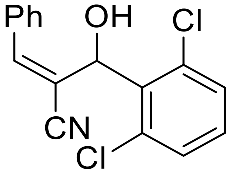

According to the **TP1**, a solution of cinnamonitrile (**1a**, 0.20 M, 26 mg, 0.20 mmol, 1.0 equiv; *E/Z* > 99/1) in THF (total volume: 1 mL) and a solution of NaDA (0.24 M in DMEA, 0.24 mmol, 1.2 equiv) were prepared. The precooled solutions were mixed with an overall 10 mL·min^{-1} flow-rate in a T-mixer. The combined stream passed a 0.02 mL reactor tube (0.12 s, –78 °C) and was subsequently injected in a flask containing a stirred solution of 2,6-dichlorobenzaldehyde (**3c**, 52 mg, 0.30 mmol, 1.5 equiv) in THF. The reaction was instantaneously quenched by the addition of *sat. aq.* NH_4Cl. The aqueous phase was extracted three times with EtOAc (3×10 mL) and the combined organic phases were dried over anhydrous $MgSO_4$ and filtrated. After removal of the solvent *in vacuo*, flash chromatographical purification (silica gel, isohexane:EtOAc = 9:1) afforded the title compound **4ac** as a colorless solid (45 mg, 0.15 mmol, 74% yield; *Z/E* = 89/11).

^{1}H-NMR (400 MHz, $CDCl_3$):

(*Z*)-2-((4-Chlorophenyl)(hydroxy)methyl)-3-phenylacrylonitrile: δ / ppm = 7.50 – 7.48 (m, 2H), 7.48 – 7.47 (m, 1H), 7.41 – 7.38 (m, 3H), 7.30 – 7.26 (m, 2H), 7.18 (dd, *J* = 8.7, 7.3 Hz, 1H), 6.27 – 6.20 (m, 1H), 3.83 (s, 1H).

(*E*)-2-((4-Chlorophenyl)(hydroxy)methyl)-3-phenylacrylonitrile: δ / ppm = 7.79 – 7.76 (m, 2H), 7.51 – 7.38 (m, 7H), 6.31 (d, *J* = 2.2 Hz, 1H), 2.69 (s, 1H).

^{13}C-NMR (100 MHz, $CDCl_3$):

(*Z*)-2-((4-Chlorophenyl)(hydroxy)methyl)-3-phenylacrylonitrile: δ / ppm = 148.8, 135.0 (2C), 134.3, 133.3, 130.3 (2C), 129.6 (2C), 129.5 (2C), 128.9 (2C), 118.7, 116.2, 68.5.

(*E*)-2-((4-Chlorophenyl)(hydroxy)methyl)-3-phenylacrylonitrile: δ / ppm = 143.3, 135.4, 134.3, 133.1, 130.7 (3C), 129.8 (2C), 129.2 (2C), 129.0 (2C), 116.8, 112.0, 77.1.

IR (Diamond-ATR, neat): $\tilde{\nu}$ / cm^{-1} = 3492, 3437, 3066, 2923, 2854, 2211, 1792, 1683, 1601, 1579, 1561, 1492, 1446, 1437, 1305, 1228, 1202, 1184, 1148, 1093, 1077, 1033, 983, 943, 890, 826, 793, 782, 767, 756, 720, 696.

MS (EI, 70 eV): *m/z* (%) = 285 (12), 281 (15), 250 (26), 227 (10), 226 (10), 225 (76), 214 (19), 209 (34), 208 (10), 207 (7 8), 191 (16), 177 (10), 175 (69), 174 (10), 173 (100), 130 (11), 102 (11), 78 (12), 75 (12).

HRMS (EI): *m/z* calc. for [$C_{16}H_{11}Cl_2NO$]: 303.0218; found: 303.0218.

m.p. (°C): 86.0 – 88.1.

(*Z*)-4-Hydroxy-3-phenyl-4-(*p*-tolyl)but-2-enenitrile (4ad)

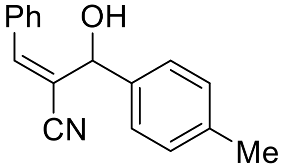

According to the **TP1**, a solution cinnamonitrile (**1a**, 0.20 M, 26 mg, 0.20 mmol, 1.0 equiv; *E/Z* > 99/1) in THF (total volume: 1 mL) and a solution of NaDA (0.24 M in DMEA, 0.24 mmol, 1.2 equiv) were prepared. The precooled solutions were mixed with an overall 10 mL·min^{-1} flow-rate in a T-mixer. The combined stream passed a 0.02 mL reactor tube (0.12 s, –78 °C) and was subsequently injected in a flask containing a stirred solution of 4-methylbenzaldehyde (**3d**, 36 mg, 0.30 mmol, 1.5 equiv) in THF. The reaction was instantaneously quenched by the addition of *sat. aq.* NH_4Cl. The aqueous phase was extracted three times with EtOAc (3×10 mL) and the combined organic phases were dried over anhydrous $MgSO_4$ and filtrated. After removal of the solvent *in vacuo*, flash chromatographical purification (silica gel, isohexane:EtOAc = 9:1) afforded the title compound **4ad** as pale yellow crystals (46 mg, 0.19 mmol, 93% yield; *Z*/*E* > 99/1).

^{1}H-NMR (400 MHz, $CDCl_3$): δ / ppm = 7.45 – 7.37 (m, 6H), 7.35 – 7.31 (m, 2H), 7.23 (d, *J* = 8.0 Hz, 2H), 5.73 (d, *J* = 5.2 Hz, 1H), 2.38 (s, 3H).
^{13}C-NMR (100 MHz, $CDCl_3$): δ / ppm = 145.5, 138.9, 137.1, 133.3, 130.1, 129.9 (2C), 129.4 (2C), 129.0 (2C), 126.3 (2C), 118.8, 118.5, 69.6, 21.3.
IR (Diamond-ATR, neat): $\tilde{\nu}$ / cm^{-1} = 3498, 3026, 2918, 2855, 2214, 1613, 1574, 1513, 1491, 1446, 1390, 1358, 1320, 1304, 1246, 1213, 1192, 1181, 1159, 1134, 1122, 1078, 1040, 1021, 1000, 950, 931, 896, 866, 843, 826, 796, 755, 739, 697, 666.
MS (EI, 70 eV): *m/z* (%) = 283 (10), 282 (14), 281 (73), 267 (13), 265 (24), 249 (15), 248 (75), 234 (29), 232 (14), 231 (100), 230 (79), 227 (10), 225 (37), 221 (10), 220 (33), 217 (11), 216 (75).
HRMS (EI): *m/z* calc. for [$C_{17}H_{15}NO$]: 249.3130; found: 248.1071 (M – H).
m.p. (°C): 116.2 – 118.8.

2-(Cyclohex-2-en-1-yl)-3-phenylacrylonitrile (4ae)

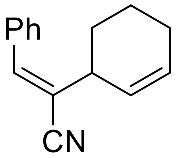

According to the **TP1**, a solution cinnamonitrile (**1a**, 0.20 M, 26 mg, 0.20 mmol, 1.0 equiv; *E/Z* > 99/1) in THF (total volume: 1 mL) and a solution of NaDA (0.26 M in DMEA, 0.26 mmol, 1.3 equiv) were prepared. The precooled solutions were mixed with an overall 10 $mL \cdot min^{-1}$ flow-rate in a T-mixer. The combined stream passed a 0.02 mL reactor tube (0.12 s, –78 °C) and was subsequently injected in a flask containing a stirred solution of 3-bromocyclohexene (**3e**, 48 mg, 0.30 mmol, 1.5 equiv) and CuCN·2LiCl (20 µL, 0.02 mmol, 0.1 equiv) in THF. The reaction was instantaneously quenched by the addition of *sat. aq.* NH_4Cl. The aqueous phase was extracted three times with EtOAc (3×10 mL) and the combined organic phases were dried over anhydrous $MgSO_4$ and filtrated. After removal of the solvent *in vacuo*, flash chromatographical purification (silica gel, isohexane:EtOAc = 9:1) afforded the title compound **4ae** as a colorless oil (46 mg, 0.19 mmol, 93% yield; *E/Z* = 90/10).

^{1}H-NMR (400 MHz, $CDCl_3$):

(*E*)-2-(Cyclohex-2-en-1-yl)-3-phenylacrylonitrile: δ / ppm = 7.44 – 7.36 (m, 3H), 7.31 – 7.27 (m, 2H), 7.24 (s, 1H), 5.97 (ddt, *J* = 10.0, 5.0, 2.6 Hz, 1H), 5.59 – 5.48 (m, 1H), 3.56 (ddp, *J* = 10.3, 5.4, 2.6 Hz, 1H), 2.19 – 1.98 (m, 2H), 1.96 – 1.86 (m, 2H), 1.82 – 1.73 (m, 1H), 1.63 – 1.51 (m, 1H).

(*Z*)-2-(Cyclohex-2-en-1-yl)-3-phenylacrylonitrile: δ / ppm = 7.76 – 7.72 (m, 2H), 7.44 – 7.33 (m, 2H), 6.96 (s, 1H), 6.03 – 6.00 (m, 1H), 5.62 (dq, *J* = 10.0, 2.5 Hz, 1H), 3.18 (d, *J* = 7.3 Hz, 1H), 2.20 (s, 1H), 2.19 – 1.98 (m, 2H), 1.96 – 1.86 (m, 2H), 1.82 (d, *J* = 2.2 Hz, 1H), 1.63 – 1.51 (m, 1H).

^{13}C-NMR (100 MHz, $CDCl_3$):

(*E*)-2-(Cyclohex-2-en-1-yl)-3-phenylacrylonitril:e δ / ppm = 143.9, 134.2, 131.4, 129.4, 129.1 (2C), 128.9 (2C), 126.5, 121.1, 119.7, 35.6, 28.5, 24.6, 21.3.

(*Z*)-2-(Cyclohex-2-en-1-yl)-3-phenylacrylonitril:e δ / ppm = 143.2, 134.2, 131.5, 130.0, 128.8 (2C), 128.4 (2C), 126.3, 121.1, 119.7, 41.7, 29.8, 25.0, 20.1.

IR (Diamond-ATR, neat): $\tilde{\nu}$ / cm^{-1} = 3023, 2929, 2926, 2880, 2857, 2839, 2835, 2210, 1616, 1490, 1456, 1446, 1436, 1431, 1302, 1132, 1075, 1048, 1029, 1000, 979, 927, 907, 889, 886, 873, 844, 778, 752, 724, 696, 676, 672, 668, 661, 655.

MS (EI, 70 eV): *m/z* (%) = 209 (28), 208 (85), 195 (12), 194 (74), 192 (31), 191 (18.22), 181 (34), 180 (14), 168 (11), 167 (85), 166 (100), 165 (19), 154 (19), 153 (24), 152 (20), 141 (26), 140 (19), 130 (19), 128 (14), 115 (27).

HRMS (EI): *m/z* calc. for [$C_{15}H_{15}N$]: 209.1204; found: 209.1198.

2-(Butylthio)-3-phenylacrylonitrile (4af)

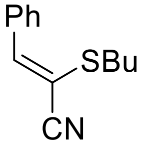

According to the **TP1**, a solution cinnamonitrile (**1a**, 0.20 M, 26 mg, 0.20 mmol, 1.0 equiv; *E/Z* > 99/1) in THF (total volume: 1 mL) and a solution of NaDA (0.24 M in DMEA, 0.24 mmol, 1.2 equiv) were prepared. The precooled solutions were mixed with an overall 10 mL·min^{-1} flow-rate in a T-mixer. The combined stream passed a 0.02 mL reactor tube (0.12 s, –78 °C) and was subsequently injected in a flask containing a stirred solution of dibutyl disulfide (**3f**, 54 mg, 0.30 mmol, 1.5 equiv) in THF. The reaction was instantaneously quenched by the addition of *sat. aq.* NH_4Cl. The aqueous phase was extracted three times with EtOAc (3×10 mL) and the combined organic phases were dried over anhydrous $MgSO_4$ and filtrated. After removal of the solvent *in vacuo*, flash chromatographical purification (silica gel, isohexane:EtOAc = 9:1) afforded the title compound **4af** as a pale yellow oil (46 mg, 0.19 mmol, 93% yield; *Z*/*E* = 54/46).

^{1}H-NMR (400 MHz, $CDCl_3$):

(*Z*)-2-(Butylthio)-3-phenylacrylonitrile: δ / ppm = 7.73 (dd, *J* = 6.5, 3.0 Hz, 1H), 7.65 – 7.60 (m, 1H), 7.45 – 7.38 (m, 3H), 7.32 (s, 1H), 2.97 – 2.89 (m, 2H), 1.66 (tt, *J* = 15.0, 7.9 Hz, 2H), 1.45 (dp, *J* = 14.4, 7.3 Hz, 2H), 0.93 (td, *J* = 7.3, 5.5 Hz, 3H).

(*E*)-2-(Butylthio)-3-phenylacrylonitrile: δ / ppm = 7.73 (dd, *J* = 6.5, 3.0 Hz, 1H), 7.65 – 7.60 (m, 1H), 7.45 – 7.38 (m, 3H), 7.25 (s, 1H), 3.06 – 3.01 (m, 2H), 1.66 (tt, *J* = 15.0, 7.9 Hz, 2H), 1.45 (dp, *J* = 14.4, 7.3 Hz, 2H), 0.93 (td, *J* = 7.3, 5.5 Hz, 3H).

^{13}C-NMR (100 MHz, $CDCl_3$):

(*Z*)-2-(Butylthio)-3-phenylacrylonitrile: δ / ppm = 146.1, 133.5, 129.9, 129.1 (2C), 128.8 (2C), 116.5, 109.5, 33.4, 31.7, 21.8, 13.7.

(*E*)-2-(Butylthio)-3-phenylacrylonitrile: δ / ppm = 142.7, 134.2, 130.7, 130.5 (2C), 128.7 (2C), 115.8, 105.5, 34.2, 32.0, 21.8, 13.7.

IR (Diamond-ATR, neat): $\tilde{\nu}$ / cm^{-1} = 2957, 2927, 2871, 2858, 2359, 2210, 1464, 1456, 1445, 1289, 1274, 919, 754, 689.

MS (EI, 70 eV): *m/z* (%) = 217 (43), 161 (24), 160 (13), 134 (100).

HRMS (EI): *m/z* calc. for [$C_{13}H_{15}NS$]: 217.0925; found: 217.0920.

(*E*)-2-(Hydroxydiphenylmethyl)-3-phenylacrylonitrile (4ag)

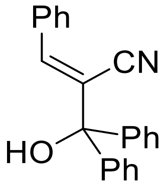

According to the **TP1**, a solution of cinnamonitrile (**1a**, 0.20 M, 26 mg, 0.20 mmol, 1.0 equiv; *E/Z* > 99/1) in THF (total volume: 1 mL) and a solution of NaDA (0.24 M in DMEA, 0.24 mmol, 1.2 equiv) were prepared. The precooled solutions were mixed with an overall 10 $mL \cdot min^{-1}$ flow-rate in a T-mixer. The combined stream passed a 0.02 mL reactor tube (0.12 s, –78 °C) and was subsequently injected in a flask containing a stirred solution of benzophenone (**3g**, 55 mg, 0.30 mmol, 1.5 equiv) in THF. The reaction was instantaneously quenched by the addition of *sat. aq.* NH_4Cl. The aqueous phase was extracted three times with EtOAc (3×10 mL) and the combined organic phases were dried over anhydrous $MgSO_4$ and filtrated. After removal of the solvent *in vacuo*, flash chromatographical purification (silica gel, isohexane:EtOAc = 9:1) afforded the title compound **4ag** as colorless crystals (51 mg, 0.16 mmol, 82% yield; *E/Z* > 99/1).

^{1}H-NMR (400 MHz, $CDCl_3$): δ / ppm = 7.78 – 7.75 (m, 2H), 7.48 – 7.36 (m, 13H), 7.12 (s, 1H), 2.91 (s, 1H).

^{13}C-NMR (100 MHz, $CDCl_3$): δ / ppm = 144.3, 143.2, 133.1, 130.7 (2C), 129.4 (2C), 129.0 (2C), 128.6 (4C), 128.6 (2C), 127.8 (4C), 118.5, 118.2, 81.3.

IR (Diamond-ATR, neat): $\tilde{\nu}$ / cm^{-1} = 3371, 2921, 2852, 2224, 1614, 1492, 1449, 1375, 1346, 1203, 1185, 1168, 1156, 1119, 1101, 1087, 1071, 1047, 1030, 1025, 1002, 950, 925, 911, 885, 770, 753, 733, 700, 688, 680, 660.

MS (EI, 70 eV): *m/z* (%) = 299 (10), 281 816), 227 (14), 226 (13), 225 (100), 209 (40), 208 (11), 207 (80), 206 (13), 191 (17), 183 (12), 151 (10), 105 (58), 78 (11), 77 (20).

HRMS (EI): *m/z* calc. for [$C_{22}H_{17}NO$]: 311.1310 found: 311.1305.

m.p. (°C): 152.1 – 158.0.

(*E*)-2-(2-Hydroxybicyclo[2.2.1]heptan-2-yl)-3-phenylacrylonitrile (4ah)

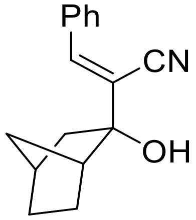

According to the **TP1**, a solution of cinnamonitrile (**1a**, 0.18 M, 23 mg, 0.18 mmol, 1.0 equiv; *E/Z* > 99/1) in THF (total volume: 1 mL) and a solution of NaDA (0.21 M in DMEA, 0.21 mmol, 1.2 equiv) were prepared. The precooled solutions were mixed with an overall 10 mL·min^{-1} flow-rate in a T-mixer. The combined stream passed a 0.02 mL reactor tube (0.12 s, –78 °C) and was subsequently injected in a flask containing a stirred solution of norcamphor (**3i**, 33 mg, 0.30 mmol, 1.7 equiv) in THF. The reaction was instantaneously quenched by the addition of *sat. aq.* NH_4Cl. The aqueous phase was extracted three times with EtOAc (3×10 mL) and the combined organic phases were dried over anhydrous $MgSO_4$ and filtrated. After removal of the solvent *in vacuo*, flash chromatographical purification (silica gel, isohexane:EtOAc = 9:1) afforded the title compound **4ah** as a colorless solid (39 mg, 0.16 mmol, 82% yield; *E/Z* > 99/1, *d.r.* > 99/1).

According to the **TP2**, a solution of cinnamonitrile (**1a**, 0.20 M, 26 mg, 0.20 mmol, 1.0 equiv; *E/Z* > 99/1) in THF (total volume: 1 mL) and a solution of NaTMP (0.26 M in hexane, 0.26 mmol, 1.3 equiv) were prepared. The precooled solutions were mixed with an overall 10 mL·min^{-1} flow-rate in a T-mixer. The combined stream passed a 0.02 mL reactor tube (0.12 s, –78 °C) and was subsequently injected in a flask containing a stirred solution of norcamphor (**3h**, 33 mg, 0.30 mmol, 1.5 equiv) in THF. The reaction was instantaneously quenched by the addition of *sat. aq.* NH_4Cl. The aqueous phase was extracted three times with EtOAc (3×10 mL) and the combined organic phases were dried over anhydrous $MgSO_4$ and filtrated. After removal of the solvent *in vacuo*, flash chromatographical purification (silica gel, isohexane:EtOAc = 9:1) afforded the title compound **4ah** as a colorless solid (34 mg, 0.14 mmol, 71% yield; *E*/*Z* > 99/1, *d.r.* > 99/1).

^{1}H-NMR (400 MHz, $CDCl_3$): δ / ppm = 7.80 – 7.73 (m, 2H), 7.47 – 7.37 (m, 3H), 7.25 (s, 1H), 2.58 (dd, *J* = 3.8, 1.5 Hz, 1H), 2.37 (t, *J* = 4.9 Hz, 1H), 2.31 – 2.26 (m 1H), 2.13 – 2.06 (m 1H), 1.93 (s, 1H), 1.72 – 1.61 (m, 2H), 1.57 – 1.45 (m, 2H), 1.45 – 1.42 (m, 1H), 1.41 – 1.40 (m, 1H).

^{13}C-NMR (100 MHz, $CDCl_3$): δ / ppm = 141.5, 133.5, 130.4, 129.2 (2C), 129.0 (2C), 120.0, 118.6, 80.5, 47.1, 45.6, 39.0, 37.5, 28.7, 22.4.

IR (Diamond-ATR, neat): $\tilde{\nu}$ / cm^{-1} = 3432, 2952, 2870, 2212, 1608, 1599, 1575, 1495, 1476, 1448, 1374, 1366, 1339, 1326, 1309, 1292, 1271, 1254, 1211, 1185, 1165, 1126, 1077, 1046, 1030, 1013, 969, 956, 927, 890, 871, 843, 806, 755, 734, 690, 666.

MS (EI, 70 eV): *m/z* (%) = 221 (31), 220 (15), 194 (15), 193 (100), 192 (87), 191 (34), 190 (25), 178 (66), 177 (10), 170 (11), 166 (16), 165 (72), 152 (13), 143 (10), 115 (14), 91 (19), 77 (14).

HRMS (EI): *m/z* calc. for [$C_{16}H_{17}NO$]: 239.1310 found: 239.1315.

m.p. (°C): 74.5 – 77.3.

(*E*)-2-Benzylidene-3-cyclopropyl-3-hydroxybutanenitrile (4ai)

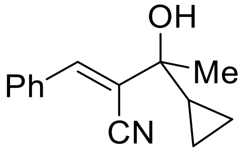

According to the **TP1**, a solution of cinnamonitrile (**1a**, 0.18 M, 23 mg, 0.18 mmol, 1.0 equiv; *E/Z* > 99/1) in THF (total volume: 1 mL) and a solution of NaDA (0.21 M in DMEA, 0.21 mmol, 1.2 equiv) were prepared. The precooled solutions were mixed with an overall 10 mL·min^{-1} flow-rate in a T-mixer. The combined stream passed a 0.02 mL reactor tube (0.12 s, –78 °C) and was subsequently injected in a flask containing a stirred solution of cyclopropylmethylketone (**3i**, 25 mg, 0.30 mmol, 1.7 equiv) in THF. The reaction was instantaneously quenched by the addition of *sat. aq.* NH_4Cl. The aqueous phase was extracted three times with EtOAc (3×10 mL) and the combined organic phases were dried over anhydrous $MgSO_4$ and filtrated. After removal of the solvent *in vacuo*, flash chromatographical purification (silica gel, isohexane:EtOAc = 9:1) afforded the title compound **4ai** as a yellow oil (30 mg, 0.14 mmol, 78% yield; *E/Z* > 99/1).

According to the **TP2**, a solution of cinnamonitrile (**1a**, 0.20 M, 26 mg, 0.20 mmol, 1.0 equiv; *E/Z* > 99/1) in THF (total volume: 1 mL) and a solution of NaTMP (0.26 M in hexane, 0.26 mmol, 1.3 equiv) were prepared. The precooled solutions were mixed with an overall 10 mL·min^{-1} flow-rate in a T-mixer. The combined stream passed a 0.02 mL reactor tube (0.12 s, –78 °C) and was subsequently injected in a flask containing a stirred solution of methylcyclopropyl ketone (**3i**, 25 mg, 0.30 mmol, 1.5 equiv) in THF. The reaction was instantaneously quenched by the addition of *sat. aq.* NH_4Cl. The aqueous phase was extracted three times with EtOAc (3×10 mL) and the combined organic phases were dried over anhydrous $MgSO_4$ and filtrated. After removal of the solvent *in vacuo*, flash chromatographical purification (silica gel, isohexane:EtOAc = 9:1) afforded the title compound **4ai** as a yellow oil (29 mg, 0.14 mmol, 68% yield; *E/Z* > 99/1).

^{1}H-NMR (400 MHz, $CDCl_3$): δ / ppm = 7.78 (dd, *J* = 7.5, 1.7 Hz, 2H), 7.46 – 7.38 (m, 4H), 1.65 (s, 1H), 1.52 (s, 3H), 1.36 – 1.27 (m, 1H), 0.66 – 0.48 (m, 4H).

^{13}C-NMR (100 MHz, $CDCl_3$): δ / ppm = 141.1, 133.6, 130.3, 129.2 (2C), 128.9 (2C), 119.1, 118.3, 72.9, 26.9, 21.1, 2.2, 1.2.

IR (Diamond-ATR, neat): $\tilde{\nu}$ / cm^{-1} = 3451, 2974, 2924, 2853, 2212, 1494, 1447, 1374, 1219, 1194, 1155, 1079, 1049, 1037, 1023, 1000, 961, 931, 903, 874, 757, 690.

MS (EI, 70 eV): *m/z* (%) = 195 (21), 194 (50), 193 (13), 184 (41), 180 (70), 168 (20), 167 (21), 166 (97), 165 (19), 156 (12), 155 (33), 154 (100), 153 (50), 152 (35), 141 (17), 140 (20), 139 (16), 128 (15), 127 (26), 126 (15), 115 (22), 91 (16), 77 (11).

HRMS (EI): *m/z* calc. for [$C_{14}H_{15}NO$]: 213.1154; found: 213.1148

(*E*)-2-Benzylidene-3-hydroxy-3-methylheptanenitrile (4aj)

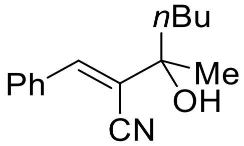

According to the **TP1**, a solution of cinnamonitrile (**1a**, 0.20 M, 26 mg, 0.20 mmol, 1.0 equiv; *E/Z* > 99/1) in THF (total volume: 1 mL) and a solution of NaDA (0.24 M in DMEA, 0.24 mmol, 1.2 equiv) were prepared. The precooled solutions were mixed with an overall 10 mL·min^{-1} flow-rate in a T-mixer. The combined stream passed a 0.02 mL reactor tube (0.12 s, –78 °C) and was subsequently injected in a flask containing a stirred solution of hexan-2-one (**3j**, 30 mg, 0.30 mmol, 1.5 equiv) in THF. The reaction was instantaneously quenched by the addition of *sat. aq.* NH_4Cl. The aqueous phase was extracted three times with EtOAc (3×10 mL) and the combined organic phases were dried over anhydrous $MgSO_4$ and filtrated. After removal of the solvent *in vacuo*, flash chromatographical purification (silica gel, isohexane:EtOAc = 9:1) afforded the title compound **4aj** as a colorless oil (40 mg, 0.17 mmol, 87% yield; *E*/*Z* > 99/1).

According to the **TP2**, a solution of cinnamonitrile (**1a**, 0.20 M, 26 mg, 0.20 mmol, 1.0 equiv; *E/Z* > 99/1) in THF (total volume: 1 mL) and a solution of NaTMP (0.26 M in hexane, 0.26 mmol, 1.3 equiv) were prepared. The precooled solutions were mixed with an overall 10 mL·min^{-1} flow-rate in a T-mixer. The combined stream passed a 0.02 mL reactor tube (0.12 s, –78 °C) and was subsequently injected in a flask containing a stirred solution of hexan-2-one (**3j**, 30 mg, 0.30 mmol, 1.5 equiv) in THF. The reaction was instantaneously quenched by the addition of *sat. aq.* NH_4Cl. The aqueous phase was extracted three times with EtOAc (3×10 mL) and the combined organic phases were dried over anhydrous $MgSO_4$ and filtrated. After removal of the solvent *in vacuo*, flash chromatographical purification (silica gel, isohexane:EtOAc = 9:1) afforded the title compound **4aj** as a white solid (38 mg, 0.17 mmol, 83% yield; *E/Z* > 99/1).

^{1}H-NMR (400 MHz, $CDCl_3$): δ / ppm = 7.77 (dd, *J* = 7.6, 1.7 Hz, 2H), 7.47 – 7.39 (m, 3H), 7.37 (s, 1H), 1.94 – 1.82 (m, 1H), 1.79 (dd, *J* = 11.0, 5.1 Hz, 1H), 1.74 (s, 1H), 1.56 (s, 3H), 1.41 – 1.29 (m, 4H), 0.92 (t, *J* = 6.9 Hz, 3H).

^{13}C-NMR (100 MHz, $CDCl_3$): δ / ppm = 141.5, 133.5, 130.3, 129.1 (2C), 129.0 (2C), 118.7,118.1, 74.8, 41.2, 28.4, 258, 23.0, 14.1.

IR (Diamond-ATR, neat): $\tilde{\nu}$ / cm^{-1} = 3466, 2957, 2931, 2862, 2210, 1722, 1619, 1495, 1466, 1448, 1376, 1342, 1289, 1260, 1232, 1165, 1128, 1096, 1074, 1045, 1035, 945, 929, 886, 775, 746, 731, 690, 666, 656.

MS (EI, 70 eV): *m/z* (%) = 172 (39), 169 (14), 168 (47), 167 (21), 155 (11), 154 (100), 153 (11), 130 (17).

HRMS (EI): *m/z* calc. for [$C_{15}H_{19}NO$]: 229.1467; found: 229.1457.

2-(Hydroxy(2-methoxyphenyl)methyl)-3-(4-methoxyphenyl)acrylonitrile (7ak)

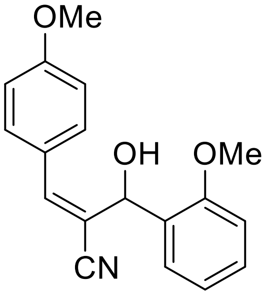

According to the **TP1**, a solution of 3-(4-methoxyphenyl)acrylonitrile (**5a**, 0.20 M, 32 mg, 0.20 mmol, 1.0 equiv; *E*/*Z* = 76/24) in THF (total volume: 1 mL) and a solution of NaDA (0.24 M in DMEA, 0.24 mmol, 1.2 equiv) were prepared. The precooled solutions were mixed with an overall 10 mL·min^{-1} flow-rate in a T-mixer. The combined stream passed a 0.02 mL reactor tube (0.12 s, –78 °C) and was subsequently injected in a flask containing a stirred solution of 2-methoxybenzaldehyde (**3k**, 41 mg, 0.30 mmol, 1.5 equiv) in THF. The reaction was instantaneously quenched by the addition of *sat. aq.* NH_4Cl. The aqueous phase was extracted three times with EtOAc (3×10 mL) and the combined organic phases were dried over anhydrous $MgSO_4$ and filtrated. After removal of the solvent *in vacuo*, flash chromatographical purification (silica gel, isohexane:EtOAc = 9:1) afforded the title compound **7ak** as colorless crystals (57 mg, 0.19 mmol, 97% yield; *Z/E* = 89/11).

^{1}H-NMR (400 MHz, $CDCl_3$):

(*Z*)-2-(Hydroxy(2-methoxyphenyl)methyl)-3-(4-methoxyphenyl)acrylonitrile: δ / ppm = 7.79 – 7.72 (m, 2H), 7.45 (dd, *J* = 7.5, 1.6 Hz, 1H), 7.38 – 7.31 (m, 2H), 7.16 (s, 1H), 6.94 – 6.90 (m, 3H), 5.69 – 5.62 (m, 1H), 3.87 (s, 3H), 3.84 (s, 3H), 3.13 – 3.08 (m, 1H).

(*E*)-2-(Hydroxy(2-methoxyphenyl)methyl)-3-(4-methoxyphenyl)acrylonitrile: δ / ppm = 7.41 (d, *J* = 7.6 Hz, 1H), 7.38 – 7.31 (m, 3H), 7.05 (d, *J* = 0.9 Hz, 1H), 7.01 (dd, *J* = 2.7, 0.9 Hz, 1H), 6.97- 6.89 (m, 3H), 5.93 (d, *J* = 5.4 Hz, 1H), 3.85 (s, 3H), 3.84 (s, 3H), 3.39 – 3.27 (m, 1H).

^{13}C-NMR (100 MHz, $CDCl_3$):

(*Z*)-2-(Hydroxy(2-methoxyphenyl)methyl)-3-(4-methoxyphenyl)acrylonitrile: δ / ppm = 161.4, 156.8, 142.5, 131.1 (2C), 129.9, 128.2, 127.9, 126.2, 121.3, 118.1, 114.4 (3C), 111.1, 72.2, 55.7, 55.5.

(*E*)-2-(Hydroxy(2-methoxyphenyl)methyl)-3-(4-methoxyphenyl)acrylonitrile: δ / ppm = 161.1, 157.1, 145.8, 131.7 (2C), 130.1, 128.1, 127.6, 126.4, 121.4, 115.4, 114.3, 111.1, 111.1 (2C), 67.2, 55.7, 55.5.

IR (Diamond-ATR, neat): $\tilde{\nu}$ / cm^{-1} = 3431, 3005, 2959, 2932, 2928, 2838, 2359, 2210, 1602, 1570, 1512, 1490, 1463, 1439, 1424, 1404, 1399, 1388, 1306, 1288, 1256, 1179, 1162, 1136, 1110, 1046, 1028, 832, 791, 756.

MS (EI, 70 eV): *m/z* (%) = 277 (18), 262 (13), 253 (10).

HRMS (EI): *m/z* calc. for [$C_{18}H_{17}NO_3$]: 295.1208 found: 295.1203.

m.p. (°C): 101.8 –106.1.

3-(3,4-Dimethylphenyl)-2-(hydroxy(2-methoxyphenyl)methyl)acrylonitrile (7bk)

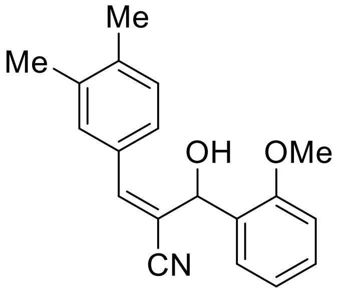

According to the **TP1**, a solution of 3-(3,4-dimethylphenyl)acrylonitrile (**5b**, 0.20 M, 31 mg, 0.20 mmol, 1.0 equiv; *E*/*Z* = 79/21) in THF (total volume: 1 mL) and a solution of NaDA (0.22 M in DMEA, 0.22 mmol, 1.10 equiv) were prepared. The precooled solutions were mixed with an overall 10 mL·min^{-1} flow-rate in a T-mixer. The combined stream passed a 0.02 mL reactor tube (0.12 s, –78 °C) and was subsequently injected in a flask containing a stirred solution of 2-methoxybenzaldehyde (**3k**, 41 mg, 0.30 mmol, 1.5 equiv) in THF. The reaction was instantaneously quenched by the addition of *sat. aq.* NH_4Cl. The aqueous phase was extracted three times with EtOAc (3×10 mL) and the combined organic phases were dried over anhydrous $MgSO_4$ and filtrated. After removal of the solvent *in vacuo*, flash chromatographical purification (silica gel, isohexane:EtOAc = 9:1) afforded the title compound **7bk** as colorless crystals (49 mg, 0.17 mmol, 84% yield; *Z/E* = 90/10).

^{1}H-NMR (400 MHz, $CDCl_3$):

(*Z*)-3-(3,4-Dimethylphenyl)-2-(hydroxy(2-methoxyphenyl)methyl)acrylonitrile: δ / ppm = 7.44 (dd, *J* = 7.5, 1.7 Hz, 1H), 7.37 – 7.31 (m, 2H), 7.16 – 7.13 (m, 3H), 7.01 (td, *J* = 7.6, 1.1 Hz, 1H), 6.94 (dd, *J* = 8.3, 1.1 Hz, 1H), 5.96 (s, 1H), 3.83 (s, 3H), 3.30 (s, 1H), 2.29 (s, 3H), 2.25 (s, 3H).

(*E*)-3-(3,4-Dimethylphenyl)-2-(hydroxy(2-methoxyphenyl)methyl)acrylonitrile: δ / ppm = 7.57 – 7.50 (m, 1H), 7.36 (d, *J* = 1.7 Hz, 1H), 7.32 (d, *J* = 1.7 Hz, 1H), 7.27 (d, *J* = 7.6 Hz, 1H), 7.19 – 7.16 (m, 2H), 7.14 – 7.11 (m, 1H), 6.92 – 6.87 (m, 1H), 5.66 (s, 1H) 4.69 (s, 1H), 3.86 (d, *J* = 4.7 Hz, 3H), 2.29 (s, 3H), 2.25 (s, 3H).

^{13}C-NMR (100 MHz, $CDCl_3$):

(*Z*)-3-(3,4-Dimethylphenyl)-2-(hydroxy(2-methoxyphenyl)methyl)acrylonitrile: δ / ppm = 156.9, 146.1, 139.1, 137.1, 131.3, 130.9, 130.1, 130.0, 128.2, 127.6, 127.2, 121.3, 119.3, 116.8, 111.0, 66.8, 55.5, 19.9 (2C).

(*E*)-3-(3,4-Dimethylphenyl)-2-(hydroxy(2-methoxyphenyl)methyl)acrylonitrile: δ / ppm = 156.7, 143.0, 139.7, 137.2, 131.1, 130.5, 130.2, 129.9, 129.1, 128.9, 128.1, 127.9, 126.7, 120.8, 110.3, 72.1, 55.6, 19.9, 19.8.

IR (Diamond-ATR, neat): $\tilde{\nu}$ / cm^{-1} = 3456, 2973, 2919, 2846, 2208, 1602, 1590, 1565, 1489, 1469, 1458, 1440, 1411, 1382, 1353, 1342, 1296, 1282, 1244, 1195, 1180, 1161, 1127, 1116, 1047, 1030, 910, 877, 853, 821, 789, 751, 724, 710, 674.

MS (EI, 70 eV): *m/z* (%) = 293 (30), 278 (21), 170 (14), 159 (10), 158 (59), 157 (12), 137 (71), 136 (13), 135 (100), 121 (13), 119 (10), 107 (35), 91 (12), 77 (31).
HRMS (EI): *m/z* calc. for [$C_{19}H_{19}NO_2$]: 293.1416; found: 293.1400.
m.p. (°C): 122.0 – 123.7.

(*Z*)-2-(Cyclohexyl(hydroxy)methyl)-3-(2,3-dihydrobenzo[*b*][1,4]dioxin-6-yl)acrylonitrile (7cl)

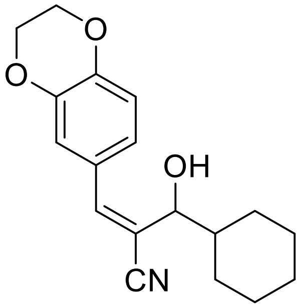

According to the **TP1**, a solution of 3-(2,3-dihydrobenzo[*b*][1,4]dioxin-6-yl)acrylonitrile (**5c**, 0.19 M, 36 mg, 0.19 mmol, 1.0 equiv; *E*/*Z* = 83/17) in THF (total volume: 1 mL) and a solution of NaDA (0.22 M in DMEA, 0.22 mmol, 1.2 equiv) were prepared. The precooled solutions were mixed with an overall 10 mL·min^{-1} flow-rate in a T-mixer. The combined stream passed a 0.02 mL reactor tube (0.12 s, –78 °C) and was subsequently injected in a flask containing a stirred solution of cyclohexanecarbaldehyde (**3l**, 34 mg, 0.30 mmol, 1.6 equiv) in THF. The reaction was instantaneously quenched by the addition of *sat. aq.* NH_4Cl. The aqueous phase was extracted three times with EtOAc (3×10 mL) and the combined organic phases were dried over anhydrous $MgSO_4$ and filtrated. After removal of the solvent *in vacuo*, flash chromatographical purification (silica gel, isohexane:EtOAc = 4:1) afforded the title compound **7cl** as a colorless oil (42 mg, 0.15 mmol, 74% yield; *Z/E* > 99/1).

^{1}H-NMR (400 MHz, $CDCl_3$): δ / ppm = 7.23 (s, 1H), 6.94 – 6.90 (m, 1H), 6.89 – 6.85 (m, 2H), 4.31 – 4.26 (m, 4H), 2.10 (d, *J* = 12.8 Hz, 1H), 1.99 (s, 1H), 1.82 – 1.75 (m, 1H), 1.72 – 1.64 (m, 3H), 1.34 – 1.21 (m, 3H), 1.18 – 1.10 (m, 1H), 1.09 – 0.98 (m, 1H), 0.87 (qd, *J* = 13.0, 12.5, 3.9 Hz, 1H).
^{13}C-NMR (100 MHz, $CDCl_3$): δ / ppm = 146.1, 145.1, 143.6, 126.8, 123.2, 118.9, 118.4, 117.7, 117.0, 71.8, 64.6, 64.3, 42.7, 29.2, 29.0, 26.2, 25.8, 25.6.
IR (Diamond-ATR, neat): $\tilde{\nu}$ / cm^{-1} = 3443, 2924, 2851, 2212, 1606, 1578, 1504, 1450, 1433, 1312, 1285, 1256, 1243, 1212, 1187, 1160, 1127, 1065, 1050, 1018, 964, 919, 887, 850, 816, 784, 730, 677.
MS (EI, 70 eV): *m/z* (%) = 217 (18), 216 (100), 198 (12), 188 (16), 55 (13), 42 (12).
HRMS (EI): *m/z* calc. for [$C_{18}H_{21}O_3N$]: 299.1521 found: 299.1515.

(*E*)-3-(4-(*Tert*-butyl)phenyl)-2-(1-hydroxycyclohexyl)acrylonitrile (7dm)

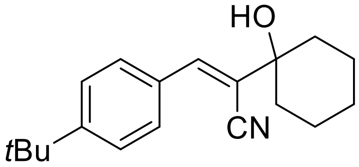

According to the **TP1**, a solution of 3-(4-(*tert*-butyl)phenyl)-2-(cyclohexyl(hydroxy) methyl)acrylonitrile (**5d**, 0.18 M, 33 mg, 0.18 mmol, 1.0 equiv; *E*/*Z* = 79/21) in THF (total volume: 1 mL) and a solution of NaDA (0.20 M in DMEA, 0.20 mmol, 1.1 equiv) were prepared. The precooled solutions were mixed with an overall 10 mL·min^{-1} flow-rate in a T-mixer. The combined stream passed a 0.02 mL reactor tube (0.12 s, –78 °C) and was subsequently injected in a flask containing a stirred solution of cyclohexanone (**3m**, 29 mg, 0.30 mmol, 1.7 equiv) in THF. The reaction was instantaneously quenched by the addition of *sat. aq.* NH_4Cl. The aqueous phase was extracted three times with EtOAc (3×10 mL) and the combined organic phases were dried over anhydrous $MgSO_4$ and filtrated. After removal of the solvent *in vacuo*, flash chromatographical purification (silica gel, isohexane:EtOAc = 9:1) afforded the title compound **7dm** as a colorless solid (34 mg, 0.5 mmol, 67% yield; *E*/*Z* > 99/1).

^{1}H-NMR (400 MHz, $CDCl_3$): δ / ppm = 7.74 – 7.69 (m, 2H), 7.46 – 7.41 (m, 2H), 7.38 (s, 1H), 1.99 – 1.87 (m, 2H), 1.76 – 1.62 (m, 8H), 1.33 (s, 9H).

^{13}C-NMR (100 MHz, $CDCl_3$): δ / ppm = 153.8, 141.0, 130.8, 129.0 (2C), 125.9 (2C), 119.4, 118.5, 73.4, 36.7 (2C), 35.0, 31.3 (3C), 25.0, 21.7 (2C).

IR (Diamond-ATR, neat): $\tilde{\nu}$ / cm^{-1} = 3451, 2961, 2930, 2854, 2211, 1708, 1607, 1507, 1460, 1446, 1430, 1412, 1384, 1367, 1352, 1317, 1290, 1266, 1260, 1221, 1200, 1172, 1131, 1107, 1077, 1056, 1039, 1015, 991, 955, 935, 926, 916, 901, 844, 828, 802, 664.

MS (EI, 70 eV): *m/z* (%) = 283 (32), 268 (43), 240 (18), 226 (45), 184 (21), 171 (17), 170 (100), 154 (11), 147 (29), 115 (13), 57 (68), 55 (13), 43 (13), 41 (31).

HRMS (EI): *m/z* calc. for [$C_{19}H_{25}NO$]: 283.1936; found: 283.1931.

m.p. (°C): 89.6 – 93.2.

(*E*)-3-(4-(*Tert*-butyl)phenyl)-2-(cyclohex-2-en-1-yl)acrylonitrile (7de)

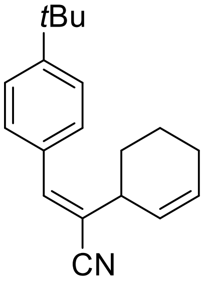

According to the **TP1**, a solution of 3-(4-(*tert*-butyl)phenyl)acrylonitrile (**5d**, 0.18 M, 33 mg, 0.18 mmol 1.0 equiv; *E*/*Z* = 79/21) in THF (total volume: 1 mL) and a solution of NaDA (0.20 M in DMEA, 0.20 mmol, 1.1 equiv) were prepared. The precooled solutions were mixed with an overall 10 mL·min^{-1} flow-rate in a T-mixer. The combined stream passed a 0.02 mL reactor tube (0.12 s, –78 °C) and was subsequently injected in a flask containing a stirred solution of 3-bromocyclohexene (**3e**, 46 mg, 0.30 mmol, 1.7 equiv) and CuCN·2LiCl (20 µL, 0.02 mmol, 0.1 equiv) in THF. The reaction was instantaneously quenched by the addition of *sat. aq.* NH_4Cl. The aqueous phase was extracted three times with EtOAc (3×10 mL) and the combined organic phases were dried over anhydrous $MgSO_4$ and filtrated. After removal of the solvent *in vacuo*, flash chromatographical purification (silica gel, isohexane:EtOAc = 49:1) afforded the title compound **7de** as a yellow oil (27 mg, 0.10 mmol, 57% yield; *E/Z* > 99/1).

^{1}H-NMR (400 MHz, $CDCl_3$): δ / ppm = 7.45 – 7.41 (m, 2H), 7.27 – 7.23 (m, 2H), 7.20 (s, 1H), 5.96 (ddt, *J* = 9.8, 4.9, 2.6 Hz, 1H), 5.54 (dd, *J* = 10.0, 2.6 Hz, 1H), 3.67 – 3.54 (m, 1H), 2.20 – 2.04 (m, 2H), 1.98 – 1.87 (m, 2H), 1.83 – 1.76 (m, 1H), 1.66 – 1.58 (m, 1H), 1.33 (s, 9H).
^{13}C-NMR (100 MHz, $CDCl_3$): δ / ppm = 152.9, 143.8, 131.4, 131.3, 129.1 (2C), 126.7, 125.9 (2C), 120.2, 119.9, 35.6, 35.0, 31.3 (3C), 28.5, 24.6, 21.4.
IR (Diamond-ATR, neat): $\tilde{\nu}$ / cm^{-1} = 3023, 2960, 2931, 2863, 2836, 2210, 1607, 1506, 1475, 1461, 1447, 1432, 1412, 1395, 1363, 1301, 1289, 1270, 1201, 1132, 1108, 1016, 980, 932, 924, 898, 874, 858, 846, 824, 723, 664.
MS (EI, 70 eV): *m/z* (%) = 250 (51), 209 (38), 208 (100), 194 (40), 192 (24), 191 (12), 182 (15), 181 (20), 180 (52) 167 (25), 166 (82), 165 (22), 154 (17), 153 (12), 152 (14), 141 (12), 115 (28), 104, (13), 91 (15), 79 (12).
HRMS (EI): *m/z* calc. for [$C_{19}H_{23}N$]: 265.1830; found: 265.1826.

(*E*)-2-(Cyclohex-2-en-1-yl)-3-(4-(trifluoromethyl)phenyl)acrylonitrile (7ee)

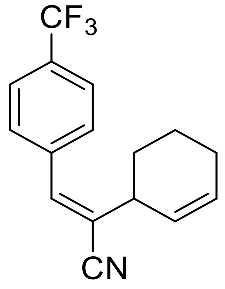

According to the **TP1**, a solution of 3-(4-(trifluoromethyl)phenyl)acrylonitrile (**5e**, 0.20 M, 39 mg, 0.20 mmol, 1.0 equiv; *E*/*Z* = 78/22) in THF (total volume: 1 mL) and a solution of NaDA (0.24 M in DMEA, 0.24 mmol, 1.2 equiv) were prepared. The precooled solutions were mixed with an overall 10 mL·min^{-1} flow-rate in a T-mixer. The combined stream passed a 0.02 mL reactor tube (0.12 s, –78 °C) and was subsequently injected in a flask containing a stirred solution of 3-bromocyclohexene (**3e**, 48 mg, 0.30 mmol, 1.5 equiv) and CuCN·2LiCl (20 µL, 1.0 M, 0.02 mmol, 0.1 equiv) in THF. The reaction was instantaneously quenched by the addition of *sat. aq.* NH_4Cl. The aqueous phase was extracted three times with EtOAc (3×10 mL) and the combined organic phases were dried over anhydrous $MgSO_4$ and filtrated. After removal of the solvent *in vacuo*, flash chromatographical purification (silica gel, isohexane:EtOAc = 19:1) afforded the title compound **7ee** as white crystals (33 mg, 0.13 mmol, 66% yield; *E/Z* > 99/1).

^{1}H-NMR (400 MHz, $CDCl_3$): δ / ppm = 7.67 (d, *J* = 8.2 Hz, 2H), 7.39 (d, *J* = 8.6 Hz, 2H), 7.26 (s, 1H), 5.99 (ddt, *J* = 9.9, 5.0, 2.6 Hz, 1H), 5.53 – 5.47 (m, 1H), 3.50 – 3.44 (m, 1H), 2.18 – 1.99 (m, 2H), 1.95 – 1.86 (m, 2H), 1.81 – 1.73 (m, 1H), 1.62 – 1.50 (m, 1H).

^{13}C-NMR (100 MHz, $CDCl_3$): δ / ppm = 142.1, 137.5, 132.0, 131.2 (q, *J* = 32.8 Hz), 129.3 (2C), 125.9 (q, *J* = 3.8 Hz, 2C), 125.9, 123.9 (q, *J* =272.5 Hz), 123.5, 119.0, 35.8, 28.4, 24.5, 21.2.

IR (Diamond-ATR, neat): $\tilde{\nu}$ / cm^{-1} = 2956, 2922, 2856, 2210, 1614, 1456, 1446, 1408, 1344, 1322, 1278, 1255, 1170, 1127, 1108, 1080, 1066, 1014, 976, 960, 934, 900, 873, 848, 830, 763, 728, 667, 656.

MS (EI, 70 eV): *m/z* (%) = 277 (18), 276 (51), 262 (22), 248 (56), 235 (97), 234 (51), 221 (26), 209 (23), 208 (61), 204 (21), 202 (20), 183 (20), 180 (51), 173 (29), 166 (100), 165 (21), 159 (53), 154 (46), 153 (22), 152 (20), 145 (29), 140 (15), 80 (15), 79 (52), 78 (27), 77 (33).

HRMS (EI): *m/z* calc. for [$C_{16}H_{14}NF_3$]: 277.1078; found: 277.1082.

m.p. (°C): 74.9 – 78.2.

(*Z*)-4-Hydroxy-3-methoxy-4-(4-(trifluoromethyl)phenyl)but-2-enenitrile (7fn)

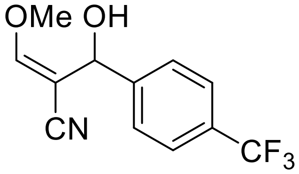

According to the **TP1**, a solution 3-methoxyacrylonitrile (**5f**, 0.20 M, 17 mg, 0.20 mmol, 1.0 equiv; *E*/*Z* = 83:17) in THF (total volume: 1 mL) and a solution of NaDA (0.21 M in DMEA, 0.21 mmol, 1.05 equiv) were prepared. The precooled solutions were mixed with an overall 10 mL·min^{-1} flow-rate in a T-mixer. The combined stream passed a 0.02 mL reactor tube (0.12 s, –78 °C) and was subsequently injected in a flask containing a stirred solution of (4-trifluoromethyl)benzaldehyde (**3n**, 52 mg, 0.30 mmol, 1.5 equiv) in THF. The reaction was instantaneously quenched by the addition of *sat. aq.* NH_4Cl. The aqueous phase was extracted three times with EtOAc (3×10 mL) and the combined organic phases were dried over anhydrous $MgSO_4$ and filtrated. After removal of the solvent *in vacuo*, flash chromatographical purification (silica gel, isohexane:EtOAc = 3:1) afforded the title compound **7fn** as a pale orange solid (36 mg, 0.14 mmol, 93% yield; *Z/E* > 99/1).

^{1}H-NMR (400 MHz, $CDCl_3$): δ / ppm = 7.63 (d, *J* = 8.2 Hz, 2H), 7.56 (d, *J* = 8.1 Hz, 2H), 6.88 (d, *J* = 0.8 Hz, 1H), 5.80 (s, 1H), 3.92 (s, 3H).

^{13}C-NMR (100 MHz, $CDCl_3$): δ / ppm = 160.3, 144.7 (d, *J* = 1.4 Hz), 130.5 (q, *J* = 32.4 Hz, 2C), 126.2, 125.8 (q, *J* = 3.8 Hz, 2C), 124.2 (q, *J* = 272.2 Hz), 116.9, 97.5, 66.5, 62.7.

IR (Diamond-ATR, neat): $\tilde{\nu}$ / cm^{-1} = 3391, 2952, 2922, 2852, 2224, 1712, 1632, 1457, 1446, 1411, 1319, 1259, 1206, 1161, 1148, 1118, 1110, 1064, 1052, 1015, 973, 961, 917, 869, 853, 788, 771, 724, 700.

MS (EI, 70 eV): *m/z* (%) = 257 (12), 256 (65), 240 (13), 238 (22), 236 (16), 228 (26), 227 (13), 226 (25), 225 (34), 224 (94), 223 (17), 223 (20), 222 (42), 214 (73), 210 (11), 208 (68), 207 (34), 206 (88), 203 (12), 200 (17), 198 (30), 197 (100), 196 (99), 195 (66), 194 (25), 188 (52), 187 (10), 186 (15), 185 (11).

HRMS (EI): *m/z* calc. for [$C_{12}H_{10}F_3NO_2$]: 257.0664; found 256.0579 (M – H).

m.p. (°C): 100.1 – 102.5.

(*Z*)-2-(Hydroxy(*p*-tolyl)methyl)-3-methoxyacrylonitrile (7fd)

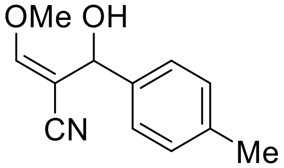

According to the **TP1**, a solution of 3-methoxyacrylonitrile (**5f**, 0.20 M, 17 mg, 0.20 mmol, 1.0 equiv; *E*/*Z* = 83:17) in THF (total volume: 1 mL) and a solution of NaDA (0.24 M in DMEA, 0.24 mmol, 1.2 equiv) were prepared. The precooled solutions were mixed with an overall 10 mL·min^{-1} flow-rate in a T-mixer. The combined stream passed a 0.02 mL reactor tube (0.12 s, –78 °C) and was subsequently injected in a flask containing a stirred solution of 4-methylbenzaldehyde (**3d**, 36 mg, 0.30 mmol, 1.5 equiv) in THF. The reaction was instantaneously quenched by the addition of *sat. aq.* NH_4Cl. The aqueous phase was extracted three times with EtOAc (3×10 mL) and the combined organic phases were dried over anhydrous $MgSO_4$ and filtrated. After removal of the solvent *in vacuo*, flash chromatographical purification (silica gel, isohexane:EtOAc = 7:3) afforded the title compound **7fd** as white crystals (40 mg, 0.20 mmol, 98% yield; *Z/E* > 99/1).

^{1}H-NMR (400 MHz, $CDCl_3$): δ / ppm = 7.34 (d, *J* = 8.1 Hz, 2H), 7.18 (d, *J* = 7.8 Hz, 2H), 6.82 (d, *J* = 0.8 Hz, 1H), 5.69 (s, 1H), 3.89 (s, 3H), 2.35 (s, 3H), 2.20 (s, 1H).
^{13}C-NMR (100 MHz, $CDCl_3$): δ / ppm = 159.7, 138.3, 138.1, 129.5 (2C), 125.8 (2C), 117.4, 98.2, 67.1, 62.4, 21.3.
IR (Diamond-ATR, neat): $\tilde{\nu}$ / cm^{-1} = 3401, 2939, 2922, 2904, 2850, 2224, 1636, 1612, 1512, 1450, 1407, 1319, 1294, 1257, 1238, 1195, 1175, 1148, 1111, 1063, 1022, 978, 972, 951, 916, 859, 840, 792, 767, 680.
MS (EI, 70 eV): *m/z* (%) = 203 (15), 171 (10), 156 (43), 121 (39), 120 (21), 115 (17), 111 (14), 97 (19), 92 (18), 91 (46), 85 (15), 83 (19), 77 (15), 71 (17), 70 (11), 65 (16), 61 (13), 57 (47), 56 (14), 55 (28), 45 (17), 44 (28), 43 (100), 42 (13), 41 (27).
HRMS (EI): *m/z* calc. for [$C_{12}H_{13}NO_2$]: 203.0946; found: 203.0944.
m.p. (°C): 129.7 – 132.3.

(*Z*)-2-((2,6-Dichlorophenyl)(hydroxy)methyl)-3-ethoxyacrylonitrile (7gc)

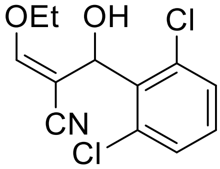

According to the **TP1**, a solution of 3-ethoxyacrylonitrile (**5g**, 0.20 M, 19 mg, 0.20 mmol, 1.0 equiv; *E*/*Z* = 68:32) in THF (total volume: 1 mL) and a solution of NaDA (0.22 M in DMEA, 0.22 mmol, 1.1 equiv) were prepared. The precooled solutions were mixed with an overall 10 mL·min^{-1} flow-rate in a T-mixer. The combined stream passed a 0.02 mL reactor tube (0.12 s, –78 °C) and was subsequently injected in a flask containing a stirred solution of 2,6-dichlorobenzaldehyde (**3c**, 52 mg, 0.30 mmol, 1.5 equiv) in THF. The reaction was instantaneously quenched by the addition of *sat. aq.* NH_4Cl. The aqueous phase was extracted three times with EtOAc (3×10 mL) and the combined organic phases were dried over anhydrous $MgSO_4$ and filtrated. After removal of the solvent *in vacuo*, flash chromatographical purification (silica gel, isohexane:EtOAc = 7:3) afforded the title compound **7gc** as a colorless solid (52 mg, 0.19 mmol, 95% yield; *Z/E* > 99/1).

^{1}H-NMR (400 MHz, $CDCl_3$): δ / ppm = 7.31 (d, *J* = 8.0 Hz, 2H), 7.20 – 7.15 (m, 1H), 6.87 (d, *J* = 1.8 Hz, 1H), 6.24 (d, *J* = 1.5 Hz, 1H), 4.03 (dq, *J* = 10.1, 7.1 Hz, 1H), 3.94 (dq, *J* = 10.1, 7.1 Hz, 1H), 3.41 (s, 1H), 1.18 (t, *J* = 7.1 Hz, 3H).

^{13}C-NMR (100 MHz, $CDCl_3$): δ / ppm = 159.0, 135.6, 135.1, 129.7 (2C), 129.2 (2C), 117.8, 95.8, 71.6, 66.2, 15.1.

IR (Diamond-ATR, neat): $\tilde{\nu}$ / cm^{-1} = 3376, 2956, 2921, 2852, 2223, 1627, 1581, 1563, 1539, 1453, 1437, 1399, 1376, 1302, 1222, 1196, 1179, 1149, 1111, 1088, 1076, 1061, 1023, 967, 910, 874, 856, 819, 771, 761, 724, 712, 699, 688.

MS (EI, 70 eV): *m/z* (%) = 190 (15), 173 (11), 61 (19), 57 (11), 45 (15), 44 (32), 43 (100), 41 (13).

HRMS (EI): *m/z* calc. for [$C_{12}H_{11}Cl_2NO_2$]: 271.0167; found: 271.0172.

m.p. (°C): 85.0 – 87.3.

(*Z*)-4-Cyclohexyl-3-ethoxy-4-hydroxybut-2-enenitrile (7gl)

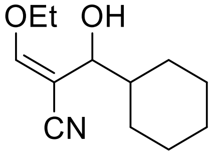

According to the **TP1**, a solution 3-ethoxyacrylonitrile (**5g**, 0.20 M, 19 mg, 0.20 mmol, 1.0 equiv; *E*/*Z* = 68:32) in THF (total volume: 1 mL) and a solution of NaDA (0.21 M in DMEA, 0.21 mmol, 1.1 equiv) were prepared. The precooled solutions were mixed with an overall 10 mL·min^{-1} flow-rate in a T-mixer. The combined stream passed a 0.02 mL reactor tube (0.12 s, –78 °C) and was subsequently injected in a flask containing a stirred solution of cyclohexanecarbaldehyde (**3l**, 34 mg, 0.30 mmol, 1.5 equiv) in THF. The reaction was instantaneously quenched by the addition of *sat. aq.* NH_4Cl. The aqueous phase was extracted three times with EtOAc (3×10 mL) and the combined organic phases were dried over anhydrous $MgSO_4$ and filtrated. After removal of the solvent *in vacuo*, flash chromatographical purification (silica gel, isohexane:EtOAc = 4:1) afforded the title compound **7gl** as a colorless liquid (38 mg, 0.18 mmol, 91% yield; *Z/E* > 99/1).

^{1}H-NMR (400 MHz, $CDCl_3$): δ / ppm = 6.90 (s, 1H), 4.26 (d, *J* = 8.5 Hz, 1H), 4.04 (q, *J* = 7.1 Hz, 2H), 2.01 (d, *J* = 12.9 Hz, 1H), 1.93 (s, 1H), 1.73 (dd, *J* = 29.6, 13.0 Hz, 2H), 1.64 (t, *J* = 15.2 Hz, 2H), 1.58 – 1.52 (m, 1H), 1.31 (t, *J* = 7.1 Hz, 3H), 1.27 – 1.21 (m, 2H), 1.19 – 1.12 (m, 1H), 0.98 (dq, *J* = 37.4, 12.3 Hz, 2H).

^{13}C-NMR (100 MHz, $CDCl_3$): δ / ppm = 159.3, 118.2, 96.4, 71.2, 70.0, 42.6, 29.1, 28.7, 26.4, 25.9, 25.7, 15.3.

IR (Diamond-ATR, neat): $\tilde{\nu}$ / cm^{-1} = 3442, 2983, 2923, 2850, 2211, 1633, 1476, 1449, 1391, 1336, 1326, 1302, 1213, 1190, 1171, 1144, 1107, 1086, 1083, 1014, 964, 920, 891, 883, 848, 809, 794, 678.

MS (EI, 70 eV): *m/z* (%) = 162 (10), 134 (29), 126 (39), 117 (12), 107 (11), 106 (16), 98 (100), 81 (48), 79 (29), 77 (11), 70 (11), 67 (20).

HRMS (EI): *m/z* calc. for [$C_{12}H_{19}NO_2$]: 209.1416; found 209.1413.

2-(2-Phenyl-1-(phenylthio)vinyl)adamantan-2-ol (7ho)

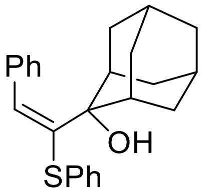

According to the **TP1**, a solution phenyl(styryl)sulfane (**5h**, 0.20 M, 43 mg, 0.20 mmol, 1.0 equiv; *E*/*Z* = 71/29) in THF (total volume: 1 mL) and a solution of NaDA (0.24 M in DMEA, 0.24 mmol, 1.2 equiv) were prepared. The precooled solutions were mixed with an overall 10 mL·min^{-1} flow-rate in a T-mixer. The combined stream passed a 0.02 mL reactor tube (0.12 s, –78 °C) and was subsequently injected in a flask containing a stirred solution of 2-adamantanone (**3o**, 45 mg, 0.30 mmol, 1.5 equiv) in THF. The reaction was instantaneously quenched by the addition of *sat. aq.* NH_4Cl. The aqueous phase was extracted three times with EtOAc (3×10 mL) and the combined organic phases were dried over anhydrous $MgSO_4$ and filtrated. After removal of the solvent *in vacuo*, flash chromatographical purification (silica gel, isohexane:EtOAc = 19:1) afforded the title compound **7ho** as colorless crystals (69 mg, 0.19 mmol, 95% yield; *E*/*Z* = 77/23).

^{1}H-NMR (400 MHz, $CDCl_3$):

(*E*/*Z*)-2-(2-Phenyl-1-(phenylthio)vinyl)adamantan-2-ol: δ / ppm = 7.65 – 7.60 (m, 2H), 7.57 (d, J = 6.9 Hz, 1H), 7.40 – 6.99 (m, 7H), 6.81 (s, 1H), 2.57 (s, 1H), 2.45 – 2.37 (m, 1H), 2.34 (t, J = 2.9 Hz, 1H), 2.28 (d, J = 13.1 Hz, 1H), 2.23 – 2.14 (m, 1H), 2.00 (d, J = 12.5 Hz, 1H), 1.95 – 1.85 (m, 1H), 1.85 – 1.67 (m, 2H), 1.65 (d, J = 3.3 Hz, 2H), 1.60 – 1.53 (m, 2H), 1.53 – 1.44 (m, 2H).

^{13}C-NMR (100 MHz, $CDCl_3$):

(*E*)-2-(2-Phenyl-1-(phenylthio)vinyl)adamantan-2-ol: δ / ppm = 139.4, 136.4, 135.9, 135.7, 129.4 (2C), 128.8 (2C), 128.7 (2C), 127.9 (2C), 127.7, 125.7, 79.0, 39.4, 37.9, 35.7, 34.8 (2C), 33.3 (2C), 27.6, 27.0.

(*Z*)-2-(2-Phenyl-1-(phenylthio)vinyl)adamantan-2-ol: δ / ppm = 144.7, 139.3, 137.4, 135.8, 132.0 (2C), 129.5 (2C), 128.6 (2C), 128.0 (2C), 127.3, 127.1, 79.0, 39.1, 37.5, 35.5, 35.0 (2C), 32.9 (2C), 26.8, 26.6.

IR (Diamond-ATR, neat): $\tilde{\nu}$ / cm^{-1} = 3524, 2952, 2911, 2898, 2891, 2851, 1492, 1487, 1480, 1469, 1445, 1439, 1380, 1364, 1344, 1327, 1317, 1308, 1303, 1292, 1284, 1267, 1178, 1172, 1162, 1128, 1118, 1112, 1103, 1097, 1083, 1068, 1057, 1041, 1025, 1007, 999, 983, 965, 951, 930, 917, 907, 890, 880, 848, 832, 806, 791, 776, 763, 749, 741, 695, 689, 681, 667, 657.

MS (EI, 70 eV): *m/z* (%) = 362 (32), 253 (13), 213 (17), 212 (100), 211 (29), 179 (12), 178 (11), 167 (15), 151 (25), 121 (11), 91 (21), 79 (12), 77 (11), 41 (10).

HRMS (EI): *m/z* calc. for [$C_{24}H_{26}OS$]: 362.1704; found 362.1697.

m.p. (°C): 101.9 – 106.1.

1,1,3-Triphenyl-2-(phenylthio)prop-2-en-1-ol (7hg)

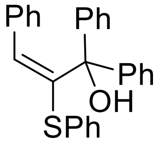

According to the **TP1**, a solution phenyl(styryl)sulfane (**5h**, 0.20 M, 43 mg, 0.20 mmol, 1.0 equiv; *E*/*Z* = 71/29) in THF (total volume: 1 mL) and a solution of NaDA (0.24 M in DMEA, 0.24 mmol, 1.2 equiv) were prepared. The precooled solutions were mixed with an overall 10 mL·min^{-1} flow-rate in a T-mixer. The combined stream passed a 0.02 mL reactor tube (0.12 s, –78 °C) and was subsequently injected in a flask containing a stirred solution of benzophenone (**3g**, 45 mg, 0.30 mmol, 1.5 equiv) in THF. The reaction was instantaneously quenched by the addition of *sat. aq.* NH_4Cl. The aqueous phase was extracted three times with EtOAc (3×10 mL) and the combined organic phases were dried over anhydrous $MgSO_4$ and filtrated. After removal of the solvent *in vacuo*, flash chromatographical purification (silica gel, isohexane:EtOAc = 19:1) afforded the title compound **7hg** as a colorless liquid (67 mg, 0.17 mmol, 85% yield; *E*/*Z* = 68/32).

^{1}H-NMR (400 MHz, $CDCl_3$):

(*E*/*Z*)-1,1,3-Triphenyl-2-(phenylthio)prop-2-en-1-ol: δ / ppm = 7.56 – 7.43 (m, 6H), 7.40 – 7.18 (m, 9H), 7.18 – 7.06 (m, 1H), 7.06 – 6.95 (m, 2H), 6.81 (ddt, *J* = 6.0, 4.8, 3.8 Hz, 1H), 6.65 (d, *J* = 7.7 Hz, 1H), 3.60 (d, *J* = 225.7 Hz, 1H).

^{13}C-NMR (100 MHz, $CDCl_3$):

(*E*/*Z*)-1,1,3-Triphenyl-2-(phenylthio)prop-2-en-1-ol: δ / ppm = 146.4, 145.2, 144.5, 140.1, 137.3, 135.4, 133.1, 131.3, 129.6, 129.5, 128.9, 128.3, 128.3, 128.3, 128.2, 128.2, 128.1, 128.1, 127.9, 127.9, 127.8, 127.7, 127.0, 125.8, 84.6, 82.9.

IR (Diamond-ATR, neat): $\tilde{\nu}$ / cm^{-1} = 3480, 3057, 3023, 2959, 2923, 2849, 1657, 1616, 1597, 1578, 1559, 1490, 1477, 1445, 1439, 1317, 1309, 1276, 1176, 1155, 1091, 1074, 1048, 1031, 1024, 1015, 999, 987, 941, 919, 909, 883, 853, 809, 788, 754, 736, 696, 661.

MS (EI, 70 eV): *m/z* (%) = 213 (18), 212 (100), 211 (19), 183 (42), 178 (14), 105 (64), 77 (36).

HRMS (EI): *m/z* calc. for [$C_{27}H_{22}OS$]: 394.1391; found 394.1389.

2-(Hydroxyiodomethyl)-3,7-dimethylocta-2,6-dienenitrile (10ap)

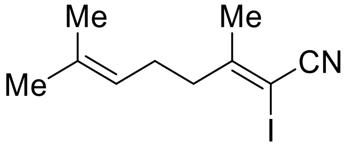

According to the **TP1**, a solution geranyl nitrile (**8a**, 0.18 M, 27 mg, 0.18 mmol, 1.0 equiv; *E/Z* = 50/50) in THF (total volume: 1 mL) and a solution of NaDA (0.22 M in DMEA, 0.22 mmol, 1.2 equiv) were prepared. The precooled solutions were mixed with an overall 10 mL·min^{-1} flow-rate in a T-mixer. The combined stream passed a 0.02 mL reactor tube (0.12 s, –78 °C) and was subsequently injected in a flask containing a stirred solution of iodine (**3p**, 76 mg, 0.30 mmol, 1.5 equiv) in THF. The reaction was instantaneously quenched by the addition of *sat. aq.* NH_4Cl. The aqueous phase was extracted three times with EtOAc (3×10 mL) and the combined organic phases were dried over anhydrous $MgSO_4$ and filtrated. After removal of the solvent *in vacuo*, flash chromatographical purification (silica gel, isohexane:EtOAc = 19:1) afforded the title compound **10ap** as a brown oil (37 mg, 0.13 mmol, 75% yield; *Z/E* = 68/32).

^{1}H-NMR (400 MHz, $CDCl_3$):

(*E*)-2-(Hydroxyiodomethyl)-3,7-dimethylocta-2,6-dienenitrile: δ / ppm = 5.13 – 5.04 (m, 1H), 2.38 (dd, *J* = 8.7, 7.0 Hz, 2H), 2.24 – 2.13 (m, 2H), 2.20 (s, 2H), 2.03 (s, 1H), 1.70 (s, 3H), 1.63 (s, 3H).

(*Z*)-2-(Hydroxyiodomethyl)-3,7-dimethylocta-2,6-dienenitrile: δ / ppm = 5.13 – 5.04 (m, 1H), 2.60 (t, *J* = 7.5 Hz, 2H), 2.24 – 2.13 (m, 2H), 2.20 (s, 2H), 2.03 (s, 1H), 1.70 (s, 3H), 1.61 (s, 3H).

^{13}C-NMR (100 MHz, $CDCl_3$):

(*E*)-2-(Hydroxyiodomethyl)-3,7-dimethylocta-2,6-dienenitrile: δ / ppm = 166.4, 134.0, 121.9, 117.8, 53.8, 42.0, 26.6, 25.8, 23.0, 17.9.

(*Z*)-2-(Hydroxyiodomethyl)-3,7-dimethylocta-2,6-dienenitrile: δ / ppm = 166.9, 134.2, 121.7, 117.6, 54.6, 38.7, 29.9, 27.3, 25.6, 17.8.

IR (Diamond-ATR, neat): $\tilde{\nu}$ / cm^{-1} = 3452, 3058, 3026, 2966, 2920, 2855, 2208, 1657, 1597, 1491, 1446, 1376, 1320, 1278, 1157, 1111, 1075, 1024, 1002, 921, 898, 825, 750, 699.

MS (EI, 70 eV): *m/z* (%) = 260 (11), 207 (40), 148 (43), 128 (10), 127 (100), 69 (99), 67 (10).

HRMS (EI): *m/z* calc. for [$C_{10}H_{14}IN$]: 275.0171; found 275.0176.

2-(Cyclohexyl(hydroxy)methyl)-3,7-dimethylocta-2,6-dienenitrile (10al)

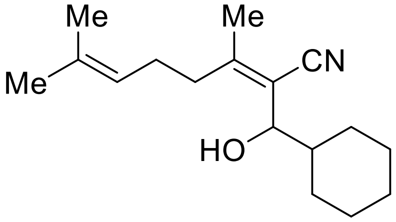

According to the **TP1**, a solution geranyl nitrile (**8a**, 0.18 M, 27 mg, 0.18 mmol, 1.0 equiv; *E/Z* = 50/50) in THF (total volume: 1 mL) and a solution of NaDA (0.22 M in DMEA, 0.22 mmol, 1.2 equiv) were prepared. The precooled solutions were mixed with an overall 10 mL·min^{-1} flow-rate in a T-mixer. The combined stream passed a 0.02 mL reactor tube (0.12 s, –78 °C) and was subsequently injected in a flask containing a stirred solution of cyclohexanecarbaldehyde (**3l**, 34 mg, 0.30 mmol, 1.7 equiv) in THF. The reaction was instantaneously quenched by the addition of *sat. aq.* NH_4Cl. The aqueous phase was extracted three times with EtOAc (3×10 mL) and the combined organic phases were dried over anhydrous $MgSO_4$ and filtrated. After removal of the solvent *in vacuo*, flash chromatographical purification (silica gel, isohexane:EtOAc = 19:1) afforded the title compound **10al** as an orange oil (28 mg, 0.11 mmol, 60% yield; *Z/E* = 64/36).

^{1}H-NMR (400 MHz, $CDCl_3$):
(*E*)-2-(Cyclohexyl(hydroxy)methyl)-3,7-dimethylocta-2,6-dienenitrile: δ / ppm = 5.15 – 5.03 (m, 1H), 4.13 – 4.05 (m, 1H), 2.43 – 2.33 (m, 1H), 2.33 – 2.04 (m, 4H), 2.11 (s, 3H), 1.82 – 1.75 (m, 1H), 1.74 – 1.64 (m, 6H), 1.62 – 1.53 (m, 2H), 1,61 (s, 3H), 1.32 – 1.23 (m, 2H), 1.15 (dd, *J* = 25.3, 6.9 Hz, 1H), 1.07 – 0.93 (m, 1H), 0.90 – 0.77 (m, 1H).
(*Z*)-2-(Cyclohexyl(hydroxy)methyl)-3,7-dimethylocta-2,6-dienenitrile: δ / ppm = 5.15 – 5.03 (m, 1H), 4.13 – 4.05 (m, 1H), 2.61 – 2.51 (m, 1H), 2.33 – 2.04 (m, 4H), 1.87 (s, 3H), 1.82 – 1.75 (m, 1H), 1.74 – 1.64 (m, 6H), 1.62 – 1.53 (m, 2H), 1,61 (s, 3H), 1.32 – 1.23 (m, 2H), 1.15 (dd, *J* = 25.3, 6.9 Hz, 1H), 1.07 – 0.93 (m, 1H), 0.90 – 0.77 (m, 1H).
^{13}C-NMR (100 MHz, $CDCl_3$):
(*E*)-2-(Cyclohexyl(hydroxy)methyl)-3,7-dimethylocta-2,6-dienenitrile: δ / ppm = 157.8, 133.9, 122.6, 117.6, 114.6, 72.7, 42.6, 34.2, 29.4, 29.4, 26.4 (2C), 26.0, 25.8, 25.8, 22.0, 17.9.
(*Z*)-2-(Cyclohexyl(hydroxy)methyl)-3,7-dimethylocta-2,6-dienenitrile: δ / ppm = 157.5, 133.5, 122.5, 117.3, 114.3, 73.1, 42.9, 39.0, 29.3, 28.9, 26.4 (2C), 26.0, 25.8, 23.0, 18.8, 17.8.
IR (Diamond-ATR, neat): $\tilde{\nu}$ / cm^{-1} = 3430, 2924, 2853, 2211, 1658, 1600, 1506, 1492, 1447, 1408, 1377, 1352, 1320, 1305, 1278, 1260, 1225, 1185, 1159, 1110, 1100, 1083, 1026, 1014, 960, 892, 833, 821, 812, 751, 700.
MS (EI, 70 eV): *m/z* (%) = 69 (19), 61 (14), 45 (13), 43 (100), 41 (11).
HRMS (EI): *m/z* calc. for [$C_{17}H_{27}NO$]: 261.2093 found 261.2080.

2-(1-Hydroxy-1,2,3,4-tetrahydronaphthalen-1-yl)-3,7-dimethylocta-2,6-dienenitrile (10aq)

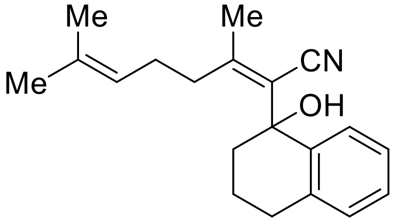

According to the **TP1**, a solution geranyl nitrile (**8a**, 0.18 M, 27 mg, 0.18 mmol, 1.0 equiv; *E/Z* = 50/50) in THF (total volume: 1 mL) and a solution of NaDA (0.22 M in DMEA, 0.22 mmol, 1.2 equiv) were prepared. The precooled solutions were mixed with an overall 10 mL·min^{-1} flow-rate in a T-mixer. The combined stream passed a 0.02 mL reactor tube (0.12 s, –78 °C) and was subsequently injected in a flask containing a stirred solution of α-tetralone (**3q**, 44 mg, 0.30 mmol, 1.7 equiv) in THF. The reaction was instantaneously quenched by the addition of *sat. aq.* NH_4Cl. The aqueous phase was extracted three times with EtOAc (3×10 mL) and the combined organic phases were dried over anhydrous $MgSO_4$ and filtrated. After removal of the solvent *in vacuo*, flash chromatographical purification (silica gel, isohexane:EtOAc = 19:1) afforded the title compound **10aq** as an orange oil (53 mg, 0.18 mmol, 98% yield; *Z/E* = 53/47).

^{1}H-NMR (400 MHz, $CDCl_3$):

(*E*)-2-(1-Hydroxy-1,2,3,4-tetrahydronaphthalen-1-yl)-3,7-dimethylocta-2,6-dienenitrile: δ / ppm = 7.33 – 7.27 (m, 1H), 7.24 – 7.17 (m, 2H), 7.14 – 7.09 (m, 1H), 4.75 – 4.71 (m, 1H), 2.90 – 2.71 (m, 2H), 2.57 – 2.41 (m, 1H), 2.26 – 2.16 (m, 3H), 2.14 (s, 3H), 2.06 – 1.95 (m, 3H), 1.95 – 1.82 (m, 2H), 1.61 (s, 3H), 1.47 (s, 3H).

(*Z*)-2-(1-Hydroxy-1,2,3,4-tetrahydronaphthalen-1-yl)-3,7-dimethylocta-2,6-dienenitrile δ / ppm = 7.33 – 7.27 (m, 1H), 7.24 – 7.17 (m, 2H), 7.14 – 7.09 (m, 1H), 5.18 – 5.11 (m, 1H), 2.90 – 2.71 (m, 2H), 2.57 – 2.41 (m, 1H), 2.26 – 2.16 (m, 3H), 2.06 – 1.95 (m, 3H), 1.95 – 1.82 (m, 2H), 1.73 (s, 3H), 1.62 (s, 3H), 1.41 (s, 3H).

^{13}C-NMR (100 MHz, $CDCl_3$):

(*E*)-2-(1-Hydroxy-1,2,3,4-tetrahydronaphthalen-1-yl)-3,7-dimethylocta-2,6-dienenitrile: δ / ppm = 157.1, 140.5, 137.1, 132.9, 129.5, 128.4, 127.7, 127.2, 122.9, 120.4, 118.5, 73.3, 38.1, 34.7, 29.7, 25.9, 25.8, 23.6, 19.3, 17.7.

(*Z*)-2-(1-Hydroxy-1,2,3,4-tetrahydronaphthalen-1-yl)-3,7-dimethylocta-2,6-dienenitrile: δ / ppm = 157.0, 140.1, 137.2, 133.2, 129.5, 128.3, 127.6, 127.2, 122.6, 120.4, 118.1, 72.9, 39.9, 37.3, 29.7, 26.6, 25.6, 19.8, 19.2, 17.8.

IR (Diamond-ATR, neat): $\tilde{\nu}$ / cm^{-1} = 3443, 3020, 2928, 2864, 2209, 1609, 1488, 1449, 1440, 1376, 1327, 1277, 1195, 1182, 1158, 1112, 1090, 1076, 1038, 1020, 978, 955, 943, 931, 906, 876, 848, 824, 782, 758, 732.

MS (EI, 70 eV): *m/z* (%) = 277 (11), 227 (11), 226 (60), 208 (14), 194 (10), 147 (29), 129 (11), 118 (11), 115 (10), 91 (28), 69 (100), 41 (58).

HRMS (EI): *m/z* calc. for [$C_{20}H_{25}NO$]: 295.1936; found 295.1932.

2-((4-Chlorophenyl)(cyclopropyl)(hydroxy)methyl)-3-methylhept-2-enenitrile (10br)

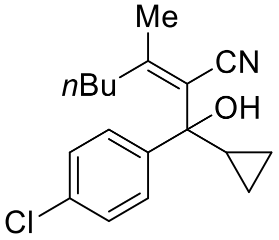

According to the **TP1**, a solution 3-methylhept-2-enenitrile (**8b**, 0.20 M, 25 mg, 0.20 mmol, 1.0 equiv; *E*/*Z* = 65/35) in THF (total volume: 1 mL) and a solution of NaDA (0.24 M in DMEA, 0.24 mmol, 1.2 equiv) were prepared. The precooled solutions were mixed with an overall 10 mL·min^{-1} flow-rate in a T-mixer. The combined stream passed a 0.02 mL reactor tube (0.12 s, –78 °C) and was subsequently injected in a flask containing a stirred solution of (4-chlorophenyl)(cyclopropyl)methanone (**3r**, 45 mg, 0.30 mmol, 1.5 equiv) in THF. The reaction was instantaneously quenched by the addition of *sat. aq.* NH_4Cl. The aqueous phase was extracted three times with EtOAc (3×10 mL) and the combined organic phases were dried over anhydrous $MgSO_4$ and filtrated. After removal of the solvent *in vacuo*, flash chromatographical purification (silica gel, isohexane:EtOAc = 19:1) afforded the title compound **10br** as a colorless oil (67 mg, 0.17 mmol, 85% yield; *Z/E* = 55/45).

(*E*/*Z*)-2-((4-Chlorophenyl)(cyclopropyl)(hydroxy)methyl)-3-methylhept-2-enenitrile:

^{1}H-NMR (400 MHz, $CDCl_3$):

δ / ppm = 7.31 (qd, *J* = 8.7, 2.0 Hz, 4H), 2.54 – 2.33 (m, 1H), 2.12 (s, 2H), 2.06 – 1.98 (m, 1H), 1.94 (d, *J* = 6.4 Hz, 1H), 1.60 (s, 1H), 1.56 – 1.42 (m, 2H), 1.38 (dt, *J* = 14.9, 7.3 Hz, 1H), 1.21 – 1.08 (m, 1H), 0.95 (q, *J* = 8.4, 7.2 Hz, 3H), 0.67 (dt, *J* = 15.5, 7.8 Hz, 2H), 0.57 – 0.47 (m, 1H), 0.42 (d, *J* = 5.2 Hz, 2H).

^{13}C-NMR (100 MHz, $CDCl_3$):

(*Z*)-2-((4-Chlorophenyl)(cyclopropyl)(hydroxy)methyl)-3-methylhept-2-enenitrile: δ / ppm = 164.2, 144.0, 133.1, 128.2 (2C), 127.3 (2C), 118.6, 118.2, 74.6, 35.2, 29.2, 24.2, 22.9, 22.6, 13.78, 2.3, 2.3.

(*E*)-2-((4-Chlorophenyl)(cyclopropyl)(hydroxy)methyl)-3-methylhept-2-enenitrile: δ / ppm = 164.3, 143.3, 133.2, 128.4 (2C), 127.3 (2C), 118.5, 118.2, 74.8, 39.7, 30.2, 24.0, 23.6, 20.5, 14.1, 2.3, 2.2.

IR (Diamond-ATR, neat): $\tilde{\nu}$ / cm^{-1} = 3432, 3009, 2956, 2928, 2871, 2861, 2358, 2338, 2208, 1604, 1489, 1456, 1401, 1186, 1163, 1092, 1014, 986, 869, 832, 827, 824, 807.

MS (EI, 70 eV): *m/z* (%) = 277 (12), 275 (36), 246 (12), 220 (14), 218 (43), 206 (11), 204 (11), 184 (12), 181 (25), 150 (64), 141 (33), 139 (100), 125 (20).

HRMS (EI): *m/z* calc. for [$C_{18}H_{21}ClNO$]: 302.1312; found: 302.1306 [M – H].

2-(1-Hydroxy-1,2,3,4-tetrahydronaphthalen-1-yl)-3-methylhex-2-enenitrile (10cq)

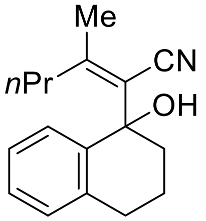

According to the **TP1**, a solution (*E*)-3-methylhex-2-enenitrile (**8c**, 0.20 M, 22 mg, 0.20 mmol, 1.0 equiv; *E*/*Z* > 99/1) in THF (total volume: 1 mL) and a solution of NaDA (0.24 M in DMEA, 0.24 mmol, 1.2 equiv) were prepared. The precooled solutions were mixed with an overall 10 mL·min^{-1} flow-rate in a T-mixer. The combined stream passed a 0.02 mL reactor tube (0.12 s, –78 °C) and was subsequently injected in a flask containing a stirred solution of α-tetralone (**3q**, 44 mg, 0.30 mmol, 1.5 equiv) in THF. The reaction was instantaneously quenched by the addition of *sat. aq.* NH_4Cl. The aqueous phase was extracted three times with EtOAc (3×10 mL) and the combined organic phases were dried over anhydrous $MgSO_4$ and filtrated. After removal of the solvent *in vacuo*, flash chromatographical purification (silica gel, isohexane:EtOAc = 19:1) afforded the title compound **10cq** as a colorless oil (34 mg, 0.17 mmol, 67% yield; *Z/E* = 58/42).

(*Z*)-2-(1-Hydroxy-1,2,3,4-tetrahydronaphthalen-1-yl)-3-methylhex-2-enenitrile

^{1}H-NMR (400 MHz, $CDCl_3$): δ / ppm = 7.32 (dd, *J* = 7.2, 2.0, 1H), 7.20 (ddd, *J* = 7.1, 4.3, 1.9, 2H), 7.14 – 7.10 (m, 1H), 2.91 – 2.71 (m, 2H), 2.28 – 2.17 (m, 2H), 2.11 (s, 3H), 2.01 – 1.70 (m, 5H), 1.36 – 1.23 (m, 1H), 1.13 – 0.97 (m, 1H), 0.58 (t, *J* = 7.3, 3H).

^{13}C-NMR (100 MHz, $CDCl_3$): δ / ppm = 157.5, 140.6, 137.0, 129.5, 128.4, 127.8, 127.2, 120.2, 118.4, 73.0, 38.3, 36.7, 29.8, 23.4, 20.5, 19.3, 14.3.

(*E*)-2-(1-Hydroxy-1,2,3,4-tetrahydronaphthalen-1-yl)-3-methylhex-2-enenitrile

^{1}H-NMR (400 MHz, $CDCl_3$): δ / ppm = 7.33 – 7.29 (m, 1H), 7.23 – 7.18 (m, 2H), 7.13 (dt, *J*=6.0, 3.4, 1H), 2.92 – 2.72 (m, 2H), 2.44 (td, *J*=7.3, 1.9, 2H), 2.30 – 2.15 (m, 2H), 2.05 – 1.81 (m, 3H), 1.58 – 1.48 (m, 2H), 1.41 (s, 3H), 0.98 (t, *J*=7.4, 3H).

^{13}C-NMR (100 MHz, $CDCl_3$): δ / ppm = 157.5, 140.1, 137.2, 129.6, 128.3, 127.6, 127.3, 120.3, 118.2, 72.9, 41.8, 37.4, 29.7, 21.4, 19.6, 19.2, 13.7.

IR (Diamond-ATR, neat): $\tilde{\nu}$ / cm^{-1} = 3447, 2957, 2932, 2871, 2209, 1706, 1610, 1489, 1450, 1441, 1379, 1363, 1329, 1277, 1260, 1223, 1182, 1162, 1086, 1039, 1020, 979, 945, 905, 827, 783, 758, 732.

MS (EI, 70 eV): *m/z* (%) = 227 (11), 148 (11), 147 (100), 129 (21), 91 (17).

HRMS (EI): *m/z* calc. for [$C_{17}H_{21}NO$]: 255.1623; found: 254.1538 [M – H].

(*Z*)-2-(Hydroxy(2-methoxyphenyl)methyl)-4-methoxy-3-methylbut-2-enenitrile (10dk)

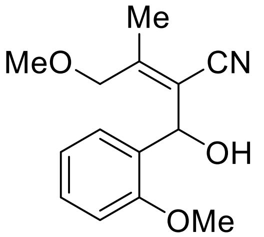

According to the **TP1**, a solution of 4-methoxy-3-methylbut-2-enenitrile (**8d**, 0.20 M, 35 mg, 0.20 mmol, 1.0 equiv; *E*/*Z* = 80/20) in THF (total volume: 1 mL) and a solution of NaDA (0.24 M in DMEA, 0.24 mmol, 1.2 equiv) were prepared. The precooled solutions were mixed with an overall 10 mL·min^{-1} flow-rate in a T-mixer. The combined stream passed a 0.02 mL reactor tube (0.12 s, –78 °C) and was subsequently injected in a flask containing a stirred solution of *m*-anisaldehyde (**3k**, 41 mg, 0.30 mmol, 1.5 equiv) in THF. The reaction was instantaneously quenched by the addition of *sat. aq.* NH_4Cl. The aqueous phase was extracted three times with EtOAc (3×10 mL) and the combined organic phases were dried over anhydrous $MgSO_4$ and filtrated. After removal of the solvent *in vacuo*, flash chromatographical purification (silica gel, isohexane:EtOAc = 1:1) afforded the title compound **10dk** as a white solid (29 mg, 0.12 mmol, 58% yield; *Z/E* > 99/1).

^{1}H-NMR (400 MHz, $CDCl_3$): δ / ppm = 7.54 – 7.47 (m, 1H), 7.31 (ddd, *J*=8.2, 7.5, 1.7, 1H), 7.02 (td, *J*=7.5, 1.1, 1H), 6.89 (dd, *J*=8.3, 1.1, 1H), 5.86 (s, 1H), 4.30 (d, *J*=13.2, 1H), 4.05 (d, *J*=13.2, 1H), 3.85 (s, 3H), 3.34 (s, 3H), 2.14 (s, 3H).

^{13}C-NMR (100 MHz, $CDCl_3$): δ / ppm = 156.3, 153.4, 129.6, 128.4, 127.2, 121.2, 116.8, 116.7, 110.6, 71.4, 65.9, 58.8, 55.4, 20.7.

IR (Diamond-ATR, neat): $\tilde{\nu}$ / cm^{-1} = 3416, 3353, 2938, 2843, 2217, 1600, 1590, 1488, 1463, 1449, 1438, 1377, 1290, 1266, 1245, 1190, 1166, 1133, 1114, 1095, 1079, 1042, 1030, 958, 942, 834, 790, 780, 756, 721, 661.

MS (EI, 70 eV): *m/z* (%) = 215 (12), 214 (26), 201 (13), 200 (100), 198 (14), 184 (29), 183 (28), 173 (25), 172 (12), 157 (19), 145 (12), 137 (13), 135 (35), 107 (29), 77 (11).

HRMS (EI): *m/z* calc. for [$C_{14}H_{17}NO_3$]: 247.1208; found: 229.1097 [M – H_2O].

m.p. (°C): 77.3 – 78.2.

2-(Cyclohex-2-en-1-yl)nona-2,4-dienenitrileenenitrile (10ee)

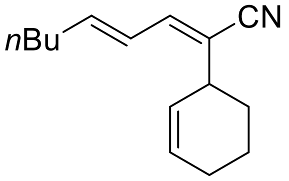

According to the **TP1**, a solution of (*2E,4E*)-nona-2,4-dienenitrile (**8e**, 0.20 M, 27 mg, 0.20 mmol, 1.0 equiv; 2*E*/2*Z* = 69/31) in THF (total volume: 1 mL) and a solution of NaDA (0.24 M in DMEA, 0.24 mmol, 1.2 equiv) were prepared. The precooled solutions were mixed with an overall 10 mL·min^{-1} flow-rate in a T-mixer. The combined stream passed a 0.02 mL reactor tube (0.12 s, –78 °C) and was subsequently injected in a flask containing a stirred solution of cyclohexene bromide (**3e**, 30 mg, 0.30 mmol, 1.5 equiv) and CuCN·2LiCl (1.0 M in THF, 20 µL, 0.02 mmol, 0.1 equiv) in THF. The reaction was instantaneously quenched by the addition of *sat. aq.* NH_4Cl. The aqueous phase was extracted three times with EtOAc (3×10 mL) and the combined organic phases were dried over anhydrous $MgSO_4$ and filtrated. After removal of the solvent *in vacuo*, flash chromatographical purification (silica gel, isohexane:EtOAc = 9:1) afforded the title compound **10ee** as a yellow oil (32 mg, 0.15 mmol, 74% yield; 2*Z/*2*E* = 77/23).

^{1}H-NMR (400 MHz, $CDCl_3$):
(2*E*,4*E*)-2-(Cyclohex-2-en-1-yl)nona-2,4-dienenitrileenenitrile: δ = 6.69 (d, *J*=11.2, 1H), 6.35 (ddd, *J*=14.5, 11.2, 1.5, 1H), 6.05 (ddt, *J*=14.4, 11.4, 7.0, 1H), 5.91 (ddt, *J*=10.1, 4.6, 2.9, 1H), 5.46 (dq, *J*=10.1, 2.4, 1H), 3.34 (dh, *J*=7.9, 2.7, 1H), 2.18 (qd, *J*=7.2, 1.4, 2H), 2.04 (ddddd, *J*=12.2, 10.7, 8.7, 5.7, 2.9, 2H), 1.92 – 1.78 (m, 2H), 1.62 (qdd, *J*=14.2, 5.7, 3.7, 2H), 1.47 – 1.26 (m, 4H), 0.90 (td, *J*=7.2, 1.9, 3H).
(2Z,4*E*)-2-(Cyclohex-2-en-1-yl)nona-2,4-dienenitrileenenitrile: δ = 6.57 (d, *J*=11.0, 1H), 6.53 – 6.43 (m, 1H), 6.08 – 5.99 (m, 1H), 5.91 (ddt, *J*=10.1, 4.6, 2.9, 1H), 5.53 (dq, *J*=10.2, 2.5, 1H), 2.98 (tq, *J*=5.6, 2.8, 1H), 2.18 (qd, *J*=7.2, 1.4, 2H), 2.04 (ddddd, *J*=12.2, 10.7, 8.7, 5.7, 2.9, 2H), 1.92 – 1.79 (m, 2H), 1.62 (qdd, *J*=14.2, 5.7, 3.7, 2H), 1.46 – 1.29 (m, 4H), 0.90 (td, *J*=7.2, 1.9, 3H).
^{13}C-NMR (100 MHz, $CDCl_3$):
(2*E*,4*E*)-2-(Cyclohex-2-en-1-yl)nona-2,4-dienenitrileenenitrile: δ / ppm = 145.1, 143.6, 130.8, 126.9, 124.2, 120.3, 116.1, 35.3, 33.0, 31.0, 28.5, 24.6, 22.4, 21.4, 14.0.
(2*Z*,4*E*)-2-(Cyclohex-2-en-1-yl)nona-2,4-dienenitrileenenitrile: δ / ppm = 144.2, 143.6, 131.0, 127.1, 126.4, 118.2, 115.5, 39.6, 32.8, 31.6, 31.1, 29.8, 24.9, 22.4, 20.1.
IR (Diamond-ATR, neat): $\tilde{\nu}$ / cm^{-1} = 2955, 2930, 2871, 2861, 2208, 1712, 1636, 1455, 1448, 1433, 1378, 1362, 1249, 1220, 1183, 1138, 1079, 1046, 973, 903, 724.
MS (EI, 70 eV): *m/z* (%) = 215 (19), 172 (15), 158 (40), 156 (15), 145 (96), 144 (80), 143 (37), 132 (20), 131 (25), 130 (89), 129 (18), 128 (19), 118 (34), 117 (100), 116 (79), 115 (38), 104 (31), 103 (18), 92 (46), 91 (42), 90 (14), 81 (15), 79 (30), 77 (23).

HRMS (EI): *m/z* calc. for [$C_{15}H_{21}N$]: 215.1674; found: 215.1669.

2-(Hydroxy(2-methoxyphenyl)methyl)nona-2,4-dienenitrile (10ek)

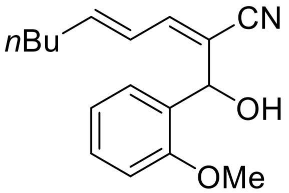

According to the **TP1**, a solution of (*2E,4E*)-nona-2,4-dienenitrile (**8e**, 0.20 M, 27 mg, 0.20 mmol, 1.0 equiv; 2*E*/2*Z* = 69/31) in THF (total volume: 1 mL) and a solution of NaDA (0.24 M in DMEA, 0.24 mmol, 1.2 equiv) were prepared. The precooled solutions were mixed with an overall 10 mL·min^{-1} flow-rate in a T-mixer. The combined stream passed a 0.02 mL reactor tube (0.12 s, –78 °C) and was subsequently injected in a flask containing a stirred solution of *m*-anisaldehyde (**3k**, 41 mg, 0.30 mmol, 1.5 equiv) in THF. The reaction was instantaneously quenched by the addition of *sat. aq.* NH_4Cl. The aqueous phase was extracted three times with EtOAc (3×10 mL) and the combined organic phases were dried over anhydrous $MgSO_4$ and filtrated. After removal of the solvent *in vacuo*, flash chromatographical purification (silica gel, isohexane:EtOAc = 9:1) afforded the title compound **10ek** as a off-white solid (45 mg, 0.16 mmol, 82% yield; 2*Z/2E* = 76/24).

^{1}H-NMR (400 MHz, $CDCl_3$):

(*2Z,4E*)-2-(Hydroxy(2-methoxyphenyl)methyl)nona-2,4-dienenitrile: δ / ppm = 7.48 (dd, *J*=7.6, 1.7, 1H), 7.31 (ddd, *J*=8.2, 7.4, 1.7, 1H), 7.01 (td, *J*=7.5, 1.1, 1H), 6.90 (dd, *J*=8.2, 1.0, 1H), 6.75 (d, *J*=11.4, 1H), 6.57 (ddt, *J*=14.5, 11.4, 1.5, 1H), 6.11 (dt, *J*=14.5, 7.0, 1H), 5.91 (d, *J*=2.6, 1H), 3.85 (s, 3H), 2.93 (s, 1H), 2.21 (qd, *J*=7.0, 1.4, 2H), 1.48 – 1.27 (m, 4H), 0.92 (t, *J*=7.2, 3H).

(*2E,4E*)-2-(Hydroxy(2-methoxyphenyl)methyl)nona-2,4-dienenitrile: δ / ppm = 7.39 – 7.28 (m, 2H), 7.01 (td, *J*=7.5, 1.1, 1H), 6.91 (dd, *J*=8.3, 1.1, 1H), 6.81 (dt, *J*=11.2, 1.0, 1H), 6.50 (ddt, *J*=15.0, 11.1, 1.5, 1H), 6.11 (dt, *J*=14.6, 7.1, 1H), 5.53 (s, 1H), 3.86 (s, 3H), 3.03 (d, *J*=6.1, 1H), 2.23 – 2.15 (m, 2H), 1.47 – 1.28 (m, 4H), 0.90 (t, *J*=7.2, 3H).

^{13}C-NMR (100 MHz, $CDCl_3$):

(*2Z,4E*)-2-(Hydroxy(2-methoxyphenyl)methyl)nona-2,4-dienenitrile: δ / ppm = 156.5, 146.3, 144.5, 129.7, 128.5, 127.2, 124.7, 121.3, 119.2, 114.1, 110.8, 66.1, 55.4, 33.0, 30.9, 22.4, 14.0.

(*2E,4E*)-2-(Hydroxy(2-methoxyphenyl)methyl)nona-2,4-dienenitrile: δ / ppm = 156.6, 145.4, 144.0, 129.7, 128.1, 127.7, 126.6, 121.2, 116.7, 113.8, 110.9, 70.8, 55.5, 32.7, 30.8, 22.3, 13.9.

IR (Diamond-ATR, neat): $\tilde{\nu}$ / cm^{-1} = 3436, 2961, 2928, 2856, 2838, 2220, 1633, 1599, 1588, 1488, 1462, 1436, 1407, 1383, 1370, 1324, 1304, 1285, 1251, 1235, 1200, 1188, 1173, 1161, 1148, 1119, 1053, 1046, 1023, 981, 947, 940, 931, 917, 860, 843, 789, 754, 724, 688.

MS (EI, 70 eV): *m/z* (%) = 240 (13), 228 (32), 214 (18).

HRMS (EI): *m/z* calc. for [$C_{17}H_{21}NO_2$]: 271.1572; found: 271.1506.

m.p. (°C): 84.1 – 85.2.

(*E*)-2-(Hydroxyadamantan-2-yl)-3-phenylacrylonitrile (4ao)

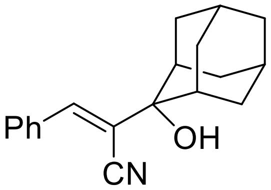

According to the **TP2**, a solution of cinnamonitrile (**1a**, 0.20 M, 26 mg, 0.20 mmol, 1.0 equiv; *E/Z* > 99/1) in THF (total volume: 1 mL) and a solution of NaTMP (0.26 M in hexane, 0.26 mmol, 1.3 equiv) were prepared. The precooled solutions were mixed with an overall 10 mL·min^{-1} flow-rate in a T-mixer. The combined stream passed a 0.02 mL reactor tube (0.12 s, –78 °C) and was subsequently injected in a flask containing a stirred solution of 2-adamantanone (**3o**, 45 mg, 0.30 mmol, 1.5 equiv) in THF. The reaction was instantaneously quenched by the addition of *sat. aq.* NH_4Cl. The aqueous phase was extracted three times with EtOAc (3×10 mL) and the combined organic phases were dried over anhydrous $MgSO_4$ and filtrated. After removal of the solvent *in vacuo*, flash chromatographical purification (silica gel, isohexane:EtOAc = 9:1) afforded the title compound **4ao** as a colorless oil (44 mg, 0.16 mmol, 79% yield; *E*/*Z* > 99/1).

^{1}H-NMR (400 MHz, $CDCl_3$): δ / ppm =7.79 (dd, *J* = 7.4, 2.3, 2H), 7.43 (dt, *J* = 4.8, 2.8, 3H), 7.22 (s, 1H), 2.39 (t, *J* = 2.9, 2H), 2.30 (d, *J* = 12.9, 2H), 1.93 – 1.79 (m, 7H), 1.73 (h, *J* = 3.9, 3.0, 4H).

^{13}C-NMR (100 MHz, $CDCl_3$): δ / ppm = 143.0, 133.6, 130.6, 129.2 (2C), 129.0 (2C), 118.4, 117.8, 75.7, 37.4, 35.7 (2C), 34.6 (2C), 32.7 (2C), 27.1, 26.9.

IR (Diamond-ATR, neat): $\tilde{\nu}$ / cm^{-1} = 3432, 3373, 2932, 2912, 2904, 2852, 2212, 1468, 1448, 1352, 1215, 1144, 1106, 1085, 1078, 1050, 1043, 1018, 1009, 968, 950, 934, 911, 765, 745, 687, 676.

MS (EI, 70 eV): *m/z* (%) = 279 (19), 278 (15), 207 (14), 173 (78), 156 (10), 152 (10), 151 (95), 140 (10), 130 (21), 129 (11), 128 (14), 115 (16), 93 (17), 91 (100), 81 (13), 79 (28), 77 (16).

HRMS (EI): *m/z* calc. for [$C_{19}H_{21}NO$]: 279.1623; found: 279.1620.

(*E*)-2-Benzylidene-3-hydroxy-3-phenylbutanenitrile (4as)

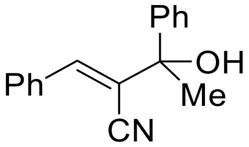

According to the **TP2**, a solution of cinnamonitrile (**1a**, 0.20 M, 26 mg, 0.20 mmol, 1.0 equiv; *E/Z* > 99/1) in THF (total volume: 1 mL) and a solution of NaTMP (0.26 M in hexane, 0.26 mmol, 1.3 equiv) were prepared. The precooled solutions were mixed with an overall 10 mL·min^{-1} flow-rate in a T-mixer. The combined stream passed a 0.02 mL reactor tube (0.12 s, –78 °C) and was subsequently injected in a flask containing a stirred solution of acetophenone (**3s**, 36 mg, 0.30 mmol, 1.5 equiv) in THF. The reaction was instantaneously quenched by the addition of *sat. aq.* NH_4Cl. The aqueous phase was extracted three times with EtOAc (3×10 mL) and the combined organic phases were dried over anhydrous $MgSO_4$ and filtrated. After removal of the solvent *in vacuo*, flash chromatographical purification (silica gel, isohexane:EtOAc = 9:1) afforded the title compound **4as** as a slightly yellow oil (29 mg, 0.12 mmol, 58% yield; *E/Z* > 99/1).

^{1}H-NMR (400 MHz, $CDCl_3$): δ / ppm = 7.70 – 7.56 (m, 2H), 7.41 (d, *J* = 7.6, 2H), 7.31 – 7.20 (m, 6H), 7.13 (s, 1H), 2.25 (s, 1H), 1.84 (s, 3H).

^{13}C-NMR (100 MHz, $CDCl_3$): δ / ppm = 143.8, 141.6, 133.3, 130.5, 129.2 (2C), 129.0 (2C), 128.9 (2C), 128.4, 125.7 (2C), 119.1, 117.9, 75.8, 28.7.

IR (Diamond-ATR, neat): $\tilde{\nu}$ / cm^{-1} = 3432, 2984, 2211, 1704, 1621, 1494, 1448, 1420, 1362, 1286, 1223, 1163, 1159, 1132, 1107, 1092, 1072, 1029, 926, 915, 902, 758, 737, 692.

MS (EI, 70 eV): *m/z* (%) = 249 (10), 248 (16), 235 (12), 234 (69), 207 (15), 206 (100), 179 (31), 178 (16), 156 (73), 130 (13), 129 (12), 128 (13), 121 (23), 105 (15), 77 (11).

HRMS (EI): *m/z* calc. for [$C_{17}H_{15}NO$]: 249.1154; found: 249.1152.

(*E*)-2-(1-Hydroxy-1,2,3,4-tetrahydronaphthalen-1-yl)-3-phenylacrylonitrile (4aq)

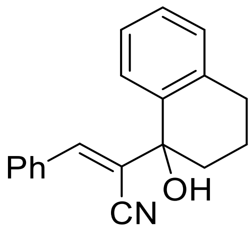

According to the **TP2**, a solution of cinnamonitrile (**1a**, 0.20 M, 26 mg, 0.20 mmol, 1.0 equiv; *E/Z* > 99/1) in THF (total volume: 1 mL) and a solution of NaTMP (0.26 M in hexane, 0.26 mmol, 1.3 equiv) were prepared. The precooled solutions were mixed with an overall 10 mL·min^{-1} flow-rate in a T-mixer. The combined stream passed a 0.02 mL reactor tube (0.12 s, –78 °C) and was subsequently injected in a flask containing a stirred solution of α-tetralone (**3q**, 44 mg, 0.30 mmol, 1.5 equiv) in THF. The reaction was instantaneously quenched by the addition of *sat. aq.* NH_4Cl. The aqueous phase was extracted three times with EtOAc (3×10 mL) and the combined organic phases were dried over anhydrous $MgSO_4$ and filtrated. After removal of the solvent *in vacuo*, flash chromatographical purification (silica gel, isohexane:EtOAc = 9:1) afforded the title compound **4aq** as a brown viscose oil (37 mg, 0.13 mmol, 67% yield; *E/Z* > 99/1).

^{1}H-NMR (400 MHz, $CDCl_3$): δ / ppm = 7.83 – 7.75 (m, 2H), 7.47 – 7.38 (m, 4H), 7.35 (dd, *J* = 7.4, 1.8 Hz, 1H), 7.25 (pd, *J* = 7.3, 1.7 Hz, 2H), 7.18 (dd, *J* = 7.5, 1.6 Hz, 1H), 2.88 (dd, *J* = 7.4, 5.1 Hz, 2H), 2.38 (ddd, *J* = 14.2, 11.1, 3.3 Hz, 1H), 2.22 (s, 1H), 2.13 – 1.82 (m, 3H).

^{13}C-NMR (100 MHz, $CDCl_3$): δ / ppm = 142.8, 138.0, 137.7, 133.4, 130.4, 129.7, 129.2 (2C), 129.0 (2C), 128.9, 127.7, 127.1, 119.1, 118.0, 75.1, 37.2, 29.5, 19.3.

IR (Diamond-ATR, neat): $\tilde{\nu}$ / cm^{-1} = 3426, 3061, 3027, 2937, 2868, 2212, 1621, 1602, 1576, 1489, 1448, 1360, 1328, 1290, 1278, 1209, 1185, 1161, 1127, 1114, 1104, 1081, 1058, 1040, 1021, 990, 935, 911, 900, 879, 858, 850, 770, 754, 734, 690, 671.

MS (EI, 70 eV): *m/z* (%) = 274 (16), 246 (11), 148 (12), 147 (100), 129 (24), 91 (28).

HRMS (EI): *m/z* calc. for [$C_{19}H_{17}NO$]: 275.1310; found: 275.1303.

(*Z*)-3-Ethoxy-2-(hydroxy(2-methoxyphenyl)methyl)acrylonitrile (7gk)

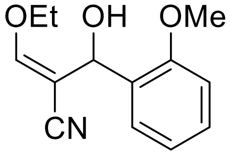

According to the **TP2**, a solution of 3-ethoxyacrylonitrile (**5g**, 0.20 M, 19 mg, 0.20 mmol, 1.0 equiv; *E/Z* = 68/32) in THF (total volume: 1 mL) and a solution of NaTMP (0.24 M in hexane, 0.24 mmol, 1.2 equiv) were prepared. The precooled solutions were mixed with an overall 10 mL·min^{-1} flow-rate in a T-mixer. The combined stream passed a 0.02 mL reactor tube (0.12 s, –78 °C) and was subsequently injected in a flask containing a stirred solution of *m*-anisaldehyde (**3k**, 41 mg, 0.30 mmol, 1.5 equiv) in THF. The reaction was instantaneously quenched by the addition of *sat. aq.* NH_4Cl. The aqueous phase was extracted three times with EtOAc (3×10 mL) and the combined organic phases were dried over anhydrous $MgSO_4$ and filtrated. After removal of the solvent *in vacuo*, flash chromatographical purification (silica gel, isohexane:EtOAc = 7:3) afforded the title compound **7gk** as a yellow solid (34 mg, 0.16 mmol, 78% yield; *Z*/*E* > 99/1).

^{1}H-NMR (400 MHz, $CDCl_3$): δ / ppm = 7.77 (d, *J* = 8.9 Hz, 1H), 7.71 – 7.66 (m, 1H), 7.36 (td, *J* = 7.5, 0.9 Hz, 1H), 7.31 (d, *J* = 8.3 Hz, 1H), 7.26 (s, 1H), 6.17 (s, 1H), 4.50 – 4.43 (m, 2H), 4.28 (s, 3H), 1.72 (t, *J* = 7.1 Hz, 3H), 1.66 (d, *J* = 5.9 Hz, 1H).

^{13}C-NMR (100 MHz, $CDCl_3$): δ / ppm = 158.6, 157.0, 129.4, 128.9, 127.7, 121.0, 118.2, 111.0, 97.3, 71.3, 65.9, 55.5, 15.4.

IR (Diamond-ATR, neat): $\tilde{\nu}$ / cm^{-1} = 3387, 2935, 2840, 2212, 1637, 1599, 1587, 1488, 1462, 1438, 1392, 1371, 1355, 1325, 1306, 1290, 1242, 1216, 1190, 1171, 1161, 1146, 1108, 1091, 1027, 1013, 949, 940, 920, 892, 855, 790, 768, 756, 732, 688.

MS (EI, 70 eV): *m/z* (%) = 233 (44), 232 (10), 204 (12), 202 (15), 188 (11), 187 (12), 174 (14), 156 (15), 137 (100), 135 (68), 121 (19), 109 (30), 108 (19), 107 (61), 98 (11), 94 (12), 91 (14), 78 (11), 77 (37), 65 (11), 43 (12).

HRMS (EI): *m/z* calc. for [$C_{13}H_{15}NO_3$]: 233.1052; found: 233.1048.

m.p. (°C): 42.9 – 47.0.

(*Z*)-3-Ethoxy-2-(hydroxydiphenylmethyl)acrylonitrile (7gg)

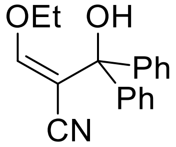

According to the **TP2**, a solution of 3-ethoxyacrylonitrile (**5g**, 0.20 M, 19 mg, 0.20 mmol, 1.0 equiv; *E/Z* = 68/32) in THF (total volume: 1 mL) and a solution of NaTMP (0.24 M in hexane, 0.24 mmol, 1.2 equiv) were prepared. The precooled solutions were mixed with an overall 10 mL·min^{-1} flow-rate in a T-mixer. The combined stream passed a 0.02 mL reactor tube (0.12 s, –78 °C) and was subsequently injected in a flask containing a stirred solution of benzophenone (**3g**, 55 mg, 0.30 mmol, 1.5 equiv) in THF. The reaction was instantaneously quenched by the addition of *sat. aq.* NH_4Cl. The aqueous phase was extracted three times with EtOAc (3×10 mL) and the combined organic phases were dried over anhydrous $MgSO_4$ and filtrated. After removal of the solvent *in vacuo*, flash chromatographical purification (silica gel, isohexane:EtOAc = 7:3) afforded the title compound **7gg** as a brown viscose oil (36 mg, 0.13 mmol, 65% yield; *Z*/*E* > 99/1).

^{1}H-NMR (400 MHz, $CDCl_3$): δ / ppm = 7.42 – 7.28 (m, 10H), 6.62 (s, 1H), 3.99 (q, *J* = 7.1 Hz, 2H), 2.78 (s, 1H), 1.30 (t, *J* = 7.1 Hz, 3H).

^{13}C-NMR (100 MHz, $CDCl_3$): δ / ppm = 162.2, 143.8 (2C), 128.5 (4C), 128.3 (2C), 127.3 (4C), 116.4, 98.7, 78.8, 71.1, 15.3.

IR (Diamond-ATR, neat): $\tilde{\nu}$ / cm^{-1} = 3428, 2983, 2211, 1628, 1600, 1492, 1447, 1395, 1370, 1304, 1218, 1183, 1144, 1107, 1088, 1019, 935, 917, 893, 768, 751, 737, 698, 672, 653.

MS (EI, 70 eV): *m/z* (%) = 183 (16), 182 (34), 105 (100), 77 (76), 69 (14), 68 (18).

HRMS (EI): *m/z* calc. for [$C_{18}H_{17}NO_2$]: 279.1259; found: 279.1255.

2-(Cyclohex-2-en-1-yl)-3,7-dimethylocta-2,6-dienenitrile (10ae)

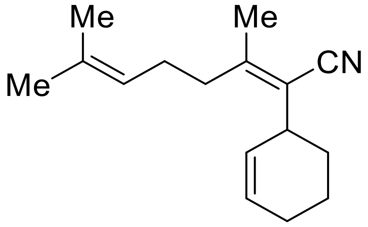

According to the **TP2**, a solution of geranyl nitrile (**8a**, 0.20 M, 30 mg, 0.20 mmol, 1.0 equiv; *E/Z* = 50/50) in THF (total volume: 1 mL) and a solution of NaTMP (0.26 M in hexane, 0.26 mmol, 1.3 equiv) were prepared. The precooled solutions were mixed with an overall 10 mL·min^{-1} flow-rate in a T-mixer. The combined stream passed a 0.02 mL reactor tube (0.12 s, –78 °C) and was subsequently injected in a flask containing a stirred solution of cyclohexene bromide (**3e**, 30 mg, 0.30 mmol, 1.5 equiv) and CuCN·2LiCl (1.0 M in THF, 20 µL, 0.02 mmol, 0.1 equiv) in THF. The reaction was instantaneously quenched by the addition of *sat. aq.* NH_4Cl. The aqueous phase was extracted three times with EtOAc (3×10 mL) and the combined organic phases were dried over anhydrous $MgSO_4$ and filtrated. After removal of the solvent *in vacuo,* flash chromatographical purification (silica gel, isohexane) afforded the title compound **10ae** as a yellow oil (25 mg, 0.11 mmol, 54% yield; *Z*/*E* = 52/48).

(*E*/*Z*)-2-(Cyclohex-2-en-1-yl)-3,7-dimethylocta-2,6-dienenitrile:

^{1}H-NMR (400 MHz, $CDCl_3$): δ / ppm = 5.88 (ddt, *J* = 10.1, 5.2, 2.8 Hz, 1H), 5.43 (dddd, *J* = 11.2, 8.7, 2.7, 1.3 Hz, 1H), 5.14 – 5.03 (m, 1H), 3.21 (dddt, *J* = 18.0, 8.8, 6.0, 3.0 Hz, 1H), 2.48 – 2.36 (m, 1H), 2.31 – 2.12 (m, 3H), 2.07 (s, 2H), 1.86 (s, 3H), 1.69 (d, *J* = 1.4 Hz, 4H), 1.66 – 1.53 (m, 6H).

^{13}C-NMR (100 MHz, $CDCl_3$): δ / ppm = 154.9, 154.8, 133.4, 133.1, 130.4, 130.3, 127.6, 127.5, 122.7, 122.6, 118.7, 118.4, 115.4, 115.1, 38.8, 36.2, 36.0, 34.0, 28.6, 28.2, 26.6, 26.5, 25.8, 25.8, 24.6, 24.6, 23.0, 21.7, 21.6, 18.4, 17.8 (2C).

IR (Diamond-ATR, neat): $\tilde{\nu}$ / cm^{-1} = 3022, 2964, 2927, 2860, 2840, 2209, 1624, 1447, 1377, 1109, 978, 901, 886, 871, 823, 764, 722.

MS (EI, 70 eV): *m/z* (%) = 214 (70), 201 (67), 200 (22), 187 (29), 186 (83), 173 (65), 172 (42), 161 (29), 160 (94), 159 (100), 158 (89), 146 (93), 145 (23), 144 (92), 133 (47), 132 (75), 131 (76), 130 (71), 119 (22), 118 (66), 117 (24), 116 (33), 91 (27), 79 (19), 69 (50), 41 (36).

HRMS (EI): *m/z* calc. for [$C_{16}H_{23}N$]: 229.1830; found: 229.1827.

***Tert*-butyl (*E*)-2-(cyclohex-2-en-1-yl)-3-phenylacrylate (13be)**

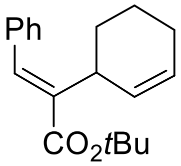

According to the **TP1**, a solution *tert*-butyl cinnamate (**11b**, 0.20 M, 41 mg, 0.20 mmol, 1.0 equiv; *E*/*Z* > 99/1) in THF (total volume: 1 mL) and a solution of NaDA (0.24 M in DMEA, 0.24 mmol, 1.2 equiv) were prepared. The precooled solutions were mixed with an overall 10 mL·min^{-1} flow-rate in a T-mixer. The combined stream passed a 0.02 mL reactor tube (0.12 s, –78 °C) and was subsequently injected in a flask containing a stirred solution of cyclohexene bromide (**3e**, 30 mg, 0.30 mmol, 1.5 equiv) and CuCN·2LiCl (1.0 M in THF, 20 µL, 0.02 mmol, 0.1 equiv) in THF. The reaction was instantaneously quenched by the addition of *sat. aq.* NH_4Cl. The aqueous phase was extracted three times with EtOAc (3×10 mL) and the combined organic phases were dried over anhydrous $MgSO_4$ and filtrated. After removal of the solvent *in vacuo*, flash chromatographical purification (silica gel, isohexane:EtOAc = 9:1) afforded the title compound **13be** as a yellow oil (23 mg, 0.12 mmol, 61% yield; *E/Z* > 99/1).

^{1}H-NMR (400 MHz, $CDCl_3$): δ / ppm = 7.58 (s, 1H), 7.40 – 7.33 (m, 2H), 7.33 – 7.27 (m, 3H), 5.68 (dddd, *J*=10.1, 5.0, 2.7, 0.9, 1H), 5.57 – 5.50 (m, 1H), 3.64 – 3.54 (m, 1H), 2.14 – 1.93 (m, 3H), 1.88 – 1.80 (m, 1H), 1.76 (dddd, *J*=11.0, 5.4, 2.7, 1.3, 1H), 1.62 – 1.55 (m, 1H), 1.52 (s, 9H).

^{13}C-NMR (100 MHz, $CDCl_3$): δ / ppm = 167.5, 138.7, 138.6, 136.2, 130.5, 129.1 (2C), 128.4 (2C), 128.0, 126.2, 80.9, 36.2, 28.3, 28.3 (3C), 24.8, 22.9.

IR (Diamond-ATR, neat): $\tilde{\nu}$ / cm^{-1} = 3020, 2976, 2929, 2861, 2835, 1705, 1626, 1493, 1446, 1391, 1366, 1302, 1243, 1223, 1206, 1161, 1138, 1106, 1075, 1051, 977, 928, 886, 851, 841, 776, 761, 742, 720, 695.

MS (EI, 70 eV): *m/z* (%) = 228 (16), 185 (11), 184 (15), 183 (100), 167 (13), 155 (13), 141 (73), 129 (15), 115 (10).

HRMS (EI): *m/z* calc. for [$C_{19}H_{24}O_2$]: 284.1776; found: 227.1064 [M – *t*Bu].

Ethyl (*E*)-2-(2-hydroxyadamantan-2-yl)-3-phenylacrylate (13ao)

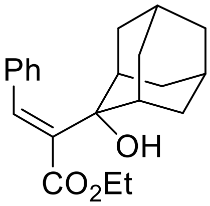

According to **TP3**, a solution of ethyl cinnamate (**11a**, 0.20 M, 35 mg, 0.20 mmol, 1.0 equiv; *E*/*Z* > 99/1) and adamantanone (**3o**, 45 mg, 0.30 mmol, 1.5 equiv) in THF (total volume: 1 mL) and a solution of NaDA (0.24 M in DMEA, 0.24 mmol, 1.2 equiv) were prepared. The precooled solutions were mixed with an overall 10 $mL \cdot min^{-1}$ flow-rate in a T-mixer. The combined stream passed a 0.02 mL reactor tube (0.12 s, –78 °C) and was subsequently injected in a flame-dried flask. The reaction was stirred for 30 min at −78 °C and quenched by the addition of *sat. aq.* NH_4Cl. The aqueous phase was extracted three times with EtOAc (3×10 mL) and the combined organic phases were dried over anhydrous $MgSO_4$ and filtrated. After removal of the solvent *in vacuo*, flash chromatographical purification (silica gel, isohexane:EtOAc = 9:1) afforded the title compound **13ao** as a white solid (43 mg, 0.13 mmol, 66% yield; *E/Z* > 99/1).

^{1}H-NMR (400 MHz, $CDCl_3$): δ / ppm = 7.34 – 7.23 (m, 5H), 6.86 (s, 1H), 4.12 (q, *J*=7.1, 2H), 3.31 (s, 1H), 2.45 – 2.32 (m, 2H), 2.10 (d, *J*=4.1, 2H), 1.96 (d, *J*=12.9, 2H), 1.86 (dp, *J*=6.5, 3.2, 2H), 1.76 (dqd, *J*=13.0, 2.7, 1.3, 2H), 1.71 (d, *J*=3.5, 2H), 1.62 (dtd, *J*=12.8, 3.5, 1.5, 2H), 1.06 (t, *J*=7.2, 3H).
^{13}C-NMR (100 MHz, $CDCl_3$): δ / ppm = 171.2, 139.3, 136.1, 131.1, 128.4 (2C), 128.2 (2C), 128.2, 76.1, 61.2, 37.7, 35.4 (2C), 34.8 (2C), 32.8 (2C), 27.5, 27.2, 13.8.
IR (Diamond-ATR, neat): $\tilde{\nu}$ / cm^{-1} = 3407, 2958, 2917, 2903, 2856, 1708, 1678, 1636, 1468, 1459, 1446, 1410, 1392, 1375, 1352, 1338, 1319, 1296, 1284, 1231, 1208, 1130, 1118, 1110, 1104, 1095, 1072, 1040, 1003, 962, 936, 926, 911, 874, 857, 756, 732, 702, 683.
MS (EI, 70 eV): *m/z* (%) = 280 (73), 279 (38), 253 (74), 252 (59), 251 (71), 235 (29), 225 (73), 224 (100), 223 (41), 204 (30), 179 (41), 167 (35), 162 (78), 159 (29), 143 (28), 141 (38), 131 (59), 129 (33), 128 (25), 121 (31), 115 (33), 103 (32), 93 (28), 91 (72), 79 (44), 77 (25).
HRMS (EI): *m/z* calc. for [$C_{21}H_{26}O_3$]: 326.1882; found: 308.1768 [M – H_2O].
m.p. (°C): 64.6 – 65.7.

3'-Methoxy-5'*H*-spiro[adamantane-2,2'-furan]-5'-one (13co)

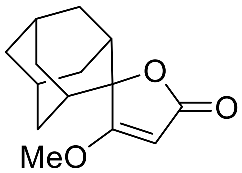

According to **TP3**, a solution of methyl Methyl (*E*)-3-methoxyacrylate (**11c**, 0.20 M, 23 mg, 0.20 mmol, 1.0 equiv; *E*/*Z* > 99/1) and adamantanone (**3o**, 45 mg, 0.30 mmol, 1.5 equiv) in THF (total volume: 1 mL) and a solution of NaDA (0.24 M in DMEA, 0.24 mmol, 1.2 equiv) were prepared. The precooled solutions were mixed with an overall 10 mL·min^{-1} flow-rate in a T-mixer. The combined stream passed a 0.02 mL reactor tube (0.12 s, –78 °C) and was subsequently injected in a flame-dried flask. The reaction was stirred for 30 min at –78 °C and quenched by the addition of *sat. aq.* NH_4Cl. The aqueous phase was extracted three times with EtOAc (3×10 mL) and the combined organic phases were dried over anhydrous $MgSO_4$ and filtrated. After removal of the solvent *in vacuo*, flash chromatographical purification (silica gel, isohexane:EtOAc = 9:1) afforded the title compound **13co** as a white solid (27 mg, 0.12 mmol, 58% yield).

^{1}H-NMR (400 MHz, $CDCl_3$): δ / ppm = 4.99 (s, 1H), 3.87 (s, 3H), 2.41 – 2.24 (m, 4H), 1.89 (dt, *J*=20.0, 3.2, 4H), 1.74 (qd, *J*=4.3, 1.9, 4H), 1.70 – 1.58 (m, 2H).

^{13}C-NMR (100 MHz, $CDCl_3$): 187.4, 171.7, 88.2, 88.1, 59.5, 37.9, 36.4 (2C), 34.7 (2C), 33.3 (2C), 26.7, 26.3.

IR (Diamond-ATR, neat): $\tilde{\nu}$ / cm^{-1} = 3111, 2946, 2908, 2895, 2864, 2851, 1754, 1619, 1471, 1455, 1436, 1377, 1346, 1315, 1286, 1254, 1203, 1177, 1113, 1104, 1066, 1050, 1041, 1018, 1009, 1002, 959, 954, 934, 924, 888, 866, 826, 802, 777, 753, 739, 709, 666.

MS (EI, 70 eV): *m/z* (%) = 235 (14), 234 (100), 233 (14), 216 (15), 206 (38), 202 (67), 201 (16), 192 (20), 184 (52), 177 (16), 175 (13), 174 (31), 169 (25), 163 (13), 161 (18), 160 (28), 157 (17), 129 (15), 127 (21), 125 (25), 117 (17), 115 (14), 93 (13), 91 (29), 79 (21).

HRMS (EI): *m/z* calc. for [$C_{14}H_{18}O_3$]: 234.1256; found: 234.1251.

m.p. (°C): 128.6 – 131.4.

Additional experiments under batch and Barbier-type conditions:

Entry	Product	Barbier-type	batch conditions	sequential flow conditions
1	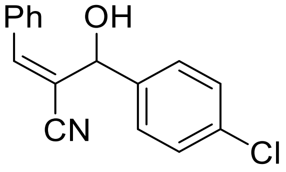 **4ab**	only traces of product according to GCMS	only traces of product according to GCMS	92% isolated yield
2	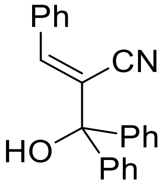 **4ag**	only traces of product according to GCMS	only traces of product according to GCMS	82% isolated yield
3	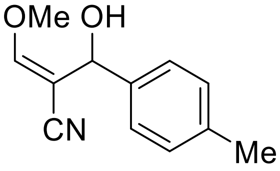 **7fd**	only traces of product according to GCMS	74% isolated yield Z/E = 9/1	98% isolated yield Z/E = 99/1
4	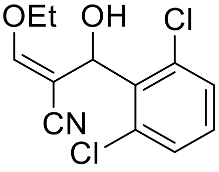 **7gc**	26% isolated yield	72% isolated yield	95% isolated yield

Table SI 2. Summarized Barbier-type, batch and sequential flow results: Barbier-type experiments were conducted according to **TP 3**, experiments under batch conditions were conducted according to **TP 4,** sequential flow condtions according to **TP 1**.

Preparation of the starting materials

Typical procedure for the synthesis of aryl acrylonitriles

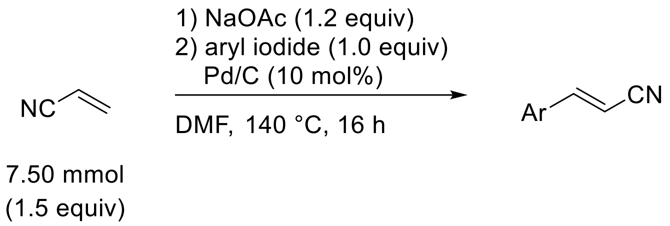

A suspension of acrylonitrile (0.40 g, 7.5 mmol, 1.5 equiv) and NaOAc (0.49 g, 6.0 mmol, 1.2 equiv) in dry DMF (15 mL) was prepared. To this suspension a solution of the aryliodine (5.0 mmol, 1.0 equiv) in dry DMF (10 mL) was added as well as 10 weight-% Pd/C (53 mg, 0.05 mmol, 0.01 equiv). The mixture was stirred at 140 °C over night. EtOAc was added to the reaction mixture. The mixture was filtrated and the filtrate was washed with an aqueous LiCl solution (10%, 3 x 30 mL), the combined aqueous layers were extracted with EtOAc (3 x 50 mL). The combined organic layers were dried over $MgSO_4$. Solvents were removed *in vacuo*. The crude product was purified by column chromatography.[4]

[4] P. An, Z. Yu, Q. Lin, *Org. Lett.* **2013**, *15*, 5496.

3-(4-Methoxyphenyl)acrylonitrile (5a)

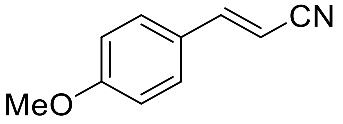

According to the typical procedure for the preparation of aryl acrylonitriles, 1-iodo-4-methoxybenzene (1.16 g, 5.0 mmol, 1.0 equiv) in DMF (10 mL) was added to a mixture of acrylonitrile (0.40 g, 7.5 mmol, 1.5 equiv) and NaOAc (0.49 g, 6.0 mmol, 1.2 equiv) in DMF (15 mL), 10 weight-% Pd/C (53 mg. 0.05 mmol, 0.01 equiv) was added and the mixture was stirred over night at 140 °C. The crude product was purified by column chromatography (isohexane:EtOAc = 9:1) to give the titel compound as colorless solid (0.62 g, 3.9 mmol, 78% yield; *E*/*Z* = 76/24).

^{1}H-NMR (400 MHz, $CDCl_3$):
(*E*)- 3-(4-Methoxyphenyl)acrylonitrile: δ / ppm = 7.40 (d, *J* = 8.7 Hz, 2H), 7.34 (d, *J* = 16.6 Hz, 1H), 6.91 (d, *J* = 8.7 Hz, 2H), 5.72 (d, *J* = 16.6 Hz, 1H), 3.85 (s, 3H).
(*Z*)- 3-(4-Methoxyphenyl)acrylonitrile: δ / ppm = 7.80 (d, *J* = 8.8 Hz, 2H), 7.04 (d, *J* = 12.1 Hz, 1H), 6.95 (d, *J* = 8.9 Hz, 2H), 5.29 (d, *J* = 12.1 Hz, 1H), 3.86 (s, 3H).
^{13}C-NMR (100 MHz, $CDCl_3$):
(*E*)- 3-(4-Methoxyphenyl)acrylonitrile: δ / ppm = 150.8, 140.6, 137.5, 130.4, 128.5 (2C), 125.0 (2C), 94.8, 19.9.
(Z)- 3-(4-Methoxyphenyl)acrylonitrile: δ / ppm = 148.8, 131.3 (2C), 130.3, 130.2, 126.6, 118.6 (2C), 93.5, 19.8.
IR (Diamond-ATR, neat): $\tilde{\nu}$ / cm^{-1} = 3056, 3026, 2968, 2953, 2932, 2914, 2846, 2212, 1699, 1614, 1599, 1568, 1509, 1490, 1471, 1458, 1440, 1420, 1309, 1297, 1274, 1249, 1173, 1113, 1022, 1003, 985, 976, 963, 942, 853, 844, 827, 818, 806, 769, 728, 719, 710, 690.
MS (EI, 70 eV): *m/z* (%) = 160 (10), 159 (100), 144 (26), 129 (17), 116 (40), 89 (22).
HRMS (EI): *m/z* calc. for [$C_{10}H_9NO$]: 159.0684; found: 159.0680.

3-(3,4-Dimethylphenyl)acrylonitrile (5b)

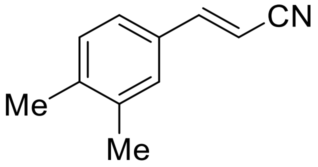

According to the typical procedure for the preparation of arylacrylonitriles, 4-iodo-1,2-dimethylbenzene (1.16 g, 5.0 mmol, 1.0 equiv) in DMF (10 mL) was added to a mixture of acrylonitrile (0.40 g, 7.5 mmol, 1.5 equiv) and NaOAc (0.49 g, 6.0 mmol, 1.2 equiv) in DMF (15 mL), 10 weight-% Pd/C (53 mg. 0.05 mmol, 0.01 equiv) was added and the mixture was stirred over night at 140 °C. The crude product was purified by column chromatography (isohexane:EtOAc = 19:1) to give the titel compound as colorless solid (0.51 g, 3.3 mmol, 65% yield; *E*/*Z* = 79/21).

^{1}H-NMR (400 MHz, $CDCl_3$):

(*E*)-3-(3,4-Dimethylphenyl)acrylonitrile: δ / ppm = 7.34 (d, *J* = 16.6 Hz, 1H), 7.23 – 7.16 (m, 3H), 5.82 (d, *J* = 16.6 Hz, 1H), 2.32 – 2.26 (m, 6H).

(*Z*)-3-(3,4-Dimethylphenyl)acrylonitrile: δ / ppm = 7.61 – 7.54 (m, 2H), 7.17 (t, *J* = 7.2 Hz, 1H), 7.05 (d, *J* = 12.1 Hz, 1H), 5.36 (d, *J* = 12.1 Hz, 1H), 2.30 (s, 6H).

^{13}C-NMR (100 MHz, $CDCl_3$):

(*E*)-3-(3,4-Dimethylphenyl)acrylonitrile: δ / ppm = 150.9, 140.8, 137.6, 131.4, 130.5, 128.6, 125.1, 118.7, 94.9, 20.0, 19.9.

(*Z*)-3-(3,4-Dimethylphenyl)acrylonitrile: δ / ppm = 148.9, 140.5, 137.4, 131.5, 130.4, 130.3, 126.7, 113.9, 93.6, 20.1, 19.9.

IR (Diamond-ATR, neat): $\tilde{\nu}$ / cm^{-1} = 3053, 3018, 2973, 2944, 2920, 2901, 2875, 2856, 2208, 1617, 1603, 1565, 1497, 1479, 1449, 1410, 1386, 1310, 1302, 1296, 1285, 1267, 1239, 1223, 1206, 1124, 1026, 1004, 976, 952, 890, 877, 828, 814, 805, 775, 756, 744, 707.

MS (EI, 70 eV): *m/z* (%) = 158 (12), 157 (100), 156 (59), 143 (10), 142 (97), 140 (10), 129 (38), 128 (16), 115 (49).

HRMS (EI): *m/z* calc. for [$C_{11}H_{11}N$]: 157.0891; found: 157.0886.

3-(2,3-Dihydrobenzo[*b*][1,4]dioxin-6-yl)acrylonitrile (5c)

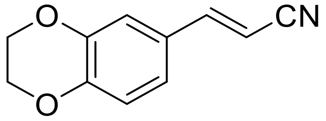

According to the typical procedure for the preparation of arylacrylonitriles, 6-iodo-2,3-dihydrobenzo[*b*][1,4]dioxine (3.93 g, 15.0 mmol, 1.0 equiv) in DMF (30 mL) was added to a mixture of acrylonitrile (1.19 g, 22.5 mmol, 1.5 equiv.) and NaOAc (1.48 g, 18.0 mmol, 1.2 equiv) in DMF (15 mL), 10 weight-% Pd/C (159 mg, 0.15 mmol, 0.01 equiv) was added and the mixture was stirred over night at 140 °C. The crude product was purified by column chromatography (isohexane:EtOAc = 9:1) to give the titel compound as colorless solid (2.05 g, 11.0 mmol, 73% yield; *E*/*Z* = 83/17).

^{1}H-NMR (400 MHz, $CDCl_3$):
(*E*)- 3-(2,3-Dihydrobenzo[*b*][1,4]dioxin-6-yl)acrylonitrile: δ / ppm = 7.27 (d, *J* = 16.5 Hz 1H), 6.99 – 6.93 (m, 2H), 6.87 (d, *J* = 8.3 Hz, 1H), 5.69 (d, *J* = 16.6 Hz, 1H), 4.34 – 4.24 (m, 4H).
(*Z*)- 3-(2,3-Dihydrobenzo[*b*][1,4]dioxin-6-yl)acrylonitrile: δ / ppm = δ 7.37 (d, *J* = 2.1 Hz, 1H), 7.35 (dd, *J* = 8.4, 2.2 Hz, 1H), 6.99 – 6.93 (m, 1H), 6.91 (d, *J* = 8.4 Hz, 1H), 5.30 (d, *J* = 12.1 Hz, 1H), 4.34 – 4.24 (m, 4H).
^{13}C-NMR (100 MHz, $CDCl_3$):
(*E*)- 3-(2,3-Dihydrobenzo[*b*][1,4]dioxin-6-yl)acrylonitrile: δ / ppm = 150.1, 146.5, 144.0, 127.4, 121.6, 118.7, 118.1, 116.1, 94.2, 64.7, 64.3.
(*Z*)- 3-(2,3-Dihydrobenzo[*b*][1,4]dioxin-6-yl)acrylonitrile: δ / ppm = 148.1, 129.0, 123.2, 118.2, 117.8, 117.6, 116.4, 92.8, 64.8, 64.3.
IR (Diamond-ATR, neat): $\tilde{\nu}$ / cm^{-1} = 2924, 2875, 2852, 2212, 2190, 1619, 1603, 1576, 1505, 1455, 1450, 1432, 1392, 1313, 1285, 1256, 1239, 1207, 1166, 1156, 1123, 1060, 1037, 1010, 959, 931, 913, 884, 856, 850, 815, 795, 783, 746, 733, 713, 664.
MS (EI, 70 eV): *m/z* (%) = 188.07 (12), 187.06 (100), 172.04 (27), 131.04 (39), 103.04 (33).
HRMS (EI): *m/z* calc. for [$C_{11}H_8NO_2$]: 187.0633; found: 197.0628.

3-(4-(*Tert*-butyl)phenyl)acrylonitrile (5d)

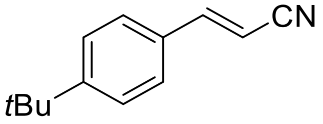

According to the typical procedure for the preparation of arylacrylonitriles, 1-(*tert*-butyl)-4-iodobenzene (3.90 g, 15.0 mmol, 1.0 equiv) in DMF (30 mL) was added to a mixture of acrylonitrile (1.19 g, 22.5 mmol, 1.5 equiv) and NaOAc (1.48 g, 18.0 mmol, 1.2 equiv) in DMF (15 mL), 10 weight-% Pd/C (159 mg. 0.15 mmol, 0.01 equiv) was added and the mixture was stirred over night at 140 °C. The crude product was purified by column chromatography (isohexane:EtOAc = 49:1) to give the titel compound as brownish oil (2.14 g, 10.8 mmol, 72% yield; *E*/*Z* = 79/21).

^{1}H-NMR (400 MHz, $CDCl_3$):
(*E*)-3-(4-(*Tert*-butyl)phenyl)acrylonitrile: δ / ppm = 7.45 – 7.38 (m, 4H), 7.38 (d, *J* = 16.3 Hz, 1H), 5.84 (d, *J* = 16.7 Hz, 1H), 1.33 (s, 9H).
(*Z*)-3-(4-(*Tert*-butyl)phenyl)acrylonitrile: δ / ppm = 7.76 (d, *J* = 8.4 Hz, 2H), 7.47 (d, *J* = 8.5 Hz, 2H), 7.10 (d, *J* = 12.1 Hz, 1H), 5.39 (d, *J* = 12.1 Hz, 1H), 1.34 (s, 9H).
^{13}C-NMR (100 MHz, $CDCl_3$):
(*E*)-3-(4-(*Tert*-butyl)phenyl)acrylonitrile: δ / ppm = 155.1, 150.6, 131.0, 127.4 (2C), 126.2 (2C), 118.6, 95.4, 35.1, 31.2 (3C).
(*Z*)-3-(4-(*Tert*-butyl)phenyl)acrylonitrile: δ / ppm = 154.8, 148.7, 131.1, 129.1 (2C), 126.0 (2C), 117.1, 94.1, 35.1, 31.2 (3C).
IR (Diamond-ATR, neat): $\tilde{\nu}$ / cm^{-1} = 3055, 2962, 2904, 2868, 2214, 1618, 1605, 1562, 1506, 1475, 1463, 1412, 1395, 1364, 1324, 1295, 1271, 1230, 1217, 1202, 1190, 1121, 1107, 1016, 968, 948, 924, 859, 842, 808, 763, 734, 719, 696.
MS (EI, 70 eV): *m/z* (%) = 185 (15), 171 (13), 170 (100), 155 (44), 154 (13), 142 (24), 115 (12).
HRMS (EI): *m/z* calc. for [$C_{13}H_{15}N$]: 185.1204; found: 185.1199.

3-(4-(Trifluoromethyl)phenyl)acrylonitrile (5e)

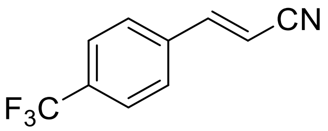

According to the typical procedure for the preparation of arylacrylonitriles, 1-iodo-4-(trifluoromethyl)benzene (4.08 g, 15.0 mmol, 1.0 equiv) in DMF (30 mL) was added to a mixture of acrylonitrile (1.19 g, 22.5 mmol, 1.5 equiv) and NaOAc (1.48 g, 18.0 mmol, 1.2 equiv) in DMF (15 mL), 10 weight-% Pd/C (159 mg. 0.15 mmol, 0.01 equiv) was added and the mixture was stirred over night at 140 °C. The crude product was purified by column chromatography (isohexane:EtOAc = 19:1) to give the titel compound as colorless solid (2.31 g, 118 mmol, 78% yield; *E*/*Z* = 78/22).

^{1}H-NMR (400 MHz, $CDCl_3$):
(*E*)- 3-(4-(Trifluoromethyl)phenyl)acrylonitrile: δ / ppm = 7.68 (d, *J* = 8.3 Hz, 2H), 7.57 (d, *J* = 8.2 Hz, 2H), 7.44 (d, *J* = 16.7 Hz, 1H), 5.99 (d, *J* = 16.7 Hz, 1H).
(*Z*)- 3-(4-(Trifluoromethyl)phenyl)acrylonitrile: δ / ppm = 7.91 (d, *J* = 8.2 Hz, 2H), 7.71 (d, *J* = 8.4 Hz, 2H), 7.19 (d, *J* = 12.1 Hz, 1H), 5.61 (d, *J* = 12.1 Hz, 1H).
^{13}C-NMR (100 MHz, $CDCl_3$):
(*E*)- 3-(4-(Trifluoromethyl)phenyl)acrylonitrile: δ / ppm = 149.0, 136.8, 132.9 (q, *J* = 32.6 Hz), 127.7 (2C), 126.3 (q, *J* = 3.7 Hz, 2C), 123.7 (d, *J* = 272.4 Hz), 117.5, 99.4.
(*Z*)- 3-(4-(Trifluoromethyl)phenyl)acrylonitrile: δ / ppm = 147.2, 136.8, 132.9 (q, *J* = 32.6 Hz), 129.3 (2C), 126.1 (q, *J* = 3.9 Hz, 2C), 123.7 (d, *J* = 272.2 Hz), 116.8, 98.1.
IR (Diamond-ATR, neat): $\tilde{\nu}$ / cm^{-1} = 3055, 3029, 2219, 1622, 1579, 1414, 1320, 1274, 1213, 1194, 1165, 1153, 1107, 1065, 1034, 1015, 978, 970, 954, 859, 851, 837, 812, 760, 733, 657.
MS (EI, 70 eV): *m/z* (%) = 198 (10), 197 (100), 196 (16), 178 (25), 176 (17), 170 (15), 169 (12), 147 (47), 128 (24).
HRMS (EI): *m/z* calc. for [$C_{10}H_6NF_3$]: 197.0452; found: 197.0447.

Phenyl(styryl)sulfane (5h)

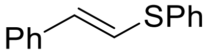

Phenylacetylene (1.02 g, 10.0 mmol, 1.0 equiv) and thiophenol (1.2 g, 10.0 mmol, 1.0 equiv) were solved in DMF (5 mL). CuI (0.06 g, 0.30 mmol, 0.03 equiv) was added the mixture was stirred for 2 d. EtOAc (40 mL) was added to the reaction mixture. The mixture was washed with an aqueous LiCl-solution (10%, 3 x 50 mL). The organic layer was dried over $MgSO_4$, solvents were removed *in vacuo* to give the titel compound as orange oil (2.04 g, 9.6 mmol, 96% yield; *E*/*Z* = 71/29).[5]

^{1}H-NMR (400 MHz, $CDCl_3$):
(*E*)-Phenyl(styryl)sulfane: δ / ppm = 7.54 (d, *J* = 7.4 Hz, 2H), 7.50 – 7.44 (m, 2H) 7.43– 7.21 (m, 6H), 6.60 (d, *J* = 10.8 Hz, 1H), 6.51 (d, *J* = 10.7 Hz, 1H).
(*Z*)-Phenyl(styryl)sulfane: δ / ppm = 7.57 – 7.21 (m, 10H), 6.89 (d, *J* = 15.5 Hz, 1H), 6.74 (d, *J* = 15.5 Hz, 1H).
^{13}C-NMR (100 MHz, $CDCl_3$):
(*E*)-Phenyl(styryl)sulfane: δ / ppm = 136.6, 136.4, 130.2 (2C), 129.3 (2C), 128.9 (2C), 128.5 (2C), 127.4, 127.4, 127.3, 126.2.
(*Z*)-Phenyl(styryl)sulfane: δ / ppm = 136.6, 135.4, 132.0, 130.0 (2C), 129.3 (2C), 128.8 (2C), 127.7 (2C), 127.1, 126.2, 123.5.
IR (Diamond-ATR, neat): $\tilde{\nu}$ / cm^{-1} = 3072, 3055, 3019, 1597, 1582, 1573, 1569, 1490, 1476, 1439, 1354, 1331, 1156, 1087, 1069, 1024, 999, 944, 908, 858, 846, 772, 738, 729, 700, 686.
MS (EI, 70 eV): *m/z* (%) = 213 (14), 212 (100), 211 (61), 179 (28), 178 (61), 167 (26), 165 (13), 152 (11), 135 (11), 134 (12), 121 (25).
HRMS (EI): *m/z* calc. for [$C_{14}H_{12}S$]: 212.0660; found: 212.0655.

[5] I. P. Beletskaya, I. G. Trostyanskaya, *Synlett*, **2012**, *4*, 535.

3-Methylhept-2-enenitrile (8b)

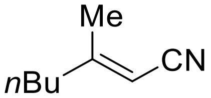

KOH (0.56 g, 10.0 mmol, 1.0 equiv) was added in a 100 mL three-necked Schlenk flask, MeCN (20 mL) was added and the mixture was heated to reflux. A solution of hexan-2-one (1.0 g, 10.0 mmol, 1.0 equiv) in MeCN (20 mL) was added dropwise over a period of 20 min. The mixture was stirred at reflux over night. The mixture was poured on ice water (50 mL), and the aqueous layer was extracted with DCM (3 x 50 mL). The combined organic layers were dried over $MgSO_4$ and solvents were removed *in vacuo* (200 mbar). The crude product was purified by column chromatography (isohexane:EtOAc = 49:1) to give the titel compound as a colorless oil (0.34 g, 2.7 mmol, 27% yield; *E*/*Z* = 65/35).[6]

^{1}H-NMR (400 MHz, $CDCl_3$):

(*E*)-3-Methylhept-2-enenitrile: δ / ppm = 5.10 (s, 1H), 2.18 (t, *J* = 7.6 Hz, 2H), 2.04 (s, 3H), 1.53 – 1.41 (m, 2H), 1.39 – 1.27 (m, 2H), 0.91 (t, *J* = 7.3 Hz, 3H).

(*Z*)-3-Methylhept-2-enenitrile: δ / ppm = 5.10 (s, 1H), 2.44 – 2.37 (t, *J* = 7.6 Hz, 2H), 1.90 (d, *J* = 1.4 Hz, 3H), 1.53 – 1.41 (m, 2H), 1.39 – 1.27 (m, 2H), 0.94 (t, *J* = 7.3 Hz, 3H).

^{13}C-NMR (100 MHz, $CDCl_3$):

(*E*)-3-Methylhept-2-enenitrile: δ / ppm = 165.8, 117.5, 95.1, 38.5, 29.3, 22.3, 21.1, 13.9.

(*Z*)-3-Methylhept-2-enenitrile: δ / ppm = 165.9, 117.3, 95.6, 36.2, 29.8, 23.0, 22.4, 14.0.

The spectra matched with those reported in the literature.[7]

---

[6] S. A. DiBiase, B. A. Lipisko, A. Haag, A. W. Raymond, G. W. Gokel, *J. Org. Chem.* **1979**, *44*, 4640.

[7] T. T. Vasilev, N. A. Kuzmina, O. V. Chakovskaya, N. E. Mysova, A. B. Terentev, *Russ. J. Org. Chem.* **2004**, *40*, 174.

3-Oxohexanenitrile

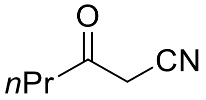

According to the literature[8], to a solution of ethyl butyrate (23.2 g, 26.4 mL, 200 mmol, 1.0 equiv) and acetonitrile (12.3 g, 15.6 mL, 300 mmol, 1.5 equiv) in THF (400 mL) was added NaH (60% dispersion in mineral oil, 12.0 g, 300 mmol, 1.5 equiv) in small portions. The mixture was heated to reflux overnight. HCl was added until pH=7. The aqueous phase was extracted three times with Et_2O (3×100 mL) and the combined organic phases were dried over anhydrous $MgSO_4$ and filtrated. After removal of the solvent *in vacuo*, flash chromatographical purification (silica gel, isohexane:Et_2O = 1:1) afforded the title compound as an orange oil (11.7 g, 106 mmol, 52% yield).

^{1}H-NMR (400 MHz, $CDCl_3$): δ / ppm = 3.44 (s, 2H), 2.60 (t, *J*=7.2, 2H), 1.67 (p, *J*=7.4, 2H), 0.95 (t, *J*=7.4, 3H).
The spectra matched with those reported in the literature[9].

(*Z*)-1-Cyanopent-1-en-2-yl 4-methylbenzenesulfonate

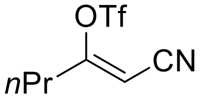

According to the literature[10], Et_3N (11.1 g, 110 mmol, 1.1 equiv) was added carefully to a solution of 3-oxohexanenitrile (11.1 g, 100 mmol, 1.0 equiv) in DCM (500 mL) at −78 °C. After stirring for 5 min triflic anhydride (18.5 mL, 110 mmol, 1.1 equiv) was added at the same temperature. The mixture was stirred for 30 min at −78 °C before it was allowed to warm to 25 °C and stirred overnight. Water (100 mL) was added. The aqueous phase was extracted three times with Et_2O (3×100 mL) and the combined organic phases were dried over anhydrous $MgSO_4$ and filtrated. After removal of the solvent *in vacuo*, flash chromatographical purification (silica gel, isohexane:EtOAc = 95:5) afforded the title compound as a yellow oil (18.5 g, 76 mmol, 76% yield, *Z/E* > 99/1).

^{1}H-NMR (400 MHz, $CDCl_3$): δ / ppm = 5.30 (t, *J*=1.2, 1H), 2.54 (td, *J*=7.5, 1.2, 2H), 1.66 (h, *J*=7.4, 2H), 1.01 (t, *J*=7.4, 3H).
^{13}C-NMR (100 MHz, $CDCl_3$): δ / ppm = 166.4, 118.4 (q, *J*=320.5), 111.9, 92.0, 36.4, 19.5, 13.2.
IR (Diamond-ATR, neat): $\tilde{\nu}$ / cm^{-1} = 2974, 2236, 1664, 1423, 1209, 1134, 1103, 1030, 914, 893, 862, 817, 780, 730, 687, 658.
MS (EI, 70 eV): *m/z* (%) = 151 (51), 136 (100), 109 (11), 69 (42), 65 (25).
HRMS (EI): *m/z* calc. for [$C_7H_8F_3NO_3S$]: 243.0177; found: 243.0171.

---

[8]S. Havel, P. Khirsariya, N. Akavaram, K. Paruch, B. Carbain *J. Org. Chem.* **2018**, *83*, 15380.
[9] Y. Chen, S. McN. Sieburth, *Synthesis*, **2002**, *15*, 2191.
[10] Z. Fang, Y. Song, T. Sarkar, E. Hamel, W. E. Fogler, G. E. Agoston, P. E. Fanwick, M. Cushman, *J. Org. Chem.* **2008**, *73*, 4241.

(*E*)-3-Methylhex-2-enenitrile (8c)

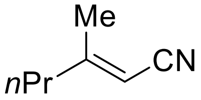

According to the literature[11], to a stirred solution of CuCN (2.51 g, 28.0 mmol, 1.4 equiv) in Et_2O (20 mL) was added MeLi (1.0 M, 28.0 mL, 28 mmol, 1.4 equiv) at −78 °C. The mixture was stirred for 2 h at −78 °C. (*Z*)-1-cyanopent-1-en-2-yl (4.86 g, 20.0 mmol, 1.0 equiv) was added dropwise and the mixture was stirred for 4 h hours at −78 °C, before it was quenched with *sat. aq.* NH_4Cl solution (30 mL). The aqueous phase was extracted three times with Et_2O (3×100 mL) and the combined organic phases were dried over anhydrous $MgSO_4$ and filtrated. After removal of the solvent *in vacuo* (100 mbar), flash chromatographical purification (silica gel, isohexane) afforded the title compound as a yellow oil (1.97 g, 18.0 mmol, 90% yield, *E/Z* > 99/1).

^{1}H-NMR (400 MHz, $CDCl_3$): δ / ppm = 5.10 (d, *J*=1.2, 1H), 2.17 (dtd, *J*=15.3, 7.6, 1.5, 2H), 2.03 (d, *J*=1.1, 2H), 1.56 – 1.43 (m, 2H), 0.91 (t, *J*=7.4, 3H).

^{13}C-NMR (100 MHz, $CDCl_3$): δ / ppm = 165.4, 117.4, 95.2, 40.7, 21.0, 20.4, 13.6.

IR (Diamond-ATR, neat): $\tilde{\nu}$ / cm^{-1} = 2964, 2936, 2876, 2218, 1632, 1458, 1443, 1420, 1384, 968, 835, 798.

MS (EI, 70 eV): *m/z* (%) = 110 (18), 94 (16), 81 (76), 80 (71), 68 (100), 67 (14), 56 (10), 41 (20).

HRMS (EI): *m/z* calc. for [$C_7H_{11}N$]: 109.0891; found: 110.0965 [M + H].

[11] A. Jolit, P. M. Walleser, G. P. A. Yap, M. A. Tius, *Angew. Chem. Int. Ed.*, **2014**, *53*, 6180; *Angew. Chem.* **2014**, *126*, 6294.

4-Methoxy-3-methylbut-2-enenitrile (8d)

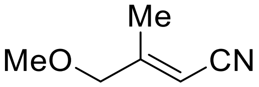

NaH (1.8 g, 45.0 mmol, 60% in mineral oil) was added to a three-necked round-bottom flask. THF was added to the flask and the suspension was cooled to 0 °C. Diethyl cyanomethyl-phosphonate (7.8 mL, 48 mmol, 1.6 equiv) was added dropwise at 0 °C. After the addition was complete (reaction mixture turned colorless), the mixture was stirred at 0 °C for 1 h. 1-Methoxypentan-2-one (2.64 g, 30.0 mmol, 1.0 equiv) in THF (100 mL) was added dropwise and the reaction mixture was heated to reflux for 3 h. The reaction mixture was cooled to 25 °C and was poured into a separatory funnel containing *sat. aq.* NH_4Cl (150 mL). The aqueous layer was extracted with Et_2O (3x150 mL). The combined organic layers were washed with brine (100 mL), dried over $MgSO_4$, filtered and concentrated. The crude product was purified by column chromatography (pentane:Et_2O = 100:0 → 95:5) to give the titel compound as a colorless oil (2.63 g, 23.7 mmol, 79% yield; *E*/*Z* = 80/20).[12]

^{1}H-NMR (400 MHz, $CDCl_3$):
(*E*)-4-Methoxy-3-methylbut-2-enenitrile: δ / ppm = 5.46 (tt, *J*=2.2, 1.1, 1H), 3.93 (dd, *J*=1.9, 0.9, 2H), 3.38 (s, 3H), 2.00 (d, *J*=1.2, 3H).
(*Z*)-4-Methoxy-3-methylbut-2-enenitrile: δ / ppm = 5.27 (h, *J*=1.4, 1H), 4.18 (s, 2H), 3.36 (s, 3H), 1.96 (d, *J*=1.6, 3H).
^{13}C-NMR (100 MHz, $CDCl_3$):
(*E*)-4-Methoxy-3-methylbut-2-enenitrile: δ / ppm = 160.4, 117.0, 94.8, 74.8, 59.0, 17.9.
(*Z*)-4-Methoxy-3-methylbut-2-enenitrile: δ / ppm = 161.3, 116.0, 97.1, 73.2, 58.6, 20.6.
IR (Diamond-ATR, neat): $\tilde{\nu}$ / cm^{-1} = 2933, 2828, 2220, 1730, 1641, 1469, 1452, 1438, 1383, 1265, 1200, 1114, 1074, 990, 955, 920, 808.
MS (EI, 70 eV): *m/z* (%) = 111 (11), 96 (100), 81 (19), 71 (28), 68 (26), 55 (16).
HRMS (EI): *m/z* calc. for [C_6H_9NO]: 111.0684; found: 111.0679.

[12] P. J. Gilligan, B. K. Folmer, R. A. Hartz, S. Koch, K. K. Nanda, S. Andreuski, L. Fitzgerald, K. Miller, W. J. Marshall, *Bioorg. Med. Chem.*, **2003**, *11*, 4093.

(*4E*)-nona-2,4-dienenitrile (8e)

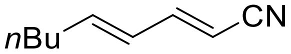

NaH (0.48 g, 12.0 mmol, 1.2 equiv, 60% in mineral oil) was dissolved in THF (10 mL) and cooled to 0 °C. Diethyl(cyanomethyl)phosphonate (2.13 g, 12.0 mmol, 1.2 equiv) was added dropwise. The resulting solution was allowed to warm to 25 °C and stirred for 30 min. After cooling to 0 °C, (*E*)-hept-2-enal (1.12 g, 10.0 mmol, 1.0 equiv) in THF (4 mL) was added dropwise. The resulting solution was stirred for 1.5 h at 25 °C. After quenching with NH_4Cl, the aqueous layer was extracted with Et_2O. The combind organic layers were dried over $MgSO_4$, solvents were removed in vacuo (200 mbar). The crude product was purified by column chromatography (pentane:Et_2O = 100:0 → 95:5) to give the titel compound as a colorless oil (1.08 g, 8.0 mmol, 80% yield, *E*/*Z* = 69/31).[13]

^{1}H-NMR (400 MHz, $CDCl_3$):

(*2E,4E*)-nona-2,4-dienenitrile: δ / ppm = 7.04 – 6.91 (m, 1H), 6.17 – 6.09 (m, 2H), 5.23 (d, *J*=15.9, 1H), 2.26 – 2.10 (m, 2H), 1.51 – 1.19 (m, 4H), 0.91 (td, *J*=7.2, 3.9, 3H).

(*2Z,4E*)-nona-2,4-dienenitrile: δ / ppm = 6.78 (t, *J*=10.9, 1H), 6.63 – 6.50 (m, 1H), 6.25 – 6.15 (m, 1H), 5.09 (d, *J*=10.7, 1H), 2.19 (dddd, *J*=12.7, 9.7, 6.4, 1.9, 2H), 1.52 – 1.20 (m, 4H), 0.91 (td, *J*=7.2, 3.9, 3H).

^{13}C-NMR (100 MHz, $CDCl_3$):

(*2E,4E*)-nona-2,4-dienenitrile: δ / ppm = 151.1, 146.2, 128.1, 118.6, 96.5, 32.7, 30.7, 22.4, 14.0.

(*2Z,4E*)-nona-2,4-dienenitrile: δ / ppm = 150.0, 146.5, 127.0, 116.8, 94.8, 32.8, 30.8, 22.4, 14.0.

The spectra matched with those reported in the literature.[14]

---

[13] J. K. Gawronski, H. M. Walborsky, *J. Org. Chem.* **1986**, *51*, 2863.
[14] C. H. Yoon, K. S. Yoo, S. W. Yi, R. K. Mishra, K. W. Jung, *Org. Lett.* **2004**, *6*, 4037.

***(E)-Tert*-butyl cinnamate (11b)**

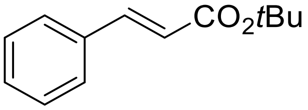

According to literature, to a solution of cinnamoyl chloride (3.32 g, 20.0 mmol, 1.0 equiv) in THF (20 mL) KOtBu solution (24 mL, 24.0 mmol, 1.0 M in THF) was added over 10 min at 0 °C. After stirring for 30 min, the reaction mixture was quenched by adding sat. aq. NH_4Cl solution. The aqueous layer was extracted with Et_2O (3x20 mL). The combined organic layers were washed with brine, dried over $MgSO_4$ and filtered. After evaporation of the solvent, the crude product was purified by column chromatography (isohexane) to give the titel compound as a colorless oil (3.27 g, 16.0 mmol, 80% yield, *E/Z* > 99/1).

^{1}H-NMR (400 MHz, $CDCl_3$): δ / ppm = 7.59 (d, *J*=16.0, 1H), 7.51 (dq, *J*=5.6, 3.1, 2H), 7.37 (dd, *J*=5.0, 2.0, 3H), 6.37 (d, *J*=16.0, 1H), 1.54 (s, 9H).

^{13}C-NMR (100 MHz, $CDCl_3$): δ / ppm = 166.5, 143.7, 134.8, 130.1, 129.0 (2C), 128.1 (2C), 120.3, 80.7, 28.3 (3C).

The spectra matched with those reported in the literature.[15]

[15] T. Onozawa, M. Kitajima, N. Kogure, H. Takayama, *J. Org. Chem.* **2018**, *83*, 15312.

NMR data

(*Z*)-2-((4-Bromophenyl)(hydroxy)methyl)-3-phenylacrylonitrile (4aa)

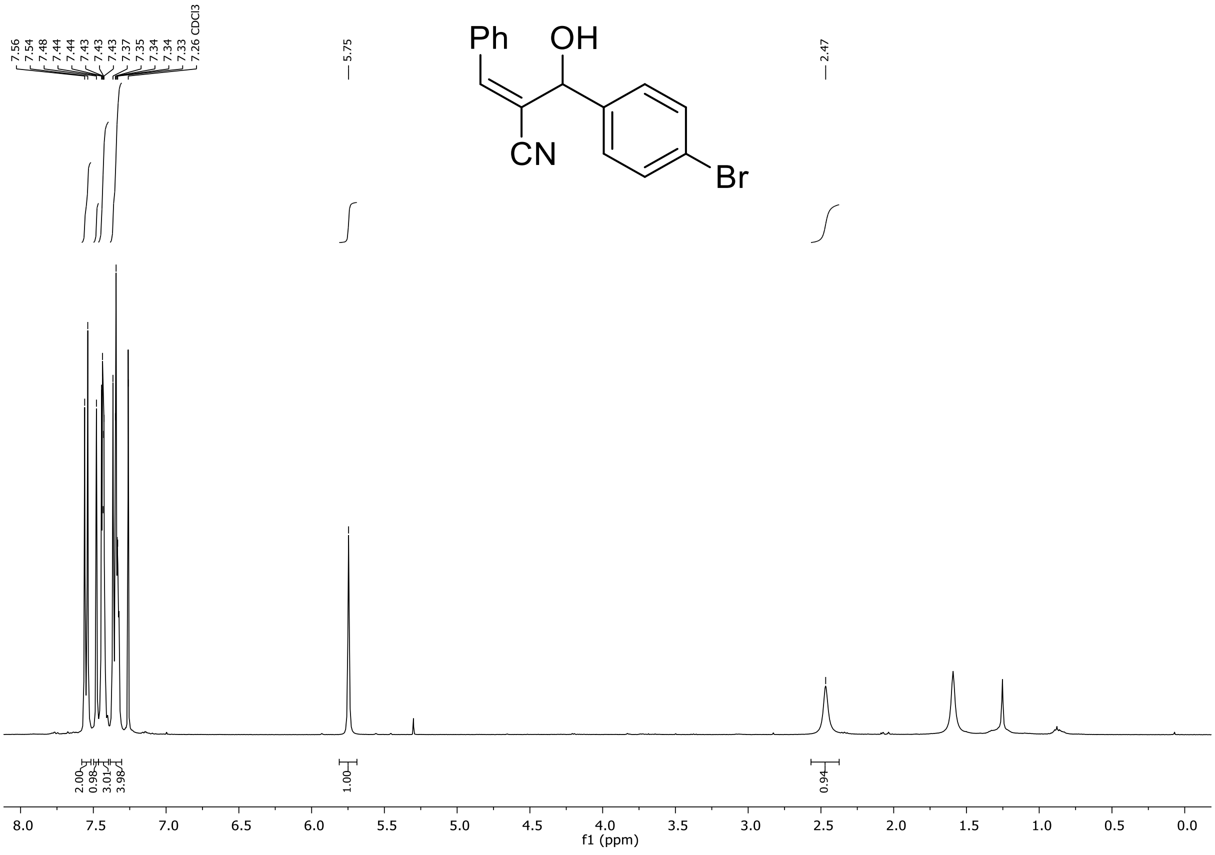

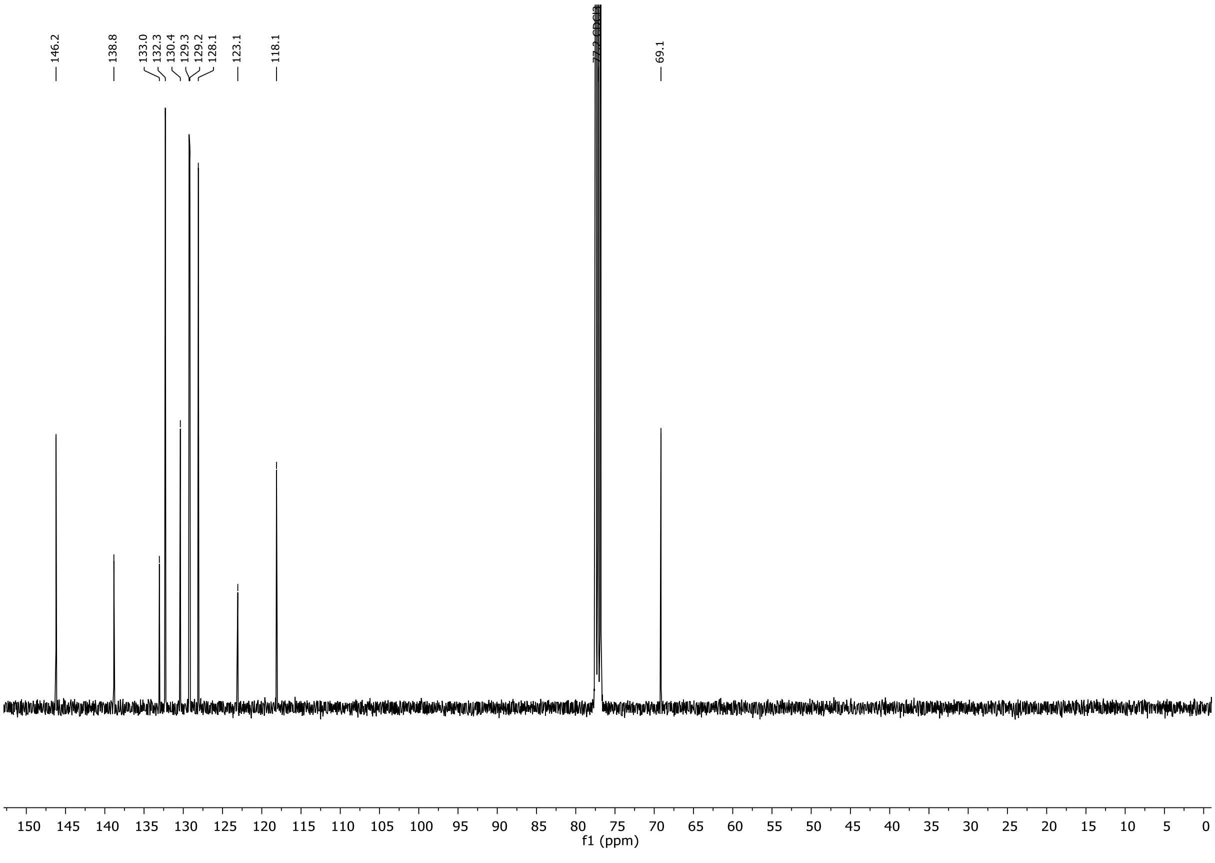

(*Z*)-2-((4-Chlorophenyl)(hydroxy)methyl)-3-phenylacrylonitrile (4ab)

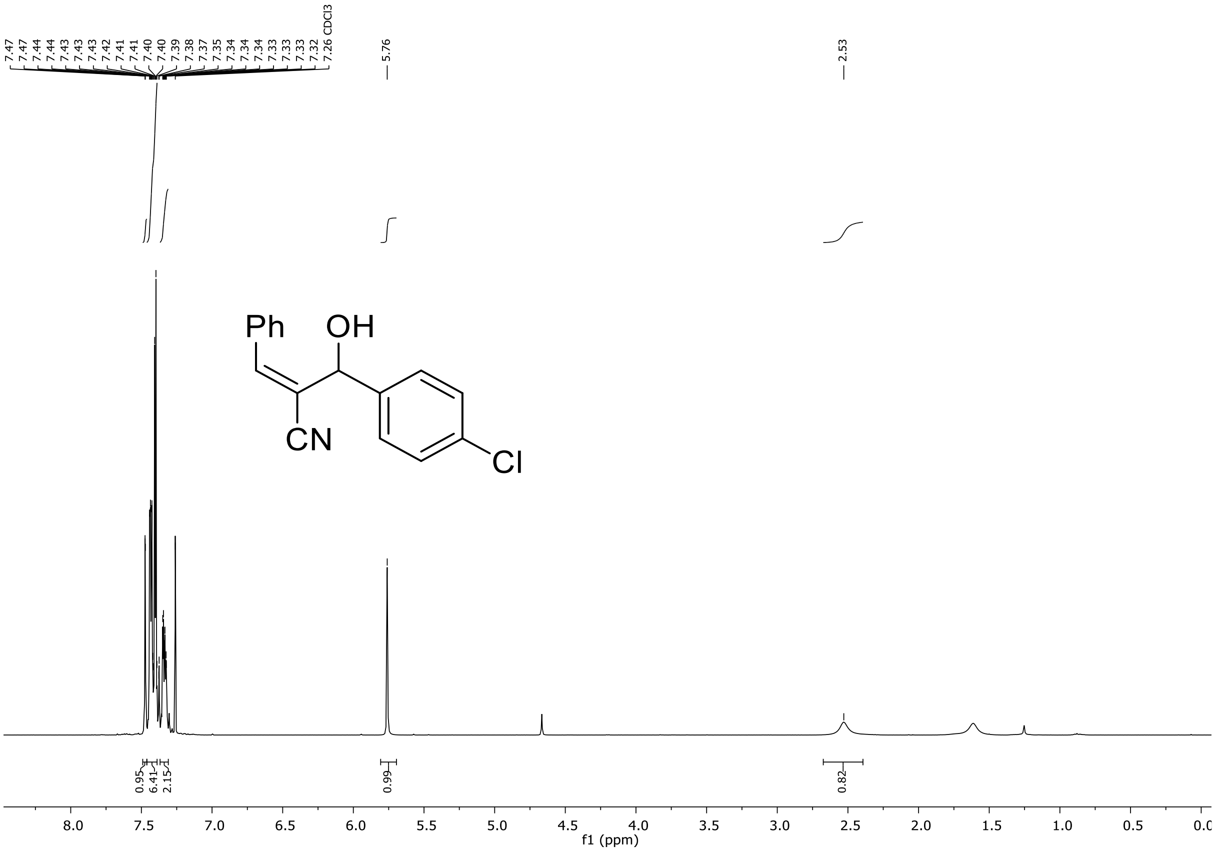

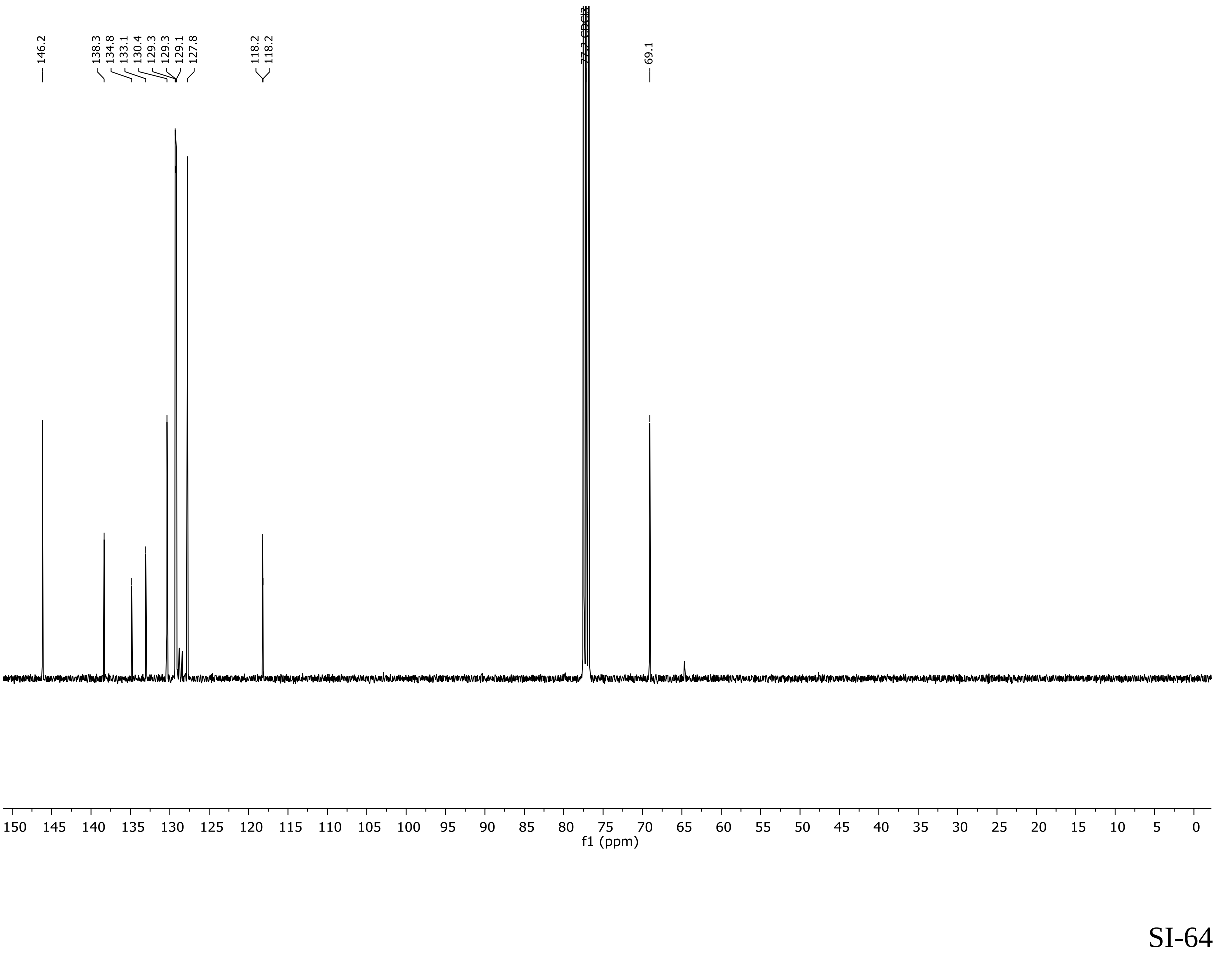

2-((2,6-Dichlorophenyl)(hydroxy)methyl)-3-phenylacrylonitrile (4ac)

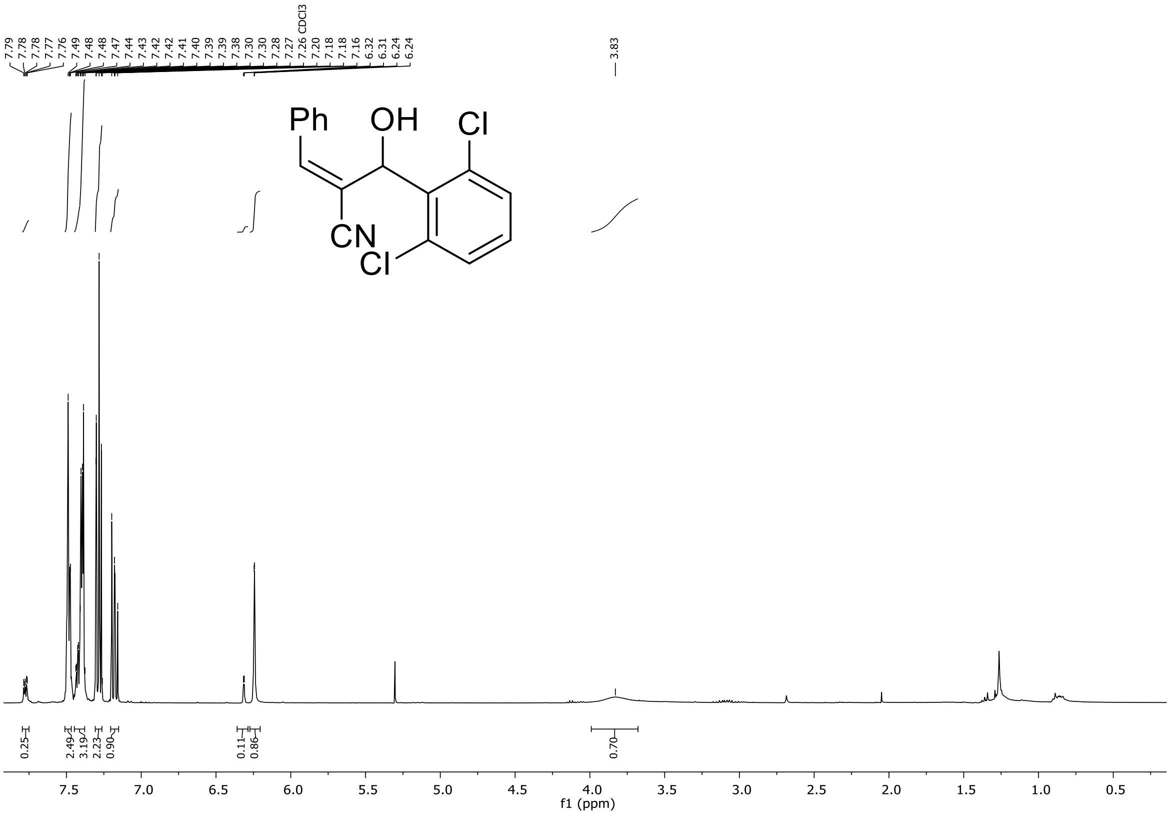

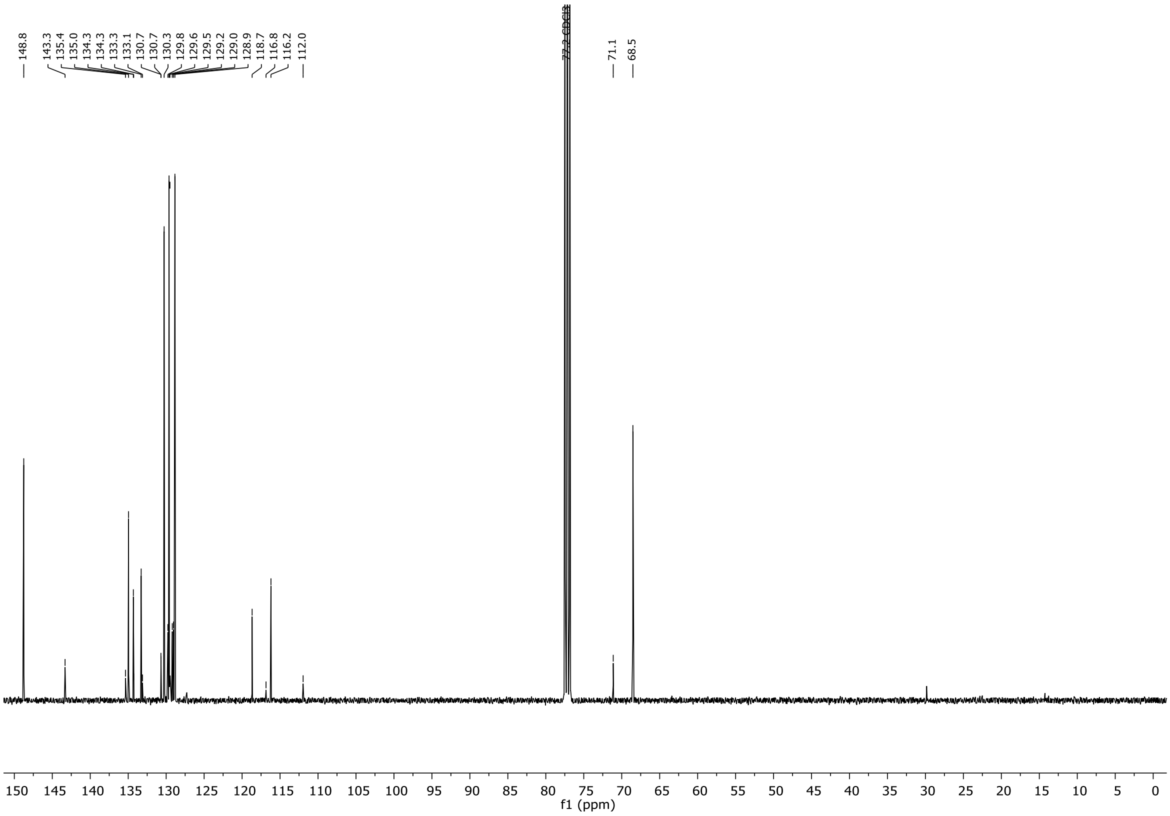

(*Z*)-4-Hydroxy-3-phenyl-4-(*p*-tolyl)but-2-enenitrile (4ad)

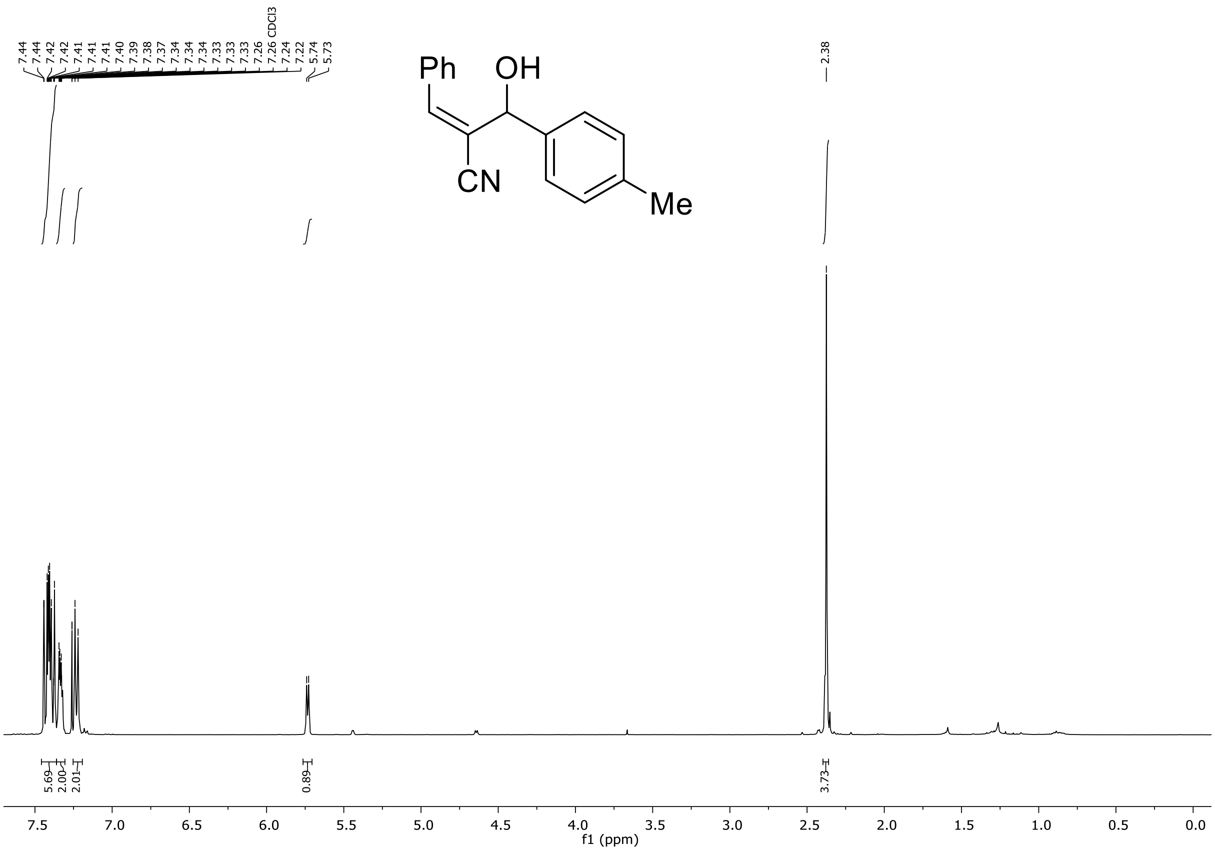

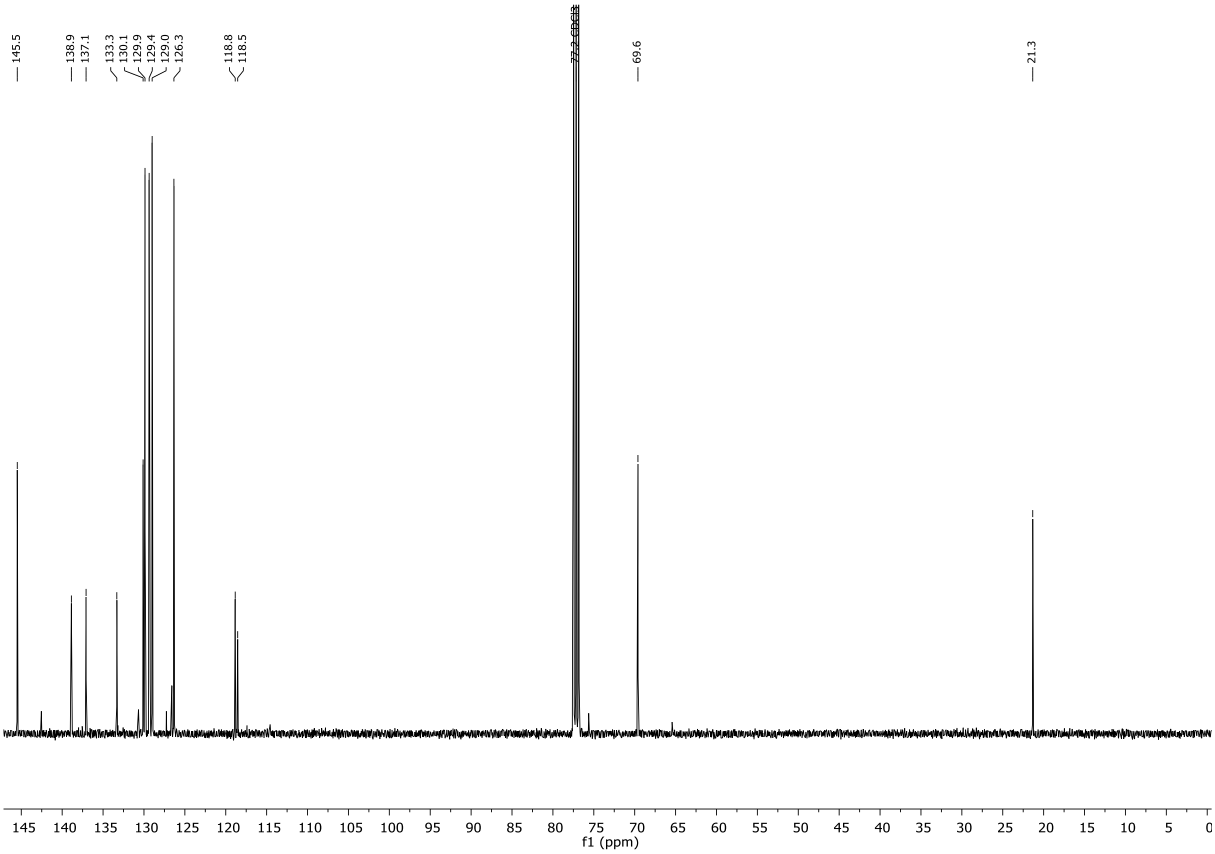

2-(Cyclohex-2-en-1-yl)-3-phenylacrylonitrile (4ae)

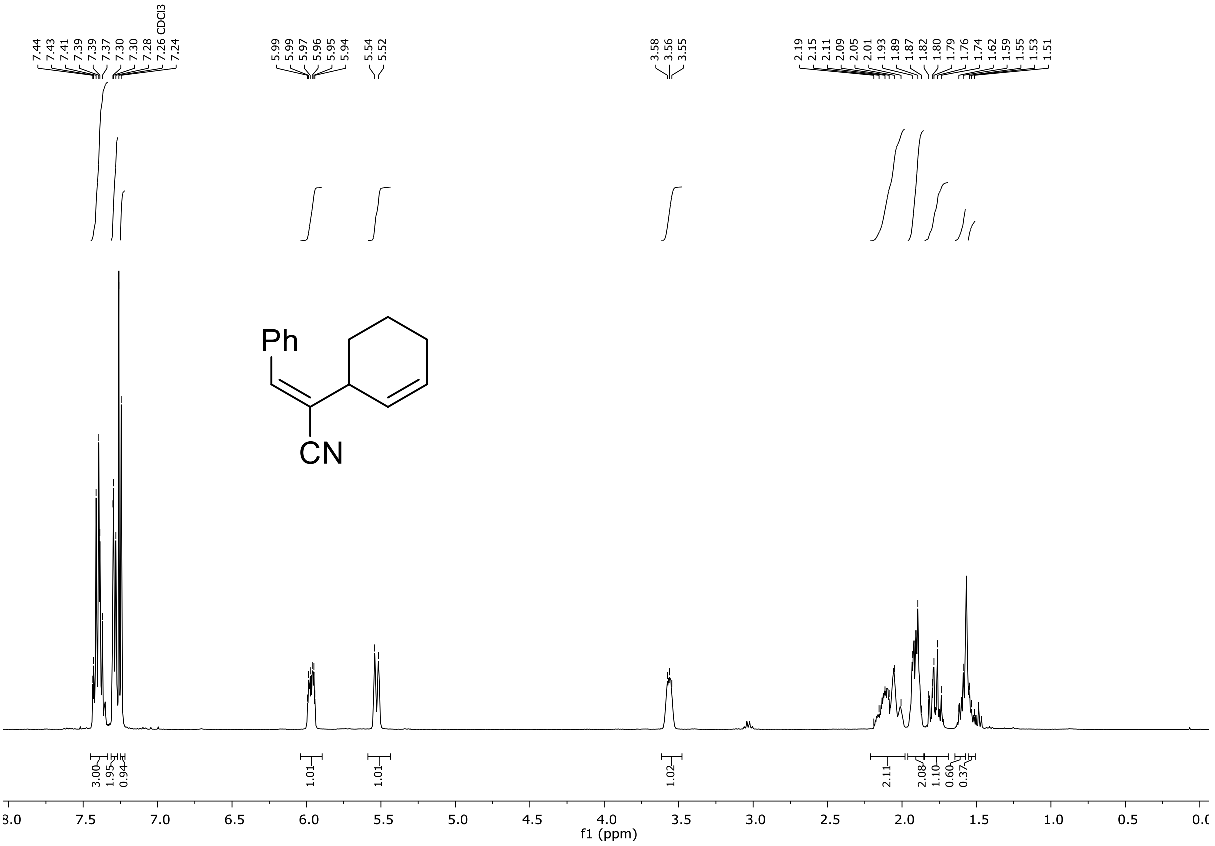

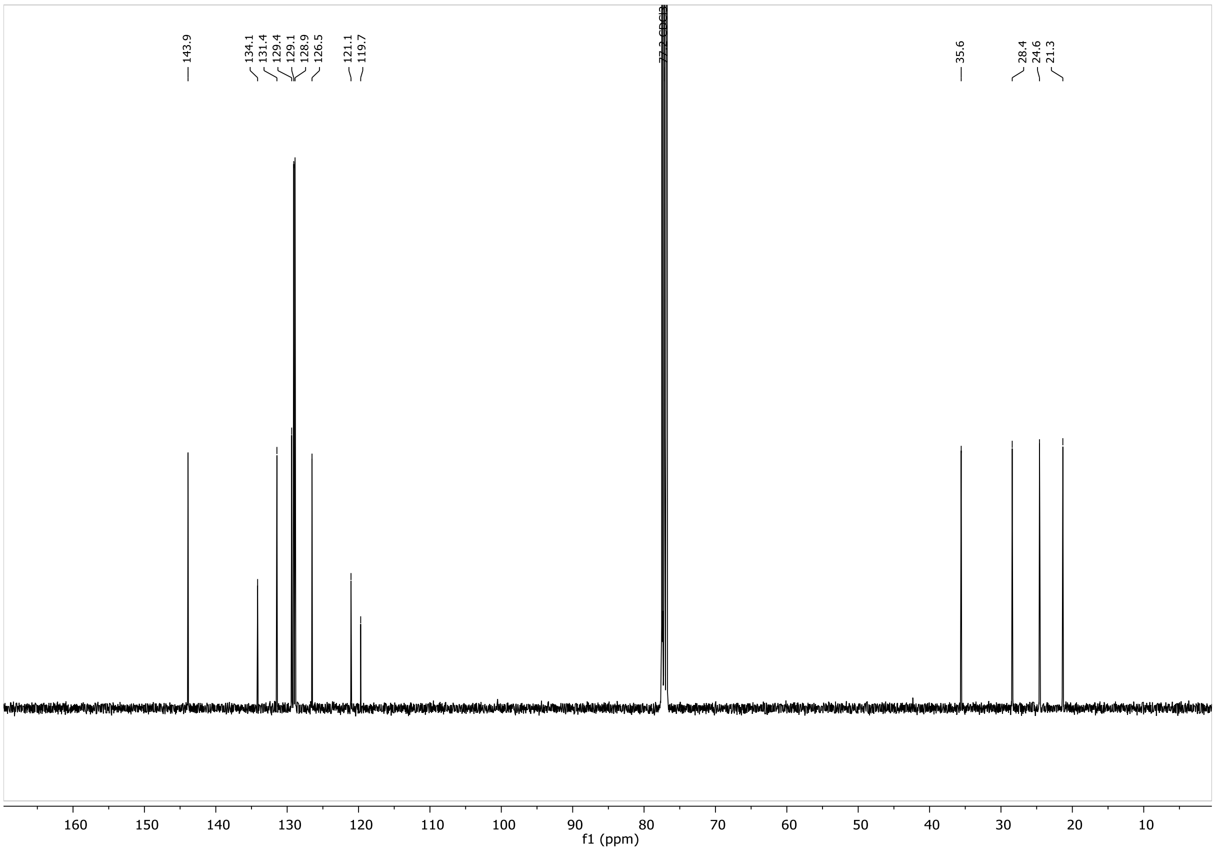

2-(Butylthio)-3-phenylacrylonitrile (4af)

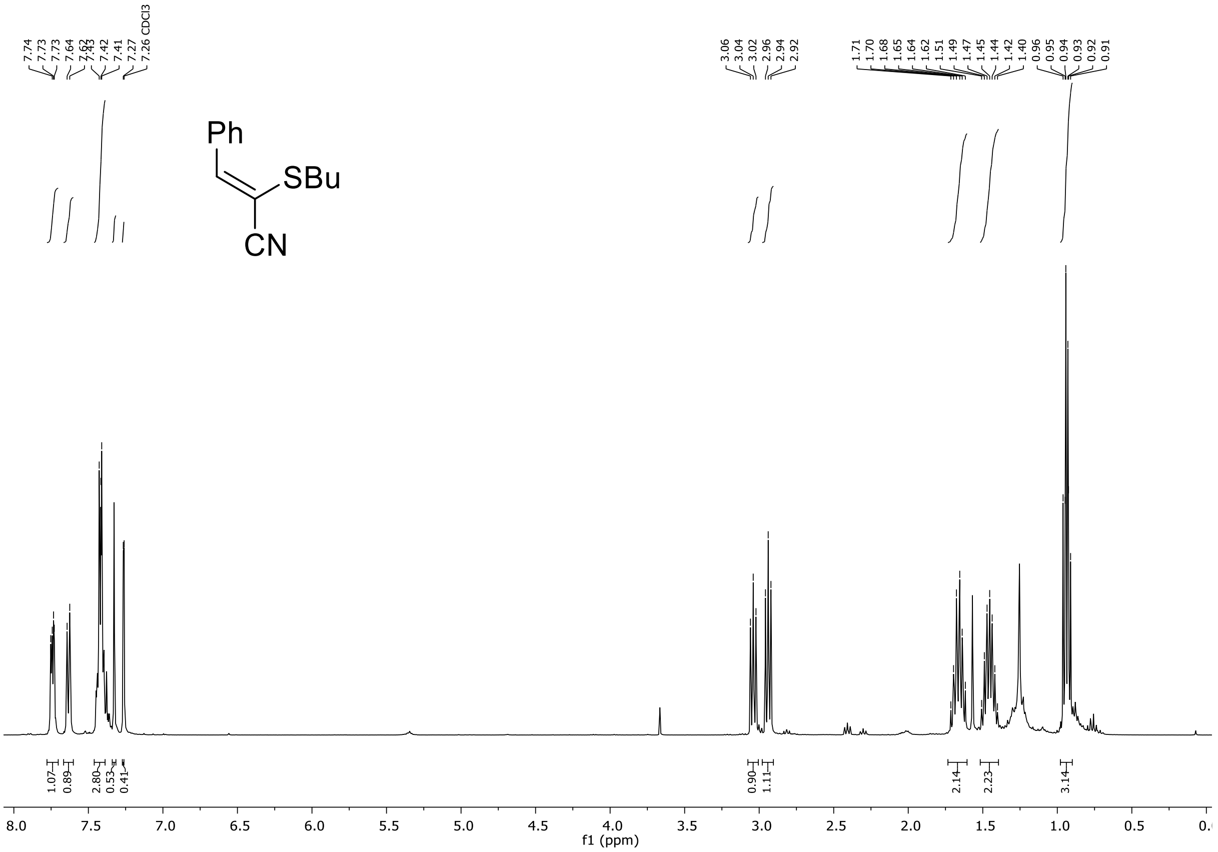

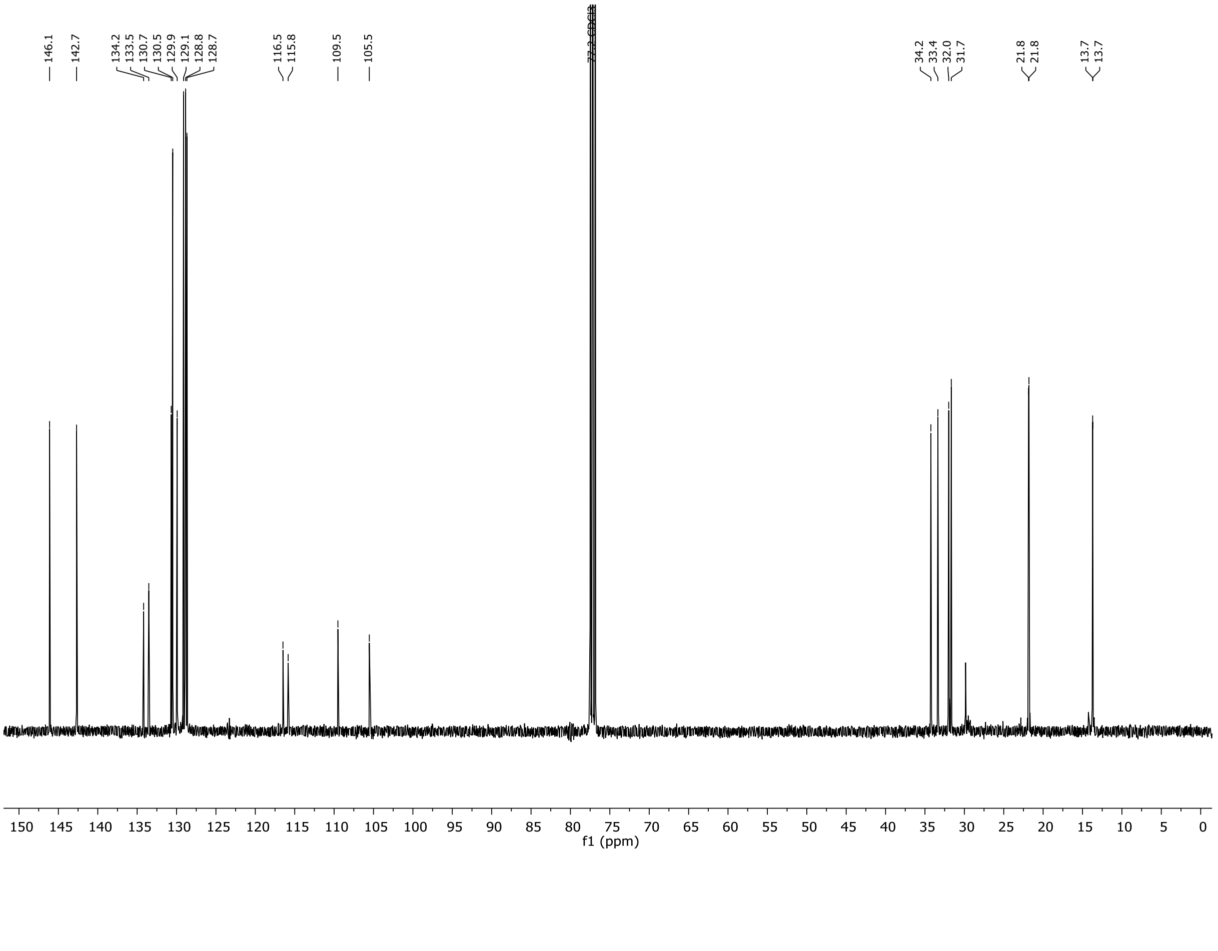

(*E*)-2-(Hydroxydiphenylmethyl)-3-phenylacrylonitrile (4ag)

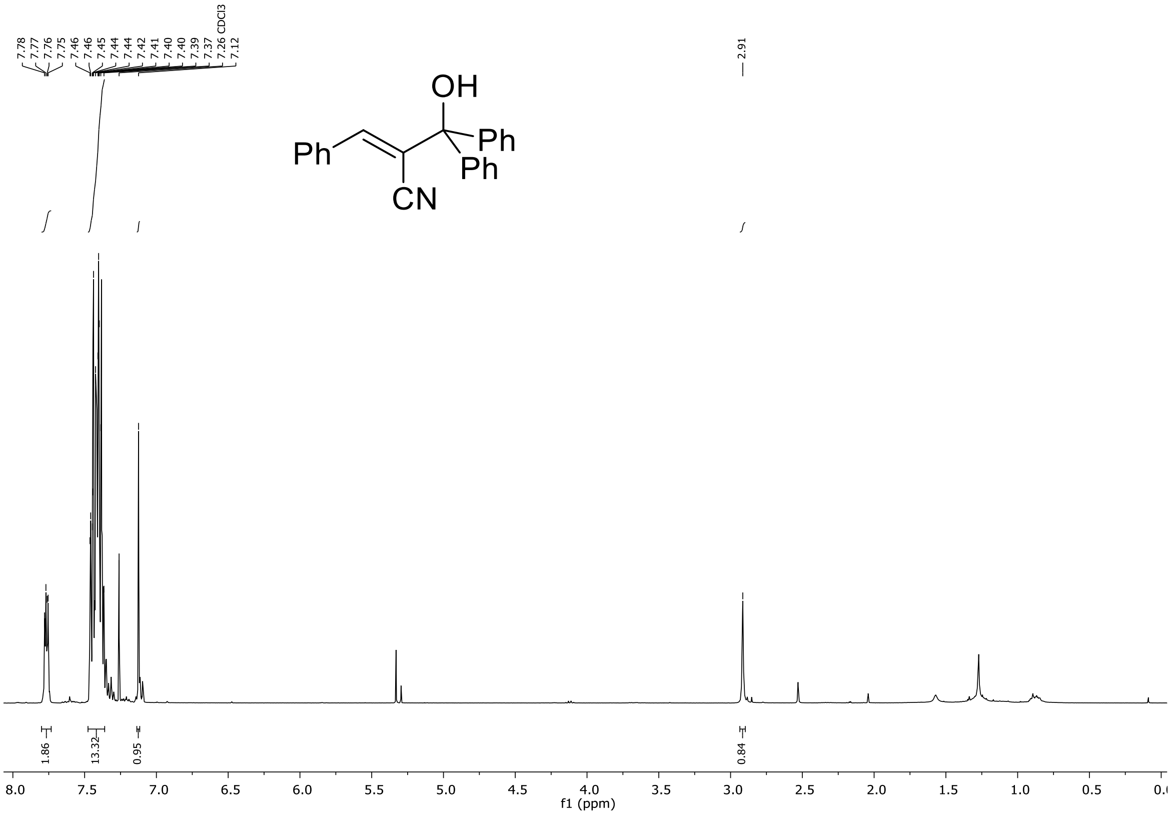

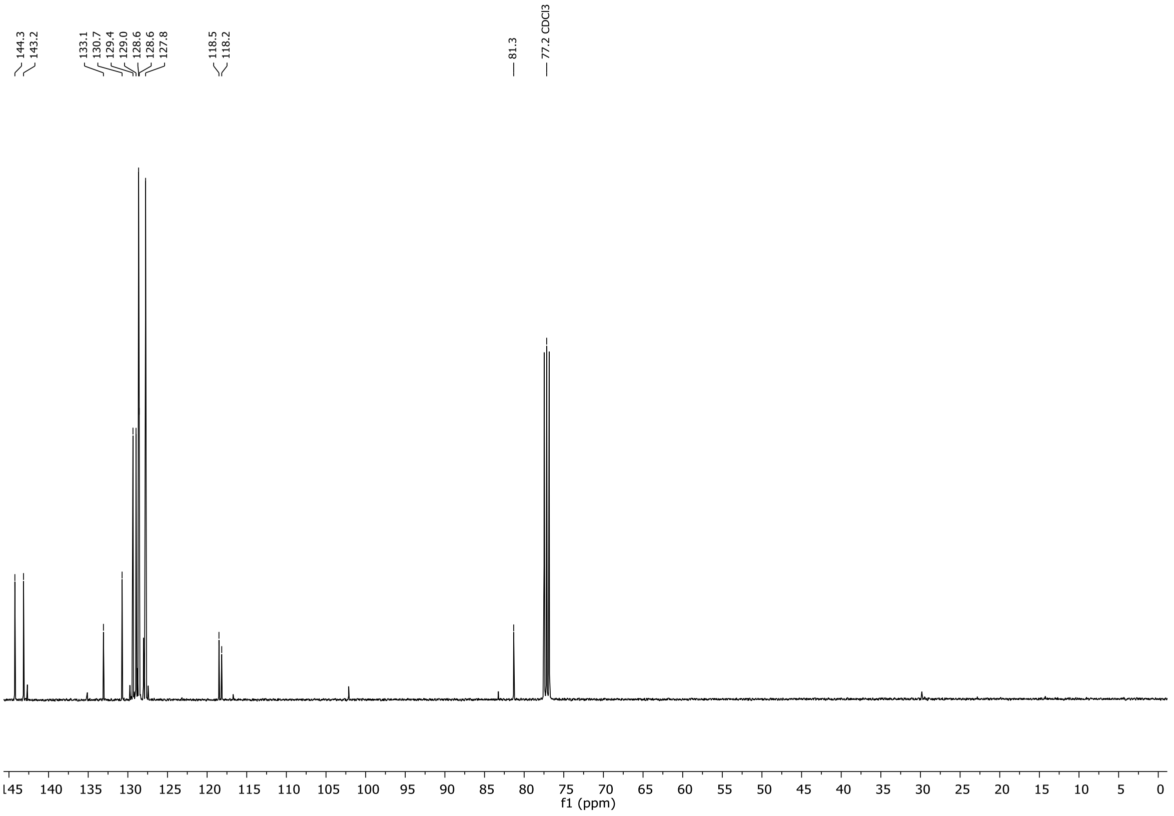

2-(2-Hydroxybicyclo[2.2.1]heptan-2-yl)-3-phenylacrylonitrile (4ah)

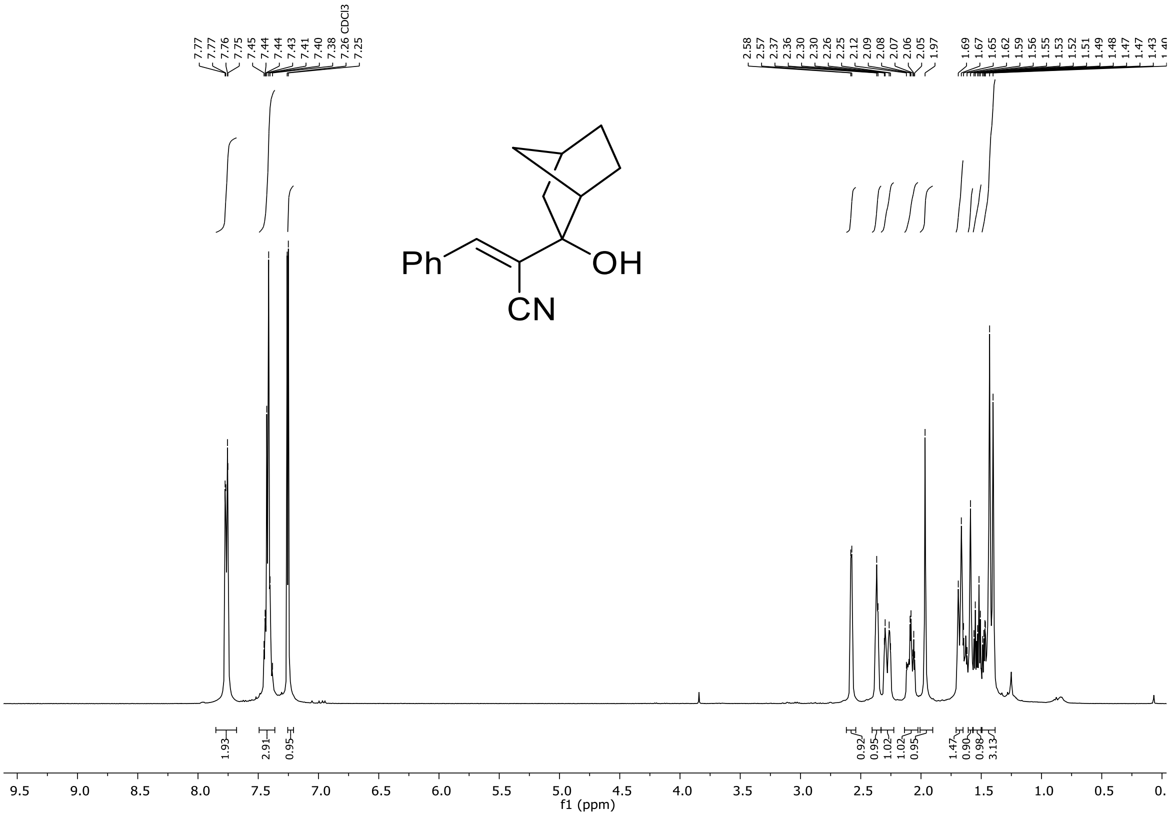

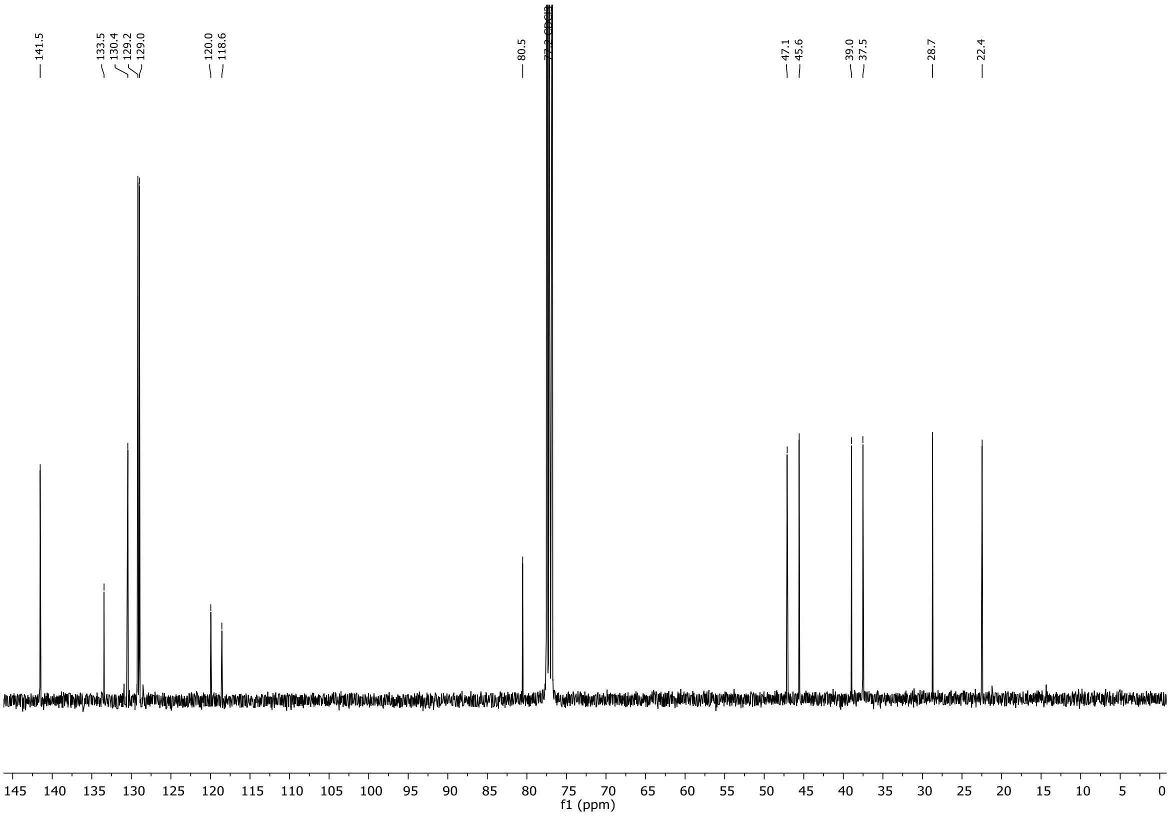

(*E*)-2-Benzylidene-3-cyclopropyl-3-hydroxybutanenitrile (4ai)

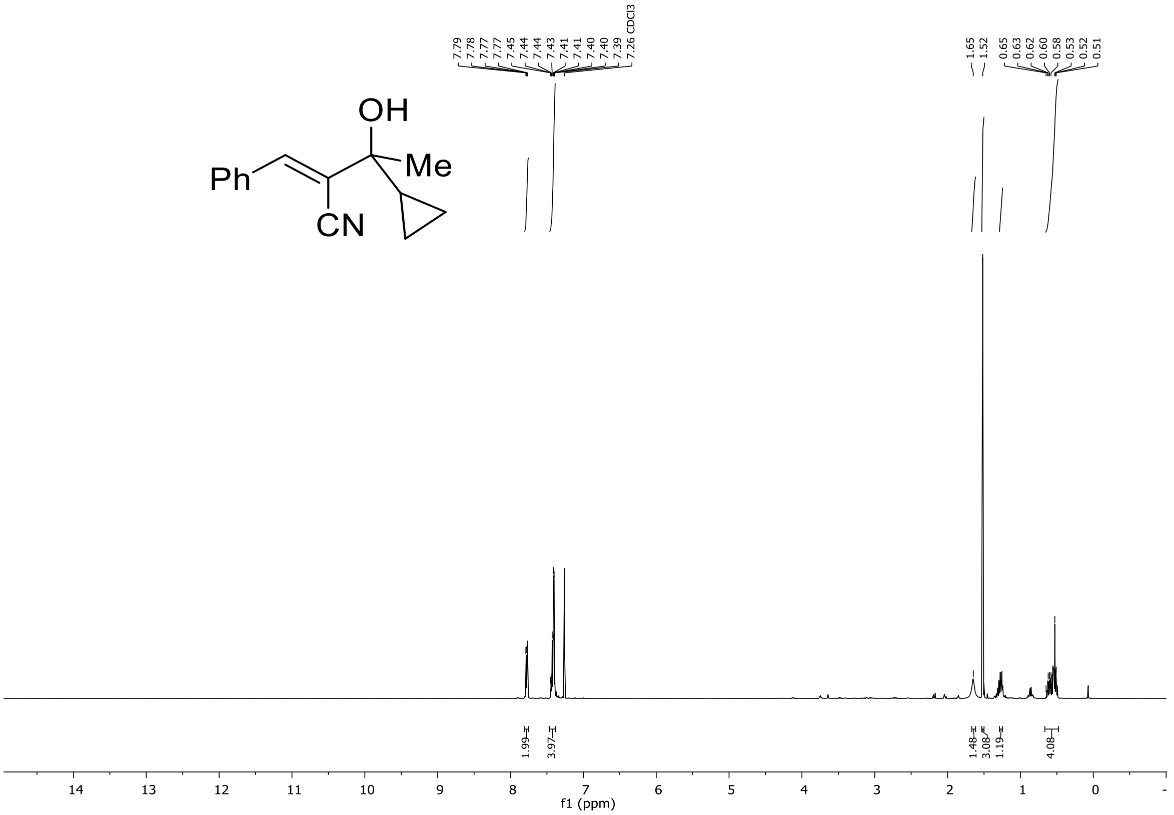

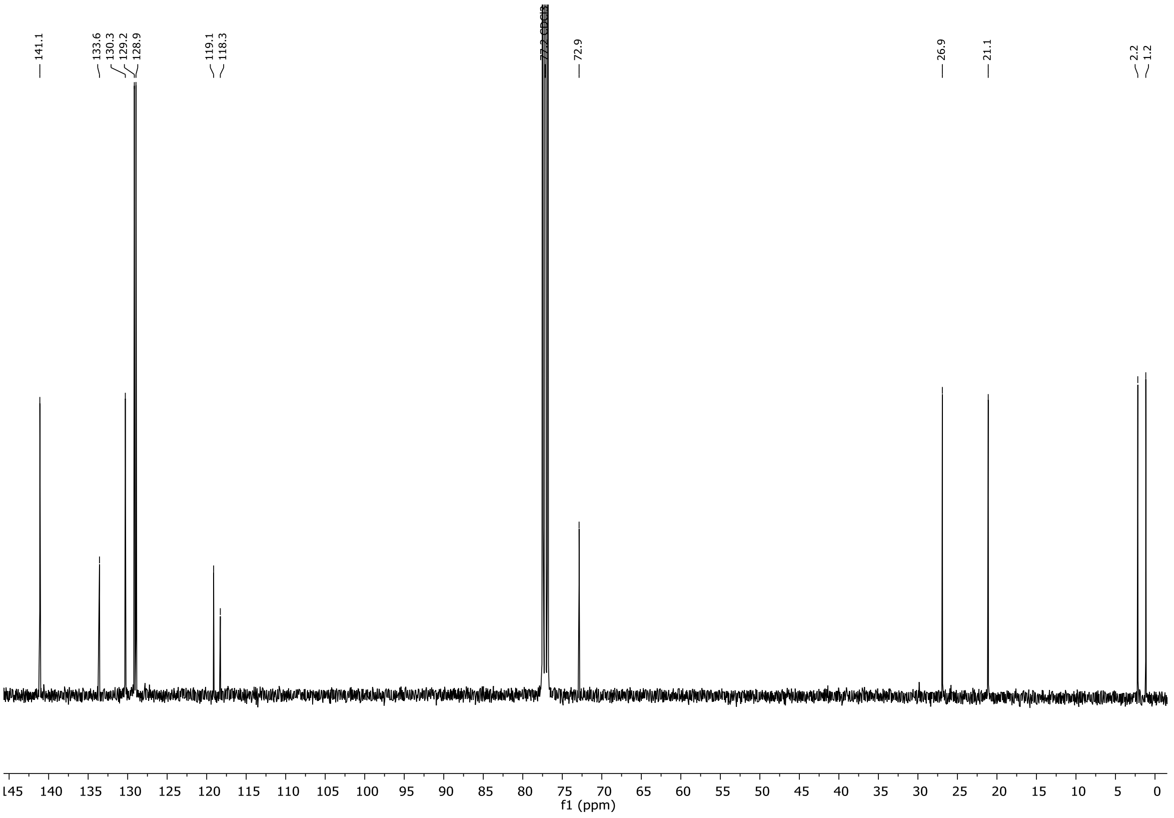

(*E*)-2-Benzylidene-3-hydroxy-3-methylheptanenitrile (4aj)

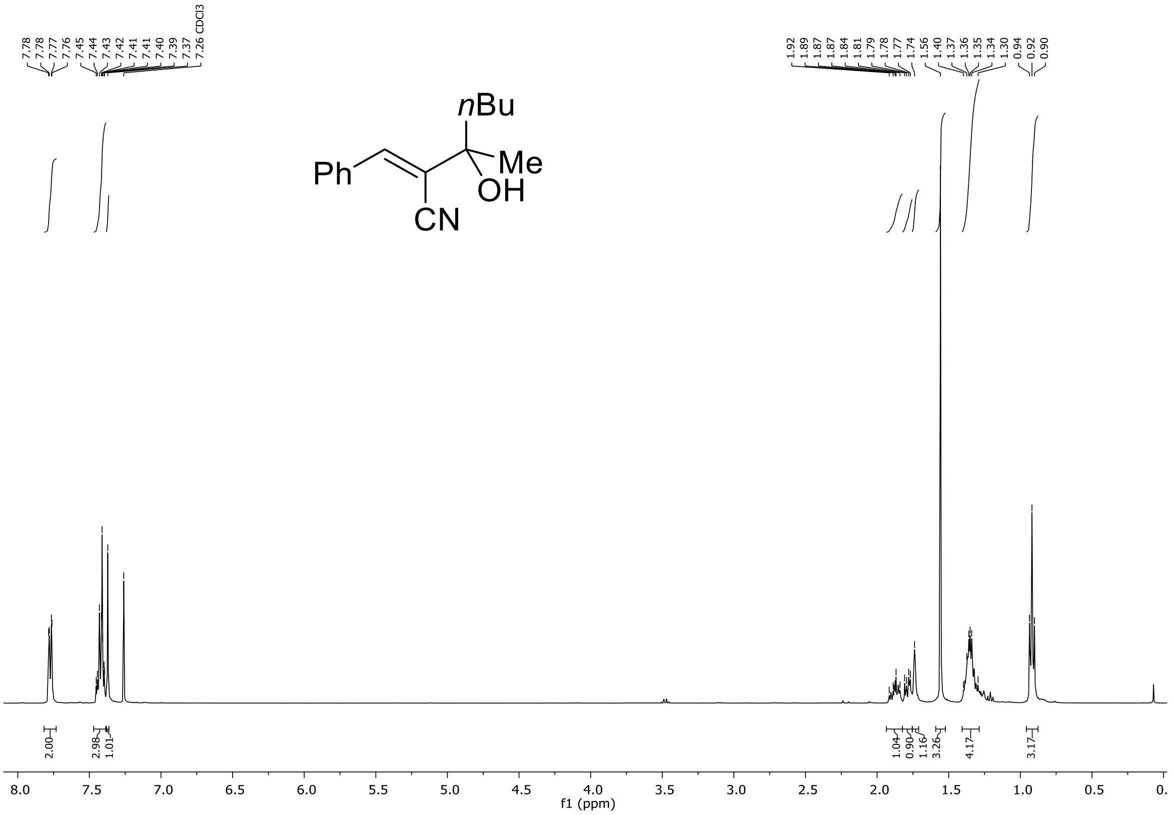

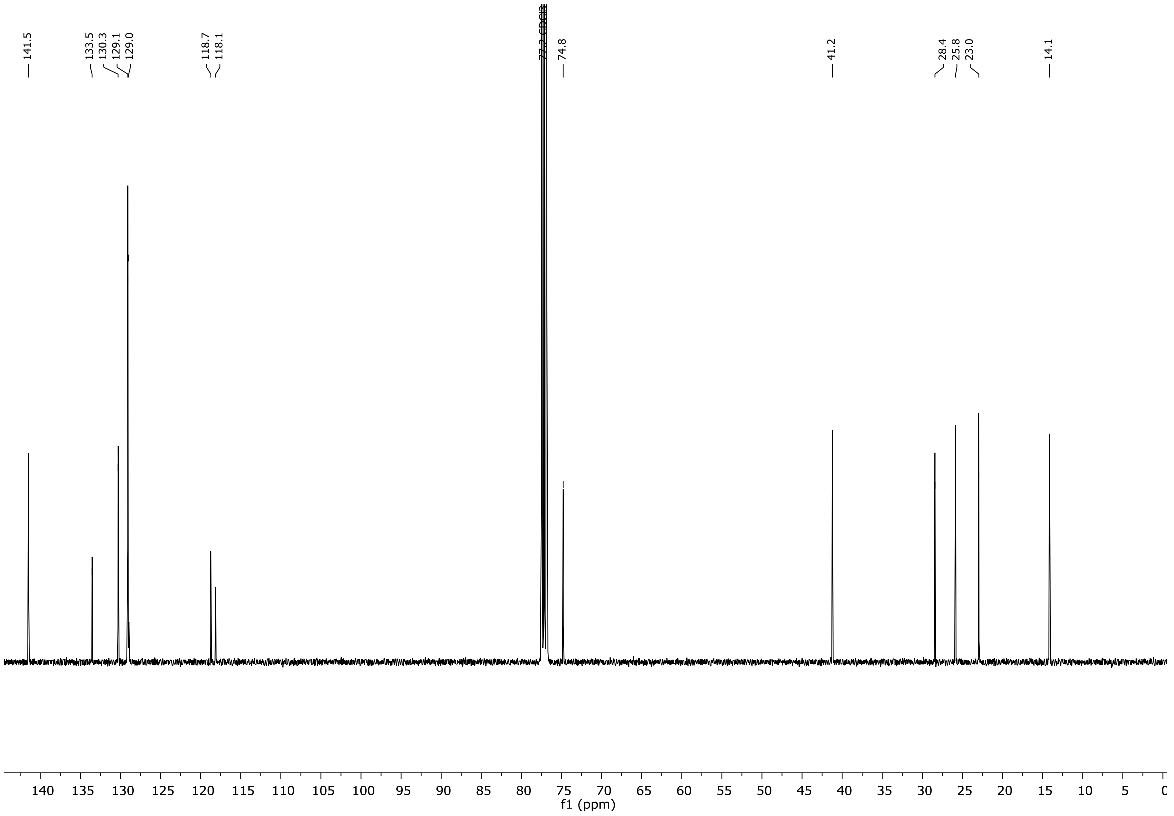

2-(Hydroxy(2-methoxyphenyl)methyl)-3-(4-methoxyphenyl) acrylonitrile (7ak)

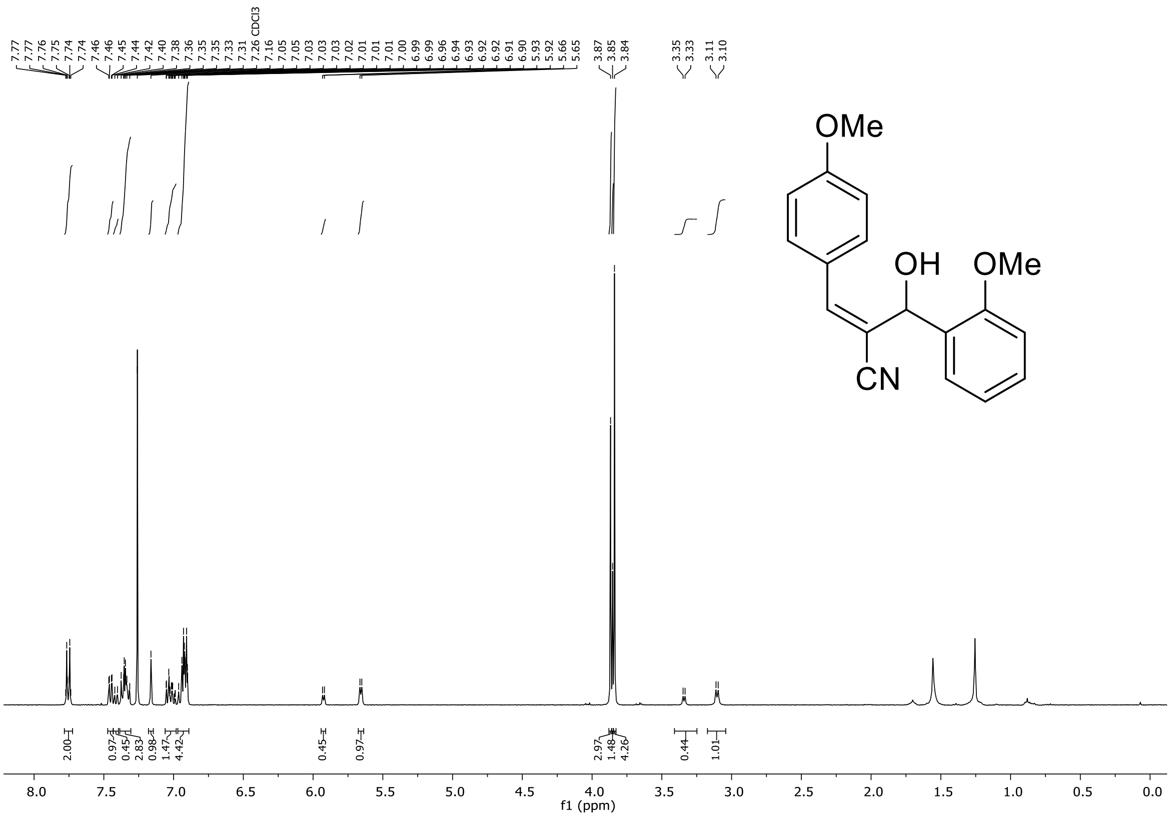

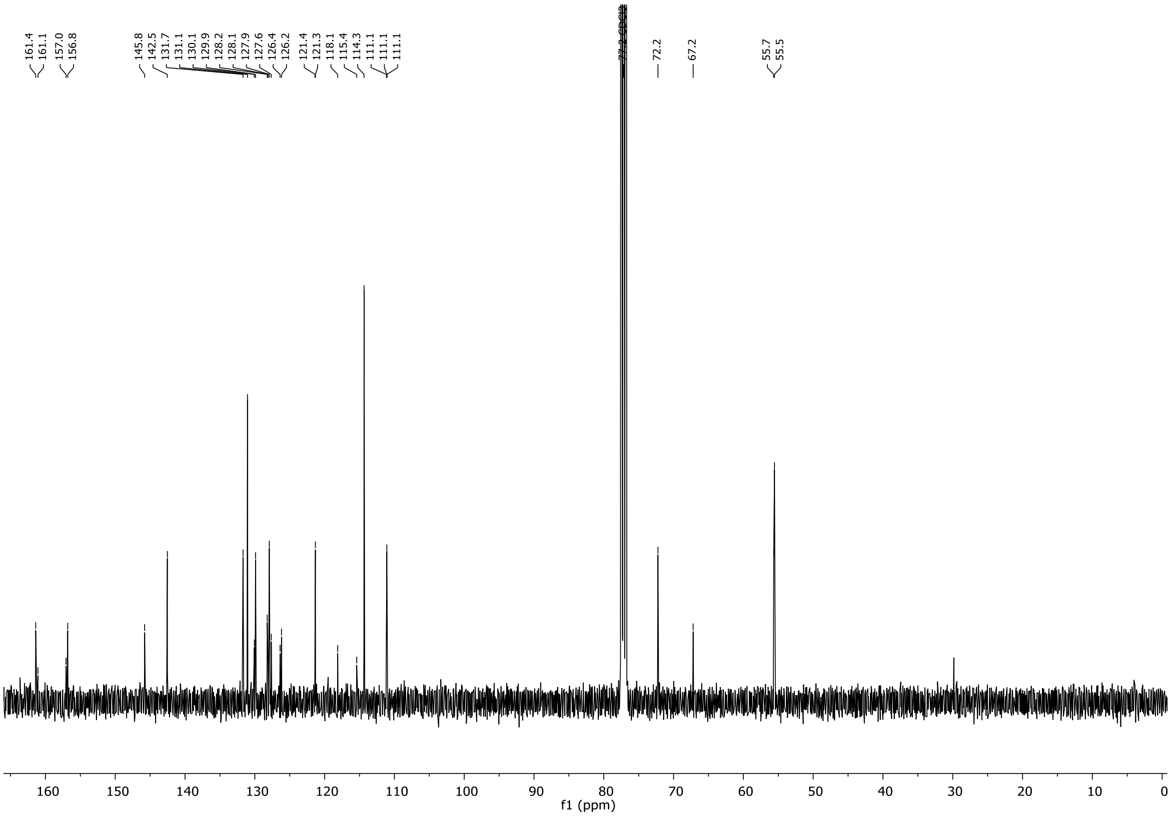

3-(3,4-Dimethylphenyl)-2-(hydroxy(2-methoxyphenyl)methyl)acrylonitrile (7bk)

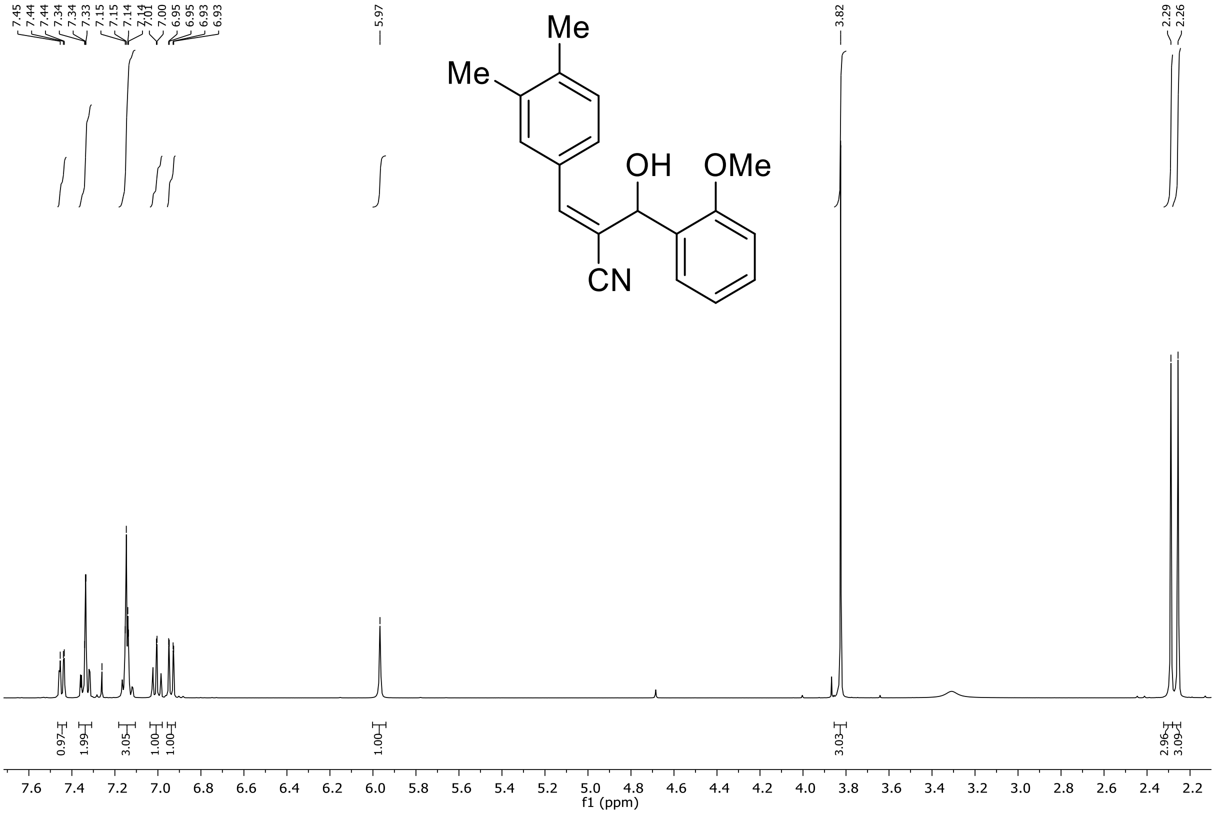

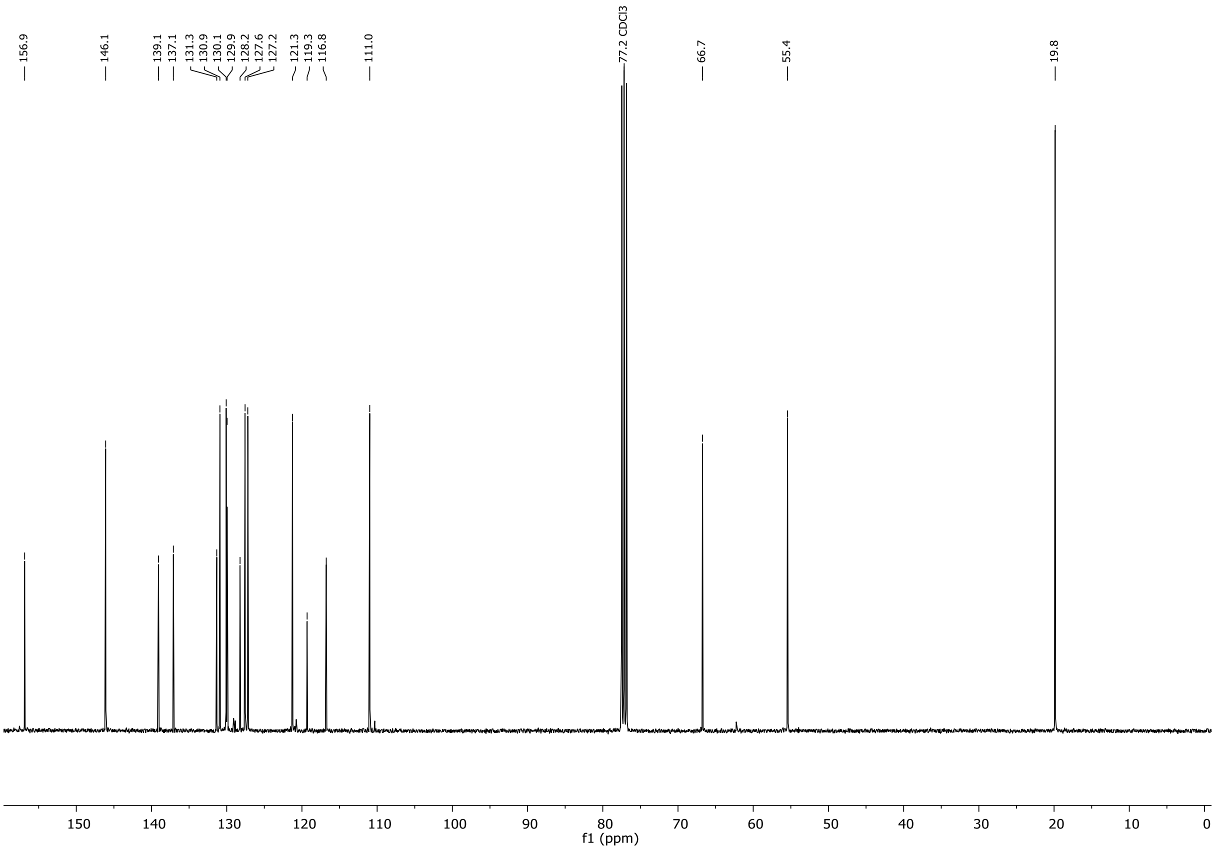

(*Z*)-2-(Cyclohexyl(hydroxy)methyl)-3-(2,3-dihydrobenzo[*b*][1,4]dioxin-6-yl)acrylonitrile (7cl)

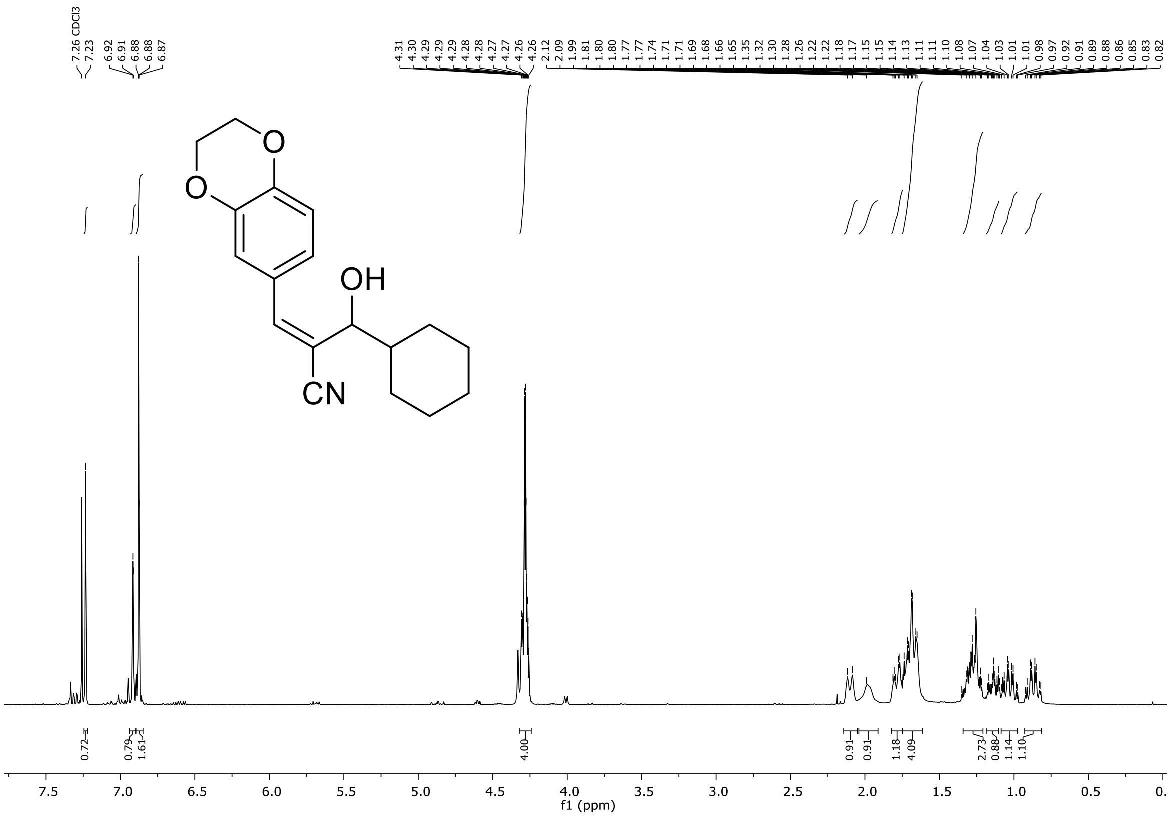

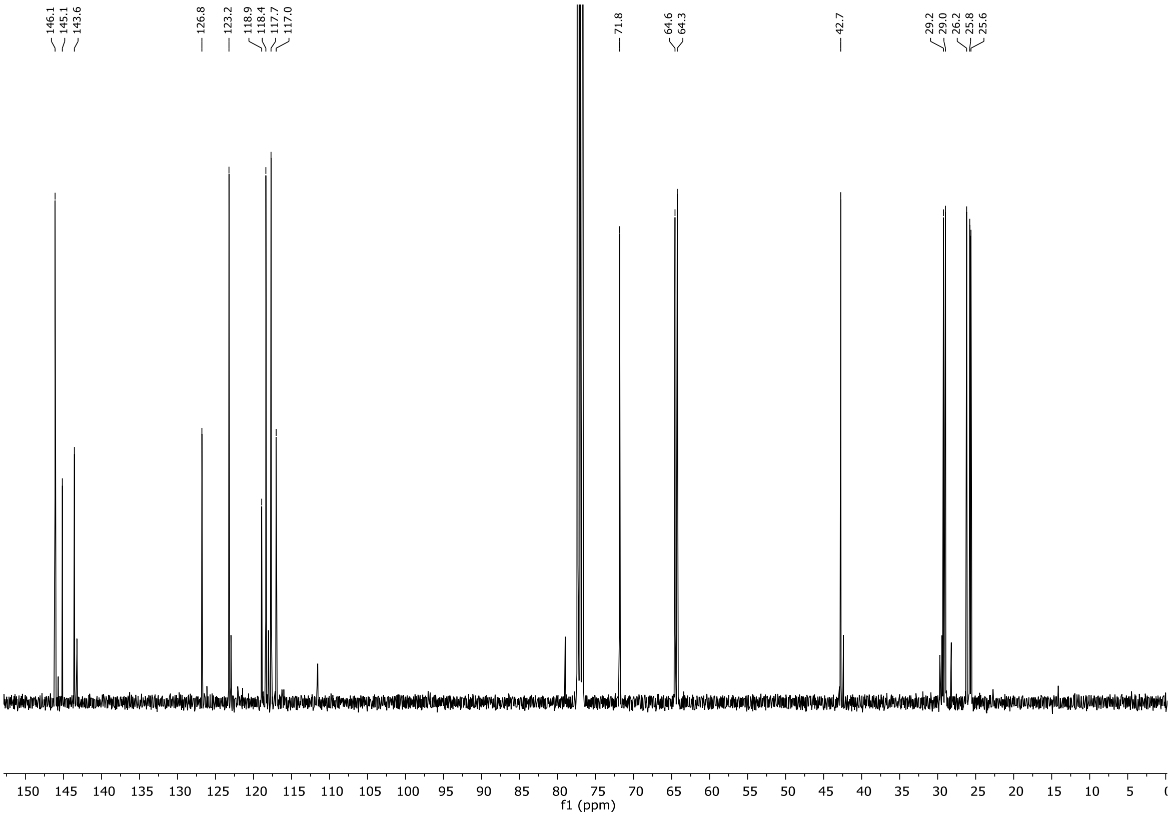

(*E*)-3-(4-(*Tert*-butyl)phenyl)-2-(1-hydroxycyclohexyl)acrylonitrile (7dm)

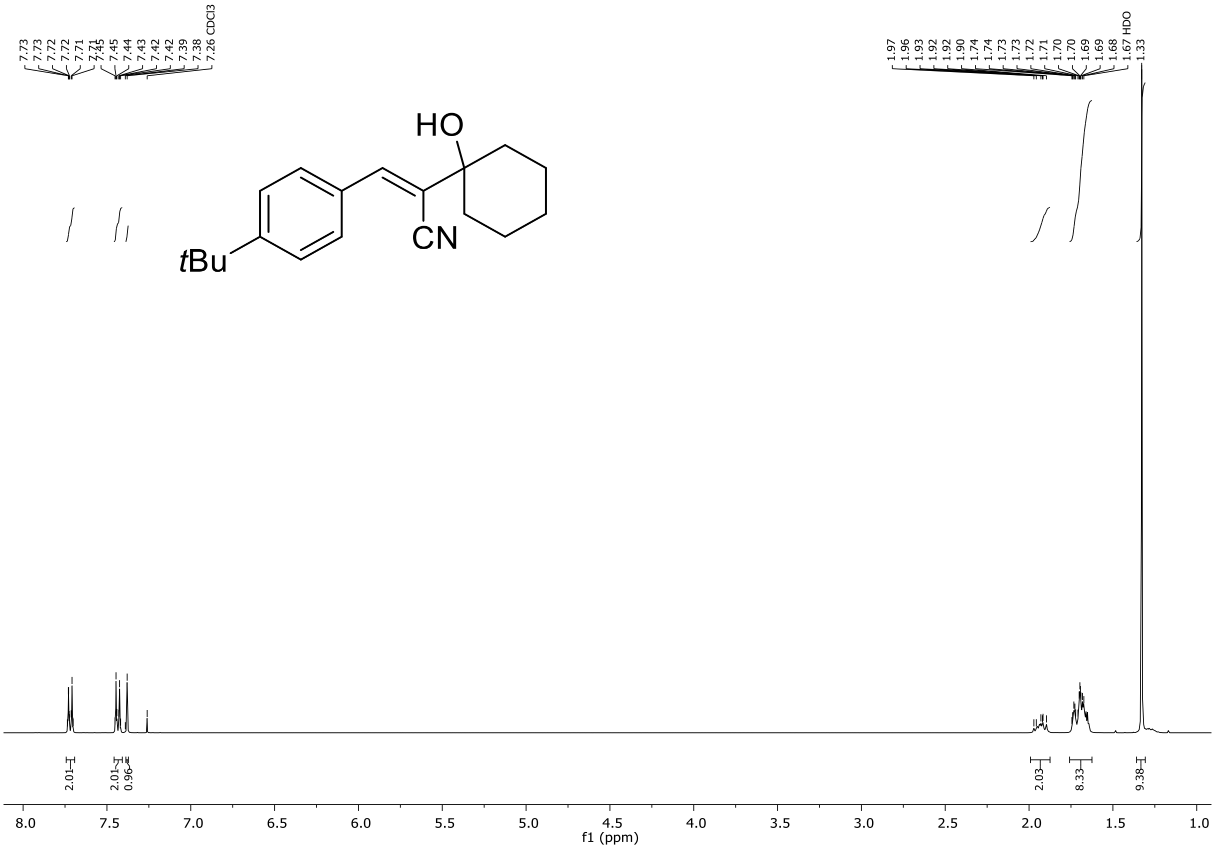

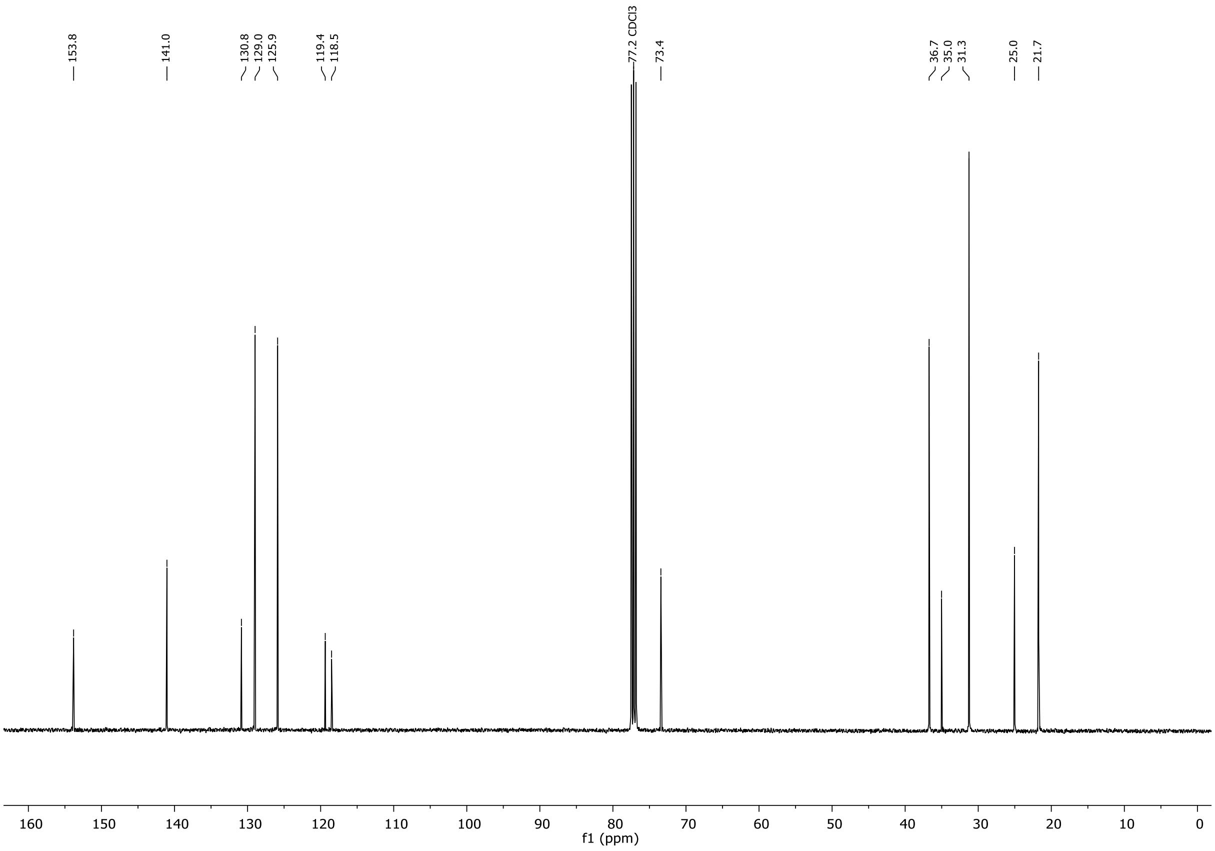

(*E*)-3-(4-(*Tert*-butyl)phenyl)-2-(cyclohex-2-en-1-yl)acrylonitrile (7de)

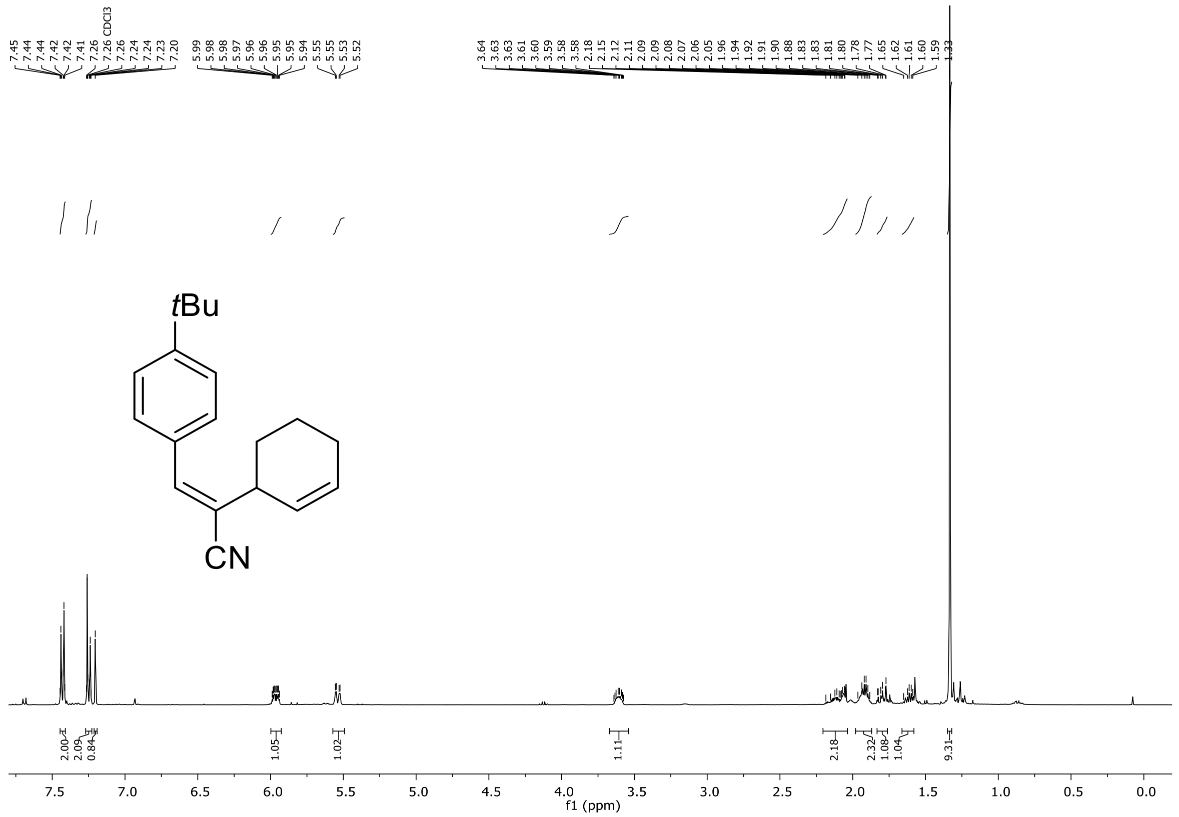

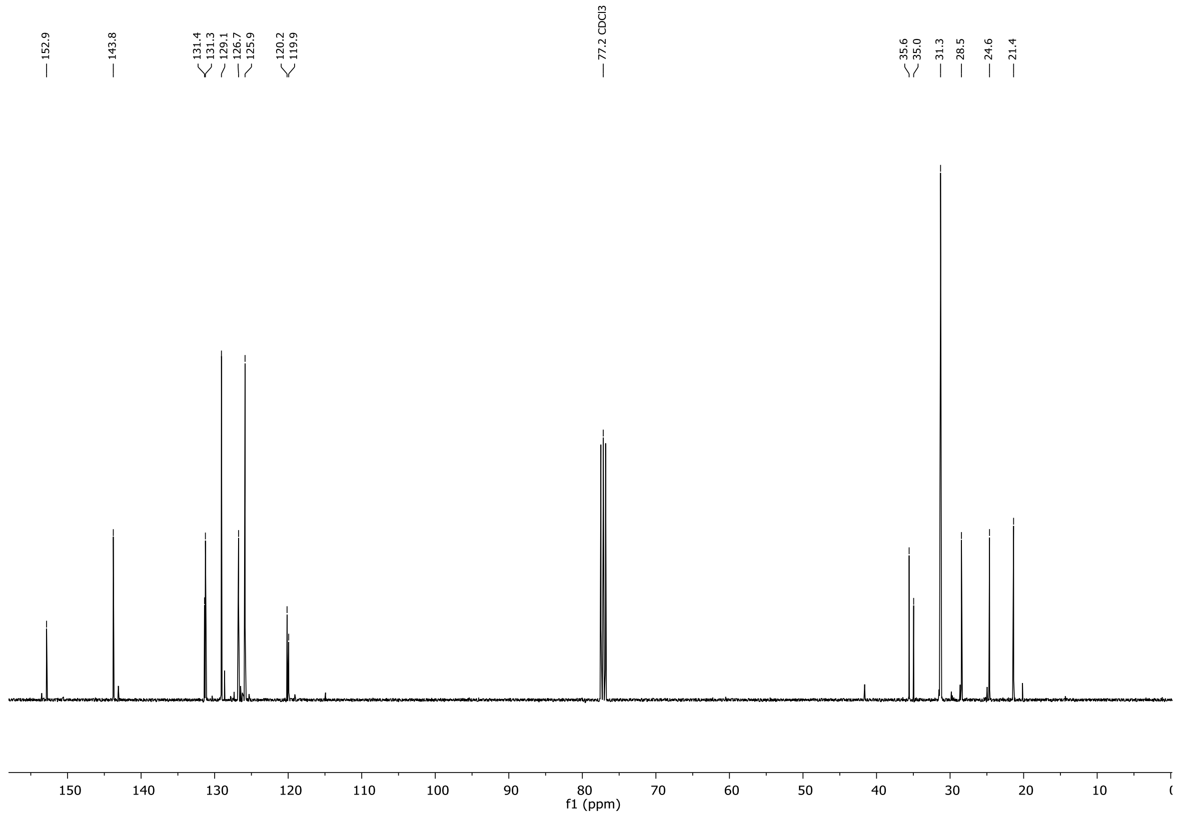

(*E*)-2-(Cyclohex-2-en-1-yl)-3-(4-(trifluoromethyl)phenyl)acrylonitrile (7ee)

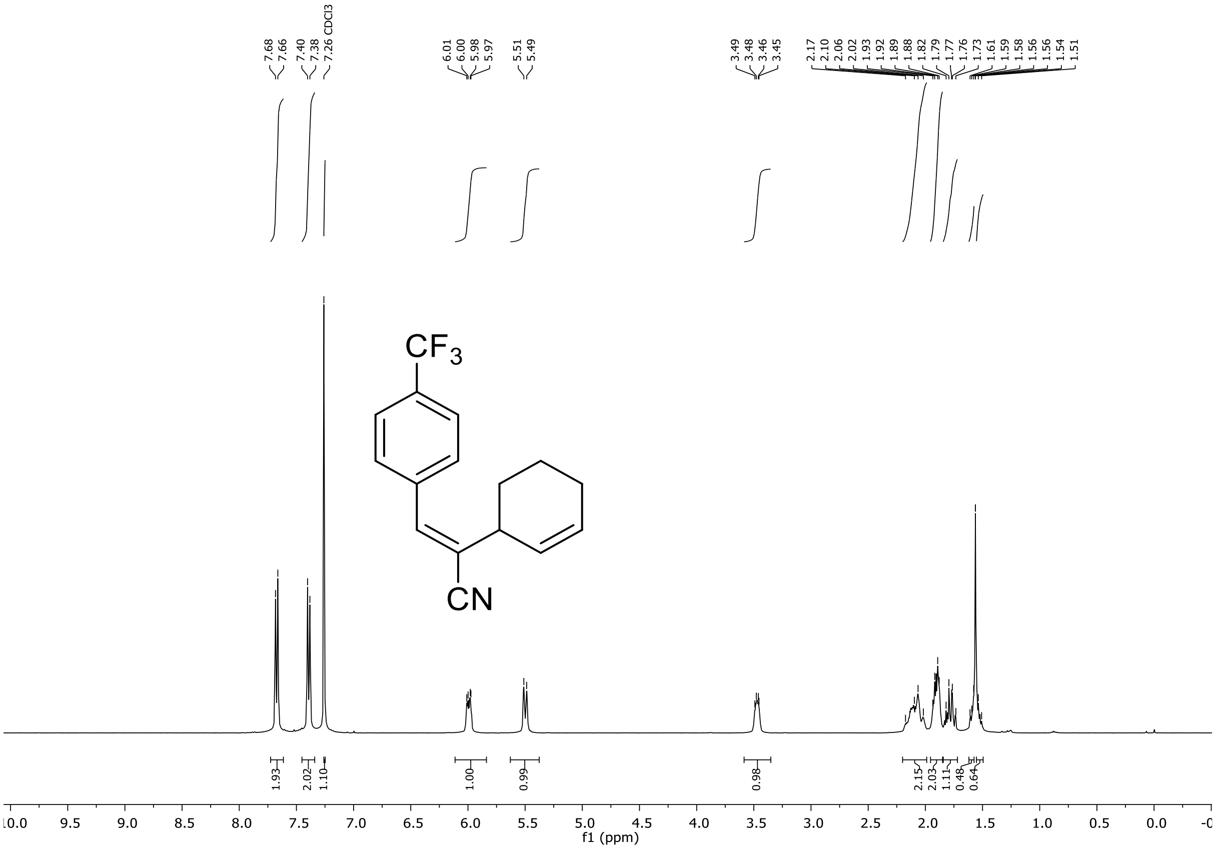

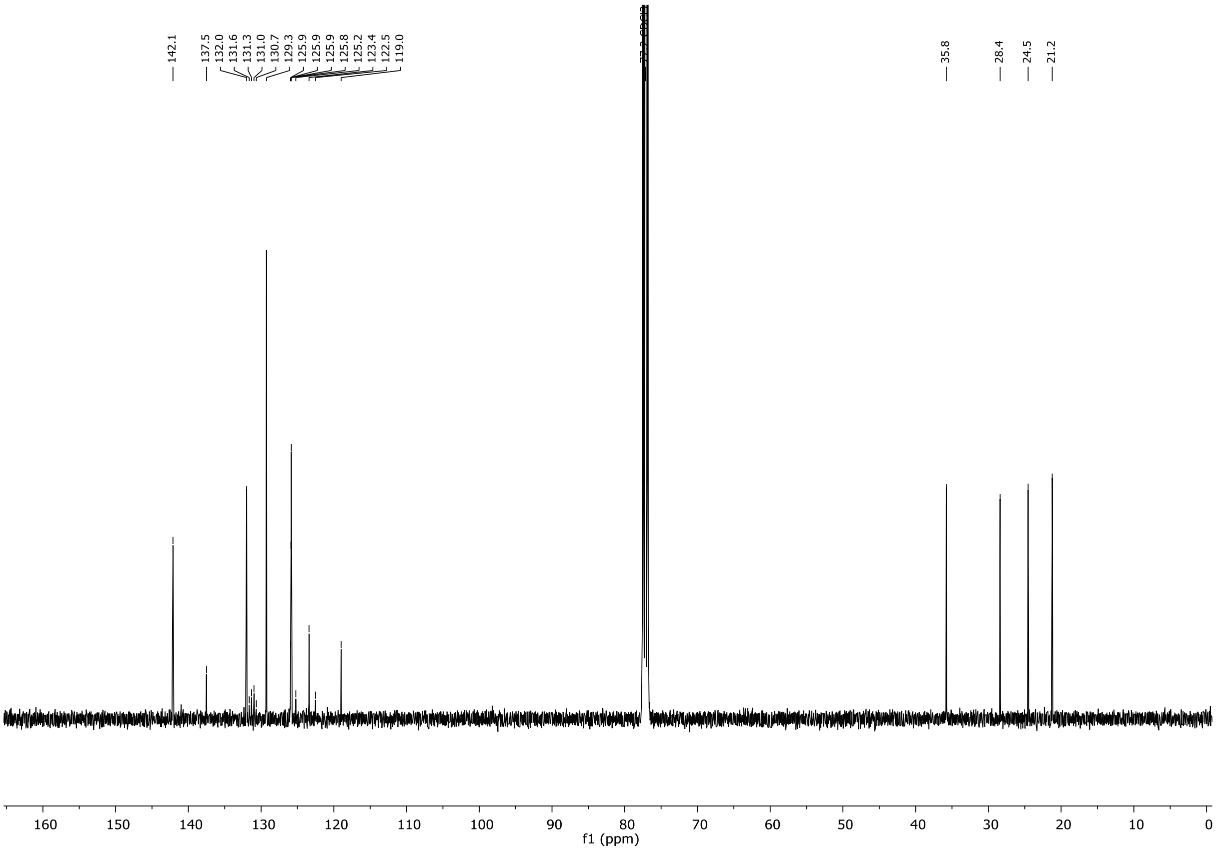

(*Z*)-4-Hydroxy-3-methoxy-4-(4-(trifluoromethyl)phenyl)but-2-enenitrile (7fn)

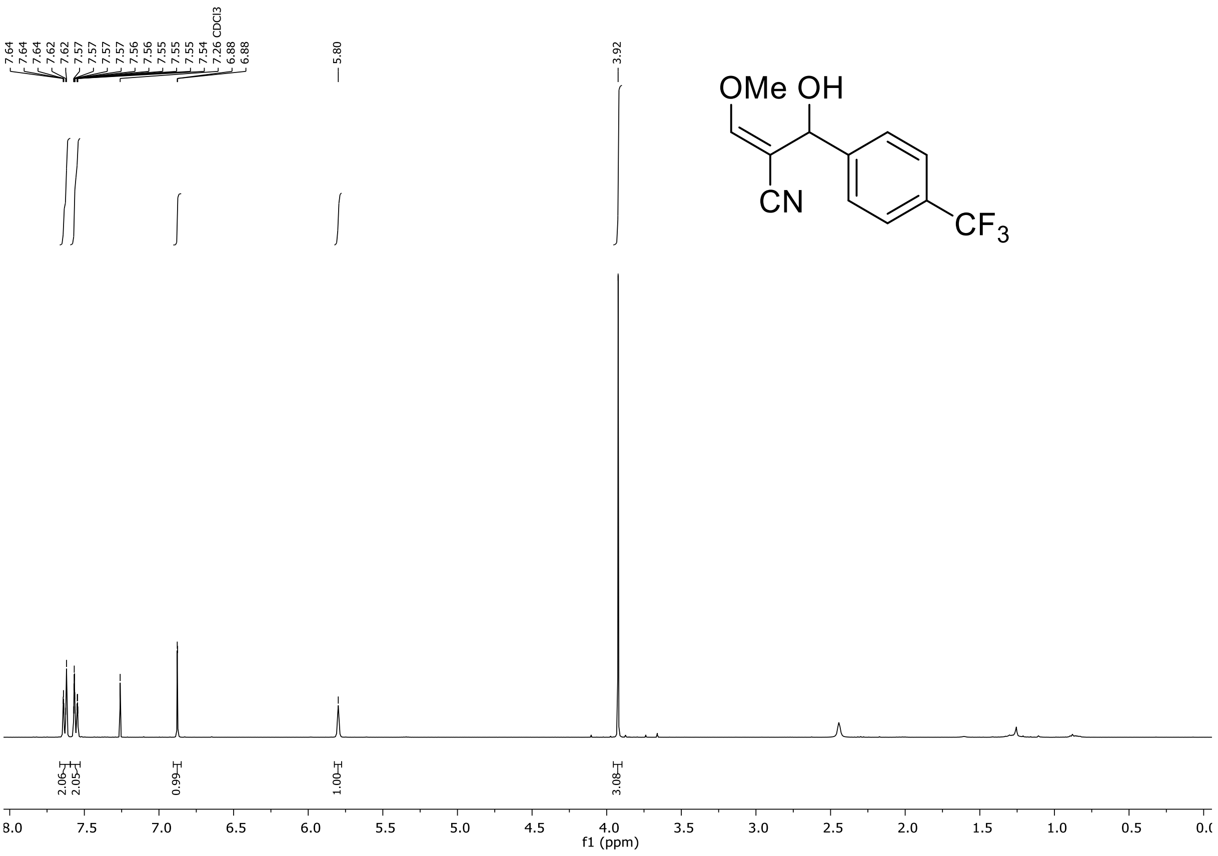

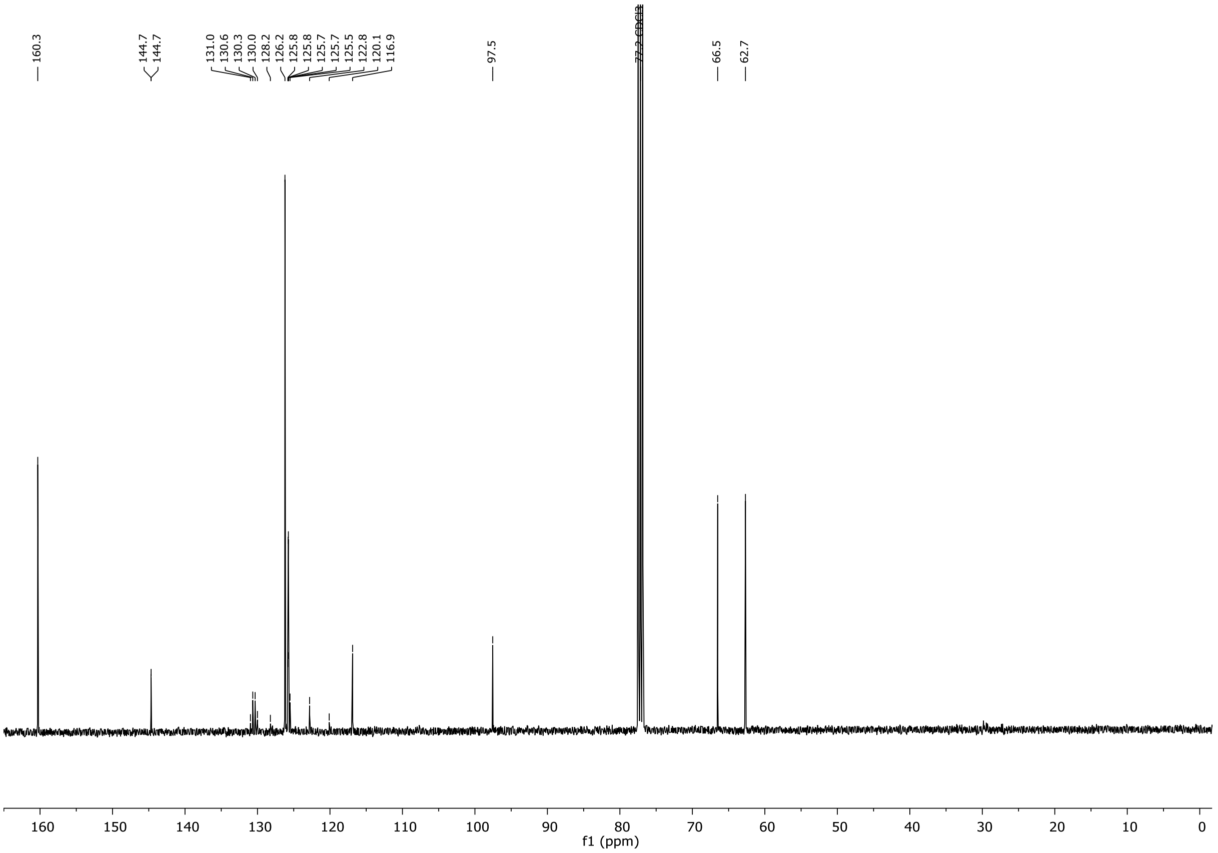

(*Z*)-2-(Hydroxy(*p*-tolyl)methyl)-3-methoxyacrylonitrile (7fd)

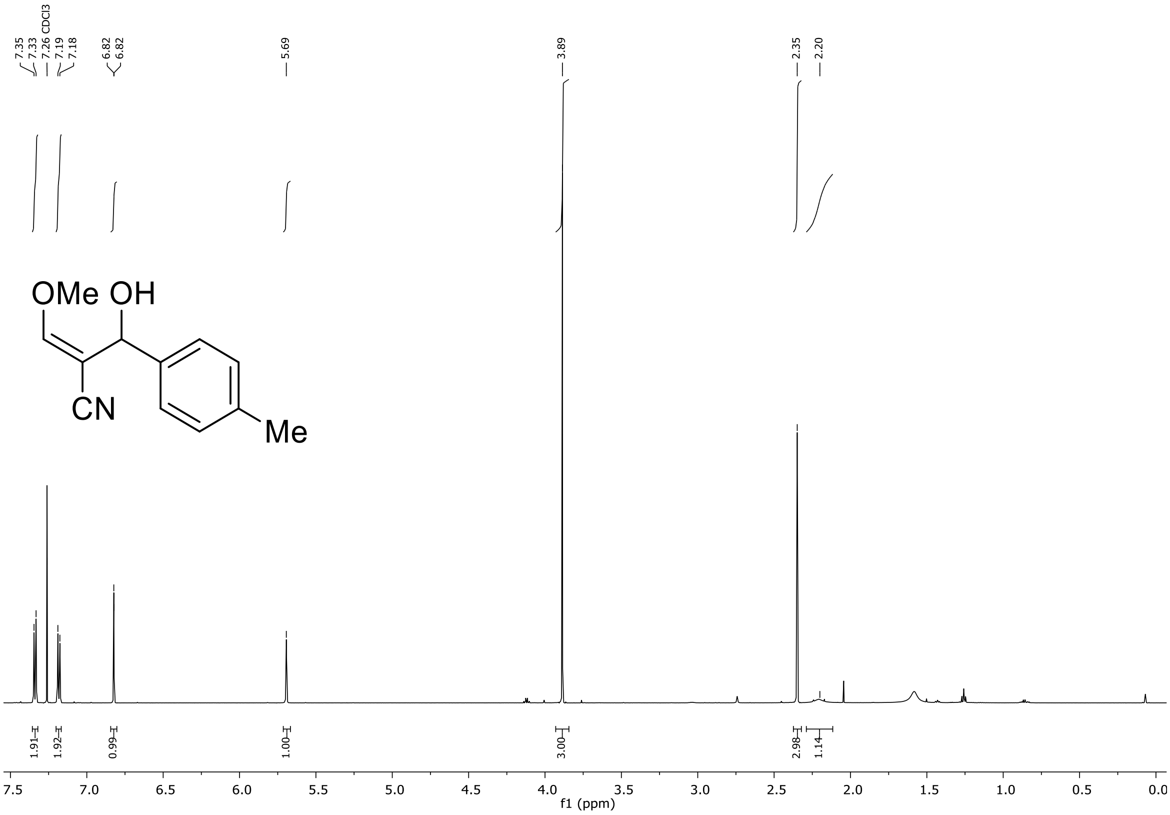

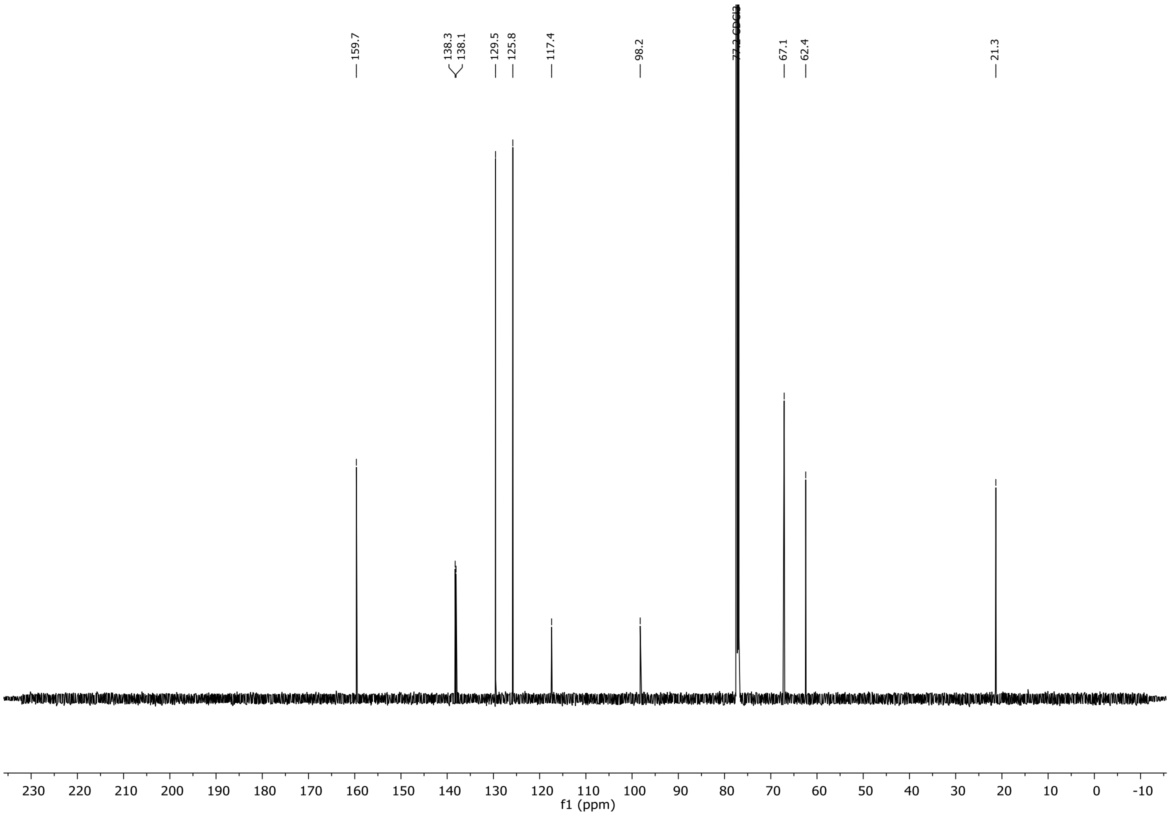

(*Z*)-4-Cyclohexyl-3-ethoxy-4-hydroxybut-2-enenitrile (7gc)

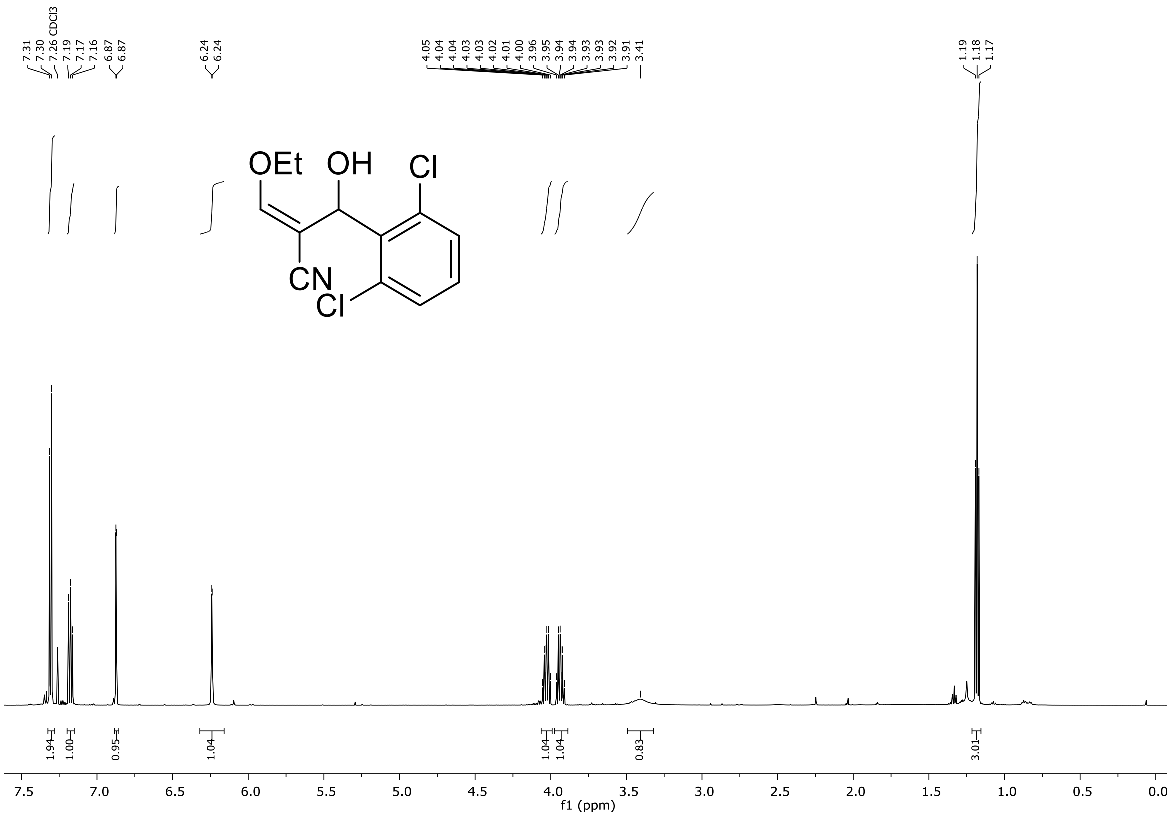

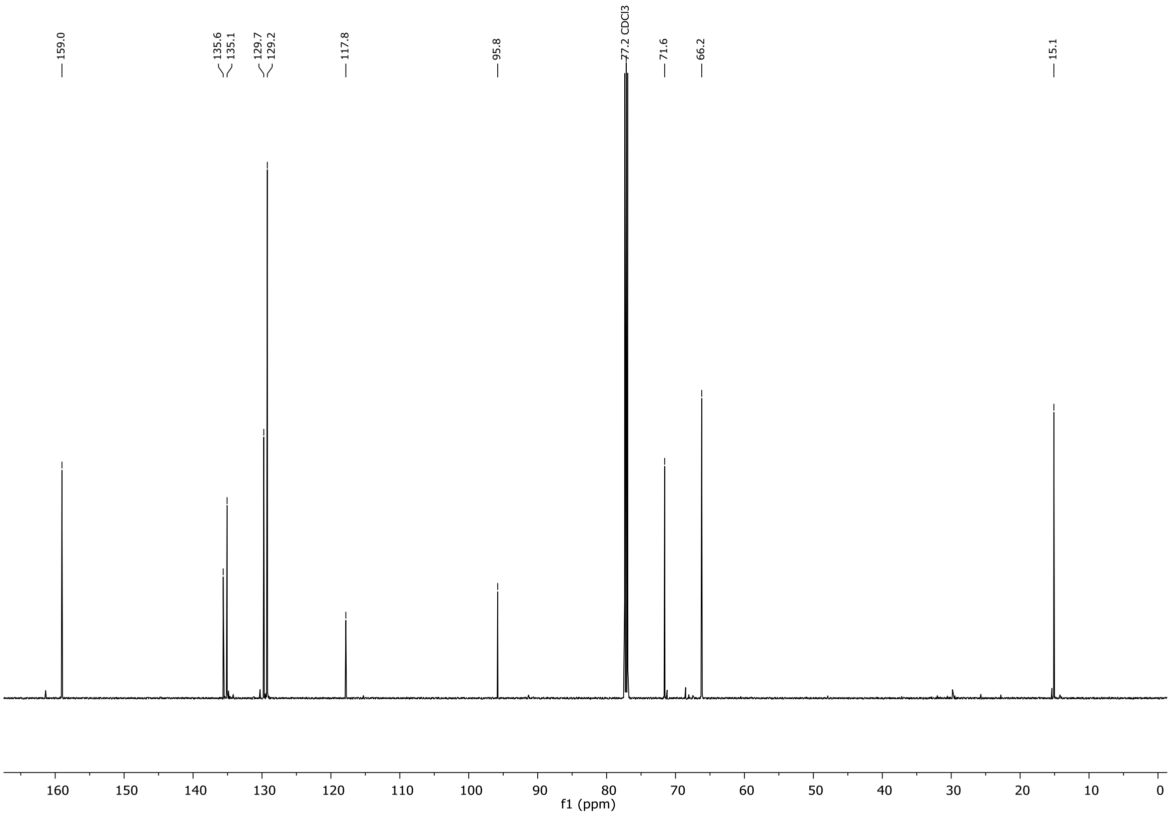

(*Z*)-4-Cyclohexyl-3-ethoxy-4-hydroxybut-2-enenitrile (7gl)

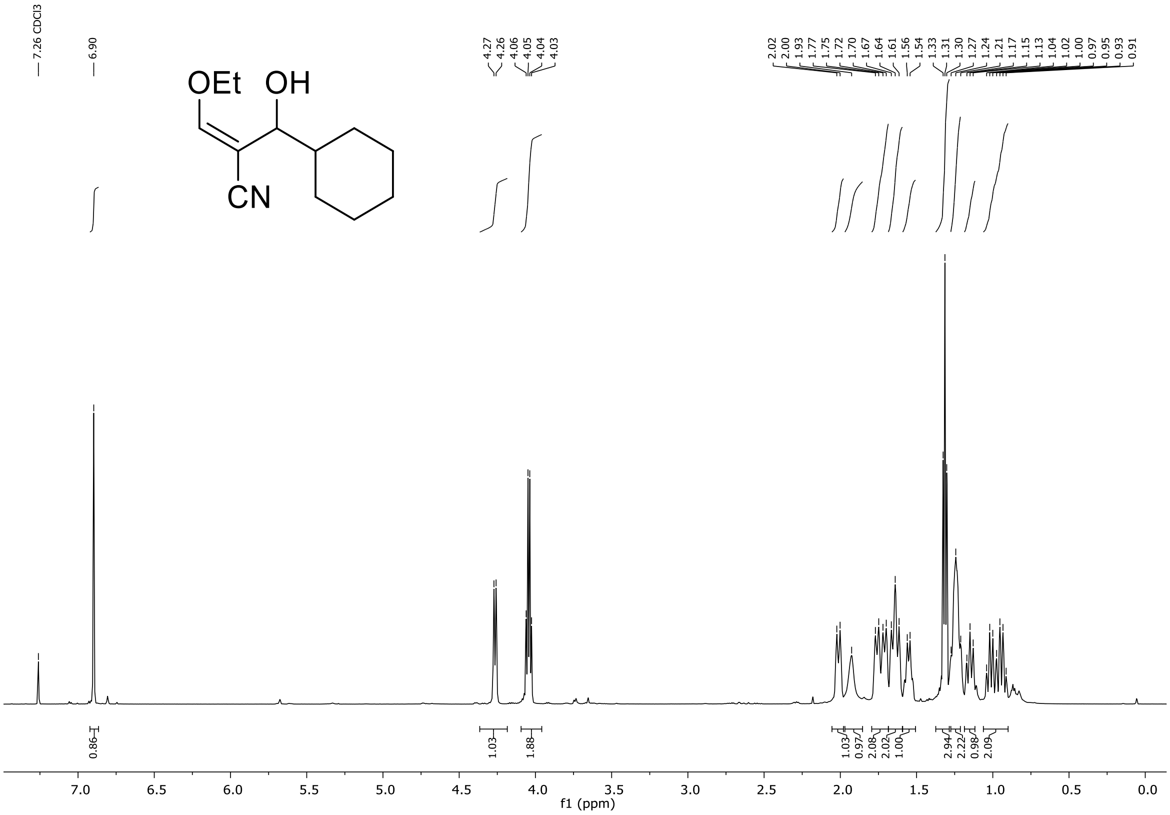

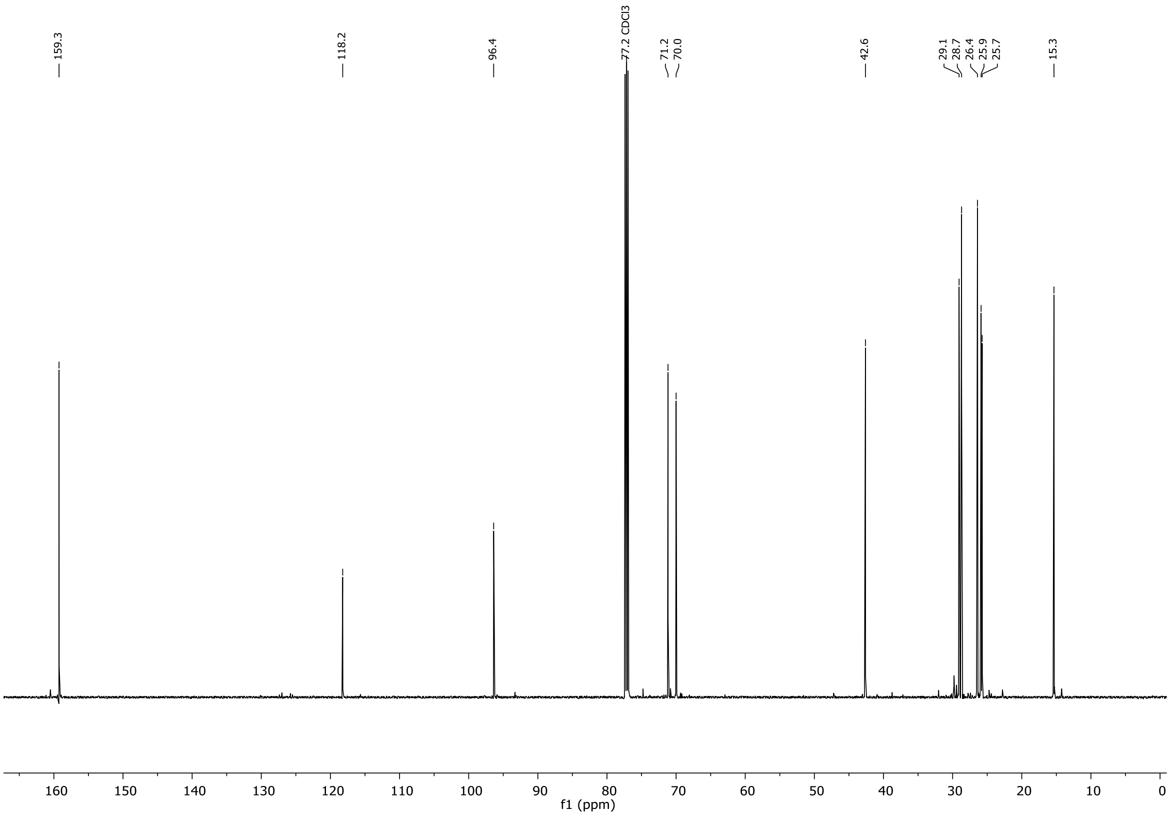

2-(2-Phenyl-1-(phenylthio)vinyl)adamantan-2-ol (7ho)

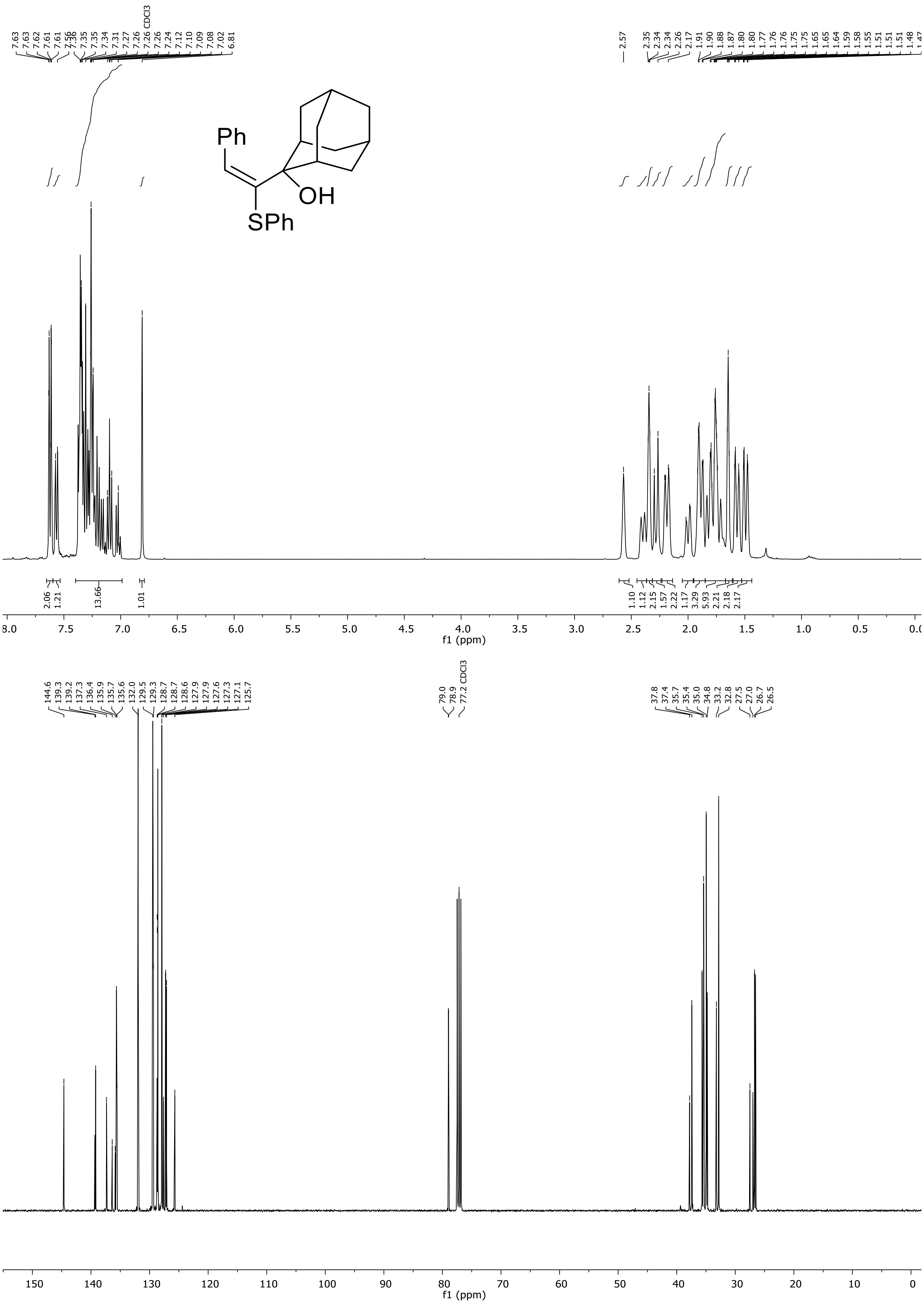

1,1,3-Triphenyl-2-(phenylthio)prop-2-en-1-ol (7hg)

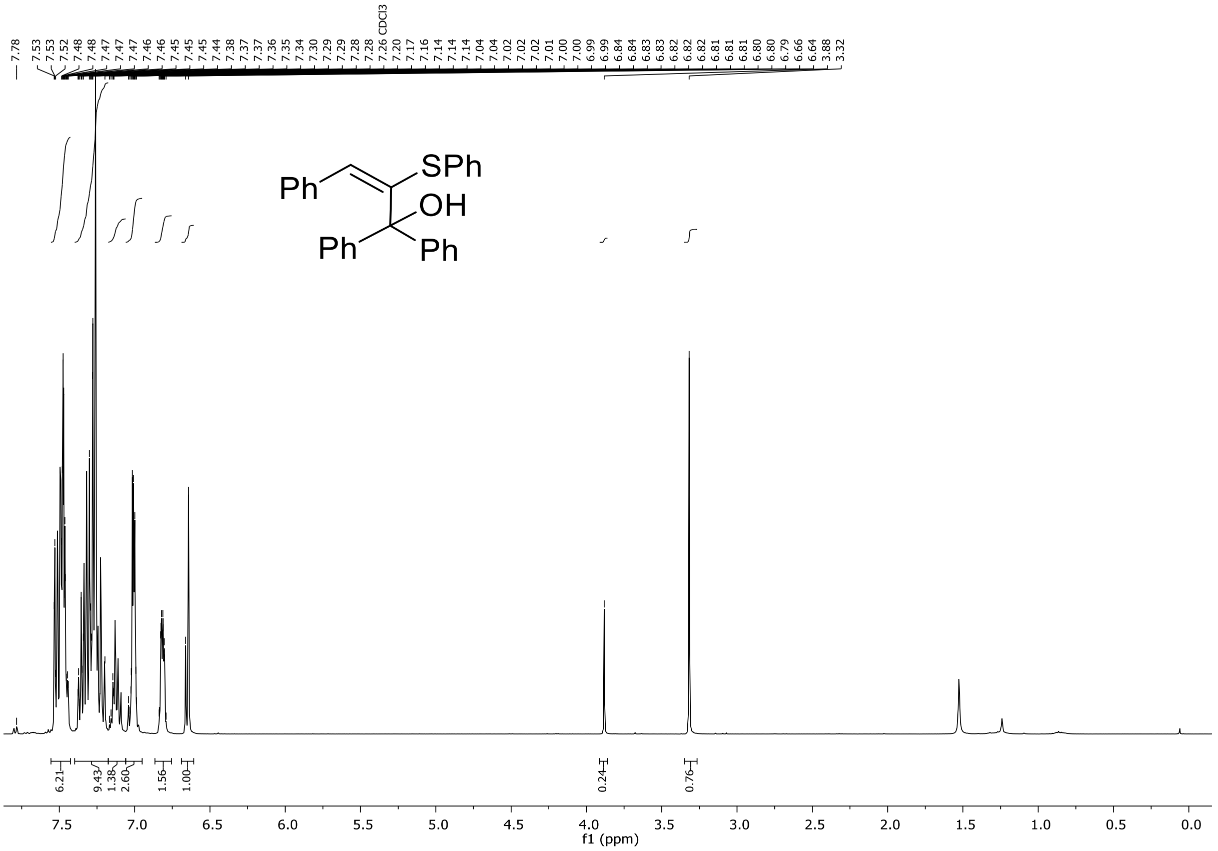

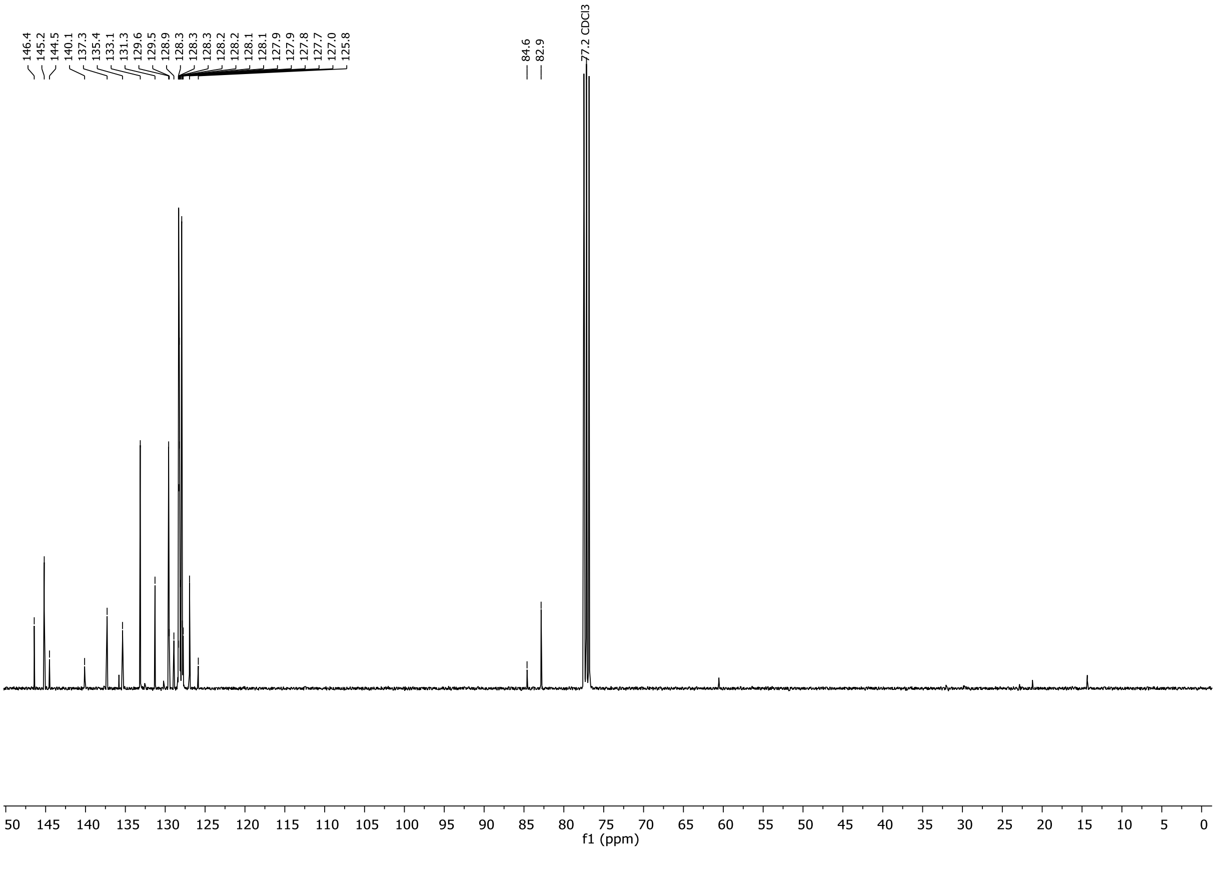

2-(Hydroxyiodomethyl)-3,7-dimethylocta-2,6-dienenitrile (10ap)

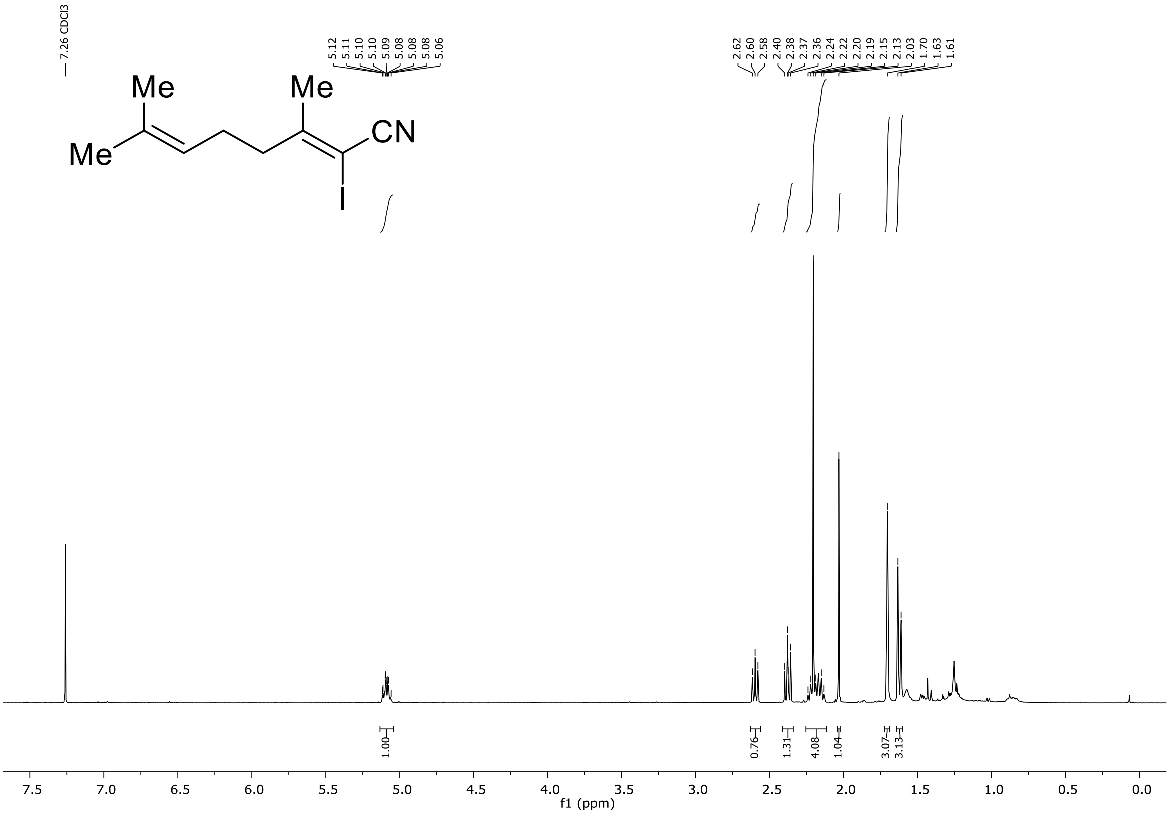

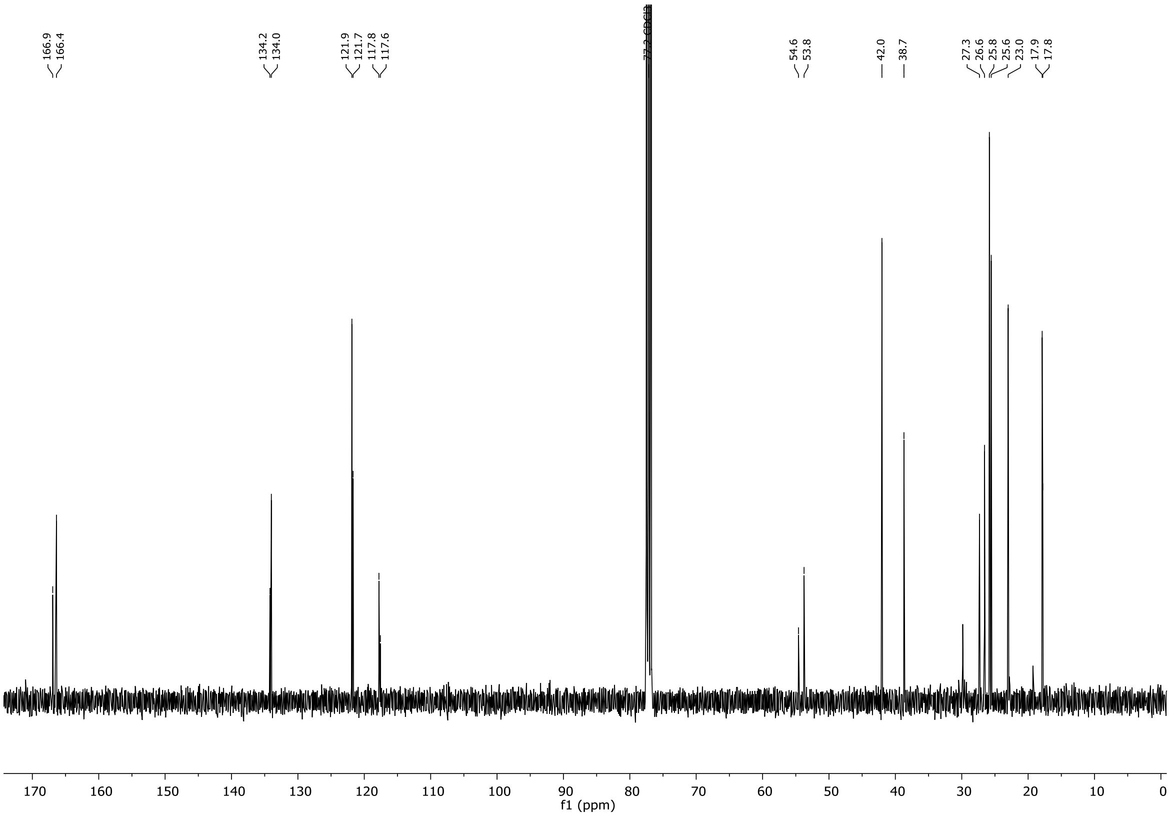

2-(Cyclohexyl(hydroxy)methyl)-3,7-dimethylocta-2,6-dienenitrile (10al)

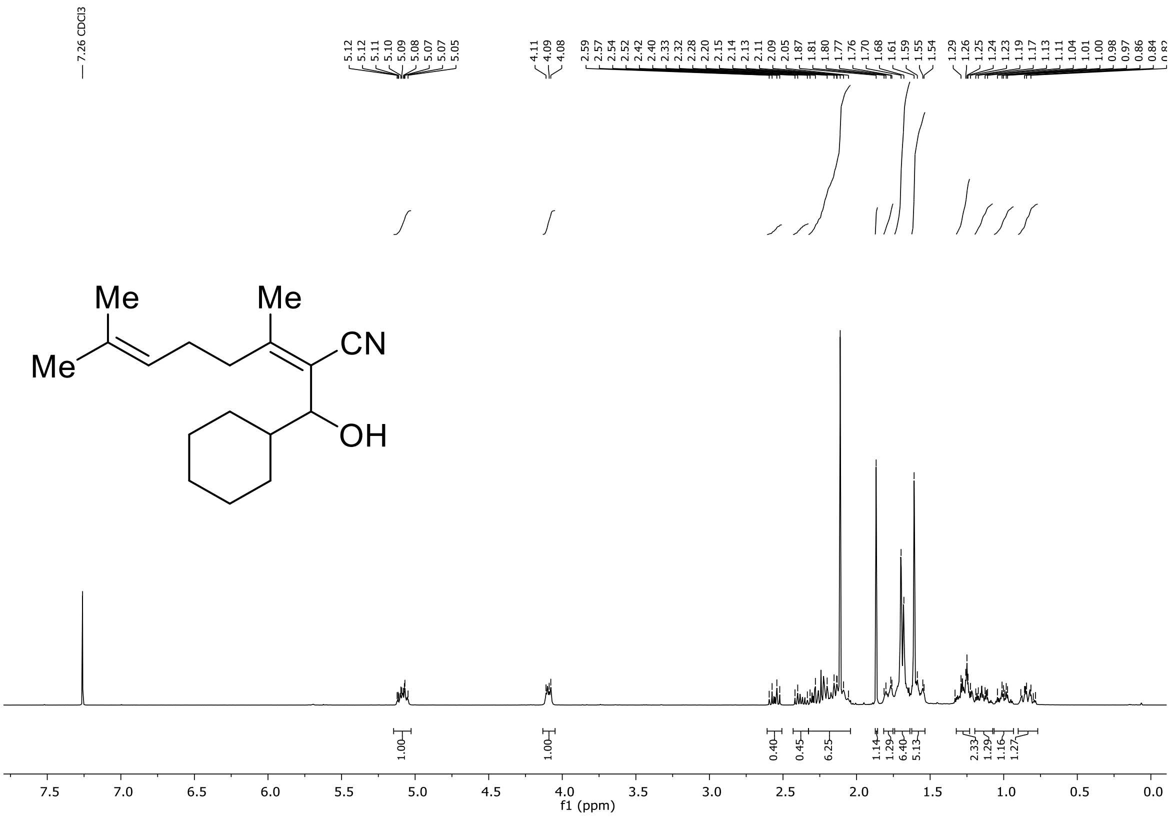

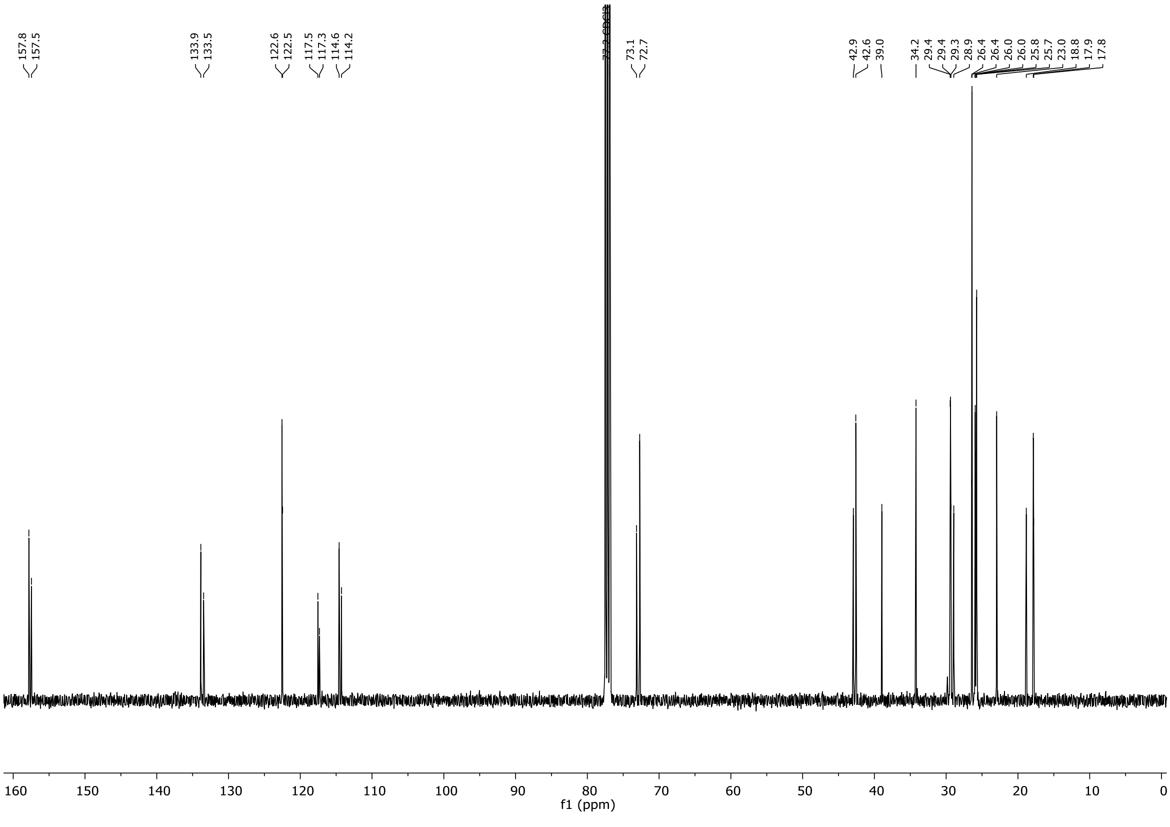

2-(1-Hydroxy-1,2,3,4-tetrahydronaphthalen-1-yl)-3,7-dimethylocta-2,6-dienenitrile (10aq)

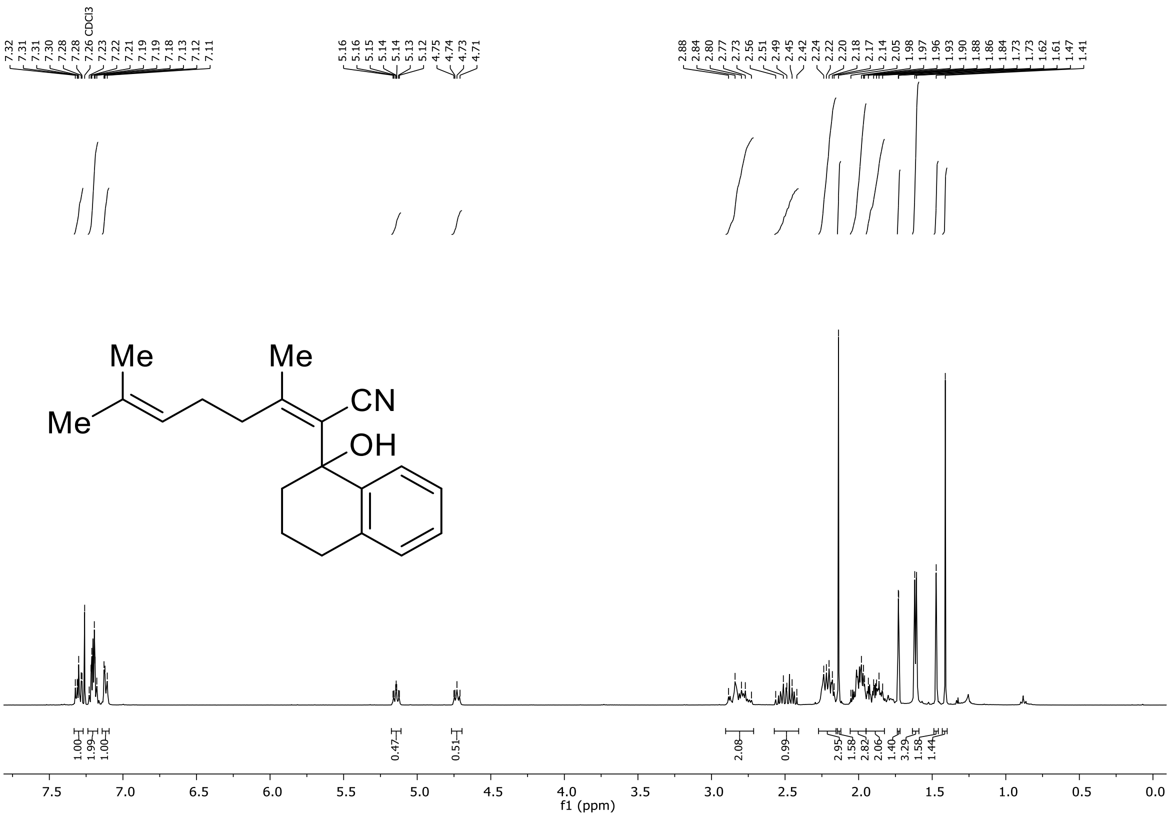

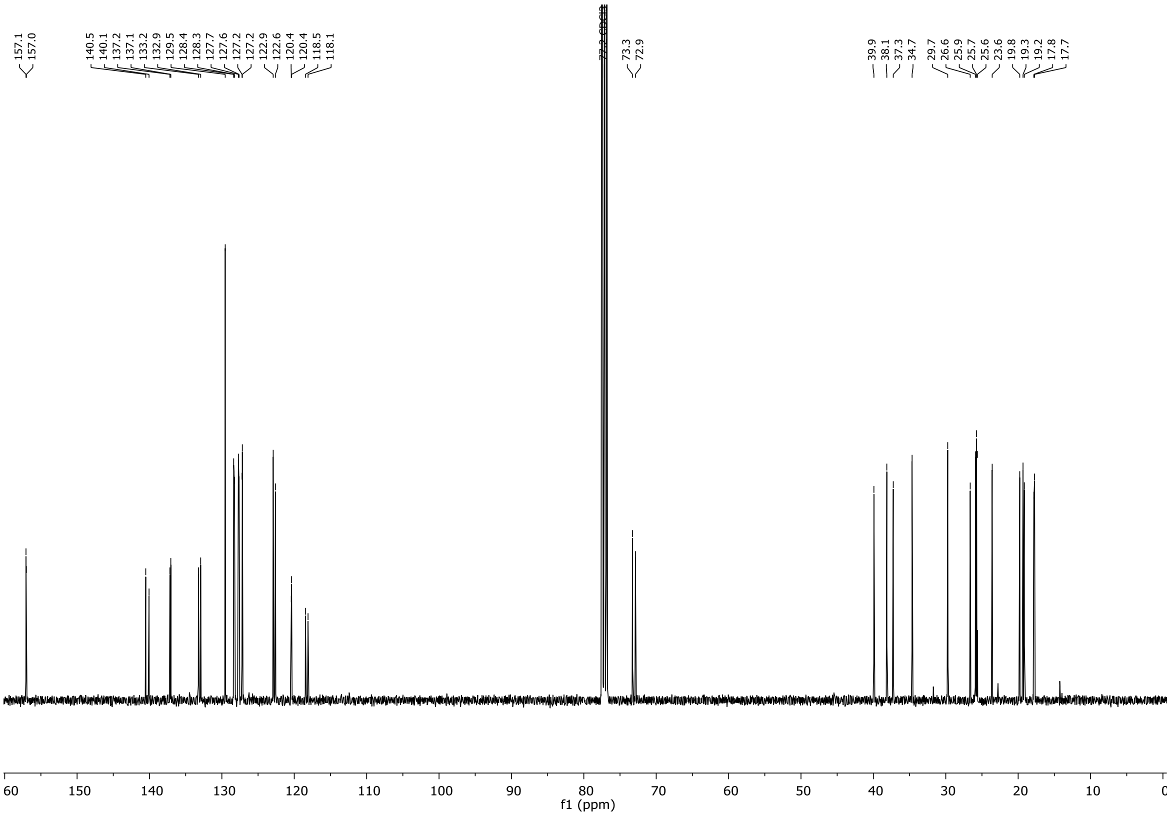

2-((4-Chlorophenyl)(cyclopropyl)(hydroxy)methyl)-3-methylhept-2-enenitrile (10br)

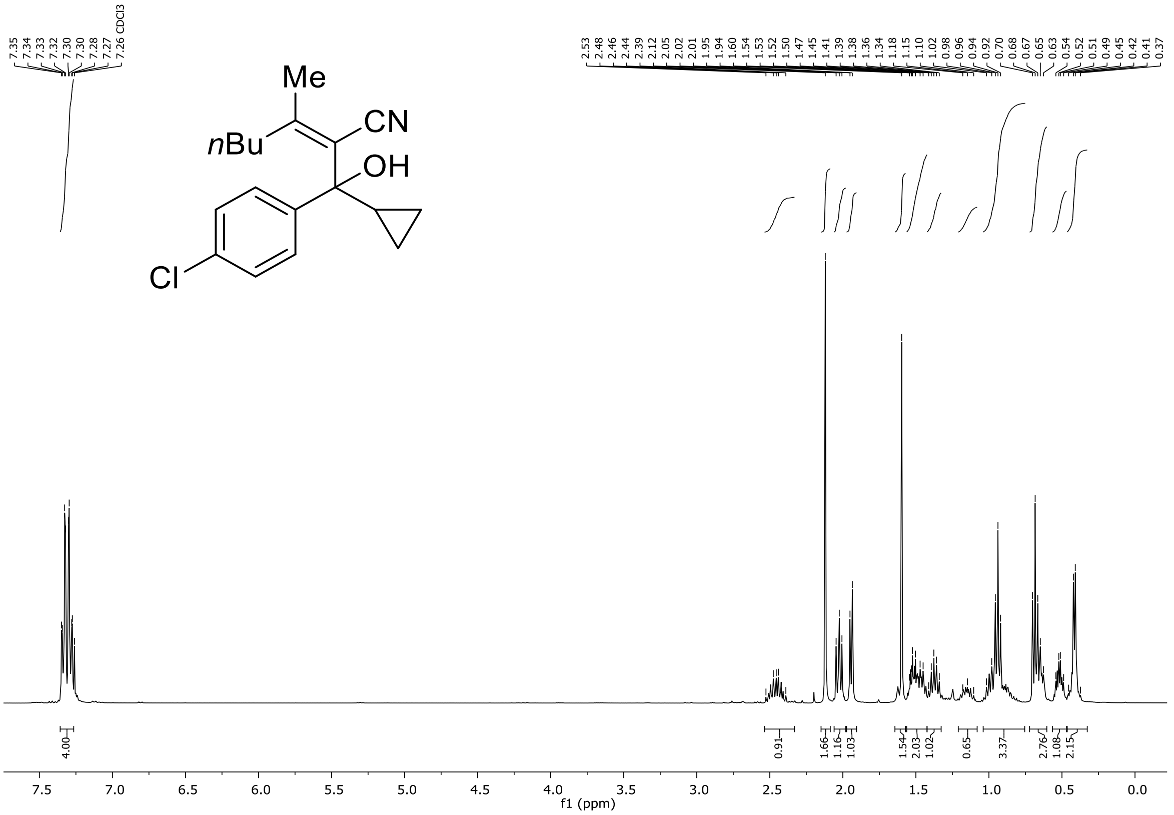

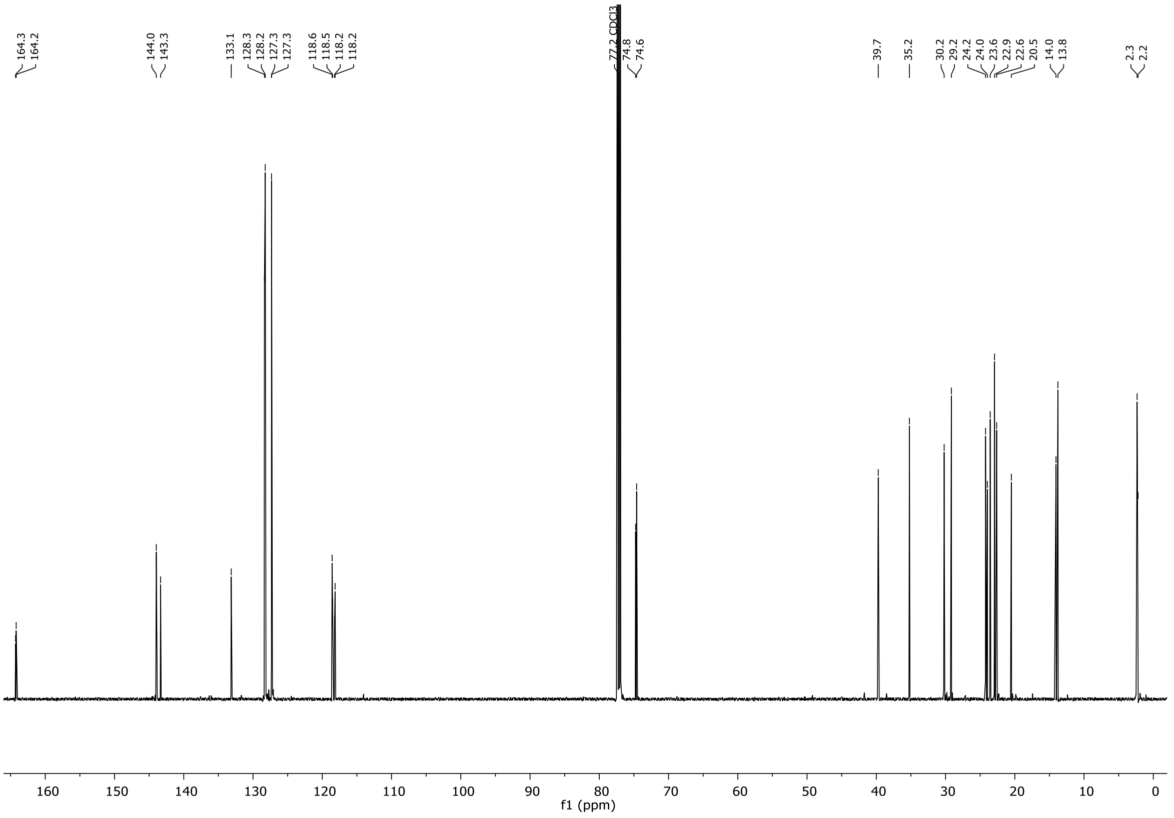

(*Z*)-2-(1-Hydroxy-1,2,3,4-tetrahydronaphthalen-1-yl)-3-methylhex-2-enenitrile (10cq)

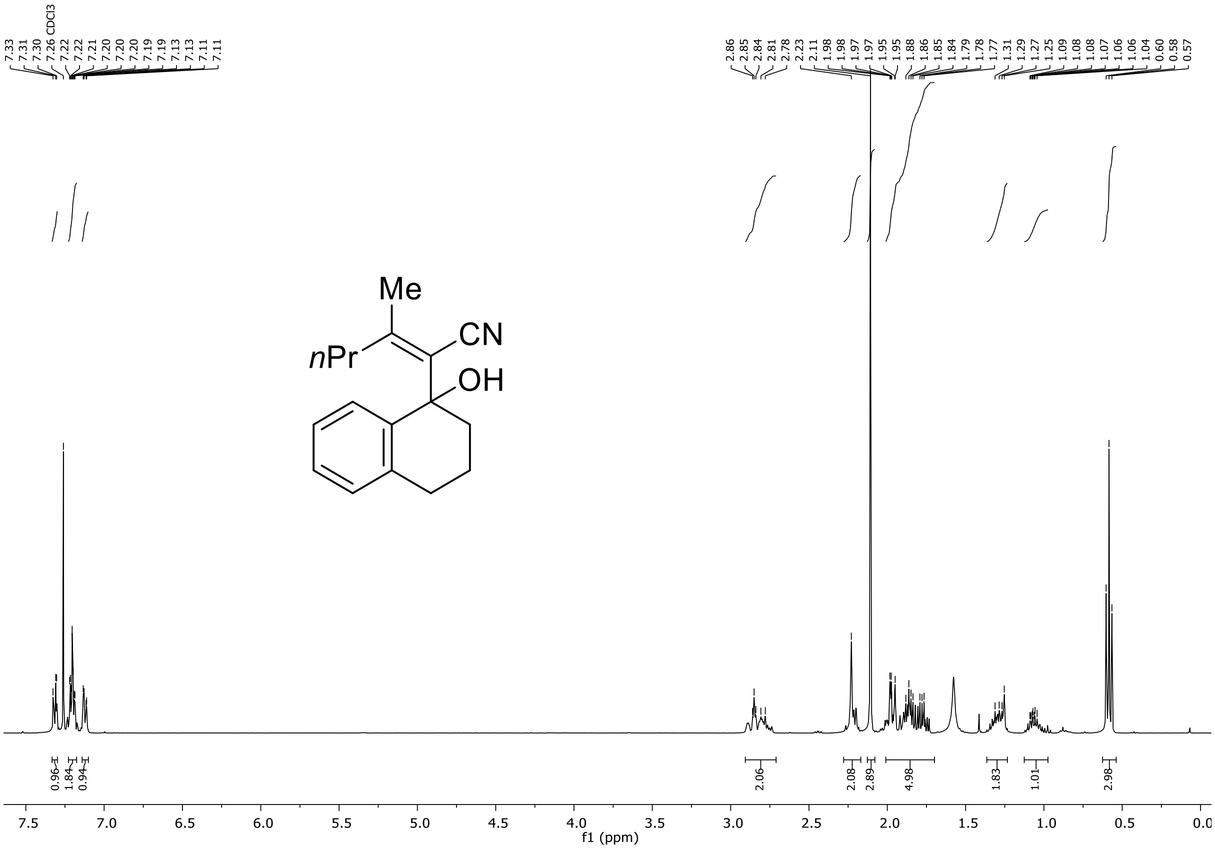

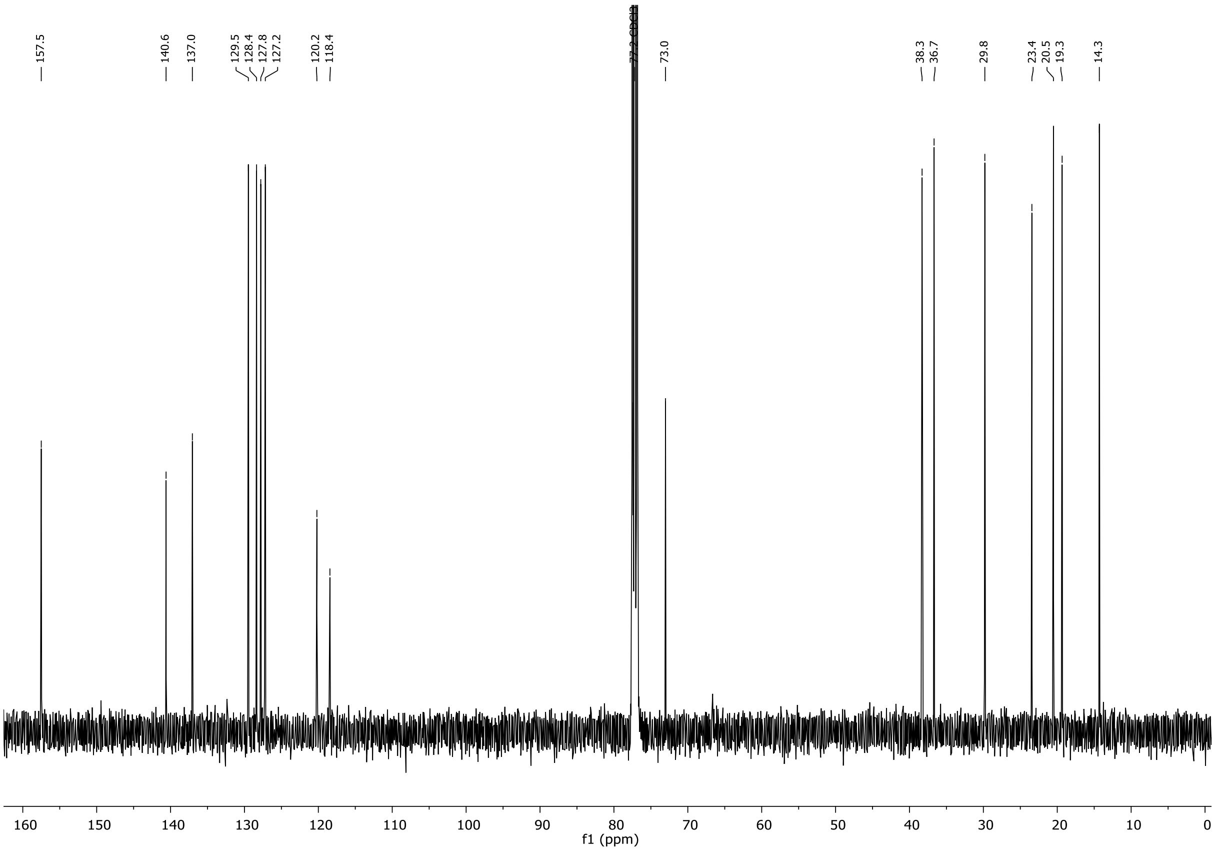

(*E*)-2-(1-Hydroxy-1,2,3,4-tetrahydronaphthalen-1-yl)-3-methylhex-2-enenitrile (10cq)

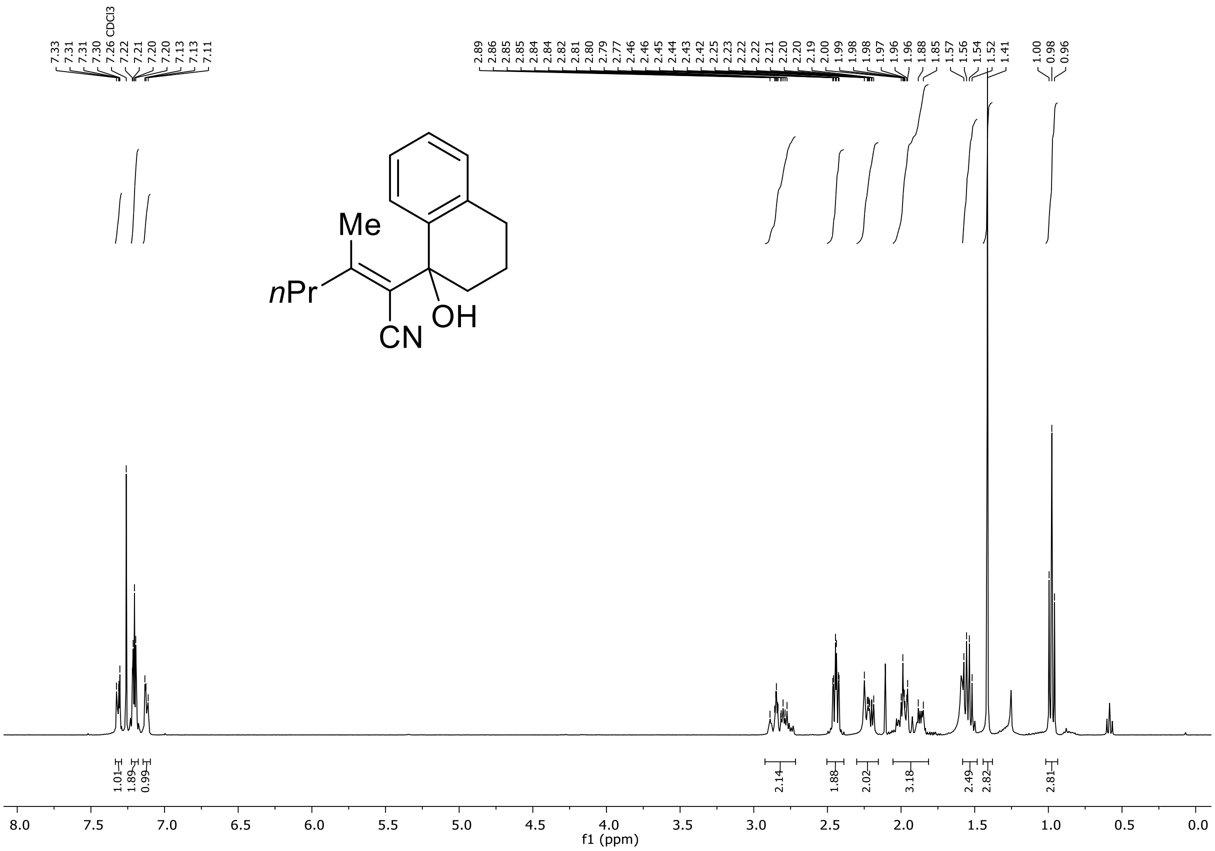

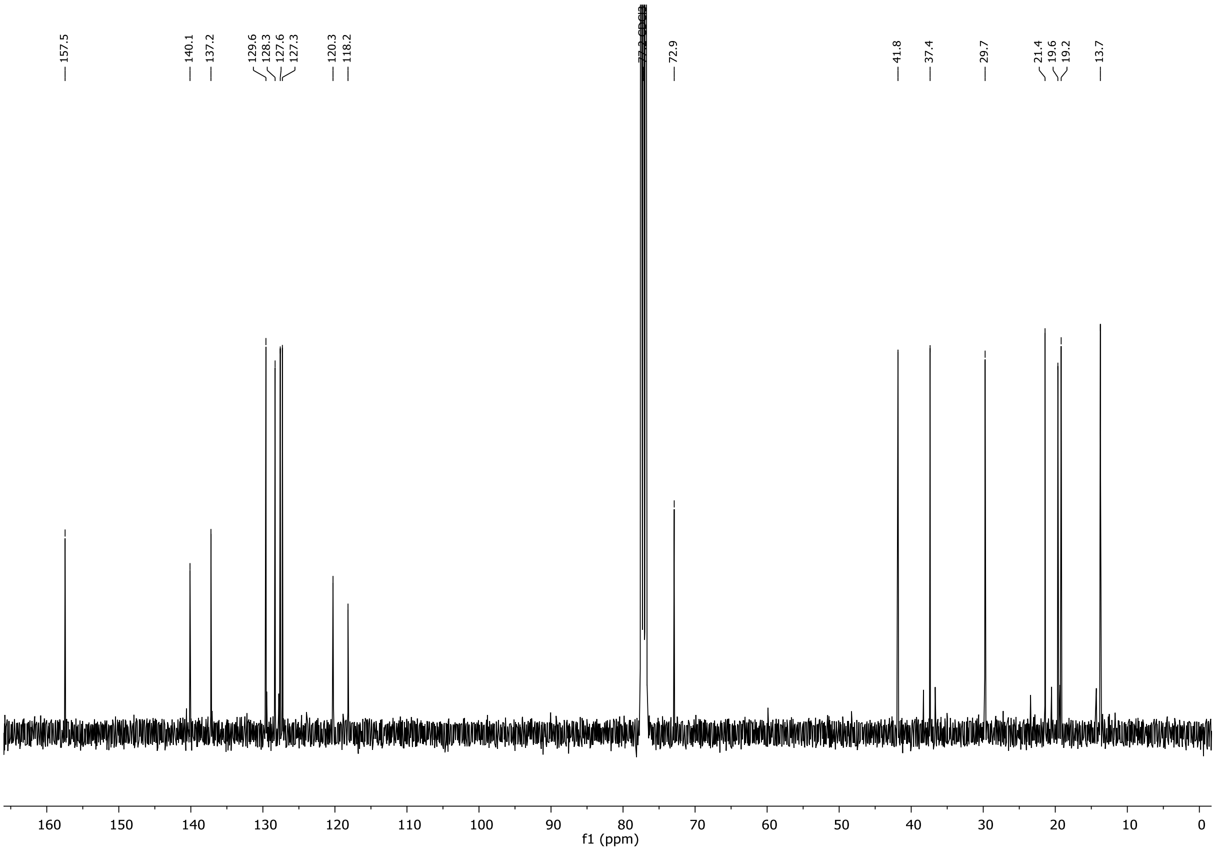

(*Z*)-2-(hydroxy(2-methoxyphenyl)methyl)-4-methoxy-3-methylbut-2-enenitrile (10dk)

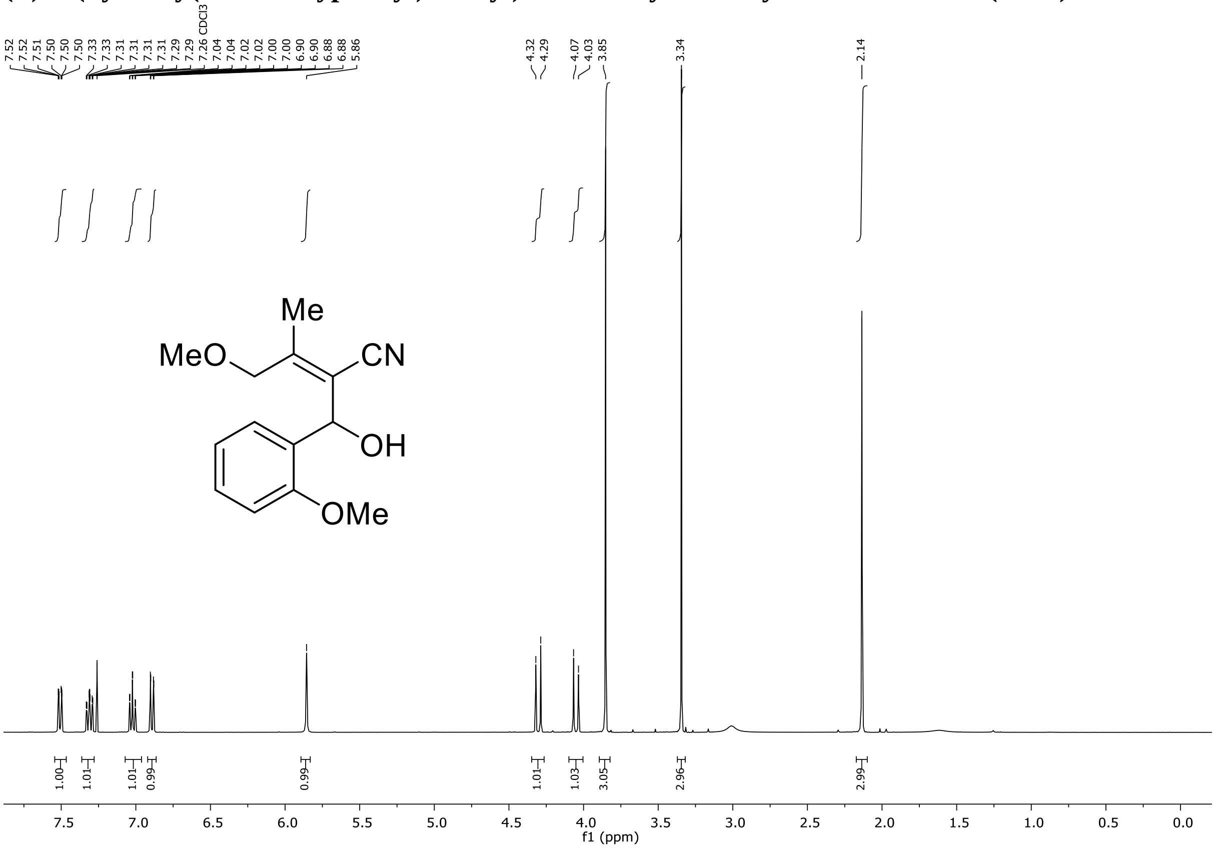

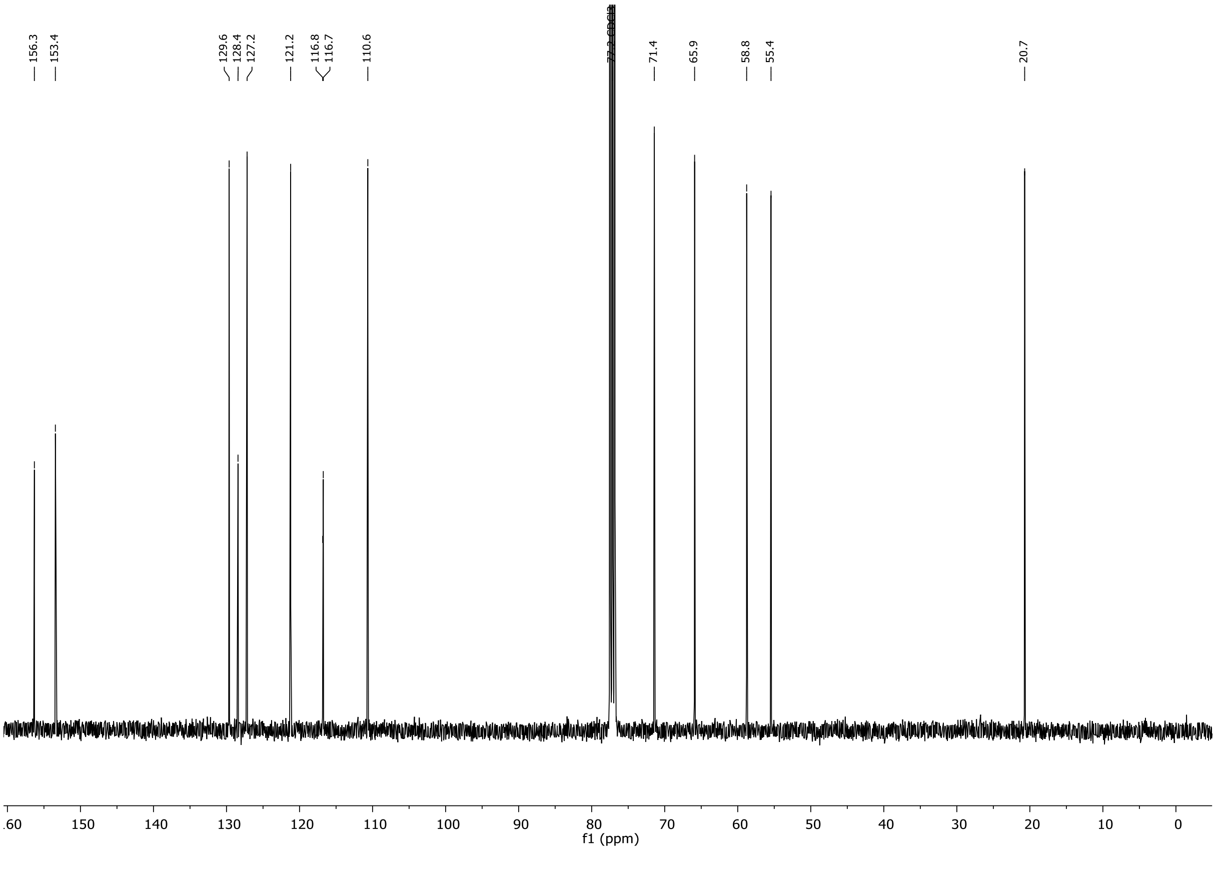

2-(Cyclohex-2-en-1-yl)nona-2,4-dienenitrileenenitrile (10ee)

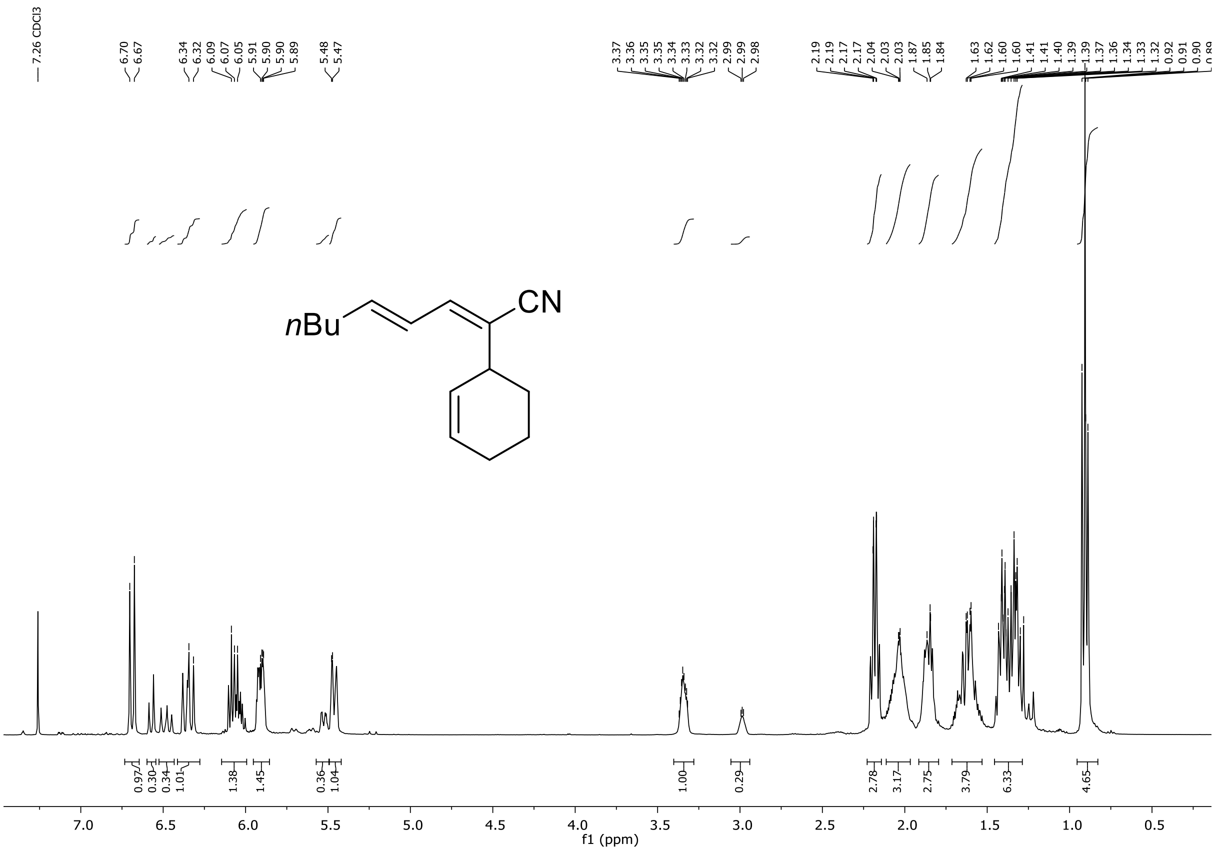

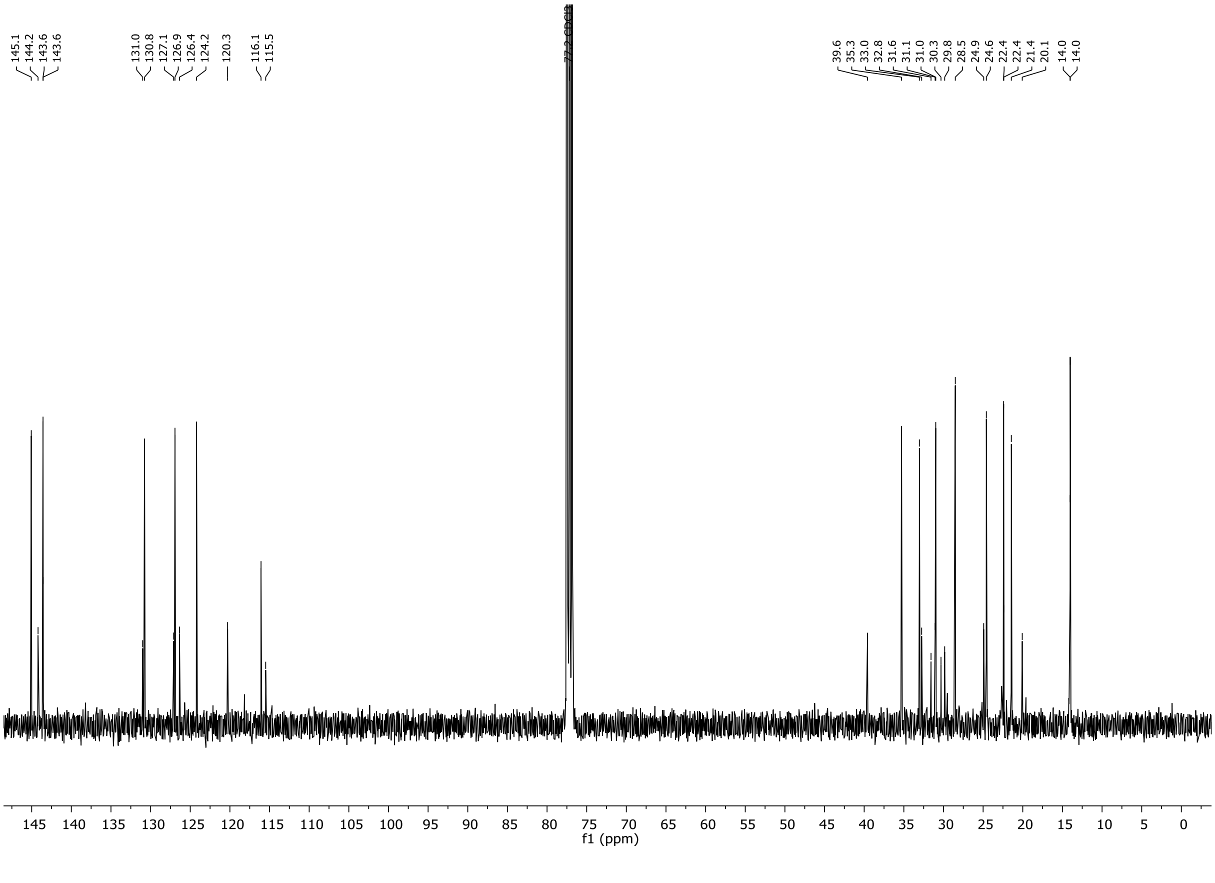

(2Z,4E)-2-(Hydroxy(2-methoxyphenyl)methyl)nona-2,4-dienenitrile (10ek)

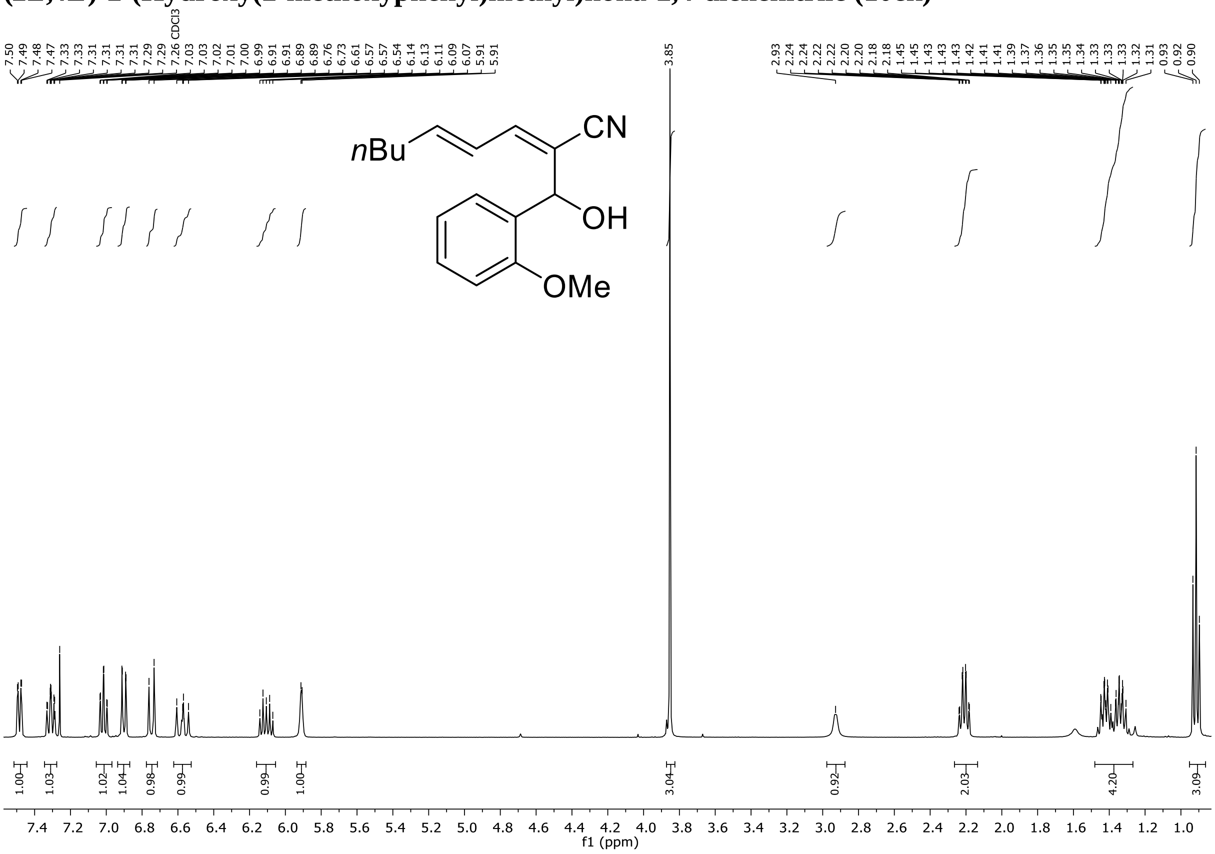

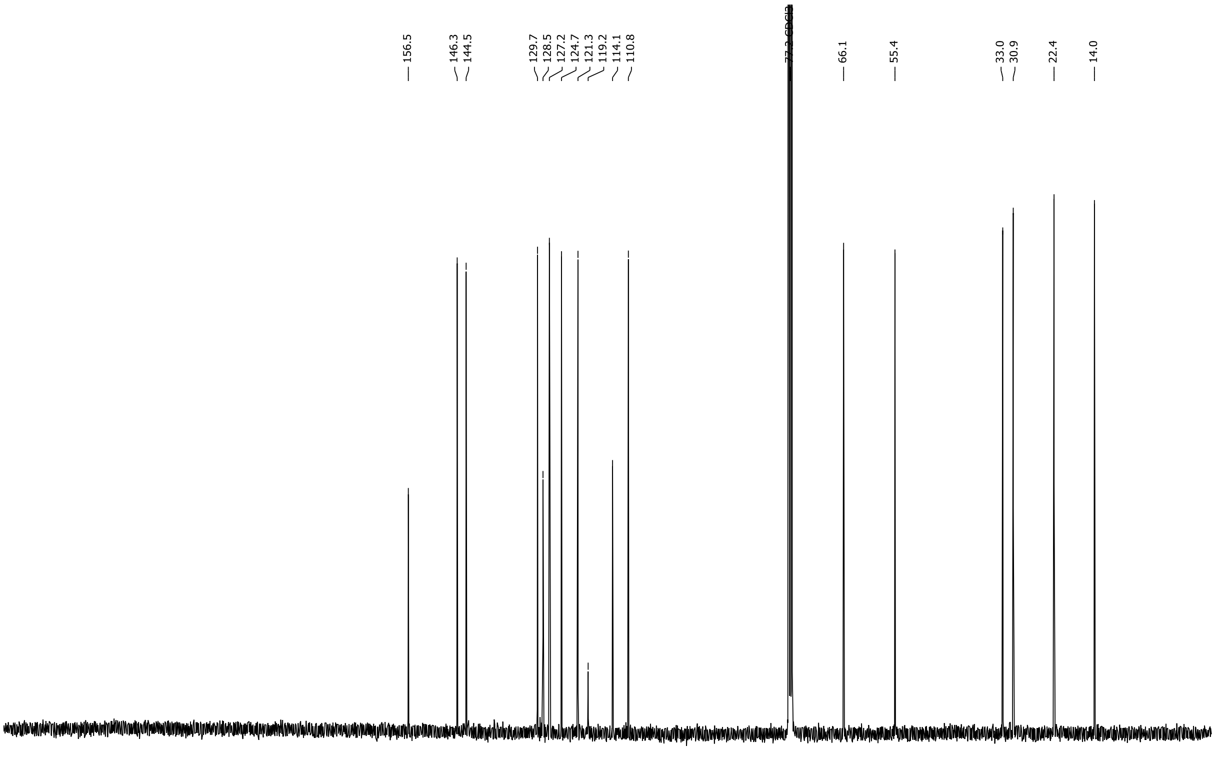

(2*E*,4*E*)-2-(Hydroxy(2-methoxyphenyl)methyl)nona-2,4-dienenitrile (10ek)

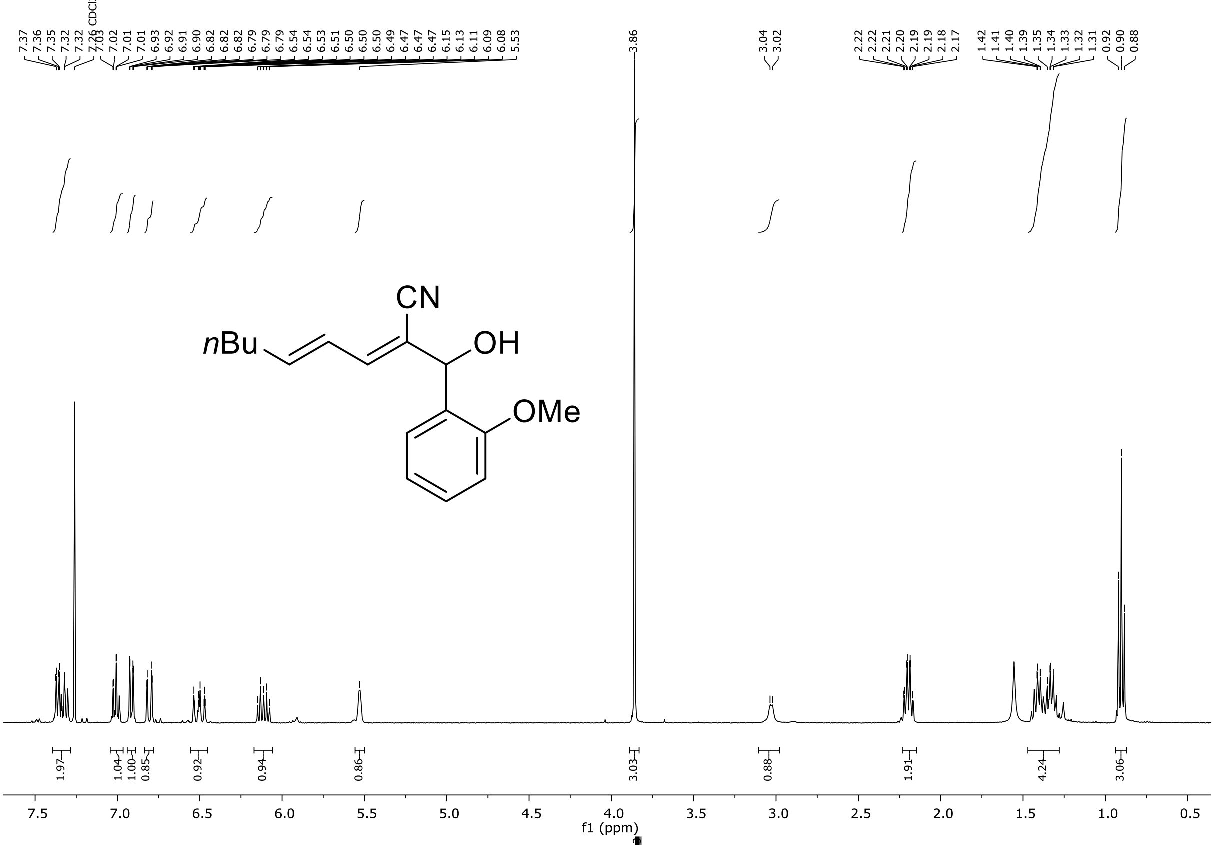

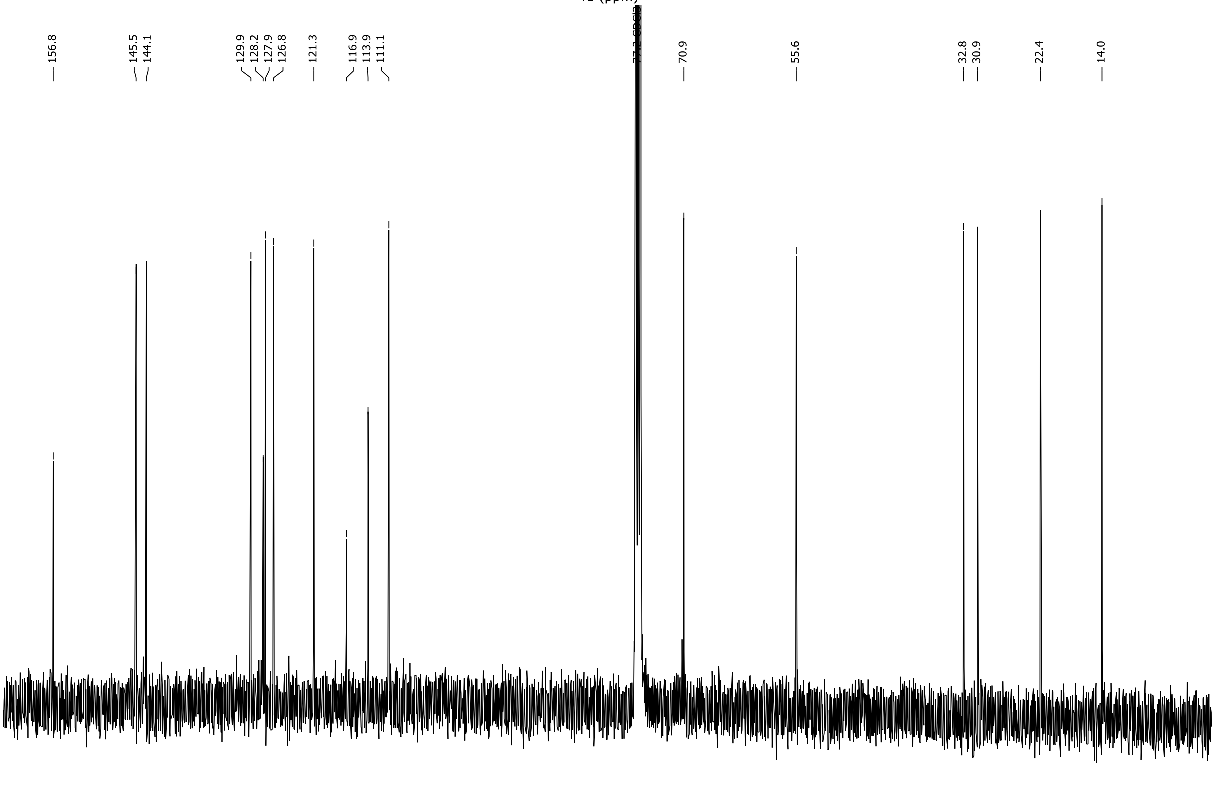

NaTMP:

(*E*)-2-(Hydroxyadamantan-2-yl)-3-phenylacrylonitrile (4ao)

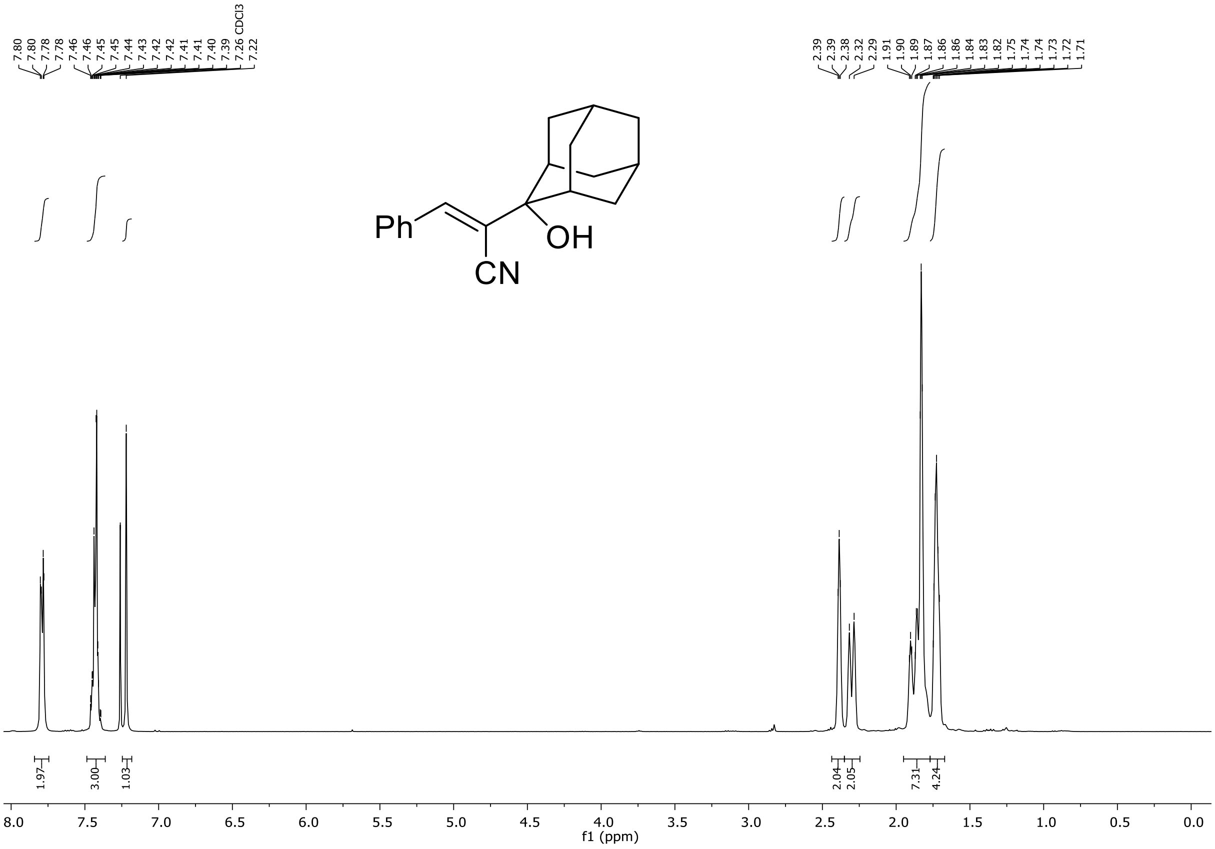

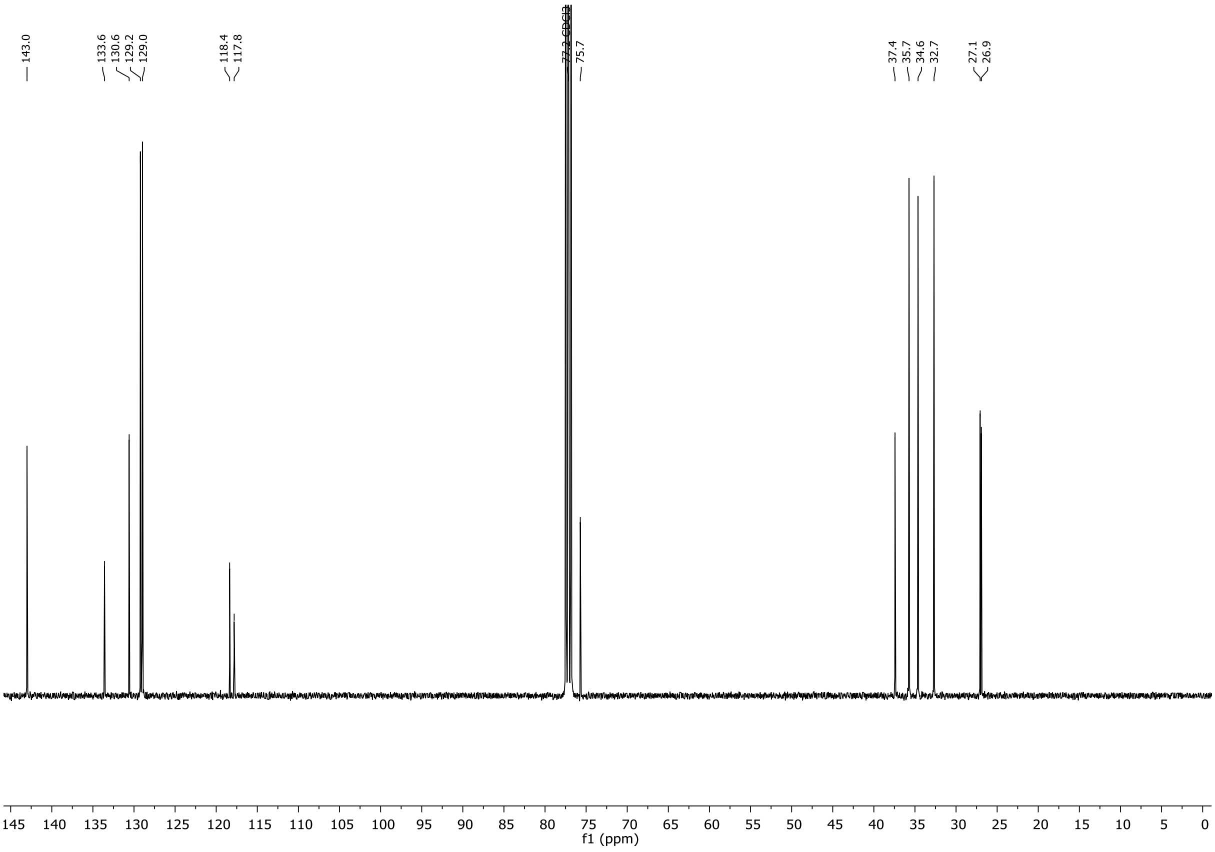

(*E*)-2-Benzylidene-3-hydroxy-3-phenylbutanenitrile (4as)

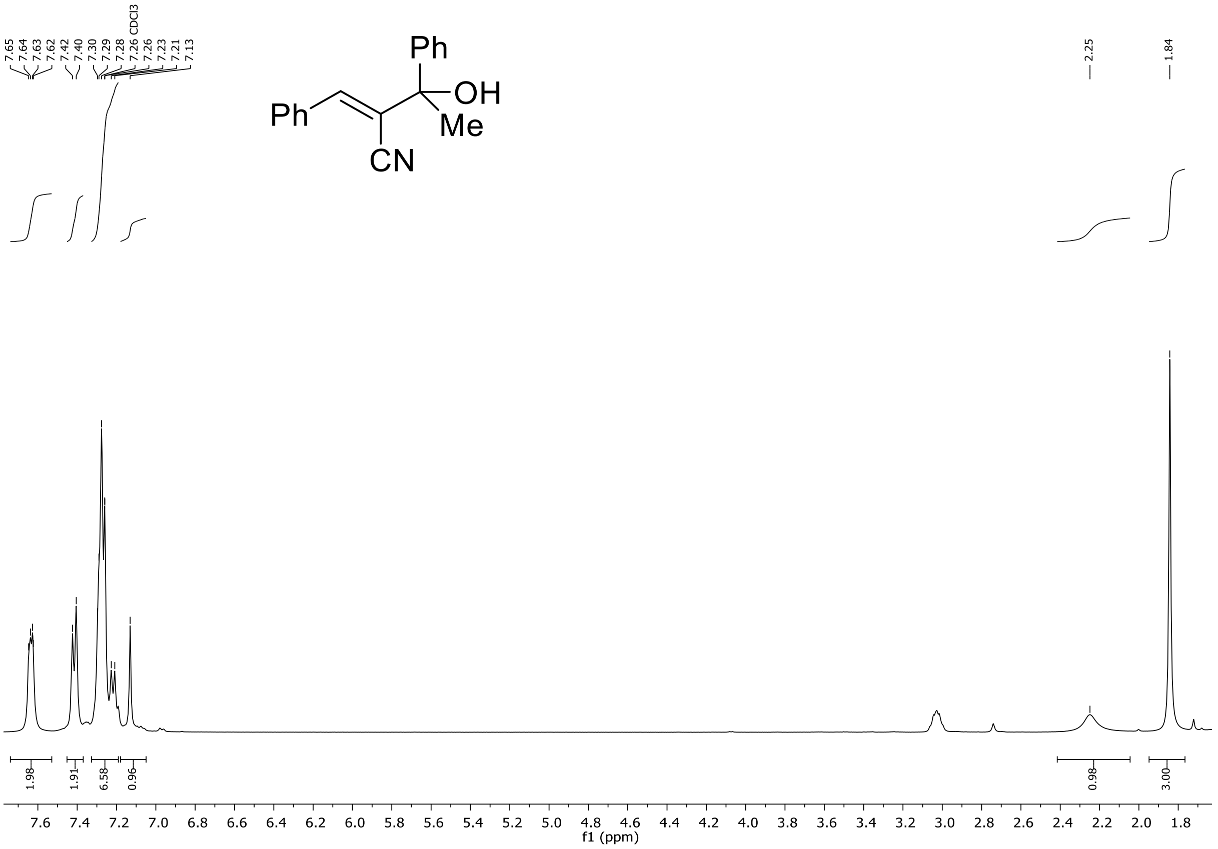

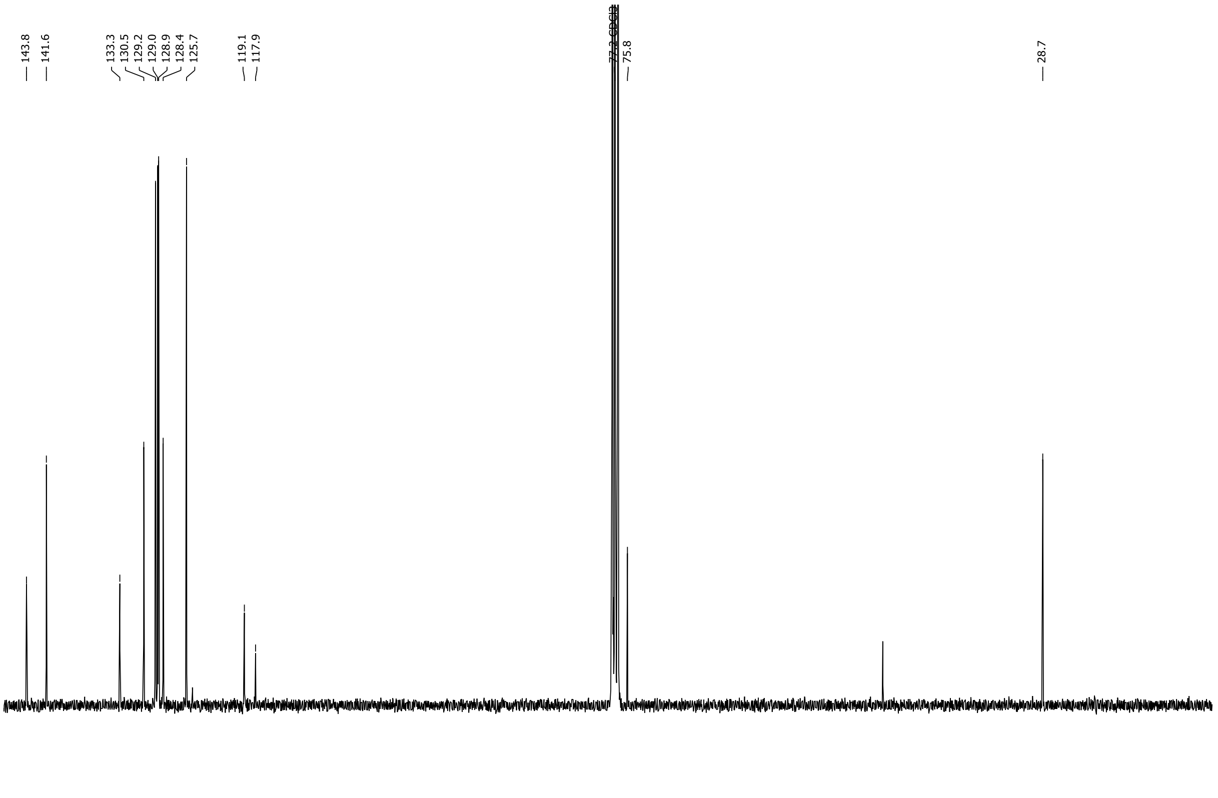

(*E*)-2-(1-Hydroxy-1,2,3,4-tetrahydronaphthalen-1-yl)-3-phenylacrylonitrile (4aq)

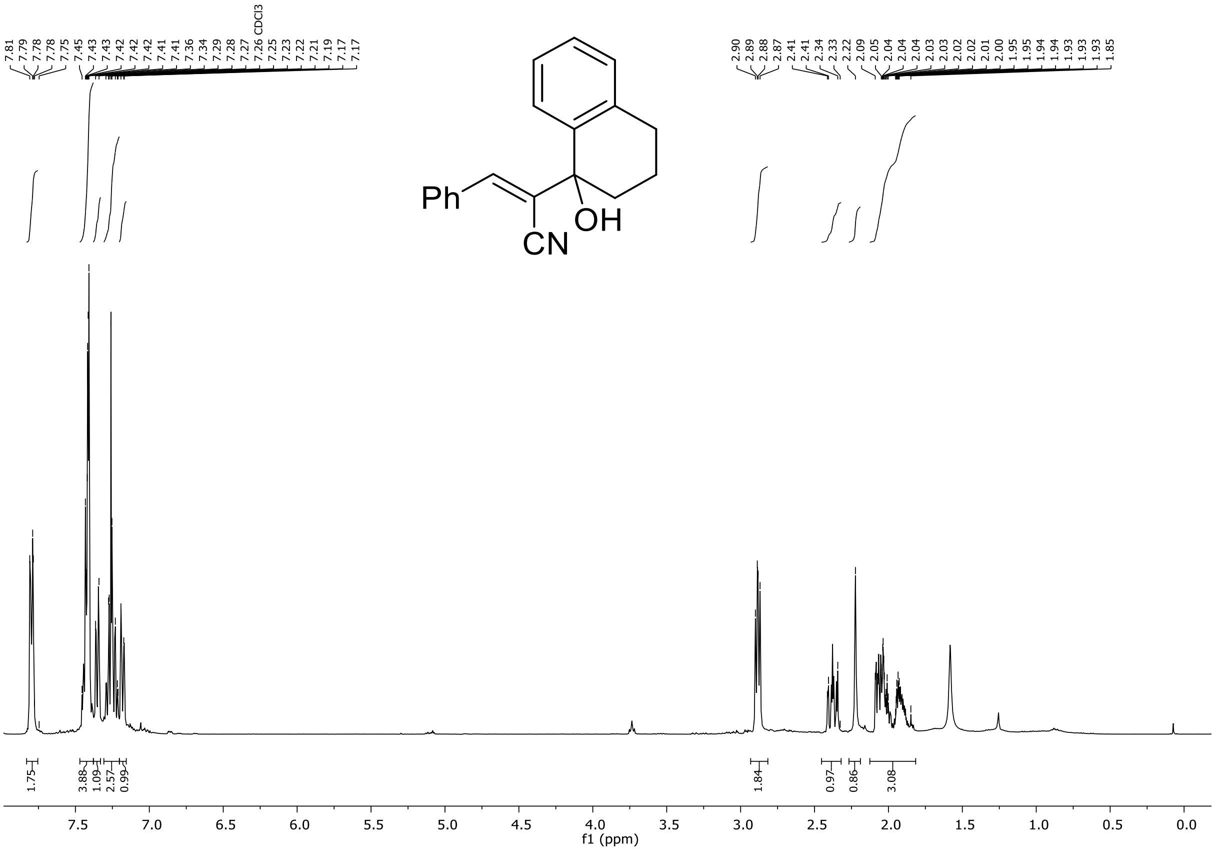

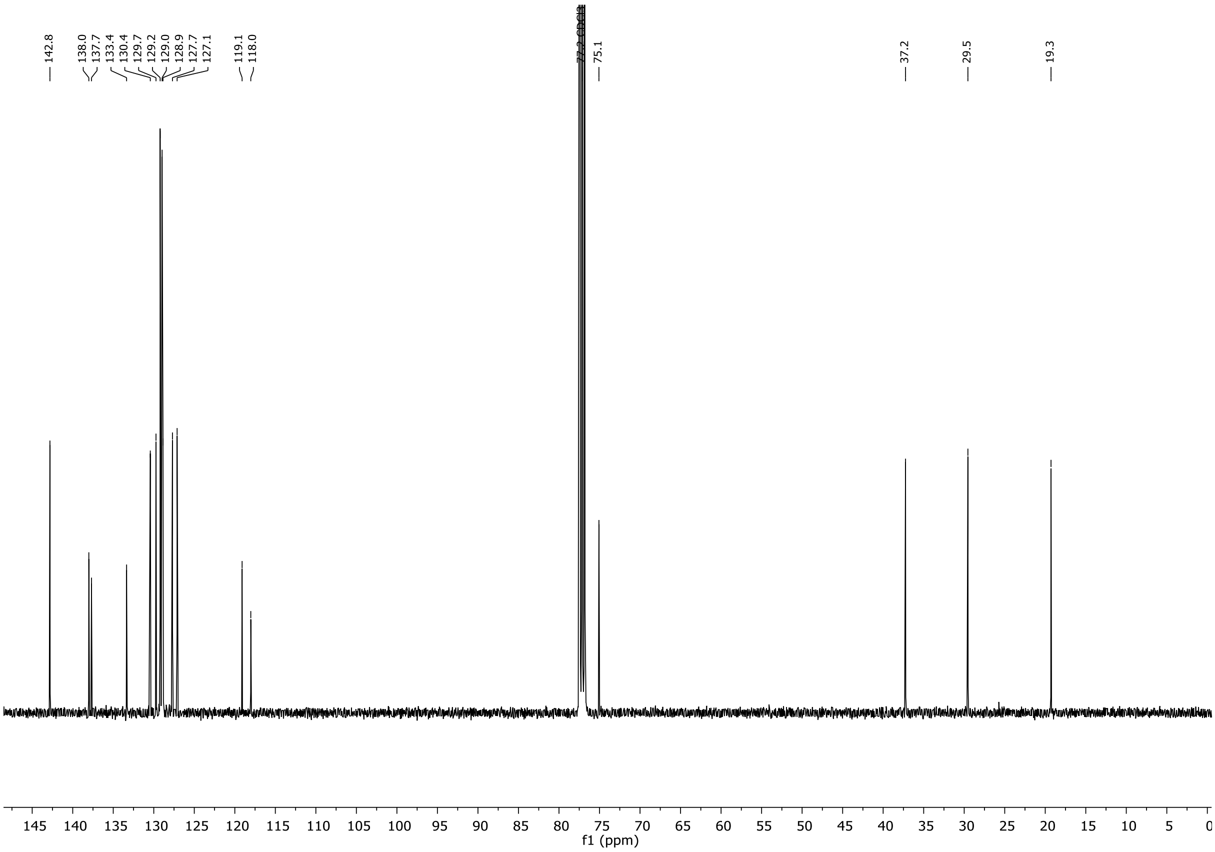

(*Z*)-3-Ethoxy-2-(hydroxy(2-methoxyphenyl)methyl)acrylonitrile (7gk)

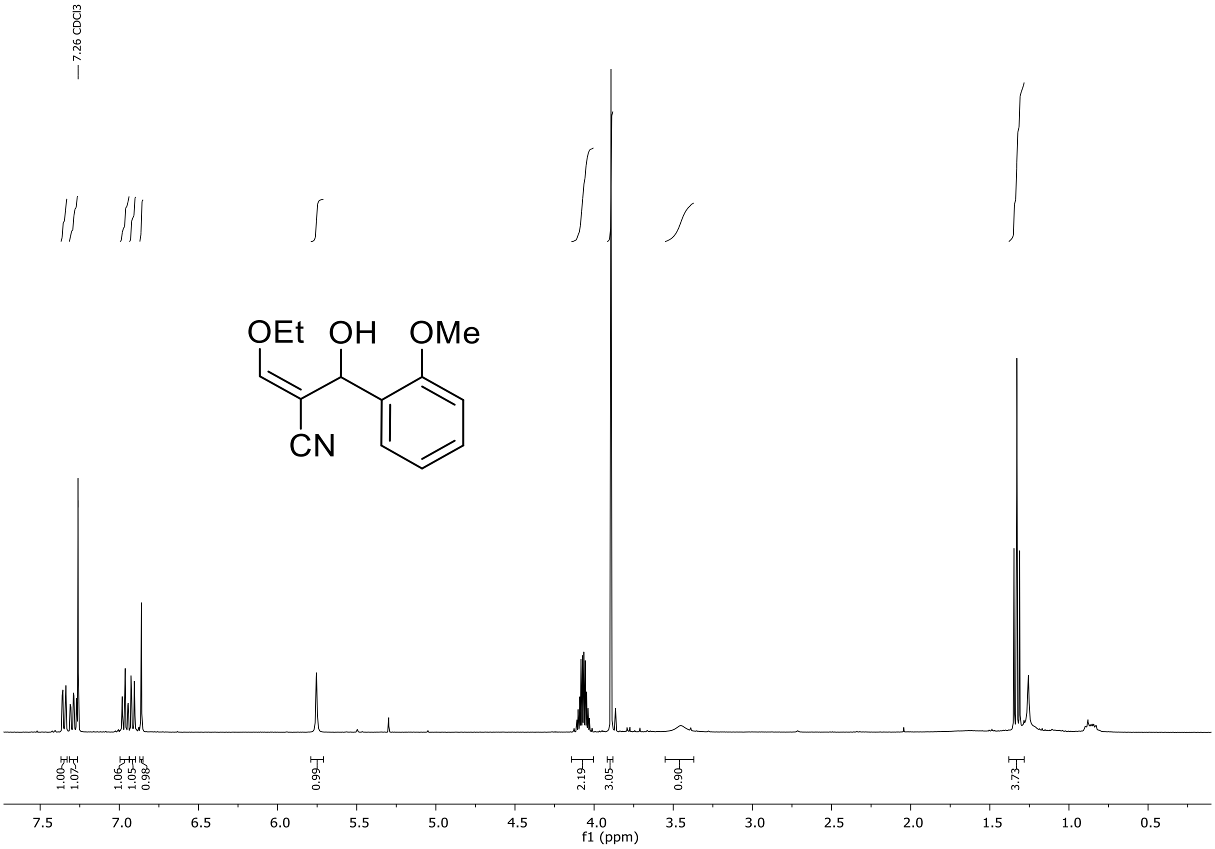

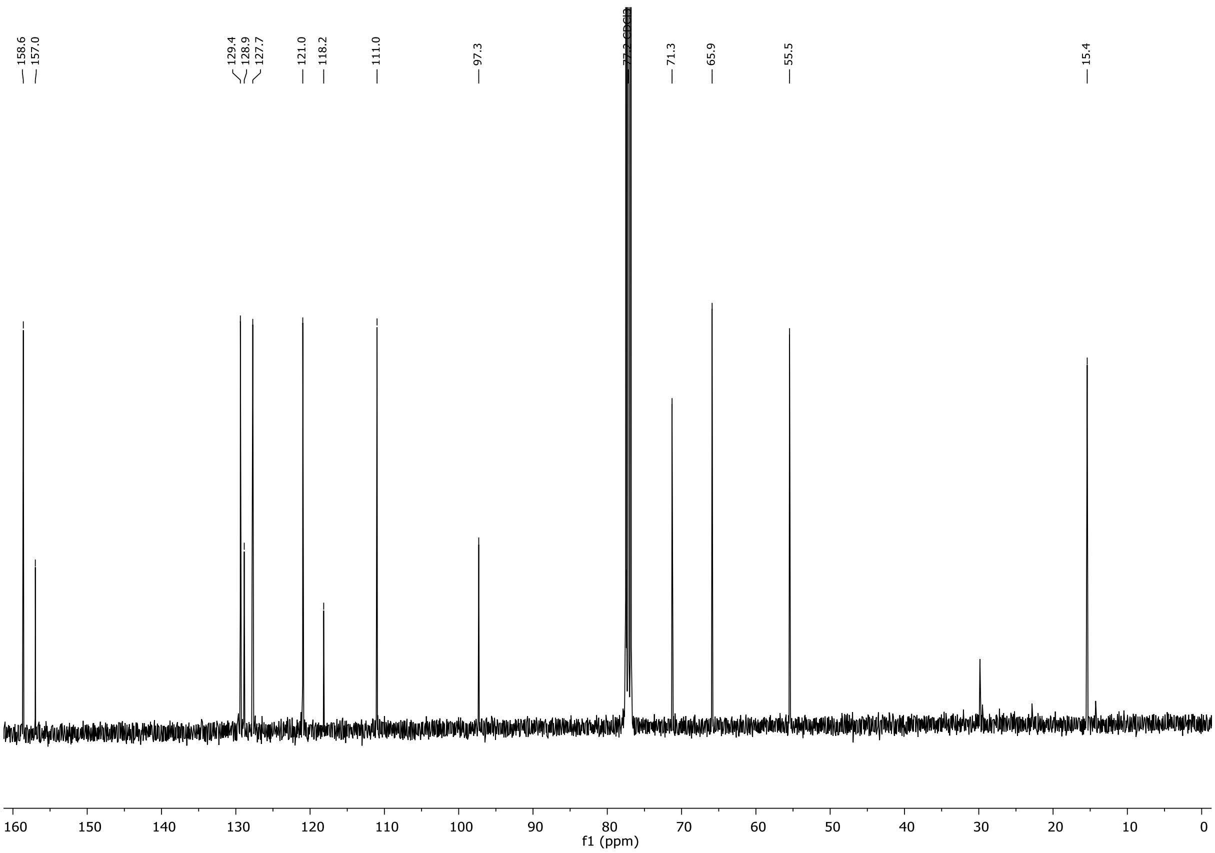

(*Z*)-2-(Hydroxydiphenylmethyl)-3-ethoxyacrylonitrile (7gg)

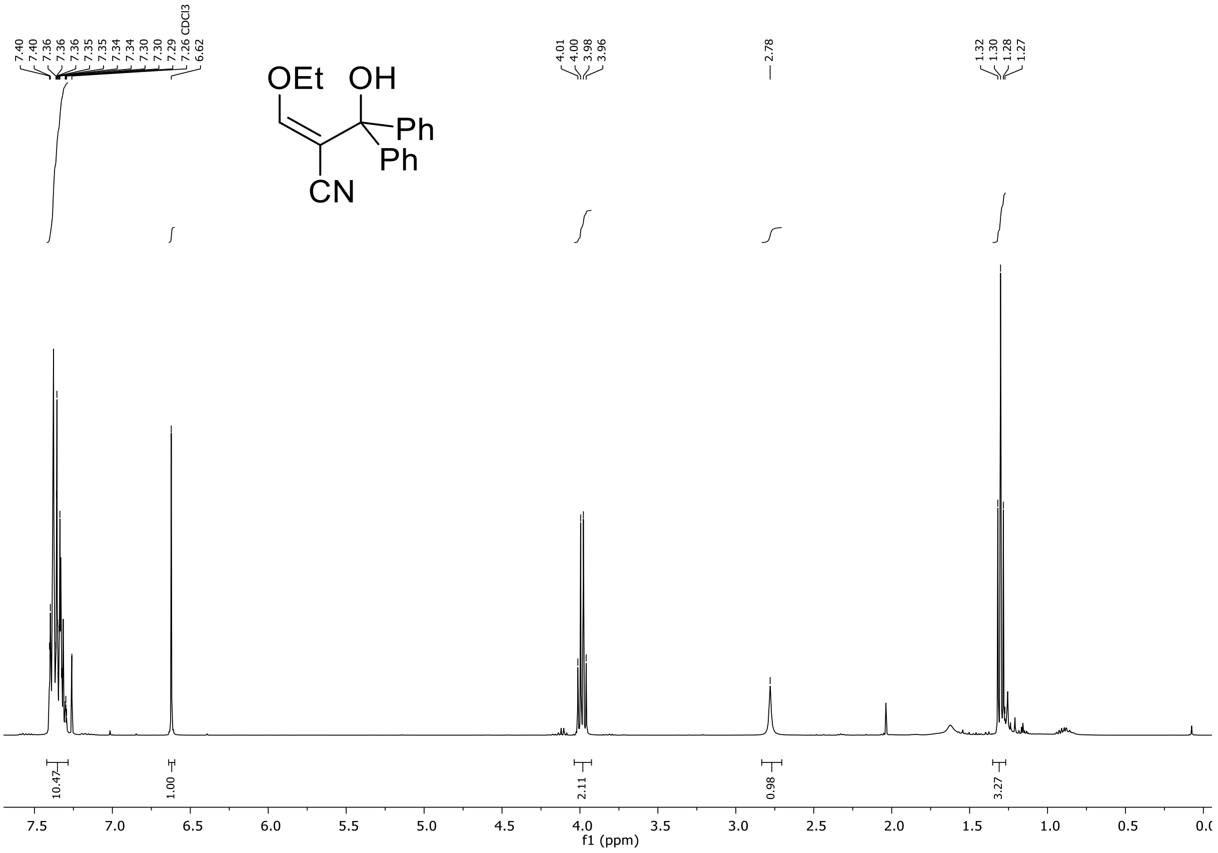

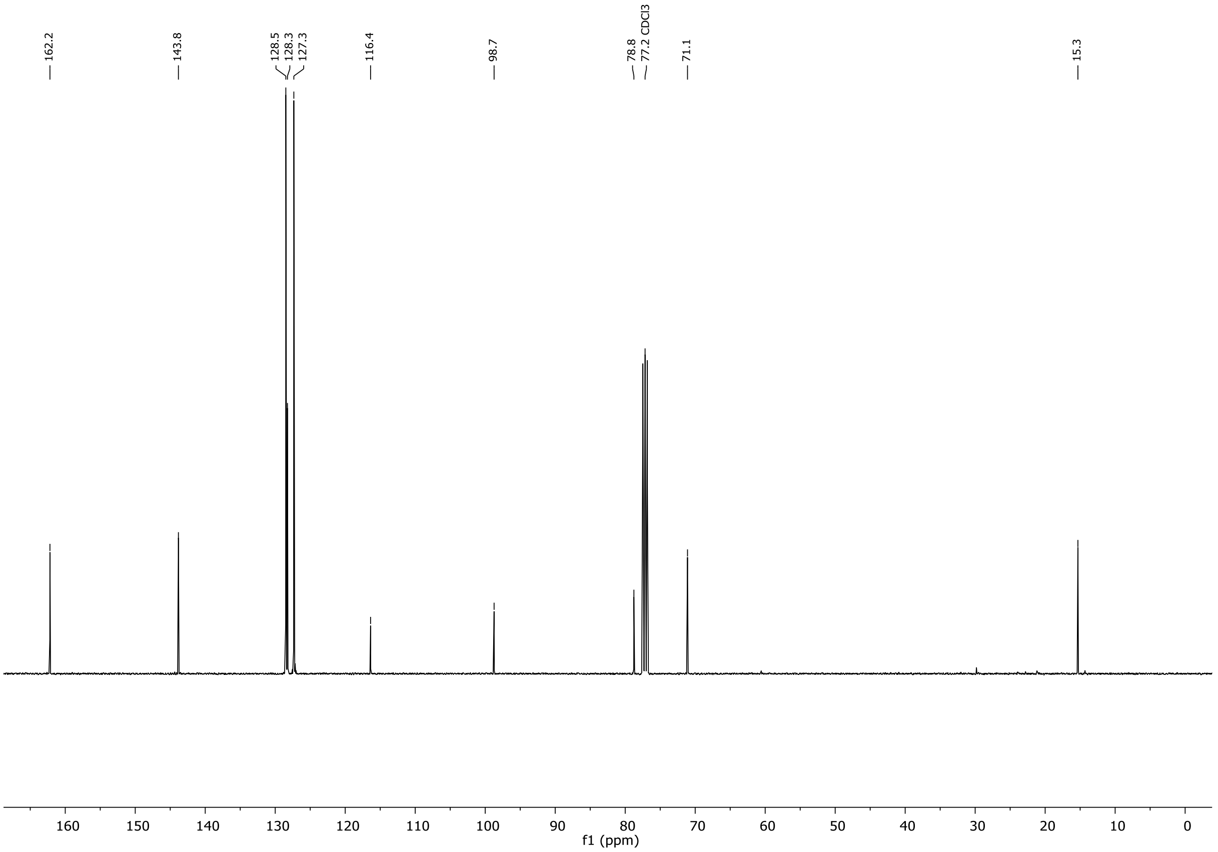

2-(Cyclohex-2-en-1-yl)-3,7-dimethylocta-2,6-dienenitrile (10ae)

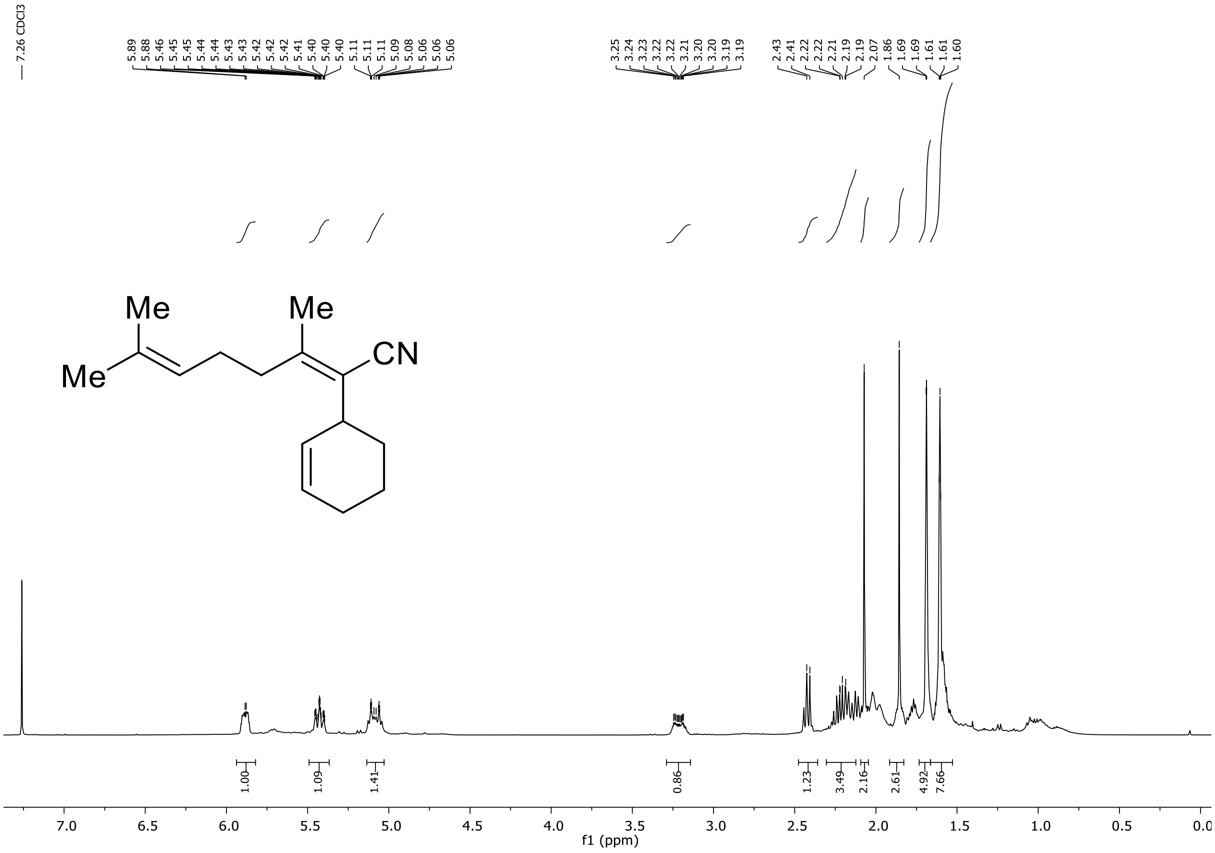

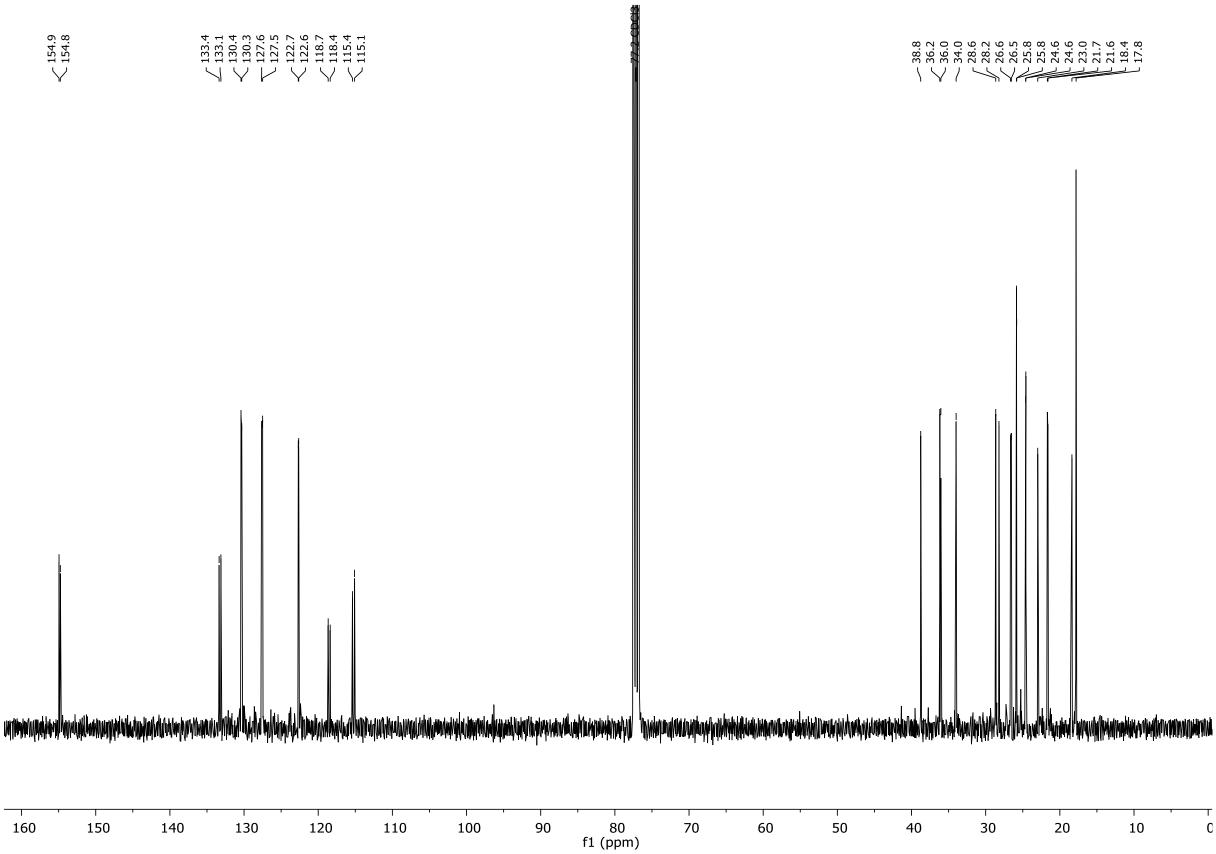

NaDA:

***Tert*-butyl (*E*)-2-(cyclohex-2-en-1-yl)-3-phenylacrylate (13be)**

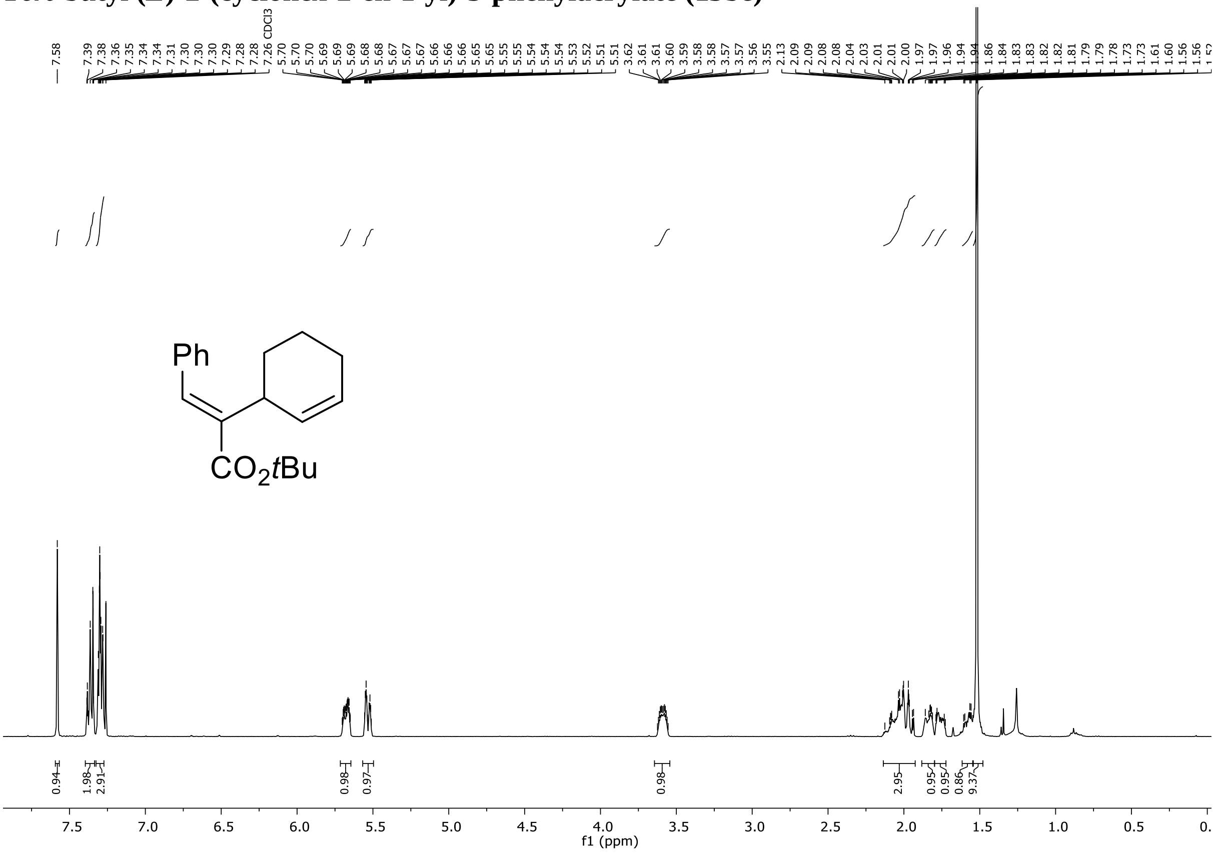

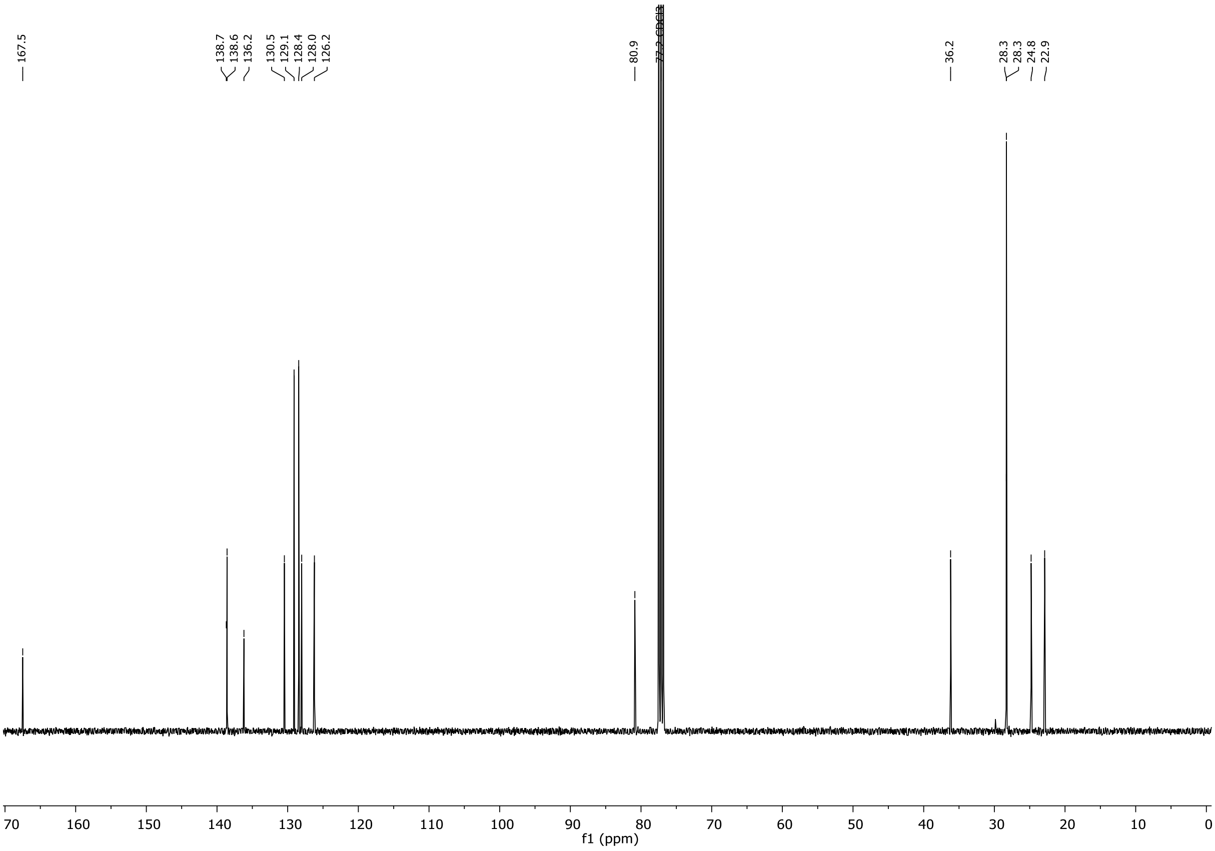

Ethyl (*E*)-2-(2-hydroxyadamantan-2-yl)-3-phenylacrylate (13ao)

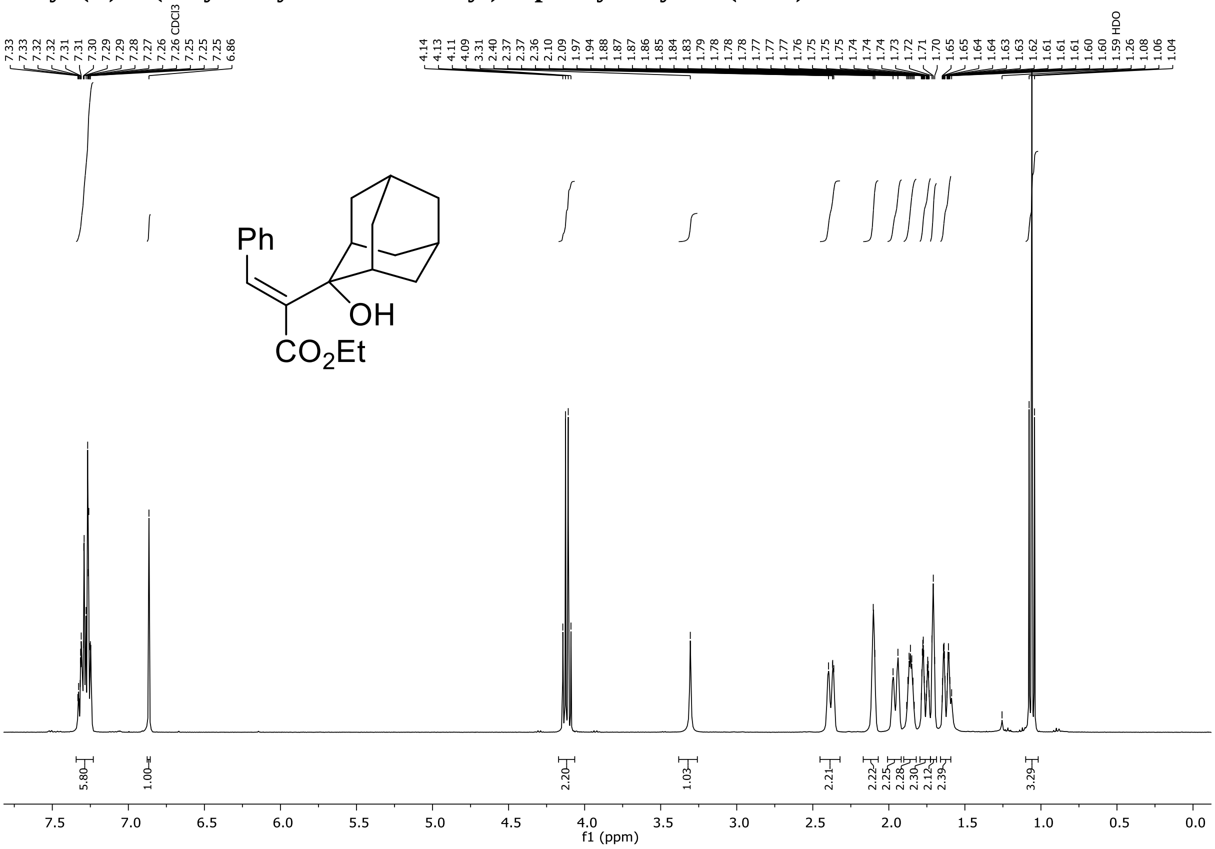

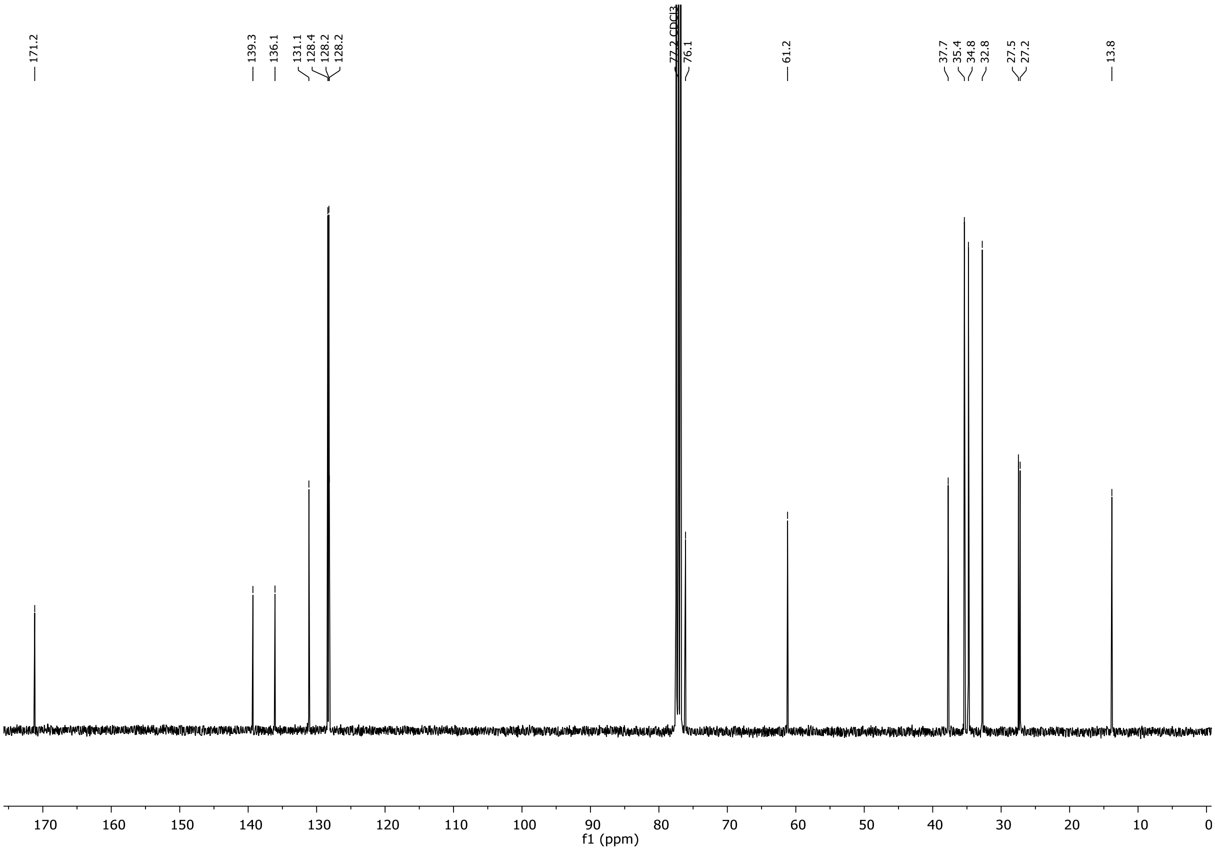

3'-Methoxy-5'*H*-spiro[adamantane-2,2'-furan]-5'-one (13co)

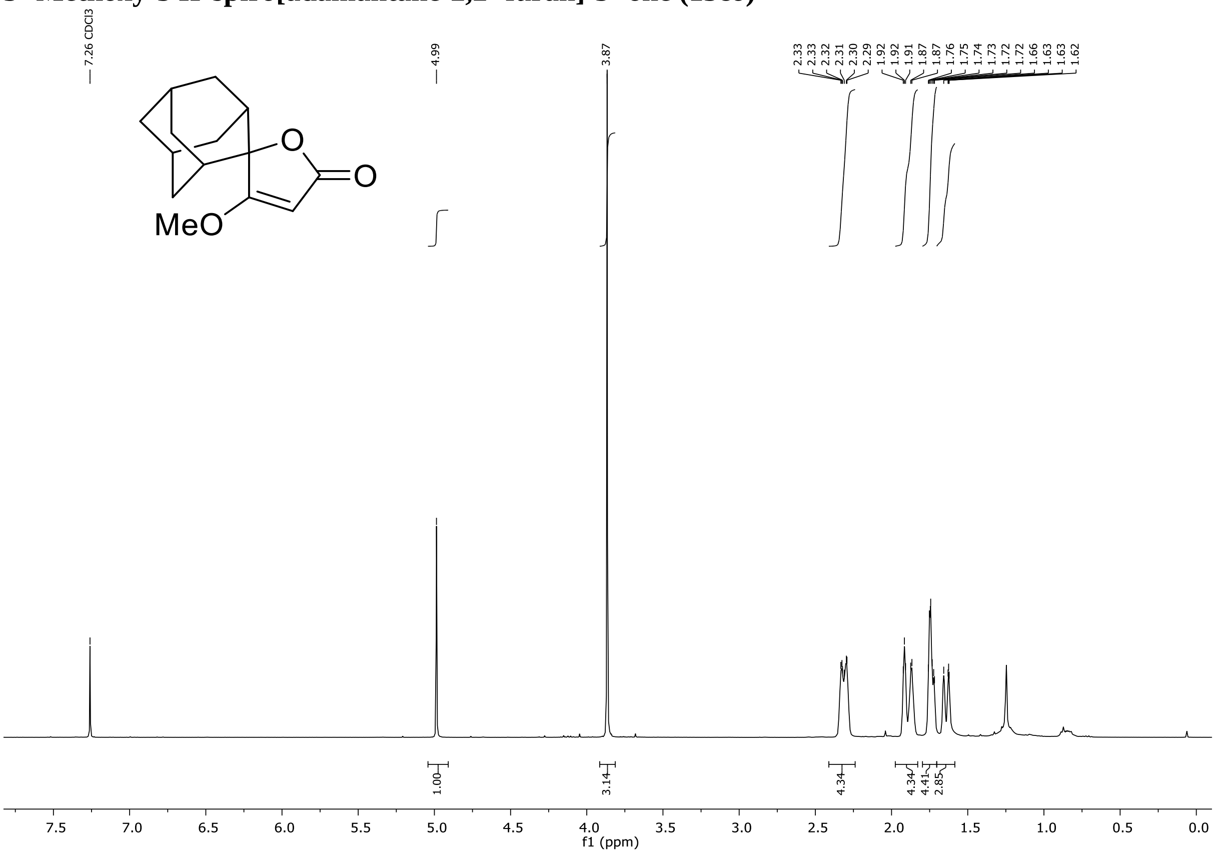

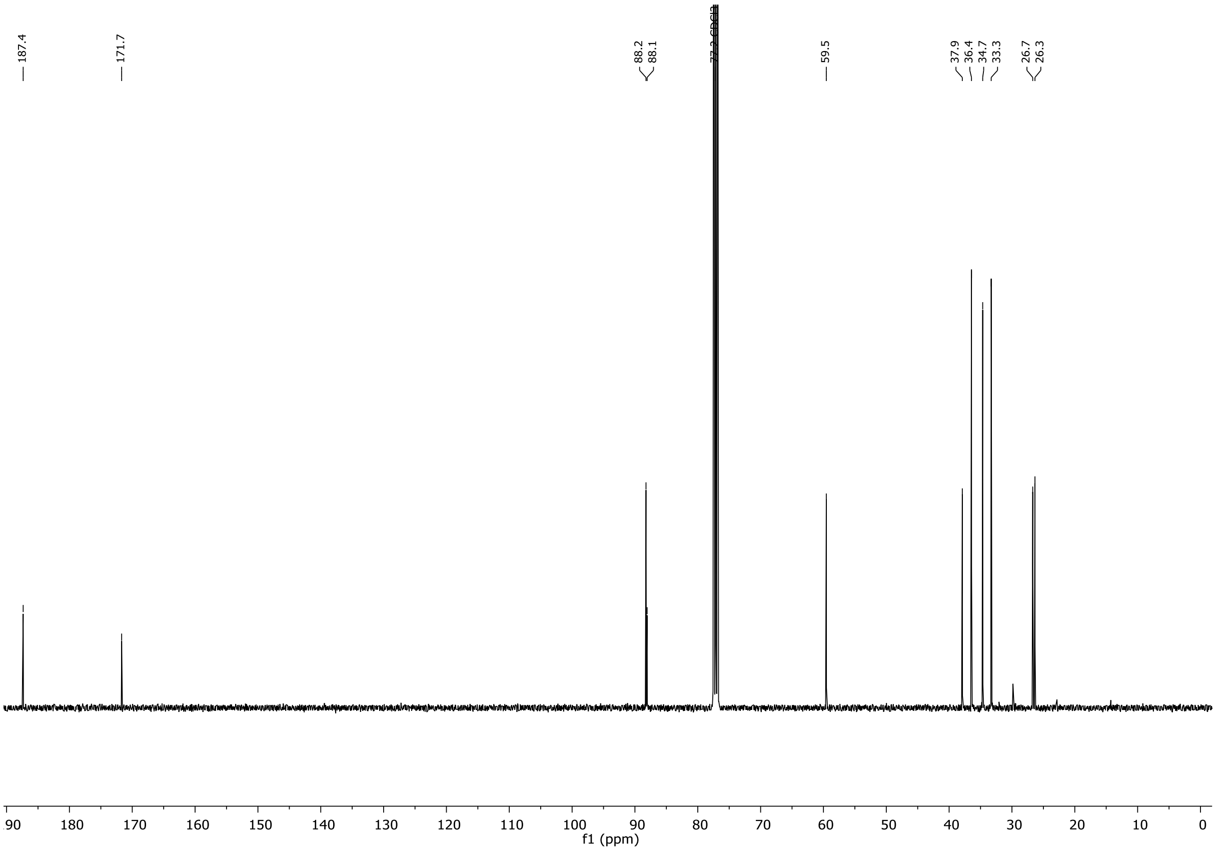

Starting material

3-(4-Methoxyphenyl)acrylonitrile (5a)

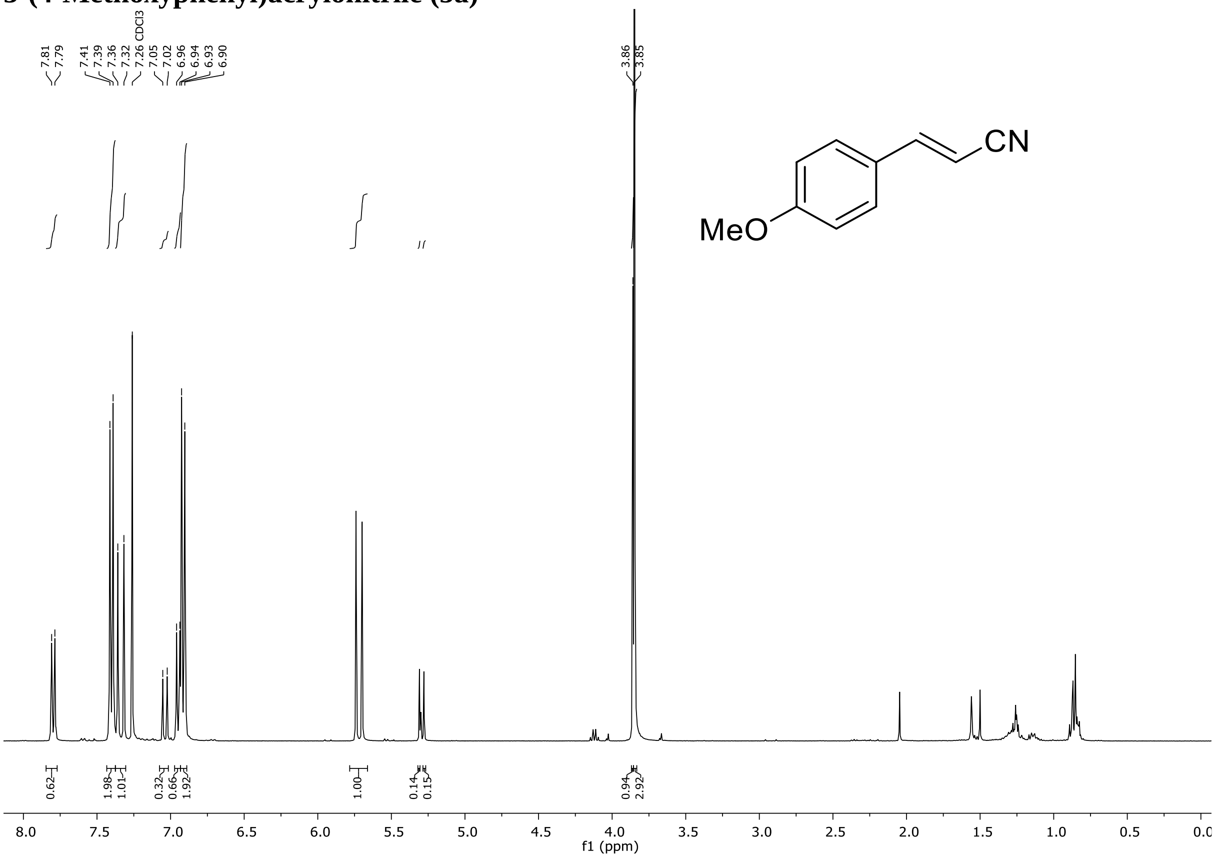

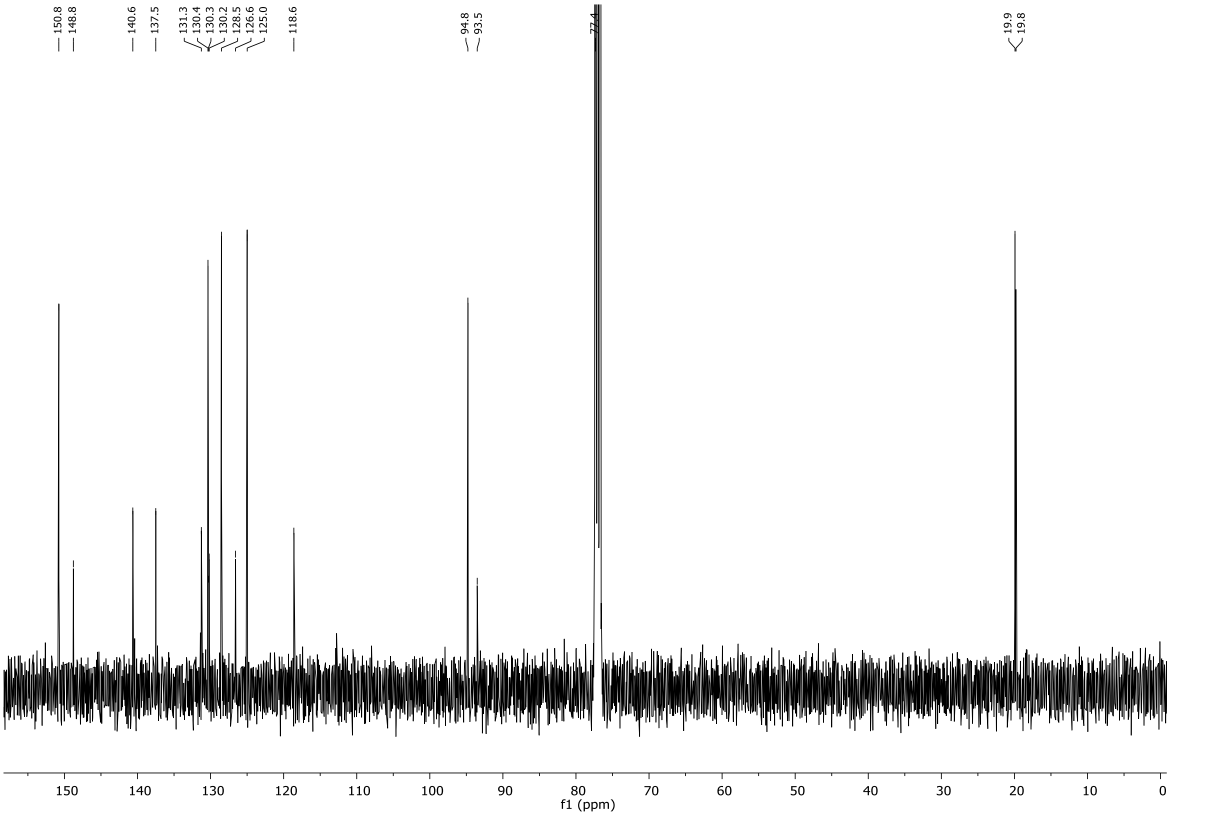

3-(3,4-Dimethylphenyl)acrylonitrile (5b)

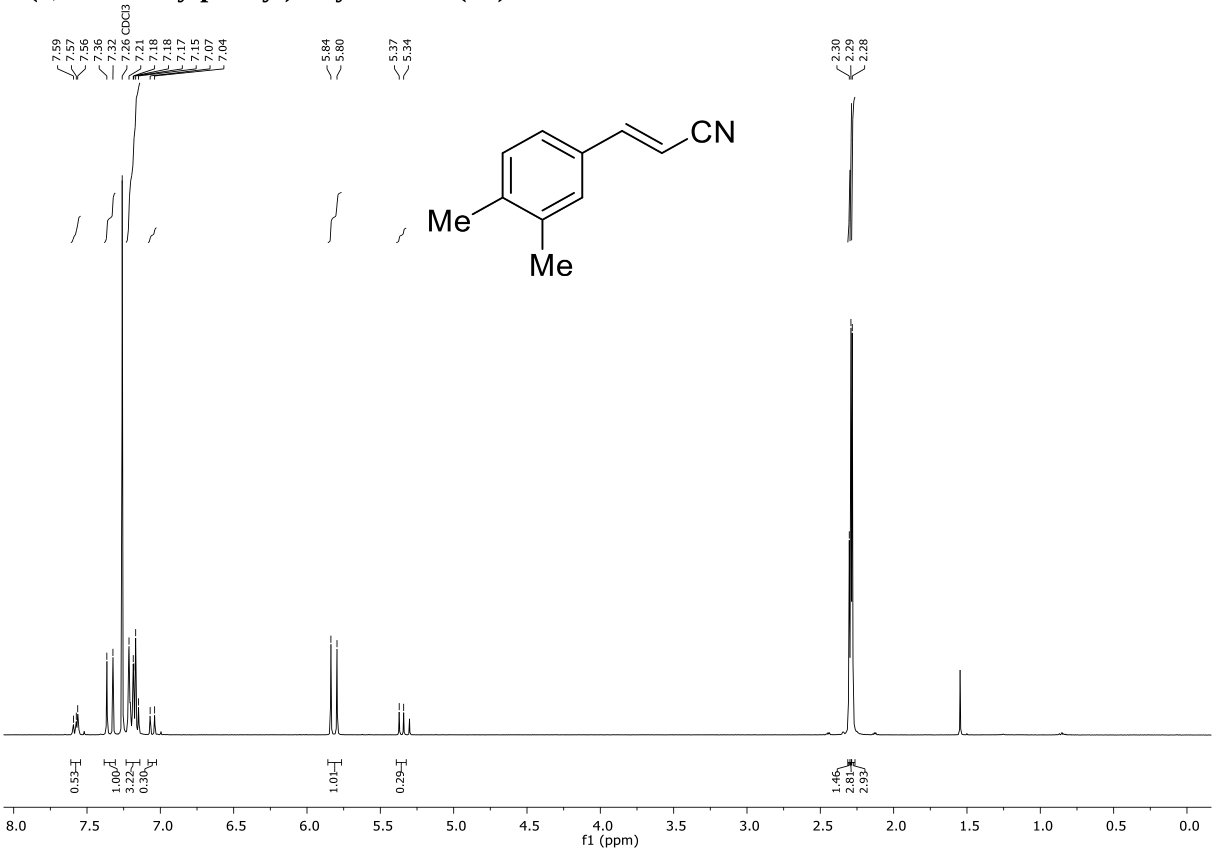

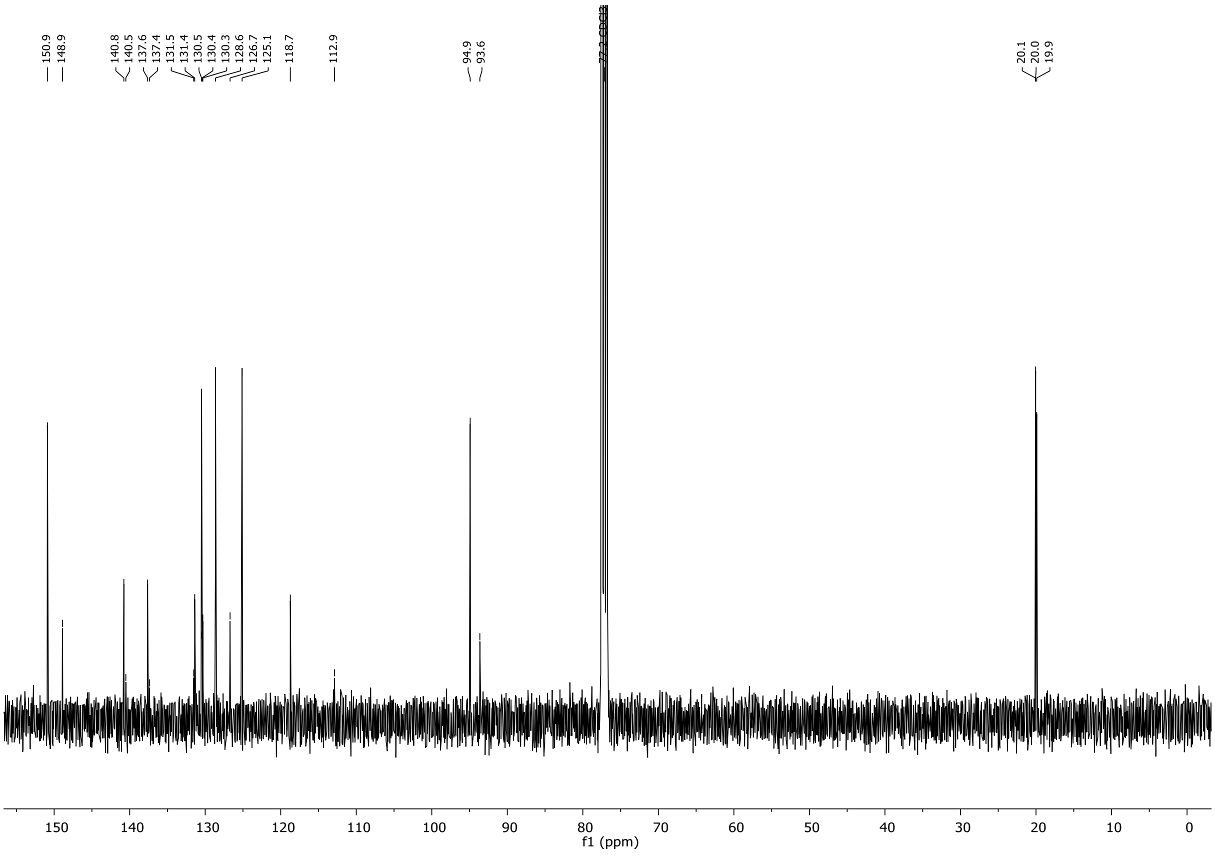

3-(2,3-Dihydrobenzo[*b*][1,4]dioxin-6-yl)acrylonitrile (5c)

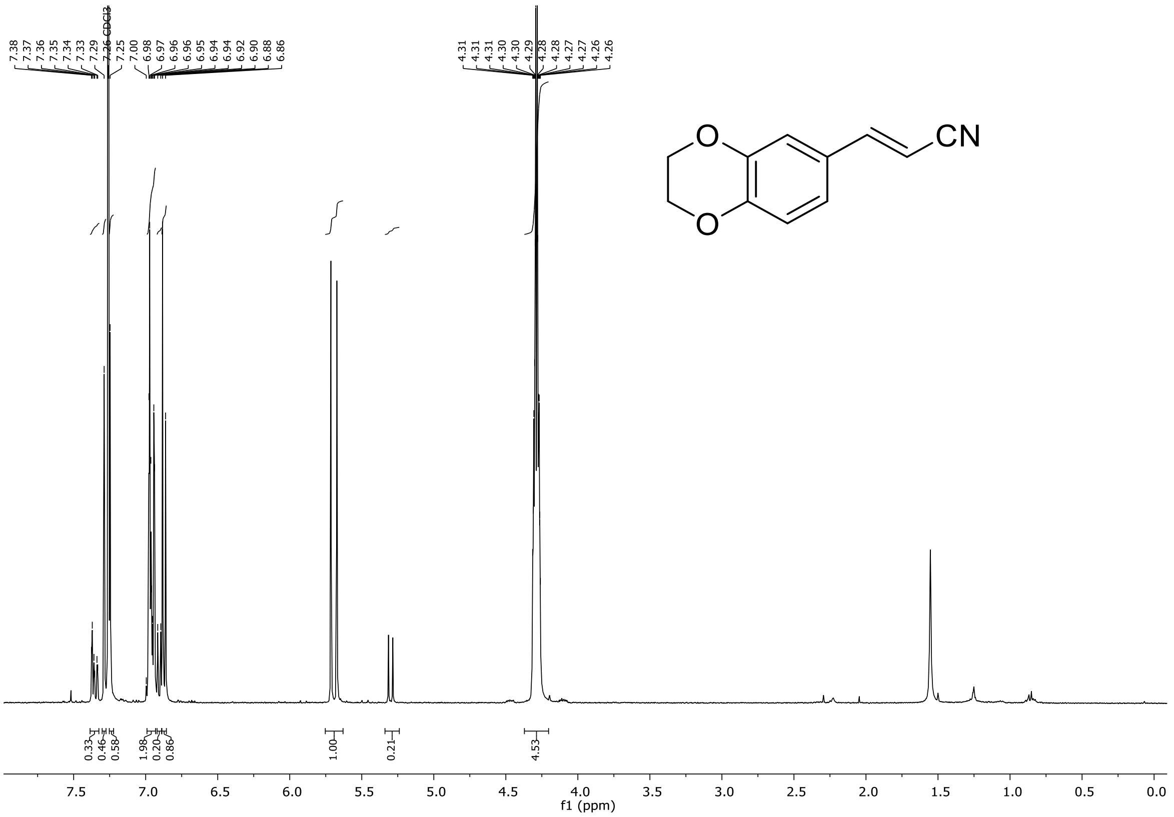

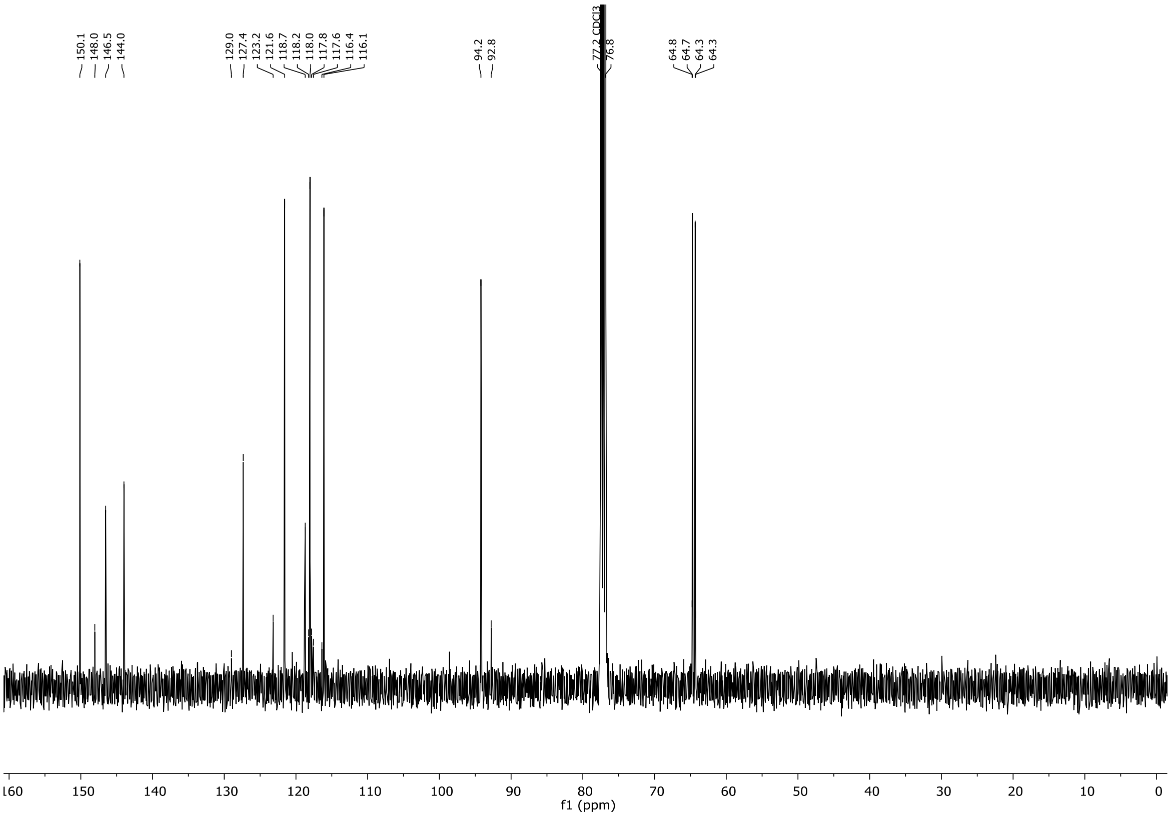

3-(4-(*Tert*-butyl)phenyl)acrylonitrile (5d)

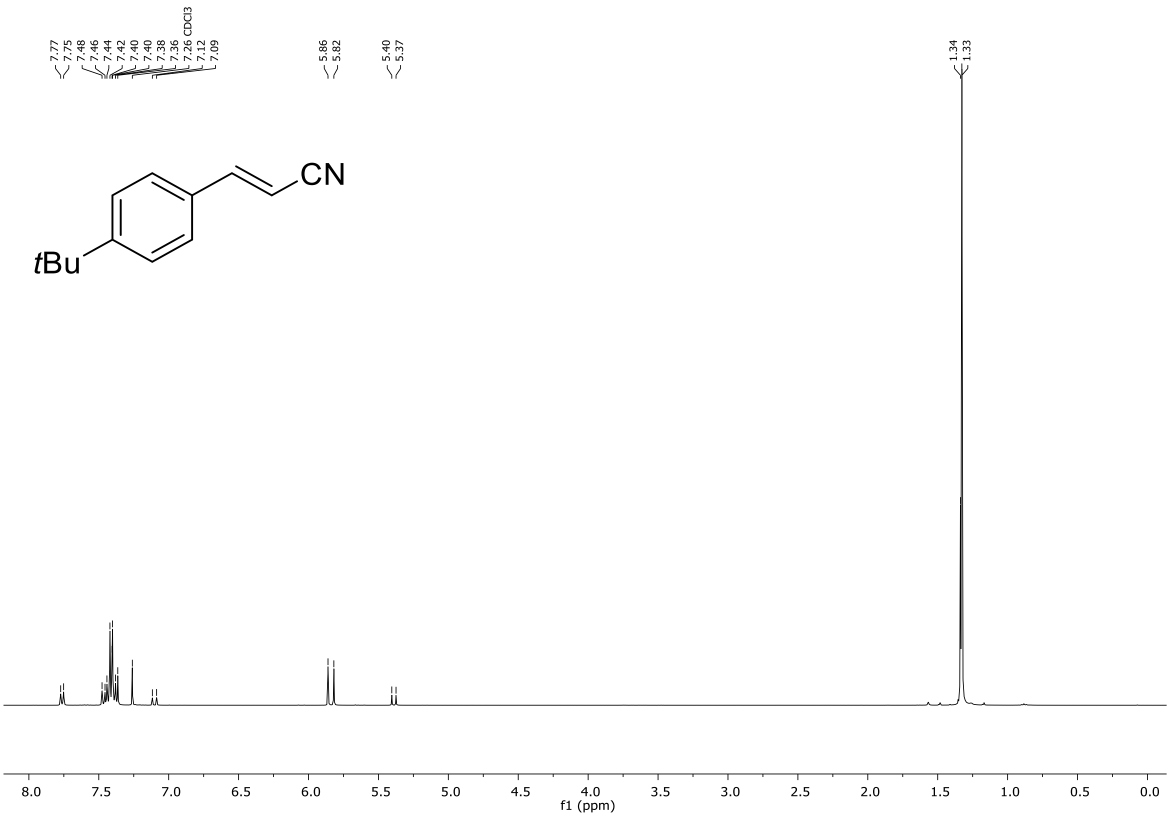

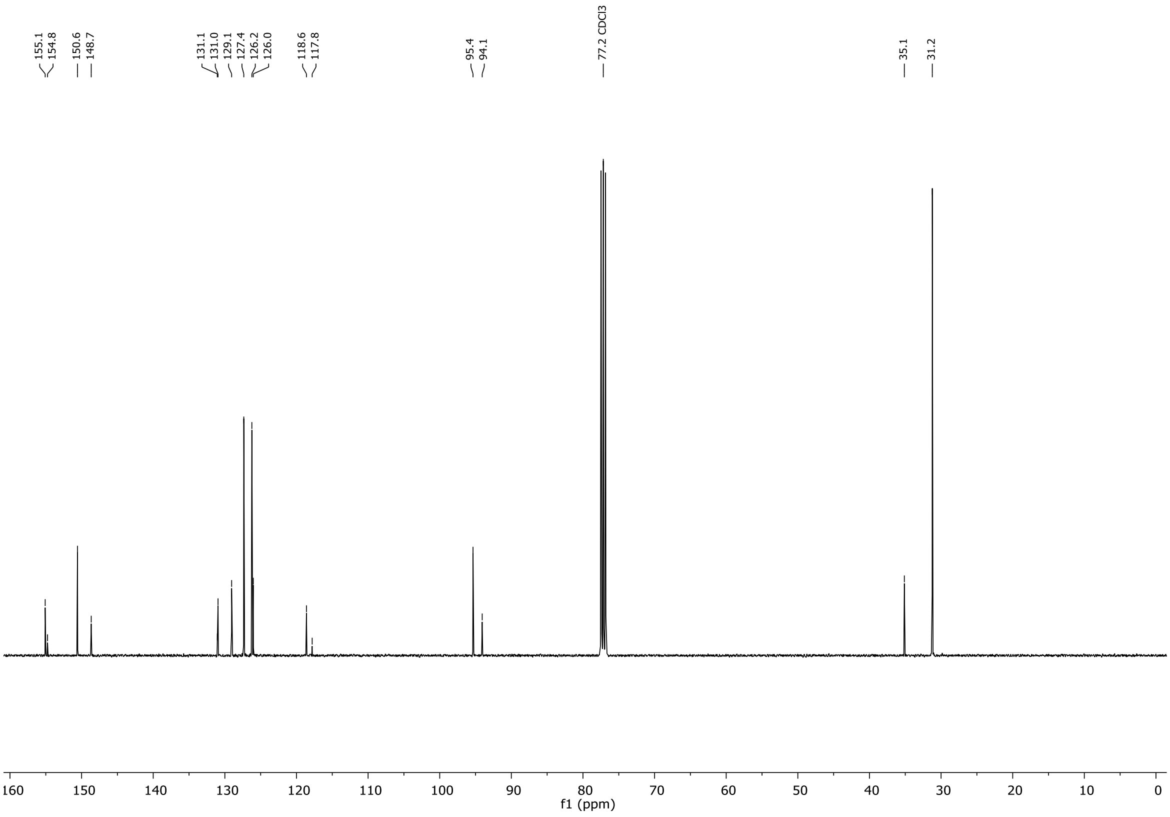

3-(4-(Trifluoromethyl)phenyl)acrylonitrile (5e)

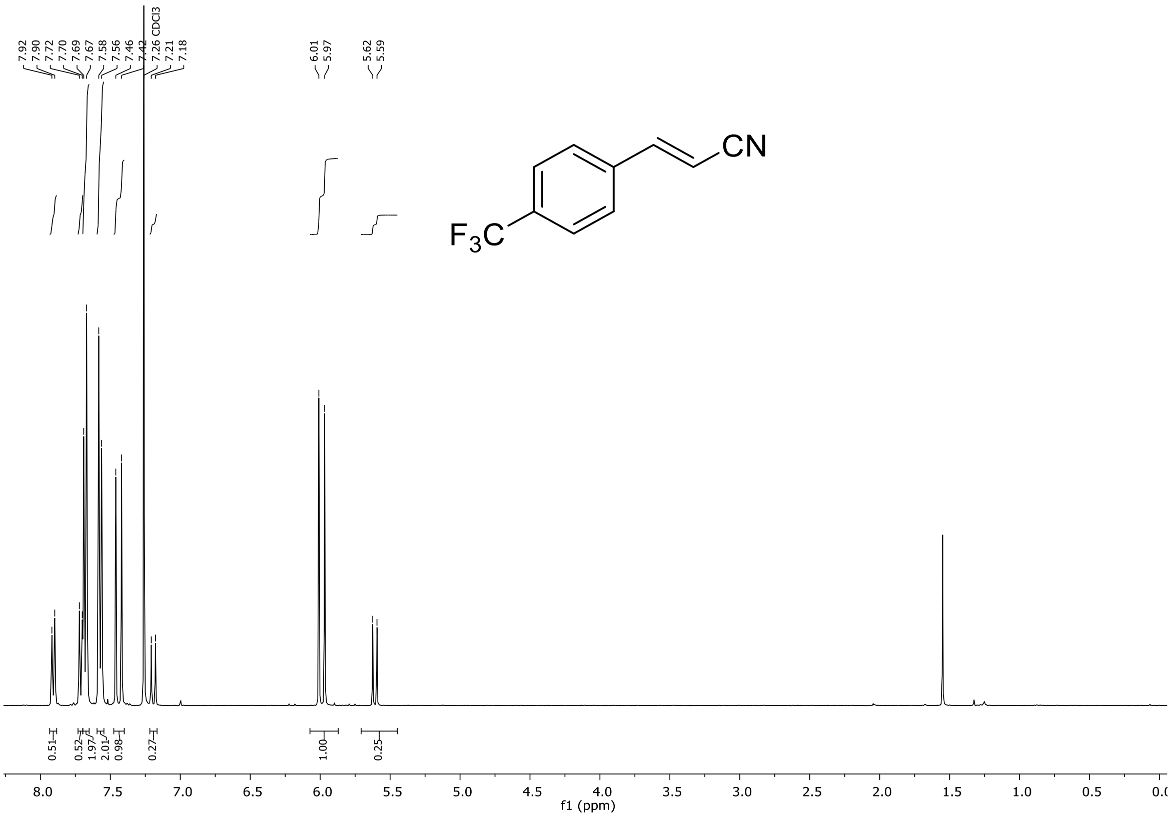

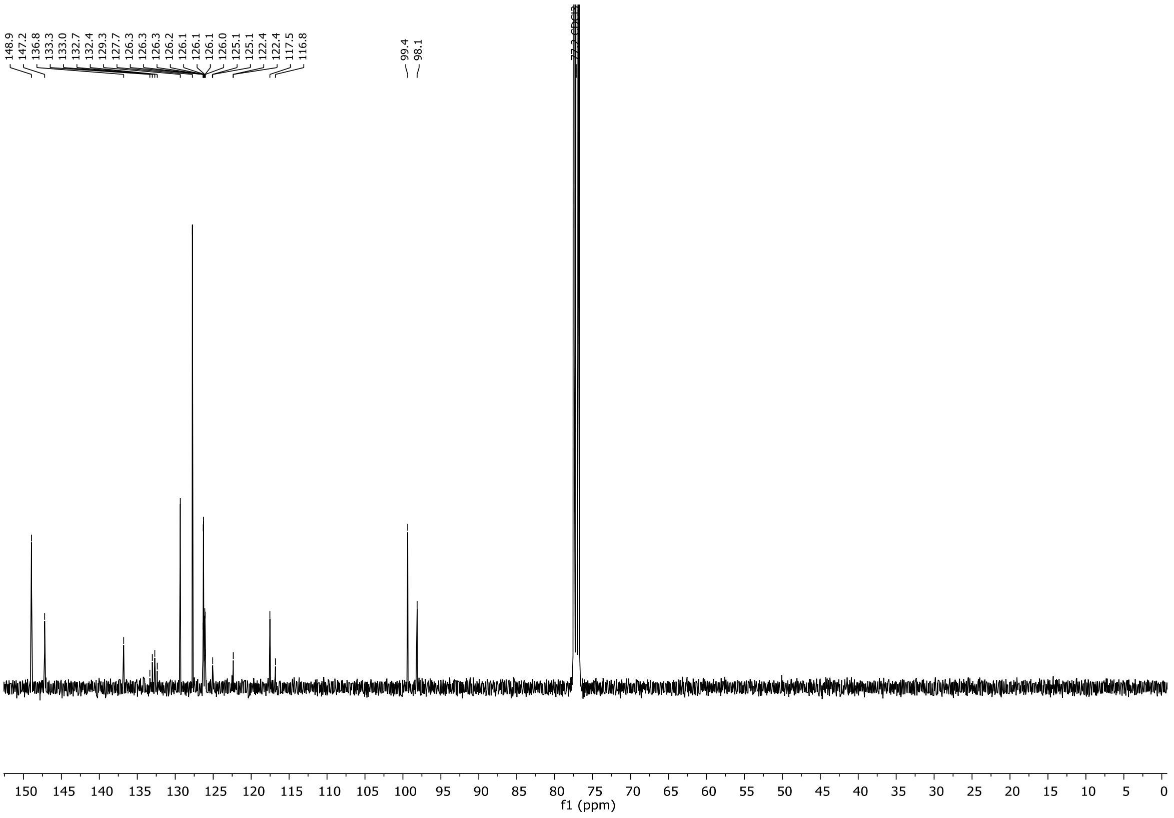

Phenyl(styryl)sulfane (5h)

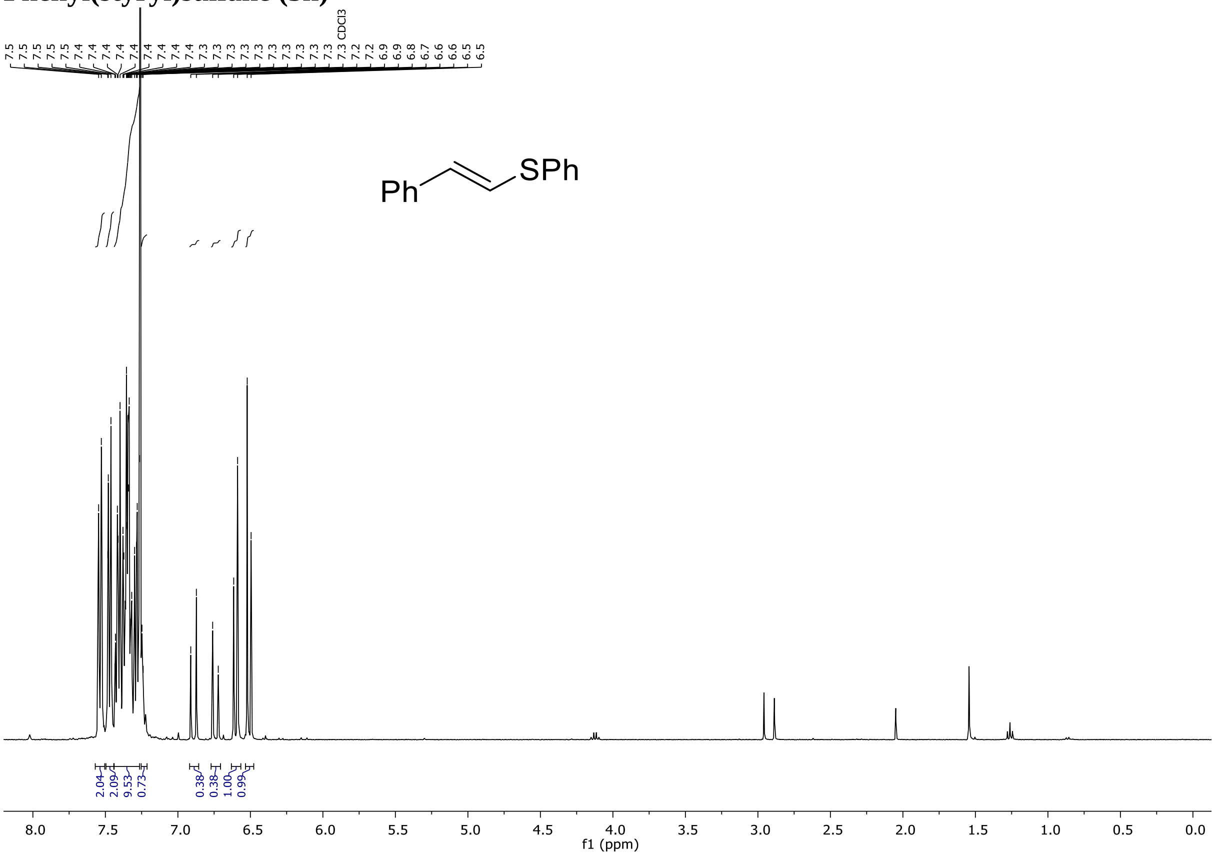

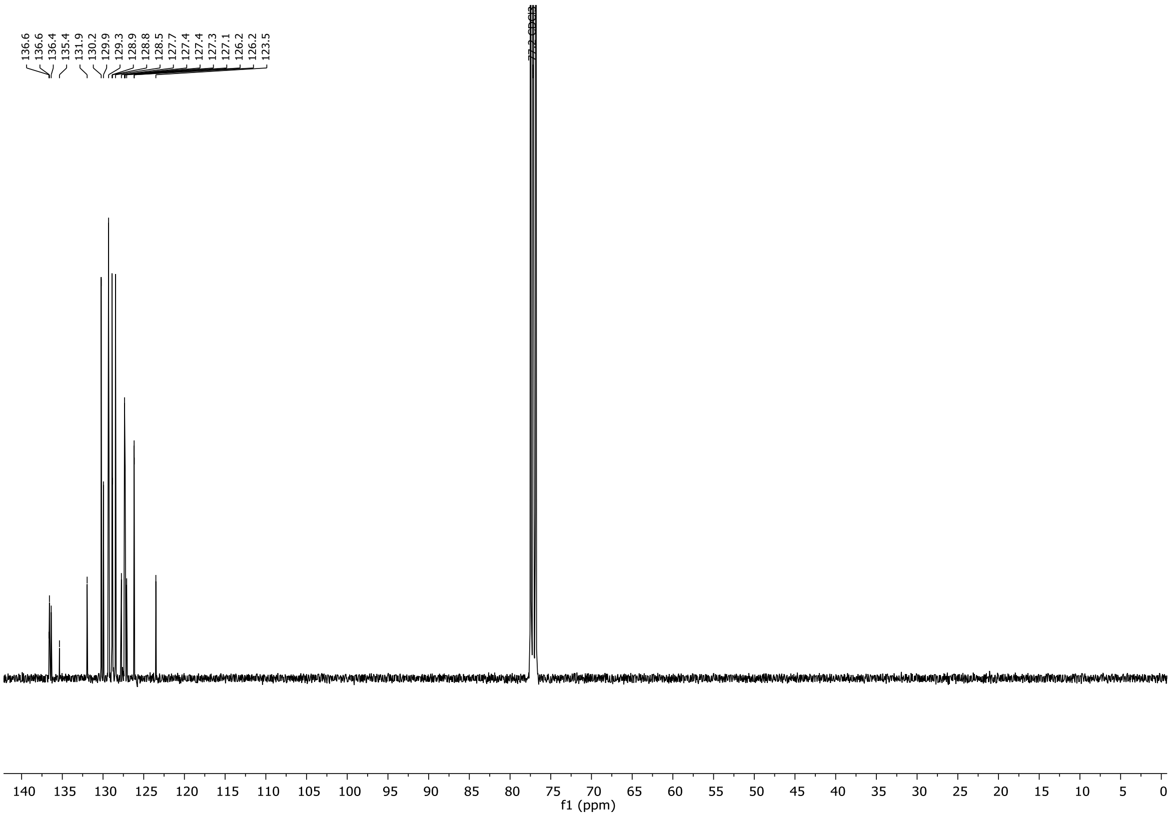

3-Methylhept-2-enenitrile (8b)

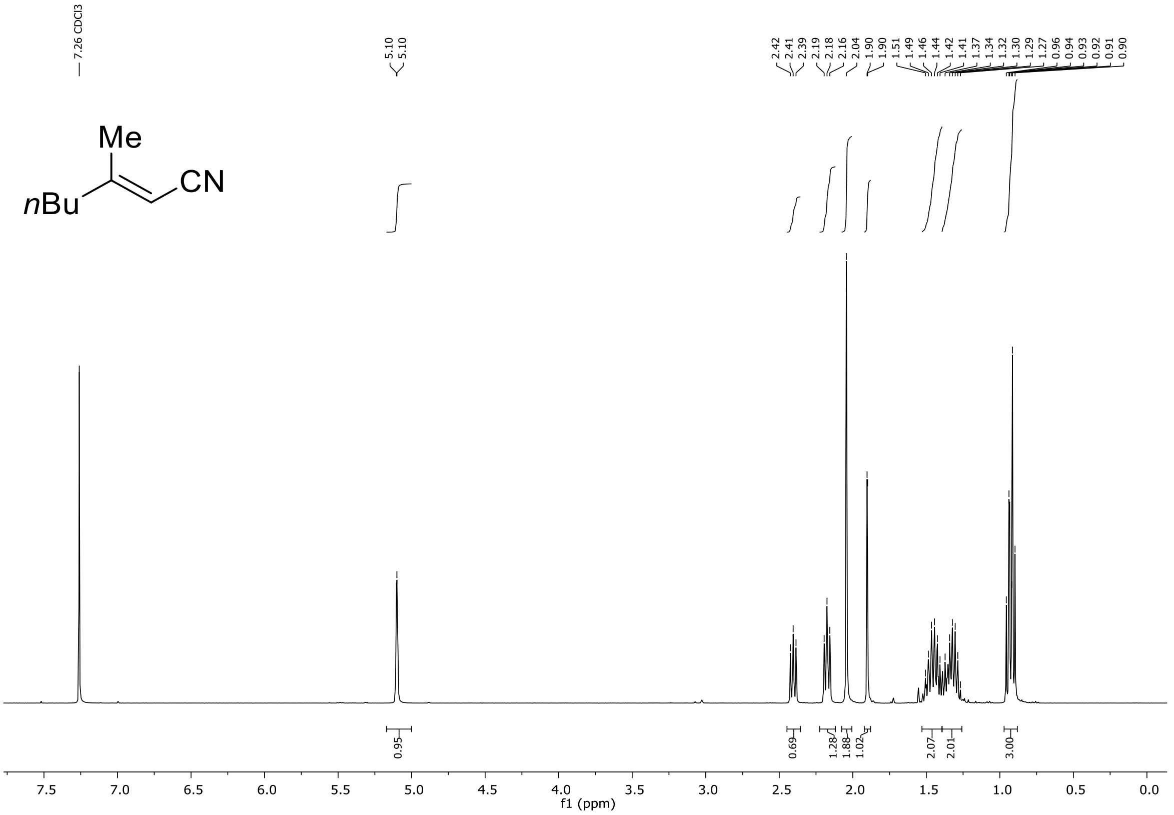

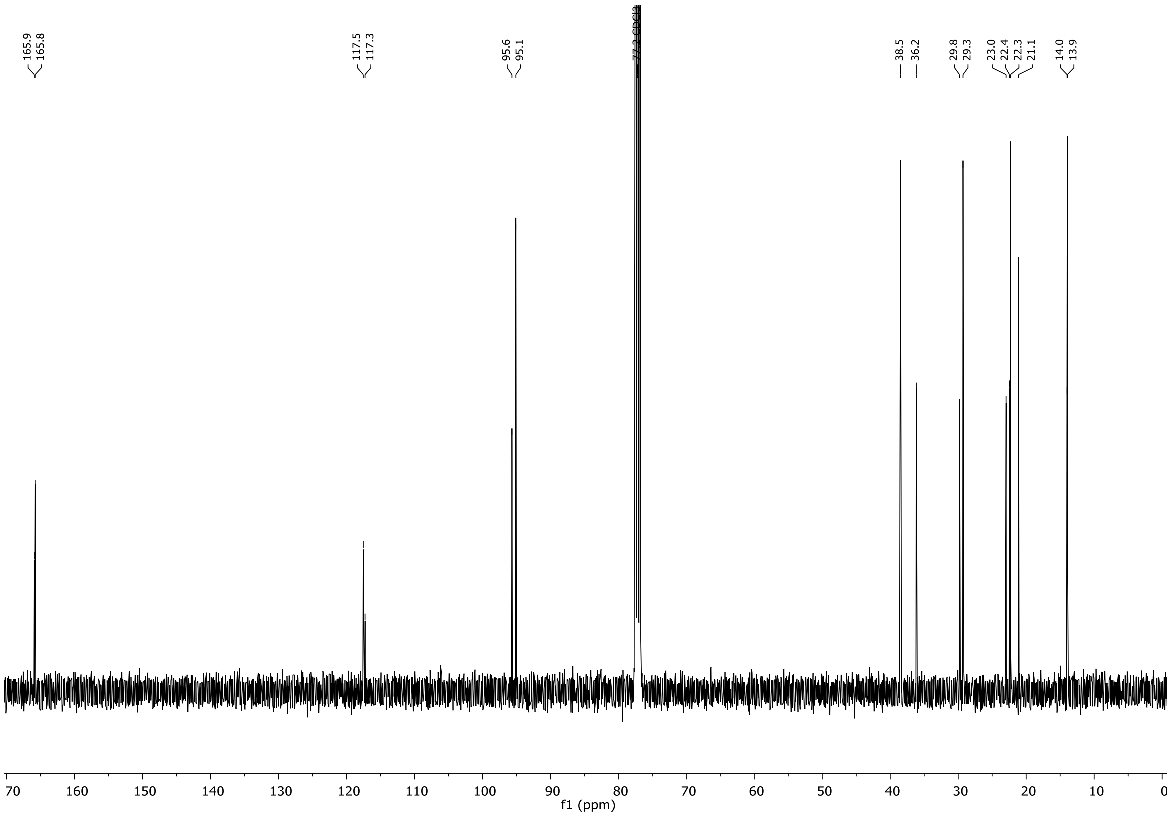

3-Oxohexanenitrile

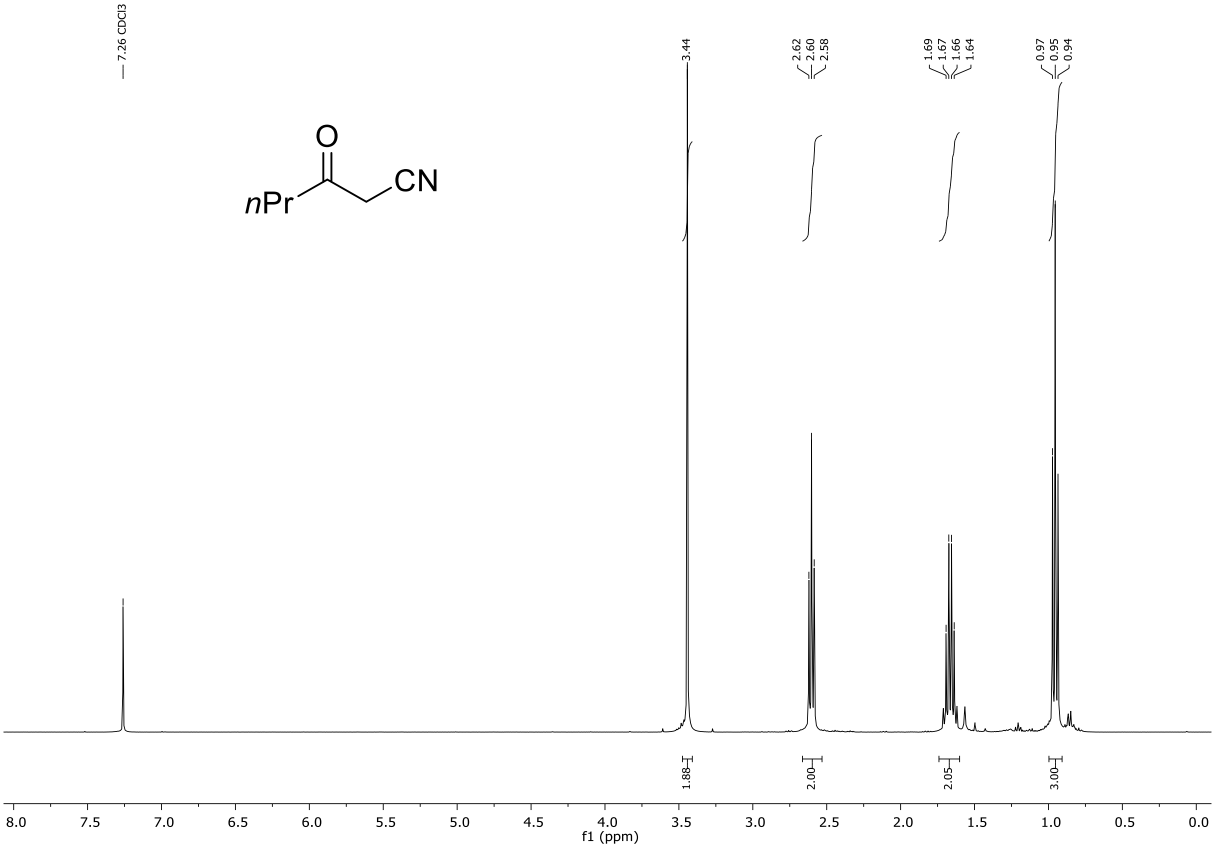

(*Z*)-1-cyanopent-1-en-2-yl trifluoromethanesulfonate

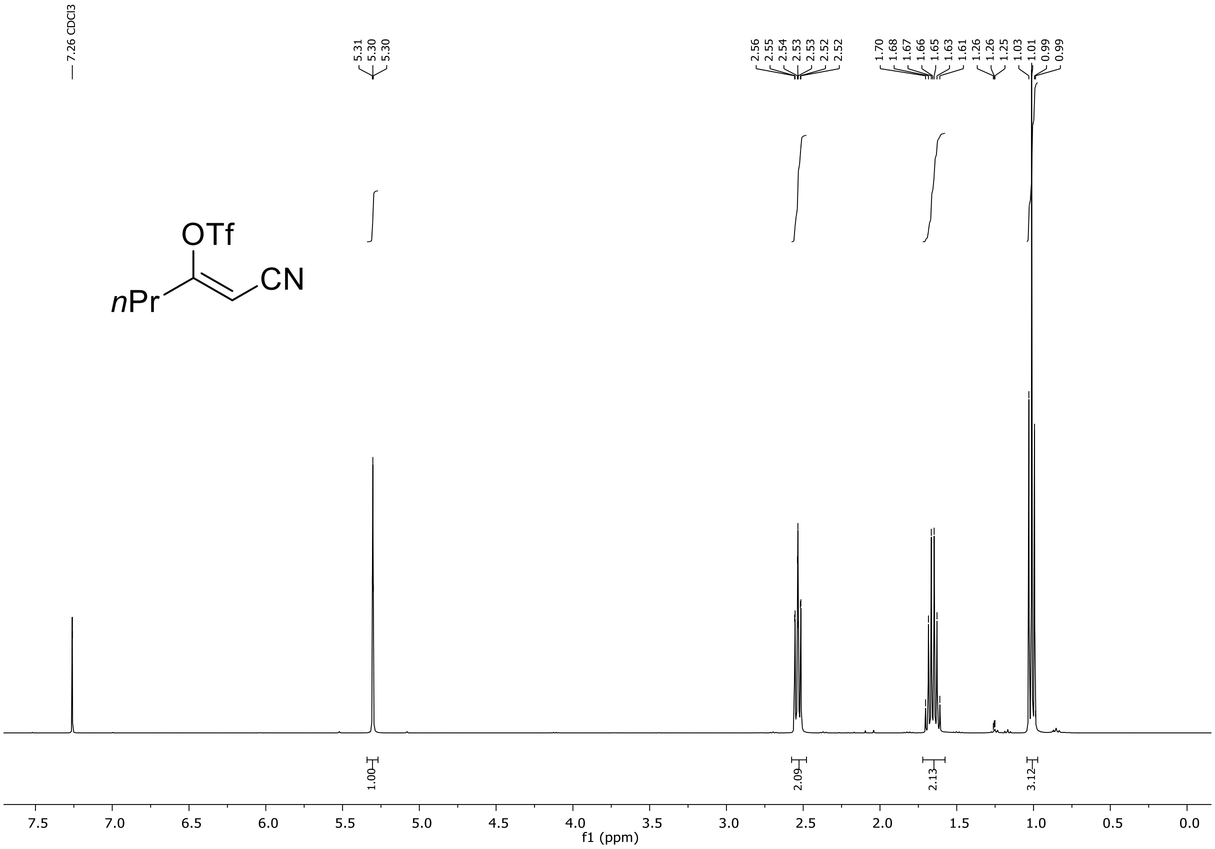

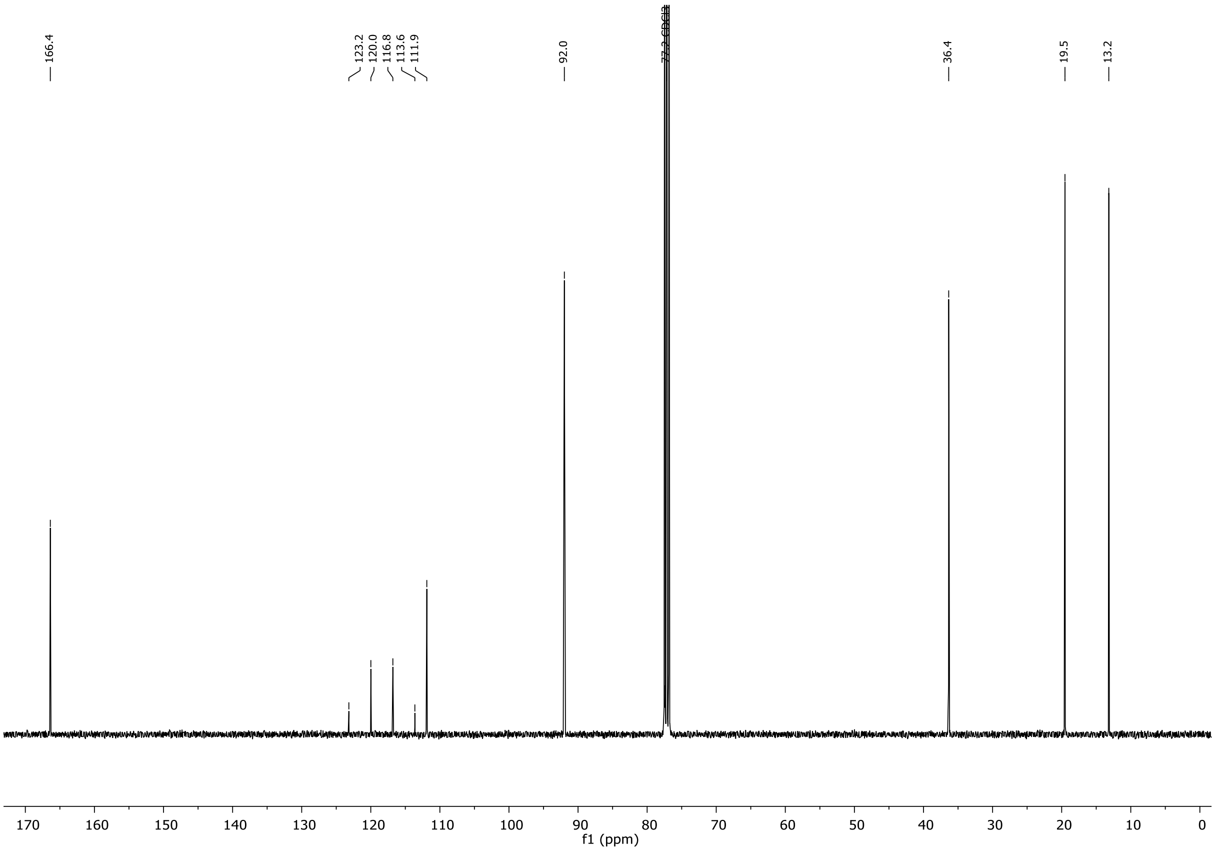

(*E*)-3-Methylhex-2-enenitrile (8c)

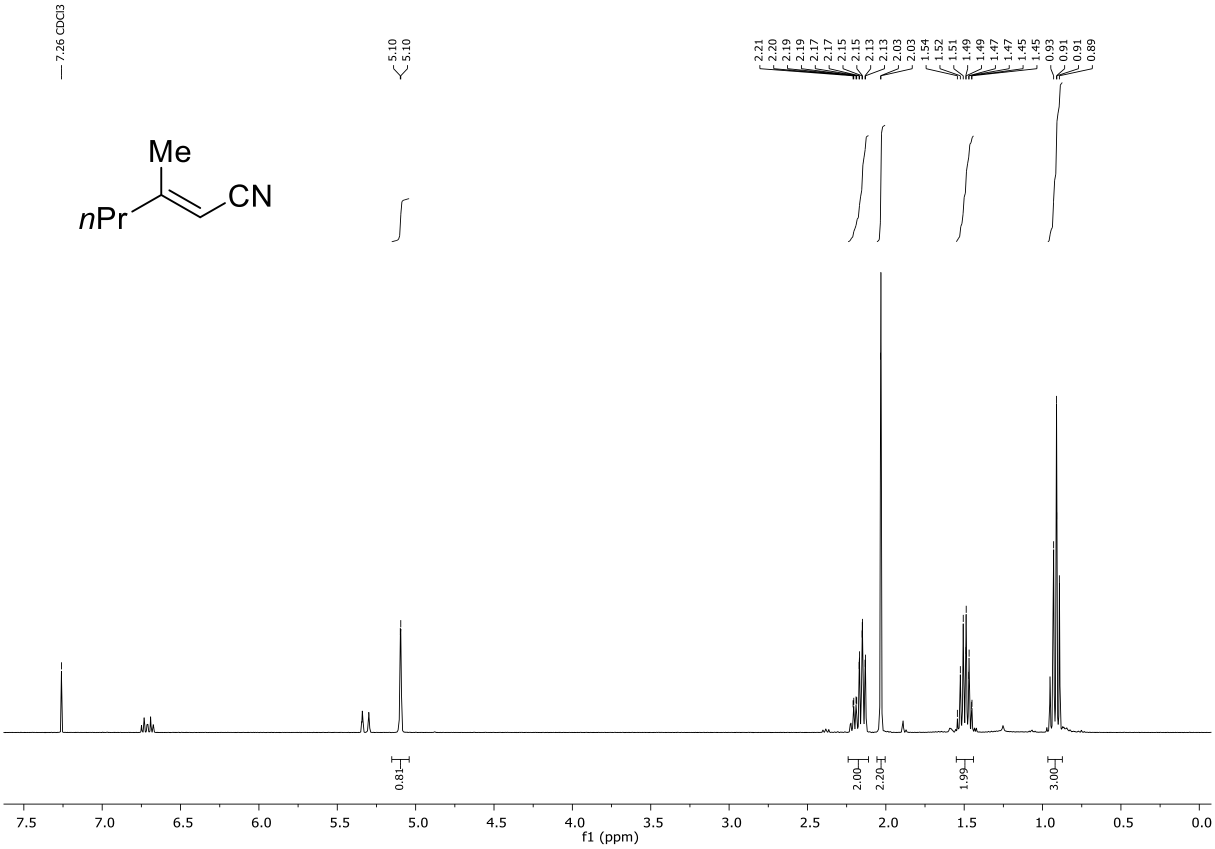

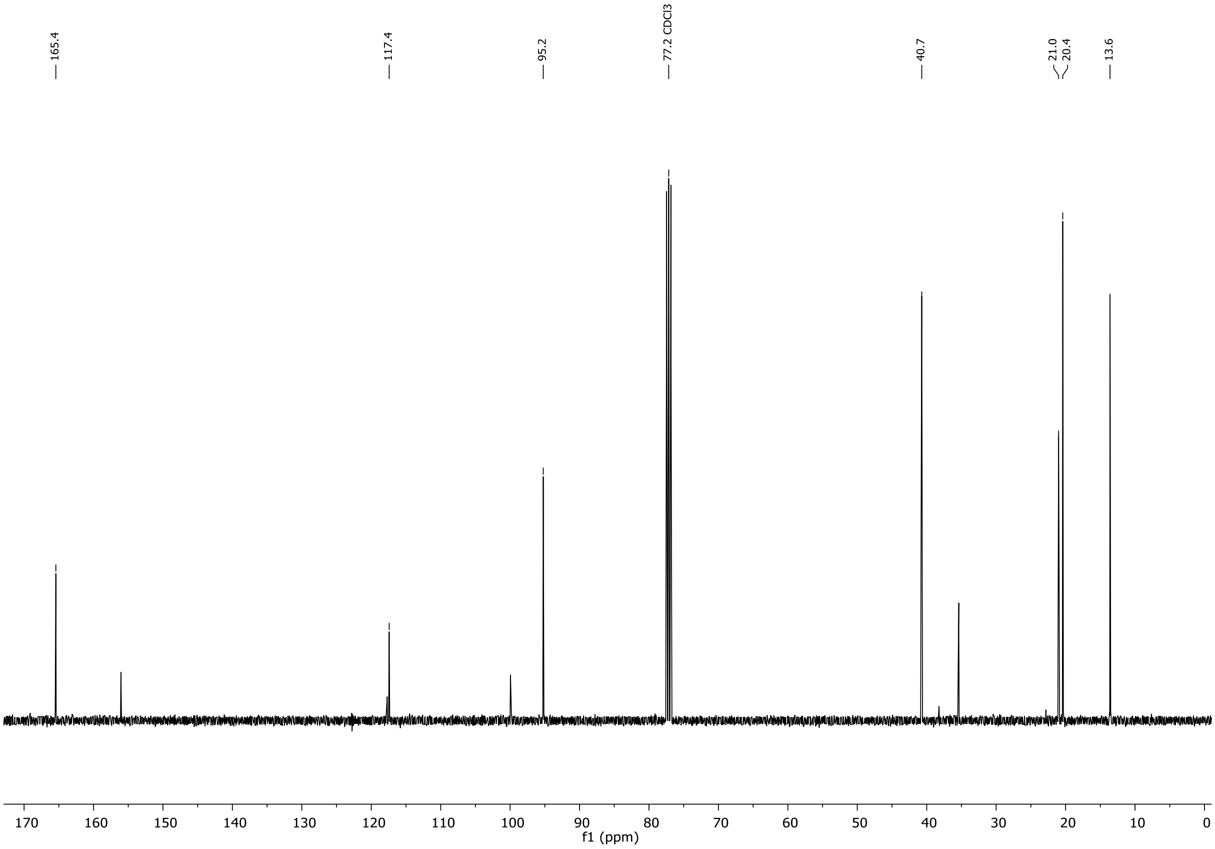

4-Methoxy-3-methylbut-2-enenitrile (8d)

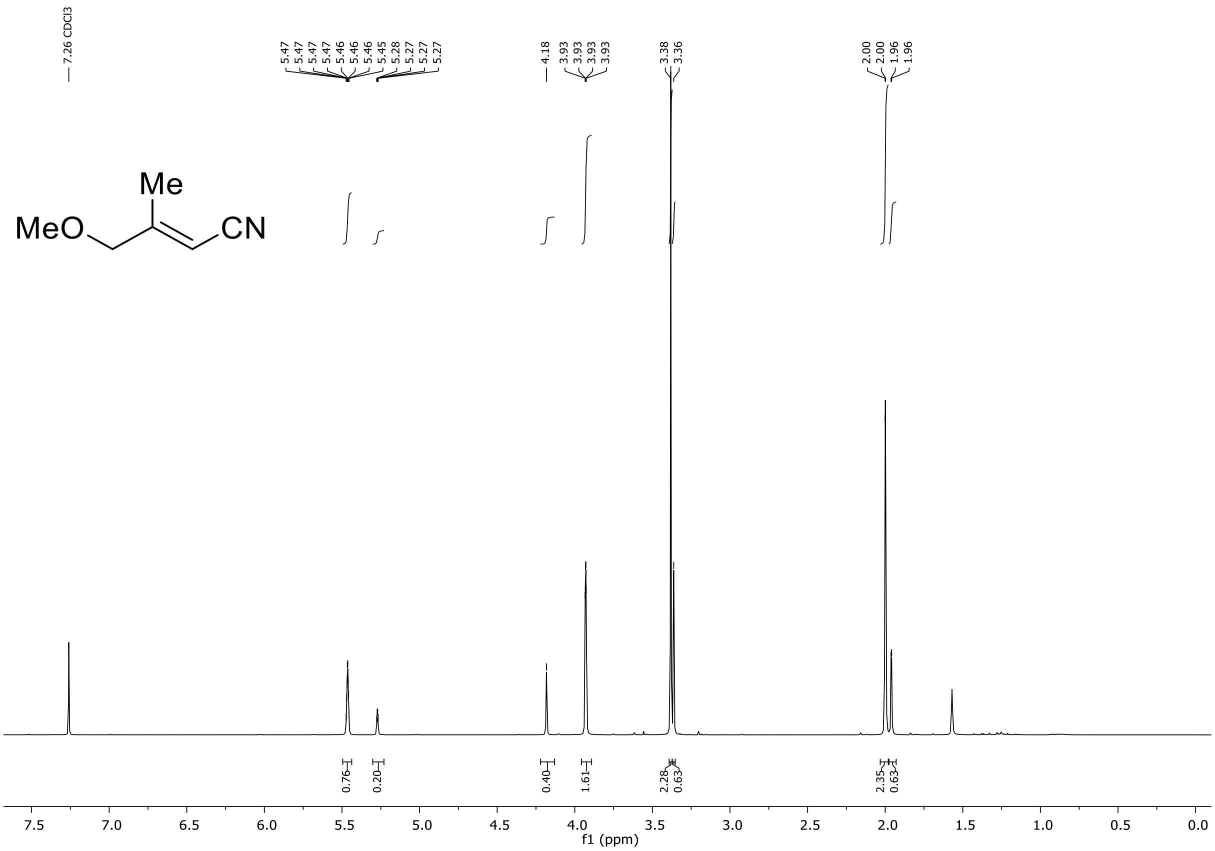

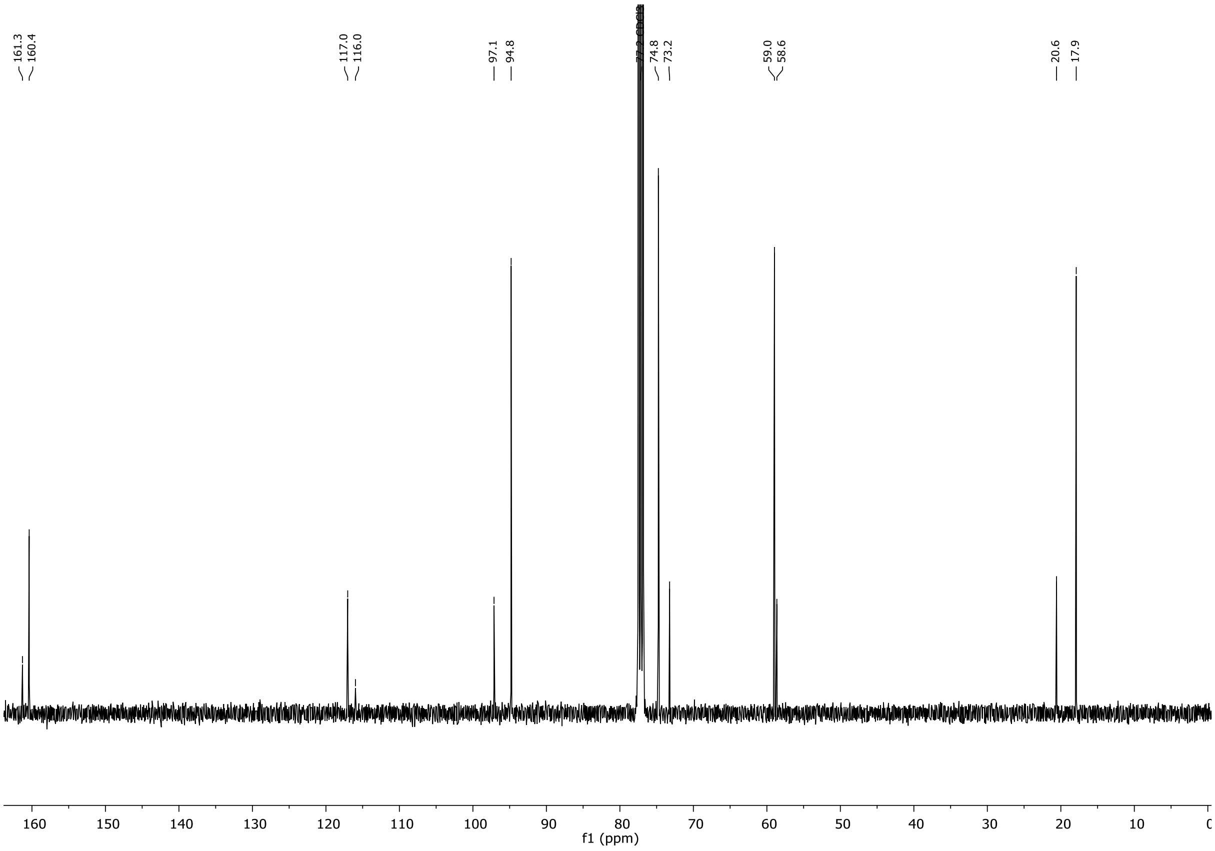

(*4E*)-Nona-2,4-dienenitrile (8e)

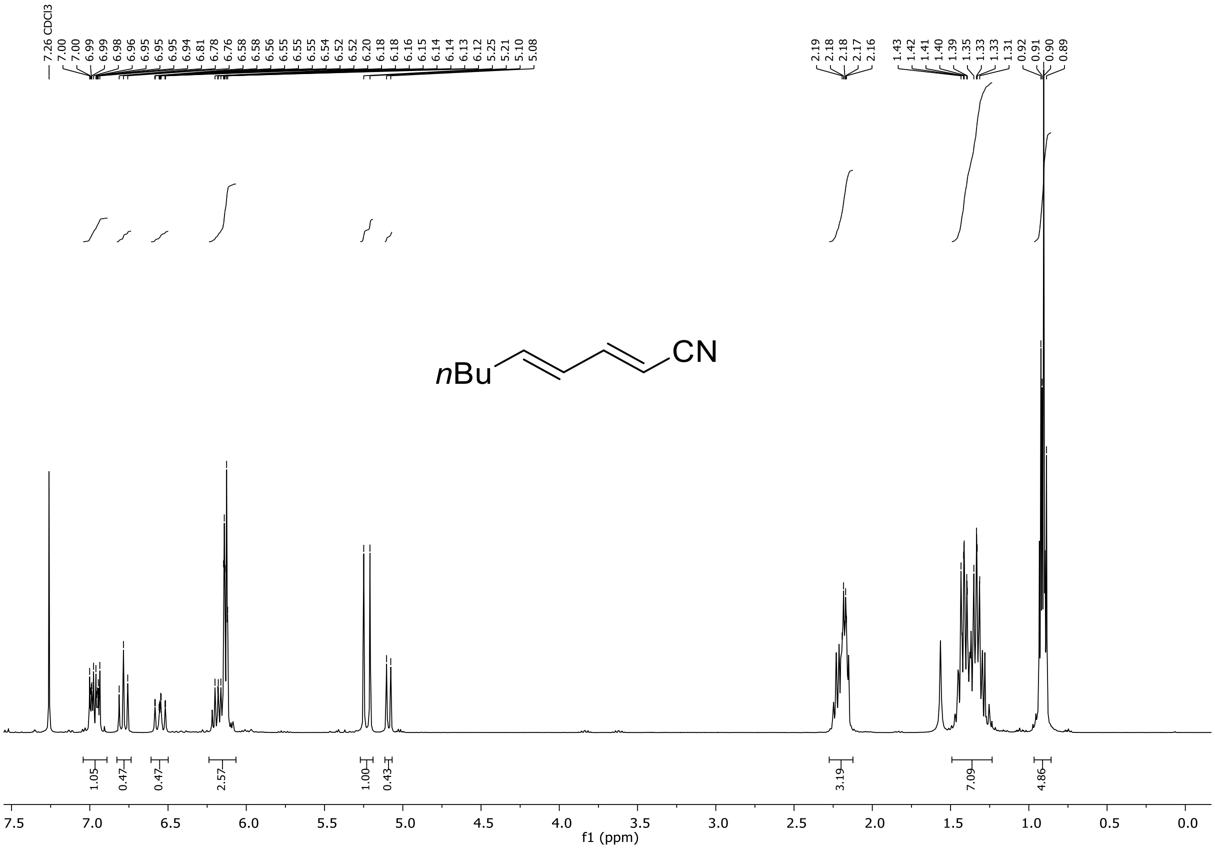

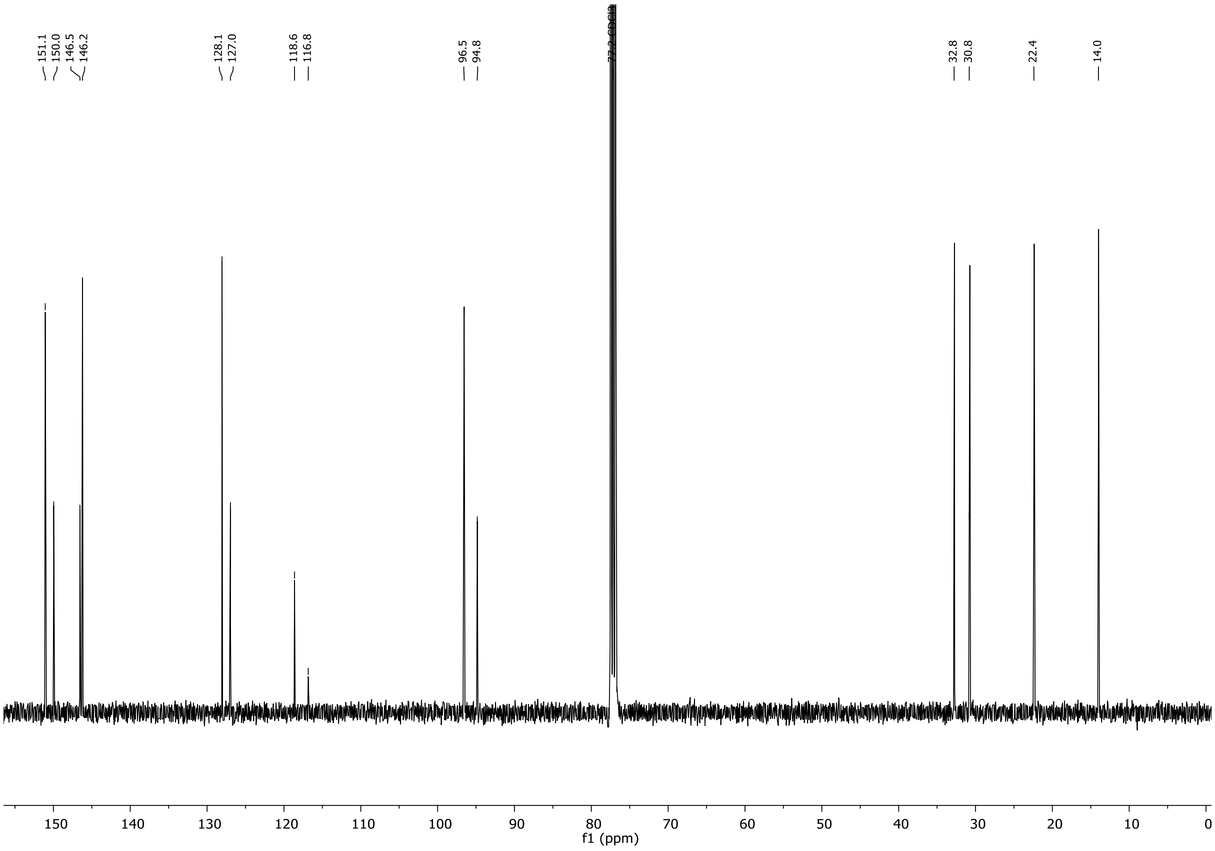

***(E)-Tert*-butyl cinnamate (11b)**

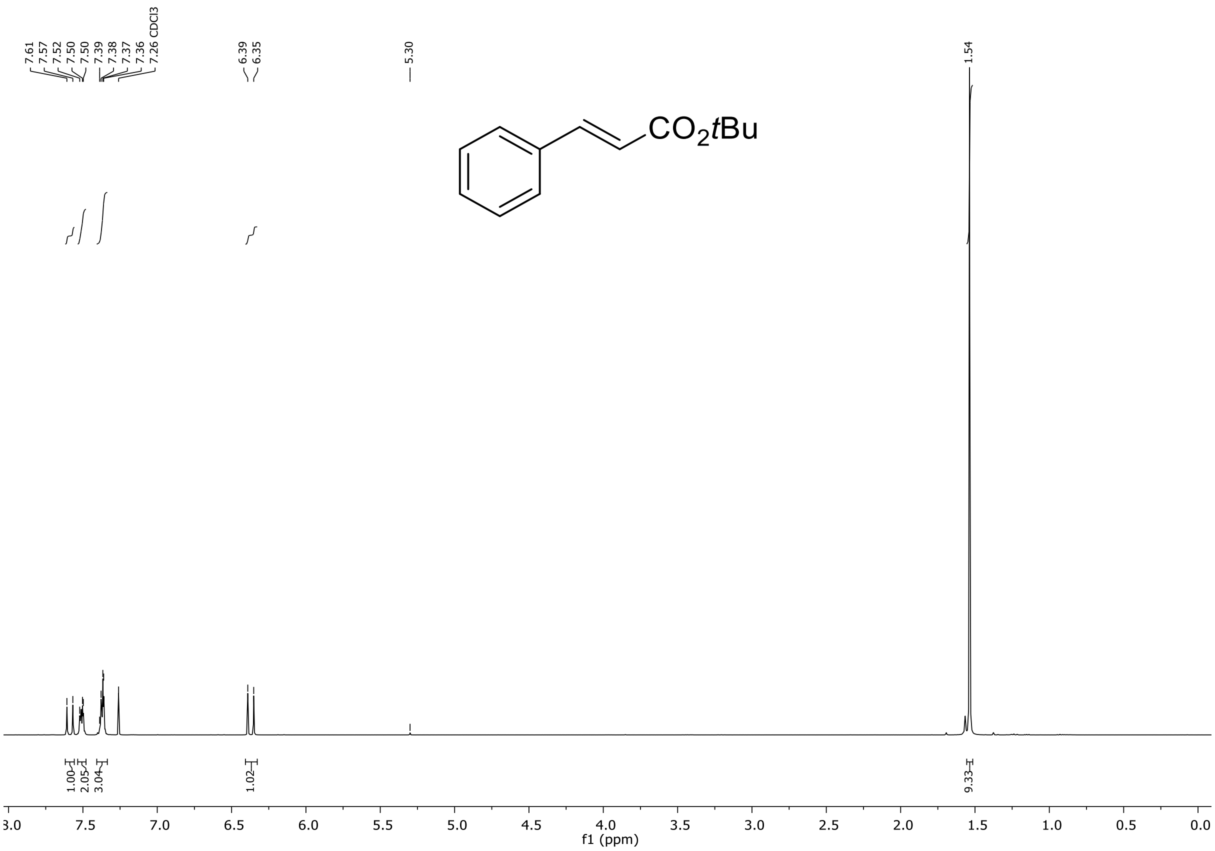

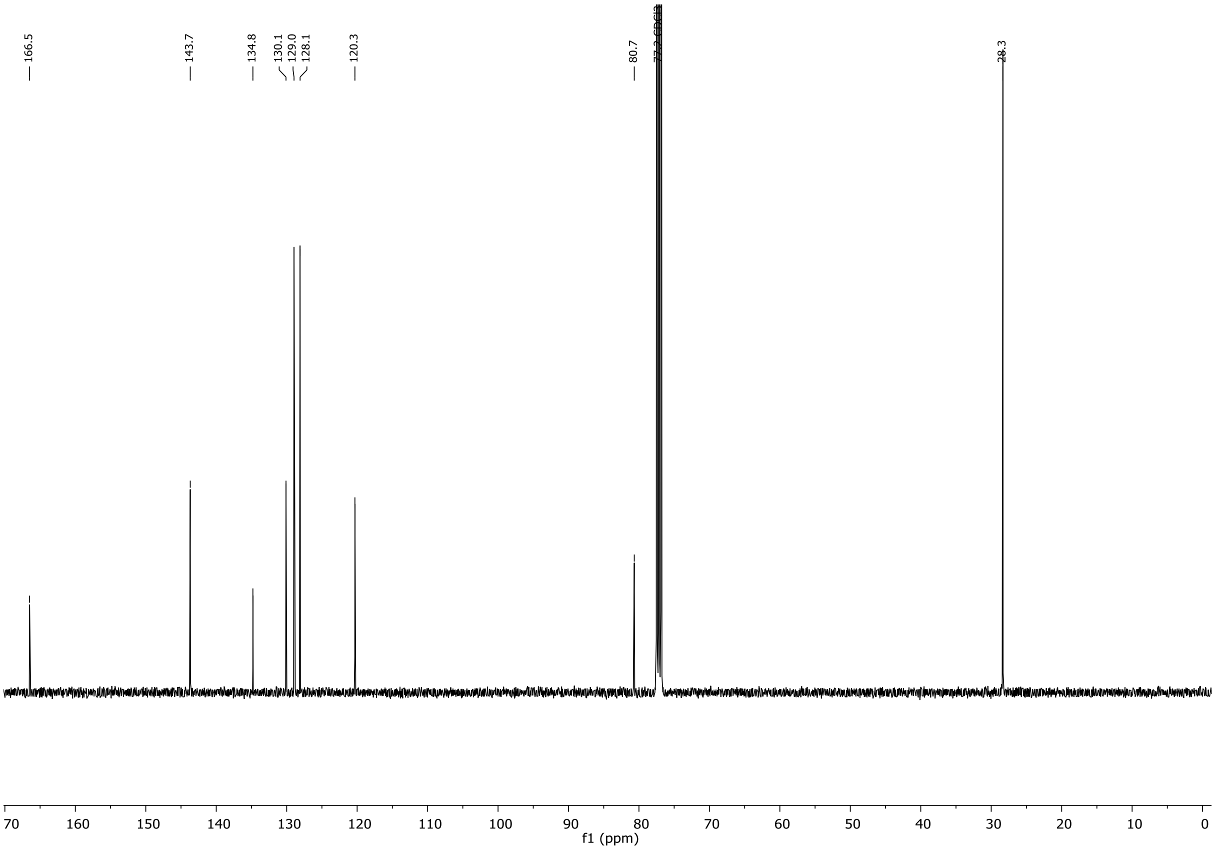

Single crystall X-ray diffraction studies

Single crystals of compound **4ad**, suitable for X-ray diffraction, were obtained by slow evaporation of CH_2Cl_2 solution. The crystals were introduced into perfluorinated oil and a suitable single crystal was carefully mounted on the top of a thin glass wire. Data collection was performed with an Oxford Xcalibur 3 diffractometer equipped with a Spellman generator (50 kV, 40 mA) and a Kappa CCD detector, operating with Mo-K_α radiation (λ = 0.71071 Å).

Data collection and data reduction were performed with the CrysAlisPro software.[16] Absorption correction using the multiscan method[17] was applied. The structures were solved with SHELXS-97,[18] refined with SHELXL-97[19] and finally checked using PLATON.[20] Details for data collection and structure refinement are summarized in Table 3.

CCDC-1831884 contains supplementary crystallographic data for this compound. These data can be obtained free of charge from The Cambridge Crystallographic Data Centre *via* www.ccdc.cam.ac.uk/data_request/cif.

---

[16] Program package CrysAlisPro 1.171.38.46 (Rigaku OD, 2015).

[17] Program package CrysAlisPro 1.171.38.46 (Rigaku OD, 2015).

[18] Sheldrick, G. M. (1997) SHELXS-97: *Program for Crystal Structure Solution*, University of Göttingen, Germany.

[19] Sheldrick, G. M. (1997) SHELXL-97: *Program for the Refinement of Crystal Structures*, University of Göttingen, Germany.

[20] Spek, A. L. (1999) PLATON: *A Multipurpose Crystallographic Tool*, Utrecht University, Utrecht, The Netherlands.

Table 3. Details for X-ray data collection and structure refinement for compound **4ad**.

	**4ad**
Empirical formula	$C_{17}H_{15}NO$
Formula mass	249.30
T[K]	143(2)
Crystal size [mm]	0.49 × 0.12 × 0.07
Crystal description	colorless rod
Crystal system	monoclinic
Space group	*P*21/*c*
a [Å]	5.4338(5)
b [Å]	15.5658(15)
c [Å]	15.8523(12)
α [°]	90
β [°]	90.515(7)
γ [°]	90
V [Å^3]	1340.8(2)
Z	4
$\rho_{calcd.}$ [g cm^{-3}]	1.235
μ [mm^{-1}]	0.077
F(000)	528
Θ range [°]	4.17 – 25.24
Index ranges	$-6 \leq h \leq 6$
	$-19 \leq k \leq 17$
	$-19 \leq l \leq 18$
Reflns. collected	9448
Reflns. obsd.	1501
Reflns. unique	2726 (R_{int} = 0.0744)
R_1, wR_2 (2σ data)	0.0570, 0.1081
R_1, wR_2 (all data)	0.1207, 0.1329
GOOF on F^2	0.994
Peak/hole [e Å^{-3}]	0.197 / -0.204

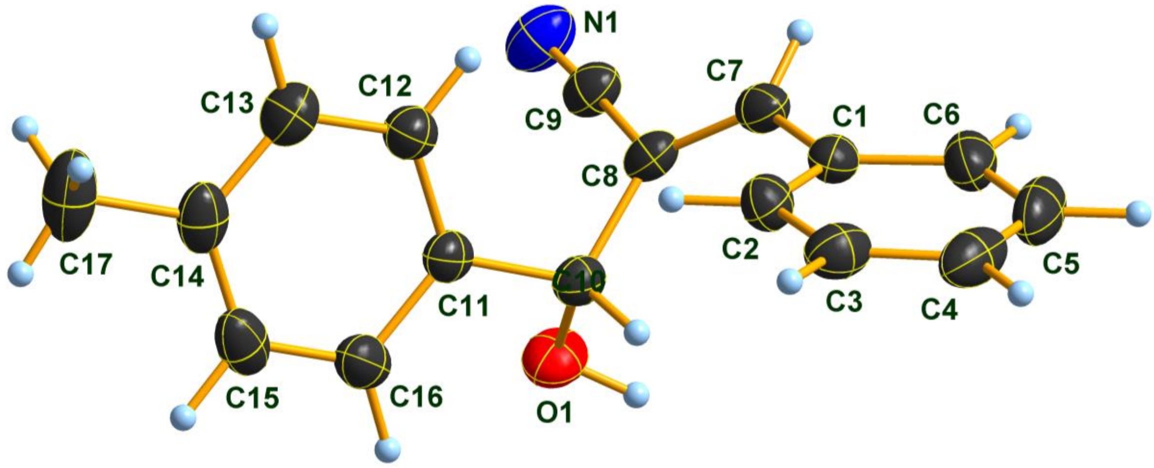

Figure 1. Molecular structure of compound **4ad** in the crystal, DIAMOND[21] representation; thermal ellipsoids are drawn at 50 % probability level.

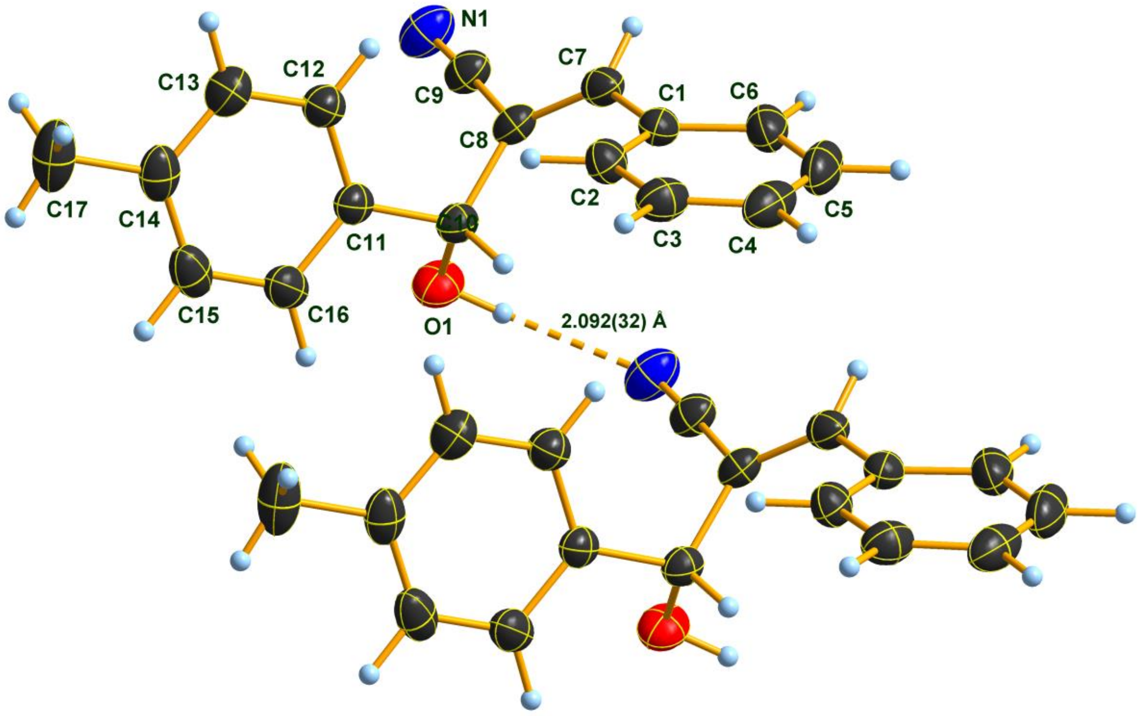

Figure 2. Hydrogen bonding in the crystal of compound **4ad**, DIAMOND[22] representation; thermal ellipsoids are drawn at 50 % probability level. Symmetry code for the second (not labeled) molecule: 1+x, y, z.

[21] DIAMOND, Crystal Impact GbR., Version 3.2i.
[22] DIAMOND, Crystal Impact GbR., Version 3.2i.

Table 4. Selected bond lengths (Å) of compound **4ad**.

O1 – C10	1.434(3)	C14 – C13	1.384(3)
C8 – C7	1.342(3)	C14 – C15	1.386(3)
C8 – C9	1.443(3)	C14 – C17	1.509(3)
C8 – C10	1.521(3)	C12 – C13	1.387(3)
C11 – C12	1.387(3)	C6 – C5	1.382(4)
C11 – C16	1.392(3)	C3 – C4	1.377(4)
C11 – C10	1.512(3)	C5 – C4	1.384(4)
C1 – C6	1.391(3)	N1 – C9	1.148(3)
C1 – C2	1.395(3)	C16 – C15	1.387(3)
C1 – C7	1.466(3)	C2 – C3	1.388(3)

Table 5. Selected bond angles (°) of compound **4ad**.

C7 – C8 – C9	119.5(2)	C15 – C14 – C17	121.4(2)
C7 – C8 – C10	126.9(2)	C13 – C12 – C11	120.7(2)
C9 – C8 – C10	113.6(2)	C5 – C6 – C1	120.5(3)
C12 – C11 – C16	118.3(2)	C14 – C15 – C16	121.6(2)
C12 – C11 – C10	122.3(2)	C4 – C3 – C2	120.5(3)
C16 – C11 – C10	119.4(2)	C6 – C5 – C4	120.3(3)
O1 – C10 – C11	107.7(2)	C14 – C13 – C12	121.4(2)
O1 – C10 – C8	108.3(2)	C3 – C4 – C5	119.6(3)
C11 – C10 – C8	114.2(2)	N1 – C9 – C8	177.5(3)
C6 – C1 – C2	118.9(2)	C8 – C7 – C1	127.1(2)
C6 – C1 – C7	119.4(2)	C3 – C2 – C1	120.1(2)
C2 – C1 – C7	121.7(2)	C13 – C14 – C15	117.6(2)
C15 – C16 – C11	120.3(2)	C13 – C14 – C17	120.9(2)

Table 6. Selected torsion angles (°) of compound **4ad**.

C12 – C11 – C10 – O1	-132.8(2)	C7 – C1 – C2 – C3	179.5(2)
C16 – C11 – C10 – O1	48.3(3)	C16 – C11 – C12 – C13	-1.0(3)
C12 – C11 – C10 – C8	-12.5(3)	C10 – C11 – C12 – C13	-179.9(2)
C16 – C11 – C10 – C8	168.7(2)	C2 – C1 – C6 – C5	2.8(3)
C7 – C8 – C10 – O1	-129.0(2)	C7 – C1 – C6 – C5	-178.6(2)
C9 – C8 – C10 – O1	48.0(2)	C13 – C14 – C15 – C16	-1.7(4)
C7 – C8 – C10 – C11	111.0(3)	C17 – C14 – C15 – C16	177.6(2)
C9 – C8 – C10 – C11	-71.9(2)	C11 – C16 – C15 – C14	0.5(4)
C12 – C11 – C16 – C15	0.9(3)	C1 – C2 – C3 – C4	-0.6(3)
C10 – C11 – C16 – C15	179.8(2)	C1 – C6 – C5 – C4	-1.3(3)
C9 – C8 – C7 – C1	177.4(2)	C15 – C14 – C13 – C12	1.6(4)
C10 – C8 – C7 – C1	-5.7(4)	C17 – C14 – C13 – C12	-177.7(2)
C6 – C1 – C7 – C8	141.0(2)	C11 – C12 – C13 – C14	-0.3(4)
C2 – C1 – C7 – C8	-40.5(3)	C2 – C3 – C4 – C5	2.2(4)
C6 – C1 – C2 – C3	-1.9(3)	C6 – C5 – C4 – C3	-1.3(4)

Single crystals of compound **4ag**, suitable for X-ray diffraction, were obtained by slow evaporation of CH_2Cl_2 solution. The crystals were introduced into perfluorinated oil and a suitable single crystal was carefully mounted on the top of a thin glass wire. Data collection was performed with an Oxford Xcalibur 3 diffractometer equipped with a Spellman generator (50 kV, 40 mA) and a Kappa CCD detector, operating with Mo-K_α radiation (λ = 0.71071 Å).

Data collection and data reduction were performed with the CrysAlisPro software.[23] Absorption correction using the multiscan method[24] was applied. The structures were solved with SHELXS-97,[25] refined with SHELXL-97[26] and finally checked using PLATON.[27] Details for data collection and structure refinement are summarized in Table 7.

CCDC-2027326 contains supplementary crystallographic data for this compound. These data can be obtained free of charge from The Cambridge Crystallographic Data Centre *via* www.ccdc.cam.ac.uk/data_request/cif.

[23] Program package 'CrysAlisPro 1.171.39.46e (Rigaku OD, 2018)'.
[24] Program package 'CrysAlisPro 1.171.39.46e (Rigaku OD, 2018)'.
[25] Sheldrick, G. M. (1997) SHELXS-97: *Program for Crystal Structure Solution*, University of Göttingen, Germany.
[26] Sheldrick, G. M. (1997) SHELXL-97: *Program for the Refinement of Crystal Structures*, University of Göttingen, Germany.
[27] Spek, A. L. (1999) PLATON: *A Multipurpose Crystallographic Tool*, Utrecht University, Utrecht, The Netherlands.

Table 7. Details for X-ray data collection and structure refinement for compound **4ag**.

	**4ag**
Empirical formula	$C_{22}H_{17}NO$
Formula mass	311.36
T[K]	143(2)
Crystal size [mm]	0.40 × 0.10 × 0.05
Crystal description	colorless rod
Crystal system	orthorhombic
Space group	*P*212121
a [Å]	8.2139(4)
b [Å]	11.0953(6)
c [Å]	18.3781(9)
α [°]	90.0
β [°]	90.0
γ [°]	90.0
V [Å^3]	1674.90(15)
Z	4
$\rho_{calcd.}$ [g cm^{-3}]	1.235
μ [mm^{-1}]	0.075
F(000)	656
Θ range [°]	3.28 – 25.24
Index ranges	$-9 \leq h \leq 10$
	$-14 \leq k \leq 14$
	$-21 \leq l \leq 24$
Reflns. collected	13597
Reflns. obsd.	3180
Reflns. unique	4144 (R_{int} = 0.0644)
R_1, wR_2 (2σ data)	0.0502, 0.0817
R_1, wR_2 (all data)	0.0763, 0.0922
GOOF on F^2	1.028
Peak/hole [e Å^{-3}]	0.201 / -0.208

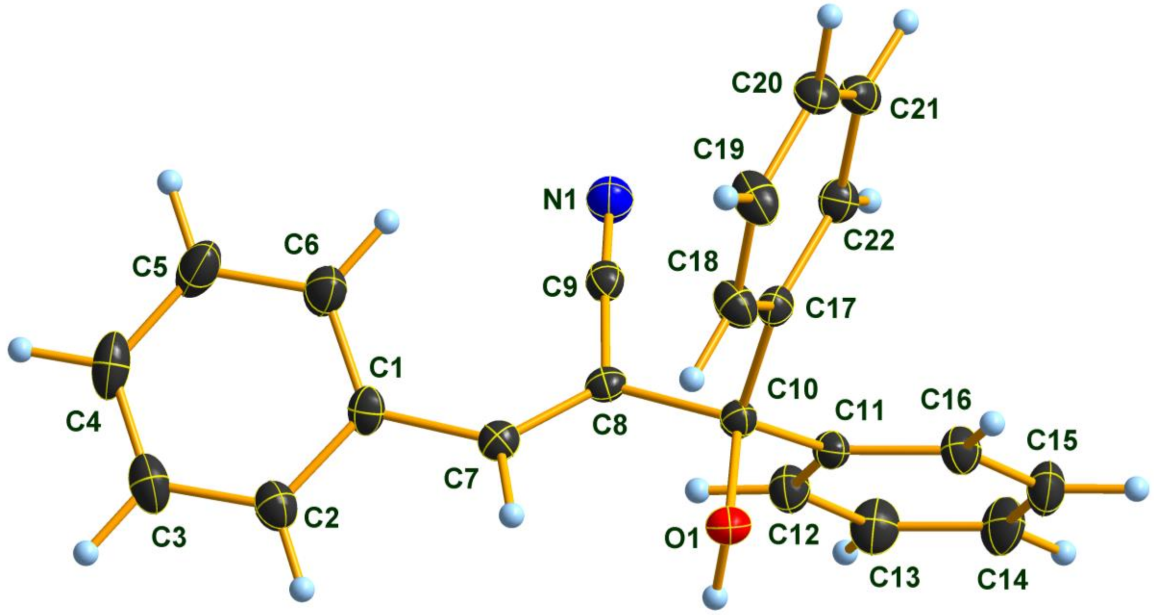

Figure 3. Molecular structure of compound **4ag** in the crystal. DIAMOND[28] representation; thermal ellipsoids are drawn at 50 % probability level.

Table 8. Selected bond lengths (Å) of compound **4ag**.

O1 – C10	1.419(3)	C15 – C14	1.382(4)
C7 – C8	1.342(3)	C1 – C6	1.394(4)
C7 – C1	1.467(3)	C1 – C2	1.398(4)
C8 – C9	1.431(4)	C2 – C3	1.383(4)
C8 – C10	1.548(3)	C22 – C21	1.385(3)
N1 – C9	1.149(3)	C3 – C4	1.376(4)
C17 – C22	1.389(3)	C13 – C14	1.377(4)
C17 – C18	1.389(3)	C5 – C4	1.381(4)
C17 – C10	1.524(3)	C5 – C6	1.383(4)
C18 – C19	1.384(4)	C12 – C11	1.383(3)
C16 – C15	1.385(4)	C12 – C13	1.392(4)
C16 – C11	1.396(3)	C20 – C19	1.381(4)
C10 – C11	1.531(3)	C20 – C21	1.385(4)

[28] DIAMOND, Crystal Impact GbR., Version 3.2i.

Table 9. Selected bond angles (°) of compound **4ag**.

C8 – C7 – C1	130.2(2)	C2 – C1 – C7	118.1(2)
C7 – C8 – C9	122.1(2)	C3 – C2 – C1	121.0(3)
C7 – C8 – C10	123.3(2)	C12 – C11 – C16	118.6(2)
C9 – C8 – C10	114.5(2)	C12 – C11 – C10	121.6(2)
C22 – C17 – C18	118.7(2)	C16 – C11 – C10	119.5(2)
C22 – C17 – C10	122.6(2)	C21 – C22 – C17	120.7(3)
C18 – C17 – C10	118.4(2)	C20 – C21 – C22	120.2(2)
C19 – C18 – C17	120.5(3)	C4 – C3 – C2	119.8(3)
C15 – C16 – C11	120.5(3)	C14 – C13 – C12	119.9(3)
N1 – C9 – C8	176.8(3)	C4 – C5 – C6	120.1(3)
O1 – C10 – C17	106.5(2)	C20 – C19 – C18	120.5(3)
O1 – C10 – C11	109.3(2)	C5 – C6 – C1	120.7(3)
C17 – C10 – C11	114.9(2)	C13 – C14 – C15	120.0(3)
O1 – C10 – C8	109.6(2)	C3 – C4 – C5	120.3(3)
C17 – C10 – C8	104.6(2)	C14 – C15 – C16	120.1(3)
C11 – C10 – C8	111.7(2)	C6 – C1 – C2	118.1(2)
C11 – C12 – C13	120.8(3)	C6 – C1 – C7	123.7(2)
C19 – C20 – C21	119.3(3)		

Table 10. Selected torsion angles (°) of compound **4ag**.

C1 – C7 – C8 – C9	-3.4(4)	C15 – C16 – C11 – C12	0.5(4)
C1 – C7 – C8 – C10	172.6(2)	C15 – C16 – C11 – C10	174.4(2)
C22 – C17 – C18 – C19	1.3(4)	O1 – C10 – C11 – C12	98.4(3)
C10 – C17 – C18 – C19	-173.2(2)	C17 – C10 – C11 – C12	-141.9(2)
C22 – C17 – C10 – O1	155.3(2)	C8 – C10 – C11 – C12	-23.0(3)
C18 – C17 – C10 – O1	-30.4(3)	O1 – C10 – C11 – C16	-75.4(3)
C22 – C17 – C10 – C11	34.1(3)	C17 – C10 – C11 – C16	44.3(3)
C18 – C17 – C10 – C11	-151.6(2)	C8 – C10 – C11 – C16	163.2(2)
C22 – C17 – C10 – C8	-88.7(3)	C18 – C17 – C22 – C21	-0.4(4)
C18 – C17 – C10 – C8	85.6(3)	C10 – C17 – C22 – C21	173.9(2)
C7 – C8 – C10 – O1	6.1(3)	C19 – C20 – C21 – C22	1.9(4)
C9 – C8 – C10 – O1	-177.6(2)	C17 – C22 – C21 – C20	-1.2(4)
C7 – C8 – C10 – C17	-107.8(3)	C1 – C2 – C3 – C4	-1.1(4)
C9 – C8 – C10 – C17	68.6(3)	C11 – C12 – C13 – C14	0.7(4)
C7 – C8 – C10 – C11	127.3(2)	C21 – C20 – C19 – C18	-1.0(4)
C9 – C8 – C10 – C11	-56.3(3)	C17 – C18 – C19 – C20	-0.6(4)
C11 – C16 – C15 – C14	0.2(4)	C4 – C5 – C6 – C1	0.2(4)
C8 – C7 – C1 – C6	-23.0(4)	C2 – C1 – C6 – C5	-1.8(4)
C8 – C7 – C1 – C2	159.2(3)	C7 – C1 – C6 – C5	-179.5(3)
C6 – C1 – C2 – C3	2.2(4)	C12 – C13 – C14 – C15	0.0(4)
C7 – C1 – C2 – C3	-179.9(3)	C16 – C15 – C14 – C13	-0.4(4)
C13 – C12 – C11 – C16	-0.9(4)	C2 – C3 – C4 – C5	-0.5(4)
C13 – C12 – C11 – C10	-174.8(2)	C6 – C5 – C4 – C3	1.0(5)

Single crystals of compound **4ah**, suitable for X-ray diffraction, were obtained by slow evaporation of CH_2Cl_2 solution. The crystals were introduced into perfluorinated oil and a suitable single crystal was carefully mounted on the top of a thin glass wire. Data collection was performed with an Oxford Xcalibur 3 diffractometer equipped with a Spellman generator (50 kV, 40 mA) and a Kappa CCD detector, operating with Mo-K_α radiation (λ = 0.71071 Å).

Data collection and data reduction were performed with the CrysAlisPro software.[29] Absorption correction using the multiscan method[30] was applied. The structures were solved with SHELXS-97,[31] refined with SHELXL-97[32] and finally checked using PLATON.[33] Details for data collection and structure refinement are summarized in Table 11.

CCDC-2027327 contains supplementary crystallographic data for this compound. These data can be obtained free of charge from The Cambridge Crystallographic Data Centre *via* www.ccdc.cam.ac.uk/data_request/cif.

[29] Program package 'CrysAlisPro 1.171.40.82a (Rigaku OD, 2020)'.

[30] Program package 'CrysAlisPro 1.171.40.82a (Rigaku OD, 2020)'.

[31] Sheldrick, G. M. (1997) SHELXS-97: *Program for Crystal Structure Solution*, University of Göttingen, Germany.

[32] Sheldrick, G. M. (1997) SHELXL-97: *Program for the Refinement of Crystal Structures*, University of Göttingen, Germany.

[33] Spek, A. L. (1999) PLATON: *A Multipurpose Crystallographic Tool*, Utrecht University, Utrecht, The Netherlands.

Table 11. Details for X-ray data collection and structure refinement for compound **4ah**.

	**4ah**
Empirical formula	$C_{16}H_{17}NO$
Formula mass	239.30
T[K]	123(2)
Crystal size [mm]	0.40 × 0.20 × 0.03
Crystal description	colorless platelet
Crystal system	monoclinic
Space group	*P*21/*c*
a [Å]	9.2621(3)
b [Å]	12.2356(4)
c [Å]	22.9278(8)
α [°]	90.0
β [°]	90.780(3)
γ [°]	90.0
V [$Å^3$]	2598.11(15)
Z	8
$\rho_{calcd.}$ [g cm^{-3}]	1.224
μ [mm^{-1}]	0.076
F(000)	1024
Θ range [°]	1.88 – 25.24
Index ranges	$-11 \leq h \leq 11$
	$-15 \leq k \leq 15$
	$-28 \leq l \leq 28$
Reflns. collected	34878
Reflns. obsd.	3828
Reflns. unique	5322 (R_{int} = 0.0521)
R_1, wR_2 (2σ data)	0.0488, 0.0935
R_1, wR_2 (all data)	0.0763, 0.1057
GOOF on F^2	1.020
Peak/hole [e $Å^{-3}$]	0.315 / -0.341

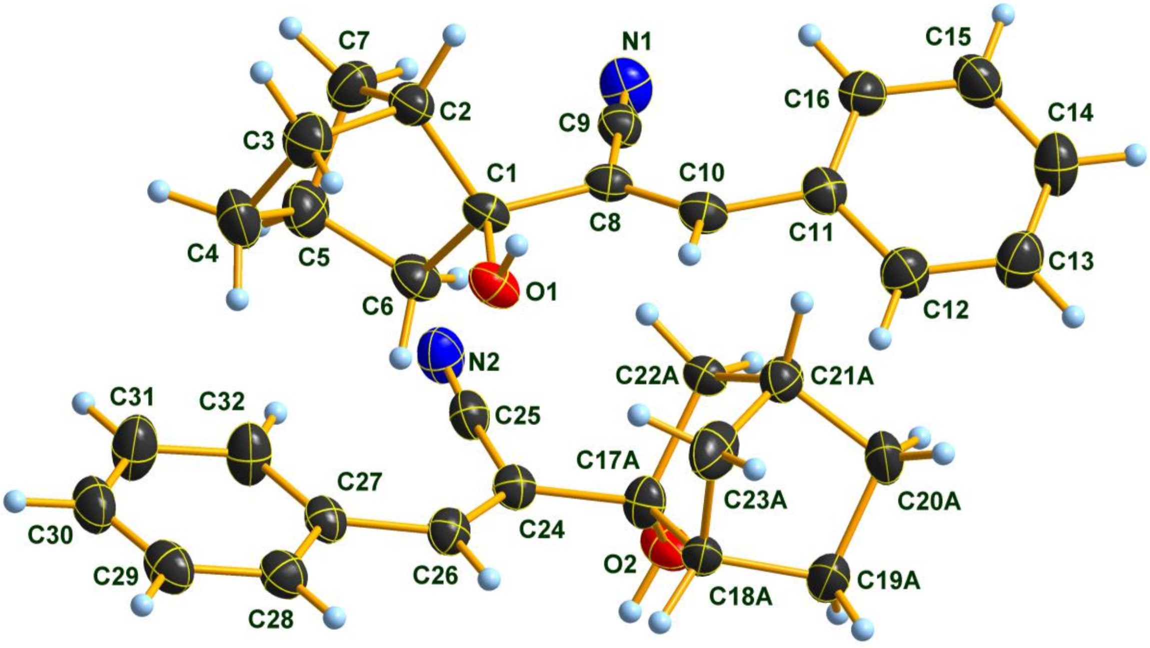

Figure 4. Molecular structure of compound **4ah** in the crystal; view of the two crystallographically independent molecules. In one of the molecules (bottom) the norbornane ring is disordered over two positions. Only the position with higher occupancy (70 %) has been shown for clarity. DIAMOND[34] representation; thermal ellipsoids are drawn at 50 % probability level.

Table 12. Selected bond lengths (Å) of compound **4ah**.

O1 – C1	1.425(2)	C32 – C31	1.380(3)
C8 – C10	1.340(2)	C3 – C4	1.552(3)
C8 – C9	1.443(2)	C30 – C31	1.378(3)
C8 – C1	1.537(2)	C17A – C18A	1.536(3)
C9 – N1	1.143(2)	C17A – C22A	1.632(3)
O2 – C17A	1.417(2)	C18A – C19A	1.527(4)
C10 – C11	1.469(2)	C18A – C23A	1.534(3)
C24 – C26	1.339(2)	C19A – C20A	1.541(4)
C24 – C25	1.439(2)	C20A – C21A	1.537(4)
C24 – C17A	1.533(2)	C21A – C23A	1.424(3)
C26 – C27	1.473(2)	C21A – C22A	1.538(4)
C12 – C13	1.383(3)	C28 – C29	1.384(2)
C12 – C11	1.394(2)	C15 – C14	1.380(3)
C11 – C16	1.393(2)	C29 – C30	1.374(3)

[34] DIAMOND, Crystal Impact GbR., Version 3.2i.

C27 – C28	1.392(2)	C14 – C13	1.375(3)
C27 – C32	1.393(2)	C16 – C15	1.383(2)
C2 – C3	1.536(2)	C1 – C6	1.556(2)
C2 – C7	1.537(3)	C5 – C6	1.520(3)
C2 – C1	1.554(2)	C5 – C7	1.521(3)
C25 – N2	1.148(2)	C5 – C4	1.537(3)

Table 13. Selected bond angles (°) of compound **4ah**.

C10 – C8 – C9	119.7(2)	C29 – C30 – C31	119.3(2)
C10 – C8 – C1	123.8(2)	C14 – C13 – C12	120.0(2)
C9 – C8 – C1	116.3(1)	C30 – C31 – C32	120.9(2)
N1 – C9 – C8	176.3(2)	C5 – C4 – C3	103.1(1)
C8 – C10 – C11	129.4(2)	O2 – C17A – C24	108.1(1)
C26 – C24 – C25	122.9(2)	O2 – C17A – C18A	120.4(2)
C26 – C24 – C17A	126.1(2)	C24 – C17A – C18A	113.7(2)
C25 – C24 – C17A	111.0(1)	O2 – C17A – C22A	105.6(2)
C24 – C26 – C27	131.4(2)	C24 – C17A – C22A	109.0(2)
C13 – C12 – C11	121.0(2)	C18A – C17A – C22A	99.0(2)
C16 – C11 – C12	118.5(2)	C19A – C18A – C23A	99.4(2)
C16 – C11 – C10	123.6(2)	C19A – C18A – C17A	109.3(2)
C12 – C11 – C10	117.8(2)	C23A – C18A – C17A	101.3(2)
C28 – C27 – C32	117.7(2)	C18A – C19A – C20A	104.5(2)
C28 – C27 – C26	117.2(2)	C21A – C20A – C19A	102.3(2)
C32 – C27 – C26	125.1(2)	C23A – C21A – C20A	102.2(2)
C3 – C2 – C7	99.6(2)	C23A – C21A – C22A	100.6(2)
C3 – C2 – C1	108.0(1)	C20A – C21A – C22A	107.4(2)
C7 – C2 – C1	102.5(1)	C21A – C22A – C17A	104.3(2)
N2 – C25 – C24	175.4(2)	C21A – C23A – C18A	99.0(2)
C15 – C16 – C11	119.9(2)	C29 – C28 – C27	121.3(2)
O1 – C1 – C8	108.0(1)	C14 – C15 – C16	120.9(2)
O1 – C1 – C2	112.9(1)	C5 – C6 – C1	103.7(1)
C8 – C1 – C2	112.7(1)	C30 – C29 – C28	120.2(2)
O1 – C1 – C6	107.7(1)	C13 – C14 – C15	119.7(2)
C8 – C1 – C6	113.2(1)	C31 – C32 – C27	120.7(2)
C2 – C1 – C6	102.2(1)	C5 – C7 – C2	94.9(1)
C6 – C5 – C7	100.7(1)	C2 – C3 – C4	103.1(2)
C6 – C5 – C4	109.5(2)	C7 – C5 – C4	101.9(2)

Table 14. Selected torsion angles (°) of compound **4ah**.

C9 – C8 – C10 – C11	3.8(3)	C1 – C2 – C7 – C5	53.0(2)
C1 – C8 – C10 – C11	179.5(2)	C7 – C2 – C3 – C4	39.3(2)
C25 – C24 – C26 – C27	-1.4(3)	C1 – C2 – C3 – C4	-67.3(2)
C17A – C24 – C26 – C27	176.9(2)	C28 – C29 – C30 – C31	-1.7(3)
C13 – C12 – C11 – C16	-2.0(3)	C15 – C14 – C13 – C12	0.4(3)
C13 – C12 – C11 – C10	177.8(2)	C11 – C12 – C13 – C14	0.8(3)
C8 – C10 – C11 – C16	32.0(3)	C29 – C30 – C31 – C32	1.3(3)
C8 – C10 – C11 – C12	-147.8(2)	C27 – C32 – C31 – C30	0.8(3)
C24 – C26 – C27 – C28	-177.8(2)	C6 – C5 – C4 – C3	74.5(2)
C24 – C26 – C27 – C32	-0.4(3)	C7 – C5 – C4 – C3	-31.5(2)
C12 – C11 – C16 – C15	1.9(3)	C2 – C3 – C4 – C5	-5.1(2)
C10 – C11 – C16 – C15	-177.9(2)	C26 – C24 – C17A – O2	125.5(2)
C10 – C8 – C1 – O1	-6.0(2)	C25 – C24 – C17A – O2	-56.0(2)
C9 – C8 – C1 – O1	169.9(1)	C26 – C24 – C17A – C18A	-10.9(3)
C10 – C8 – C1 – C2	119.5(2)	C25 – C24 – C17A – C18A	167.5(2)
C9 – C8 – C1 – C2	-64.7(2)	C26 – C24 – C17A – C22A	-120.2(2)
C10 – C8 – C1 – C6	-125.1(2)	C25 – C24 – C17A – C22A	58.2(2)
C9 – C8 – C1 – C6	50.7(2)	O2 – C17A – C18A – C19A	43.8(3)
C3 – C2 – C1 – O1	-39.7(2)	C24 – C17A – C18A – C19A	174.3(2)
C7 – C2 – C1 – O1	-144.3(1)	C22A – C17A – C18A – C19A	-70.3(2)
C3 – C2 – C1 – C8	-162.4(1)	O2 – C17A – C18A – C23A	148.0(2)
C7 – C2 – C1 – C8	93.1(2)	C24 – C17A – C18A – C23A	-81.4(2)
C3 – C2 – C1 – C6	75.8(2)	C22A – C17A – C18A – C23A	34.0(2)
C7 – C2 – C1 – C6	-28.8(2)	C23A – C18A – C19A – C20A	-30.8(3)
C32 – C27 – C28 – C29	1.9(3)	C17A – C18A – C19A – C20A	74.8(3)
C26 – C27 – C28 – C29	179.5(2)	C18A – C19A – C20A – C21A	-0.5(3)
C11 – C16 – C15 – C14	-0.7(3)	C19A – C20A – C21A – C23A	34.7(3)
C7 – C5 – C6 – C1	40.9(2)	C19A – C20A – C21A – C22A	-70.7(3)
C4 – C5 – C6 – C1	-65.8(2)	C23A – C21A – C22A – C17A	-33.7(2)
O1 – C1 – C6 – C5	112.1(2)	C20A – C21A – C22A – C17A	72.8(2)
C8 – C1 – C6 – C5	-128.6(2)	O2 – C17A – C22A – C21A	-127.0(2)
C2 – C1 – C6 – C5	-7.1(2)	C24 – C17A – C22A – C21A	117.2(2)
C27 – C28 – C29 – C30	0.1(3)	C18A – C17A – C22A – C21A	-1.8(2)
C16 – C15 – C14 – C13	-0.5(3)	C20A – C21A – C23A – C18A	-54.8(2)
C28 – C27 – C32 – C31	-2.3(3)	C22A – C21A – C23A – C18A	55.8(2)
C26 – C27 – C32 – C31	-179.7(2)	C19A – C18A – C23A – C21A	53.4(2)
C6 – C5 – C7 – C2	-57.6(2)	C17A – C18A – C23A – C21A	-58.6(2)

C4 – C5 – C7 – C2	55.2(2)	C3 – C2 – C7 – C5	-58.0(2)

Single crystals of compound **7bk**, suitable for X-ray diffraction, were obtained by slow evaporation of CH_2Cl_2 solution. The crystals were introduced into perfluorinated oil and a suitable single crystal was carefully mounted on the top of a thin glass wire. Data collection was performed with an Oxford Xcalibur 3 diffractometer equipped with a Spellman generator (50 kV, 40 mA) and a Kappa CCD detector, operating with Mo-K_α radiation (λ = 0.71071 Å).

Data collection and data reduction were performed with the CrysAlisPro software.[35] Absorption correction using the multiscan method[36] was applied. The structures were solved with SHELXS-97,[37] refined with SHELXL-97[38] and finally checked using PLATON.[39] Details for data collection and structure refinement are summarized in Table 15.

CCDC-2027330 contains supplementary crystallographic data for this compound. These data can be obtained free of charge from The Cambridge Crystallographic Data Centre *via* www.ccdc.cam.ac.uk/data_request/cif.

[35] Program package CrysAlisPro 1.171.39.46e (Rigaku OD, 2018).
[36] Program package CrysAlisPro 1.171.39.46e (Rigaku OD, 2018).
[37] Sheldrick, G. M. (1997) SHELXS-97: *Program for Crystal Structure Solution*, University of Göttingen, Germany.
[38] Sheldrick, G. M. (1997) SHELXL-97: *Program for the Refinement of Crystal Structures*, University of Göttingen, Germany.
[39] Spek, A. L. (1999) PLATON: *A Multipurpose Crystallographic Tool*, Utrecht University, Utrecht, The Netherlands.

Table 15. Details for X-ray data collection and structure refinement for compound **7bk**.

	**7bk**
Empirical formula	$C_{19}H_{19}NO_2$
Formula mass	293.35
T[K]	143(2)
Crystal size [mm]	0.47 × 0.23 × 0.11
Crystal description	colorless block
Crystal system	Triclinic
Space group	*P*-1
a [Å]	7.0122(4)
b [Å]	8.2204(6)
c [Å]	14.2407(8)
α [°]	104.500(5)
β [°]	94.810(5)
γ [°]	103.432(6)
V [Å^3]	764.15(9)
Z	2
$\rho_{calcd.}$ [g cm^{-3}]	1.275
μ [mm^{-1}]	0.082
F(000)	312
Θ range [°]	3.39 – 25.24
Index ranges	$-9 \leq h \leq 10$
	$-11 \leq k \leq 11$
	$-20 \leq l \leq 20$
Reflns. collected	15402
Reflns. obsd.	3719
Reflns. unique	4648 (R_{int} = 0.0307)
R_1, wR_2 (2σ data)	0.0447, 0.1051
R_1, wR_2 (all data)	0.0583, 0.1151
GOOF on F^2	1.032
Peak/hole [e Å^{-3}]	0.363 / -0.199

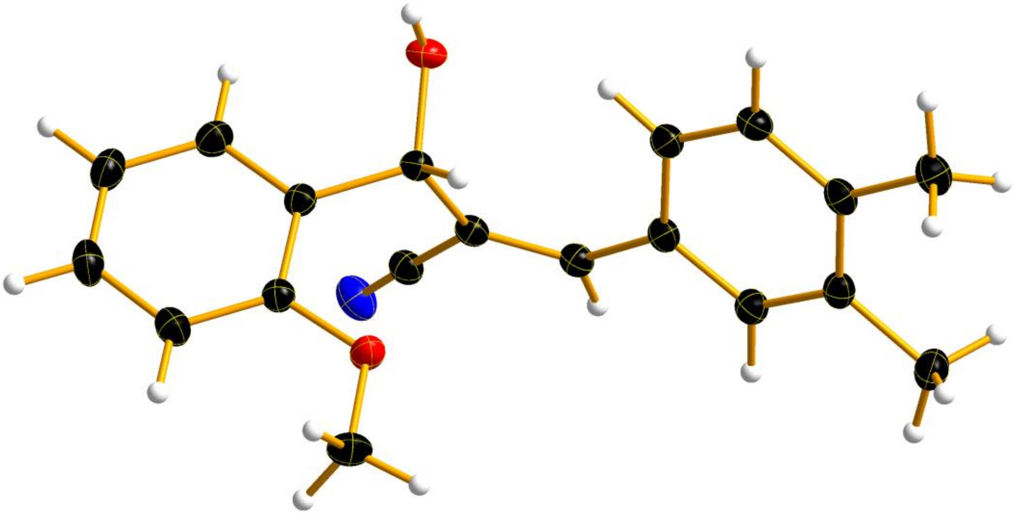

Figure 5. Molecular structure of compound **7bk** in the crystal, DIAMOND[40] representation; thermal ellipsoids are drawn at 50 % probability level.

Table 16. Selected bond lengths (Å) of compound **7bk**.

O1 – C6	1.366(1)	C10 – C15	1.398(2)
O1 – C17	1.426(1)	C16 – N1	1.147(2)
O2 – C7	1.428(1)	C2 – C3	1.391(2)
C6 – C5	1.387(2)	C14 – C15	1.388(2)
C6 – C1	1.340(1)	C3 – C4	1.380(2)
C1 – C2	1.388(2)	C5 – C4	1.387(2)
C1 – C7	1.512(2)	C9 – C10	1.464(2)
C7 – C8	1.516(2)	C8 – C16	1.440(2)
C13 – C14	1.395(2)	C11 – C12	1.390(2)
C13 – C12	1.403(2)	C11 – C10	1.401(2)
C13 – C19	1.503(2)	C12 – C18	1.503(2)
C9 – C8	1.346(2)		

Table 17. Selected bond angles (°) of compound **7bk**.

C6 – O1 – C17	117.2(1)	C15 – C14 – C13	122.2(1)
O1 – C6 – C5	124.3(1)	C14 – C15 – C10	119.5(1)
O1 – C6 – C1	115.1(1)	C4 – C3 – C2	119.3(1)
C5 – C6 – C1	120.6(1)	C6 – C5 – C4	119.4(1)
C2 – C1 – C6	118.7(1)	C3 – C4 – C5	120.9(1)

[40] DIAMOND, Crystal Impact GbR., Version 3.2i.

C2 – C1 – C7	122.2(1)	C16 – C8 – C7	117.5(1)
C6 – C1 – C7	119.1(1)	C12 – C11 – C10	122.4(1)
O2 – C7 – C1	112.7(1)	C11 – C12 – C13	118.9(1)
O2 – C7 – C8	106.9(1)	C11 – C12 – C18	120.4(1)
C1 – C7 – C8	112.2(1)	C13 – C12 – C18	120.7(1)
C14 – C13 – C12	118.7(1)	C15 – C10 – C11	118.2(1)
C14 – C13 – C19	120.3(1)	C15 – C10 – C9	124.1(1)
C12 – C13 – C19	121.0(1)	C11 – C10 – C9	117.6(1)
C8 – C9 – C10	129.3(1)	N1 – C16 – C8	178.0(1)
C9 – C8 – C16	116.0(1)	C1 – C2 – C3	121.0(1)
C9 – C8 – C7	126.6(1)		

Table 18. Selected torsion angles (°) of compound **7bk**.

C17 – O1 – C6 – C5	-1.6(2)	C19 – C13 – C12 – C11	-179.6(1)
C17 – O1 – C6 – C1	178.5(1)	C14 – C13 – C12 – C18	178.0(1)
O1 – C6 – C1 – C2	177.9(1)	C19 – C13 – C12 – C18	-1.2(2)
C5 – C6 – C1 – C2	-2.0(2)	C12 – C11 – C10 – C15	2.4(2)
O1 – C6 – C1 – C7	-3.2(1)	C12 – C11 – C10 – C9	179.4(1)
C5 – C6 – C1 – C7	176.9(1)	C8 – C9 – C10 – C15	-28.3(2)
C2 – C1 – C7 – O2	17.2(1)	C8 – C9 – C10 – C11	155.0(1)
C6 – C1 – C7 – O2	-161.6(1)	C6 – C1 – C2 – C3	1.5(2)
C2 – C1 – C7 – C8	-103.5(1)	C7 – C1 – C2 – C3	-177.4(1)
C6 – C1 – C7 – C8	77.7(1)	C12 – C13 – C14 – C15	1.1(2)
C10 – C9 – C8 – C16	178.9(1)	C19 – C13 – C14 – C15	-179.7(1)
C10 – C9 – C8 – C7	-2.5(2)	C13 – C14 – C15 – C10	-0.1(2)
O2 – C7 – C8 – C9	89.7(1)	C11 – C10 – C15 – C14	-1.7(2)
C1 – C7 – C8 – C9	-146.3(1)	C9 – C10 – C15 – C14	-178.4(1)
O2 – C7 – C8 – C16	-91.7(1)	C1 – C2 – C3 – C4	0.2(2)
C1 – C7 – C8 – C16	32.3(1)	O1 – C6 – C5 – C4	-179.1(1)
C10 – C11 – C12 – C13	-1.3(2)	C1 – C6 – C5 – C4	0.9(2)
C10 – C11 – C12 – C18	-179.8(1)	C2 – C3 – C4 – C5	-1.4(2)
C14 – C13 – C12 – C11	-0.4(2)	C6 – C5 – C4 – C3	0.9(2)

Single crystals of compound **7dm**, suitable for X-ray diffraction, were obtained by slow evaporation of CH_2Cl_2 solution. The crystals were introduced into perfluorinated oil and a suitable single crystal was carefully mounted on the top of a thin glass wire. Data collection was performed with an Oxford Xcalibur 3 diffractometer equipped with a Spellman generator (50 kV, 40 mA) and a Kappa CCD detector, operating with Mo-K_α radiation (λ = 0.71071 Å).

Data collection and data reduction were performed with the CrysAlisPro software.[41] Absorption correction using the multiscan method[42] was applied. The structures were solved with SHELXS-97,[43] refined with SHELXL-97[44] and finally checked using PLATON.[45] Details for data collection and structure refinement are summarized in Table 19.

CCDC-2027331 contains supplementary crystallographic data for this compound. These data can be obtained free of charge from The Cambridge Crystallographic Data Centre *via* www.ccdc.cam.ac.uk/data_request/cif.

[41] Program package 'CrysAlisPro 1.171.39.46e (Rigaku OD, 2018)'.
[42] Program package 'CrysAlisPro 1.171.39.46e (Rigaku OD, 2018)'.
[43] Sheldrick, G. M. (1997) SHELXS-97: *Program for Crystal Structure Solution*, University of Göttingen, Germany.
[44] Sheldrick, G. M. (1997) SHELXL-97: *Program for the Refinement of Crystal Structures*, University of Göttingen, Germany.
[45] Spek, A. L. (1999) PLATON: *A Multipurpose Crystallographic Tool*, Utrecht University, Utrecht, The Netherlands.

Table 19. Details for X-ray data collection and structure refinement for compound **7dm**.

	**7dm**
Empirical formula	$C_{19}H_{25}NO$
Formula mass	283.40
T[K]	143(2)
Crystal size [mm]	0.40 × 0.40 × 0.30
Crystal description	colorless block
Crystal system	monoclinic
Space group	*P*21/*c*
a [Å]	15.0865(5)
b [Å]	19.5830(4)
c [Å]	11.8768(3)
α [°]	90.0
β [°]	106.655(2)
γ [°]	90.0
V [$Å^3$]	3361.66(16)
Z	8
$\rho_{calcd.}$ [g cm^{-3}]	1.120
μ [mm^{-1}]	0.068
F(000)	1232
Θ range [°]	3.31 – 25.24
Index ranges	$-21 \leq h \leq 21$
	$-27 \leq k \leq 27$
	$-16 \leq l \leq 16$
Reflns. collected	68256
Reflns. obsd.	7556
Reflns. unique	10231 (R_{int} = 0.0554)
R_1, wR_2 (2σ data)	0.0510, 0.1220
R_1, wR_2 (all data)	0.0733, 0.1380
GOOF on F^2	1.027
Peak/hole [e $Å^{-3}$]	0.392 / -0.210

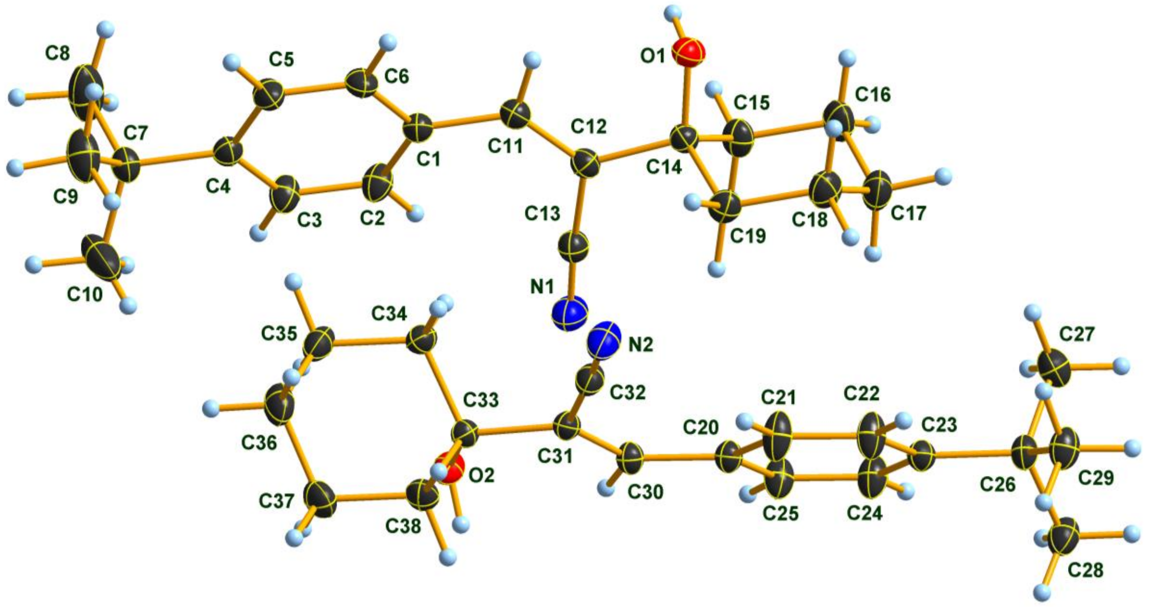

Figure 6. Molecular structure of compound **7dm** in the crystal. DIAMOND[46] representation; thermal ellipsoids are drawn at 50 % probability level.

Table 20. Selected bond lengths (Å) of compound **7dm**.

O1 – C14	1.422(1)	C3 – C2	1.388(2)
C14 – C19	1.533(2)	C36 – C35	1.517(2)
C14 – C12	1.535(2)	C36 – C37	1.525(2)
C14 – C15	1.536(2)	C16 – C17	1.526(2)
C7 – C10	1.522(2)	C16 – C15	1.528(2)
C7 – C8	1.526(2)	C18 – C17	1.522(2)
C7 – C9	1.527(2)	C13 – N1	1.149(2)
C7 – C4	1.529(2)	C22 – C21	1.390(2)
C12 – C11	1.343(2)	C4 – C5	1.391(2)
C12 – C13	1.440(2)	C4 – C3	1.399(2)
O2 – C33	1.419(1)	C1 – C2	1.394(2)
C31 – C30	1.348(2)	C1 – C6	1.396(2)
C31 – C32	1.436(2)	C32 – N2	1.151(2)
C31 – C33	1.536(2)	C6 – C5	1.389(2)
C23 – C22	1.388(2)	C25 – C24	1.389(2)
C23 – C24	1.393(2)	C25 – C20	1.399(2)
C23 – C26	1.530(2)	C20 – C21	1.394(2)
C19 – C18	1.530(2)	C38 – C37	1.526(2)

46 DIAMOND, Crystal Impact GbR., Version 3.2i.

C30 – C20	1.465(2)	C26 – C27	1.534(2)
C34 – C35	1.527(2)	C26 – C28	1.537(2)
C34 – C33	1.535(2)	C33 – C38	1.534(2)
C26 – C29	1.532(2)	C11 – C1	1.465(2)

Table 21. Selected bond angles (°) of compound **7dm**.

O1 – C14 – C19	106.6(1)	C25 – C24 – C23	121.9(1)
O1 – C14 – C12	109.6(1)	C17 – C16 – C15	110.8(1)
C19 – C14 – C12	109.4(1)	C17 – C18 – C19	110.9(1)
O1 – C14 – C15	110.4(1)	C16 – C15 – C14	111.9(1)
C19 – C14 – C15	110.4(1)	C36 – C35 – C34	111.5(1)
C12 – C14 – C15	110.3(1)	C3 – C2 – C1	120.9(1)
C10 – C7 – C8	109.2(1)	C6 – C5 – C4	121.5(1)
C10 – C7 – C9	107.2(1)	N1 – C13 – C12	175.6(1)
C8 – C7 – C9	109.3(1)	C23 – C22 – C21	122.3(1)
C10 – C7 – C4	111.4(1)	C18 – C17 – C16	110.8(1)
C8 – C7 – C4	108.1(1)	C22 – C21 – C20	121.3(1)
C9 – C7 – C4	111.6(1)	C3 – C4 – C7	121.1(1)
C11 – C12 – C13	122.6(1)	C2 – C1 – C6	117.5(1)
C11 – C12 – C14	123.2(1)	C2 – C1 – C11	124.9(1)
C13 – C12 – C14	114.2(1)	C6 – C1 – C11	117.6(1)
C30 – C31 – C32	122.1(1)	N2 – C32 – C31	176.5(1)
C30 – C31 – C33	123.8(1)	C5 – C6 – C1	121.4(1)
C32 – C31 – C33	114.1(1)	C24 – C25 – C20	121.6(1)
C22 – C23 – C24	116.3(1)	C21 – C20 – C25	116.6(1)
C22 – C23 – C26	122.3(1)	C21 – C20 – C30	125.4(1)
C24 – C23 – C26	121.4(1)	C25 – C20 – C30	118.0(1)
C18 – C19 – C14	111.8(1)	C37 – C38 – C33	112.2(1)
C31 – C30 – C20	130.1(1)	C2 – C3 – C4	121.9(1)
C35 – C34 – C33	112.3(1)	C35 – C36 – C37	110.9(1)
C23 – C26 – C29	112.4(1)	C36 – C37 – C38	110.7(1)
C23 – C26 – C27	108.4(1)	O2 – C33 – C31	110.1(1)
C29 – C26 – C27	108.5(1)	C38 – C33 – C31	109.6(1)
C23 – C26 – C28	110.7(1)	C34 – C33 – C31	109.9(1)
C29 – C26 – C28	107.6(1)	C12 – C11 – C1	130.7(1)
C27 – C26 – C28	109.3(1)	C5 – C4 – C3	116.9(1)
O2 – C33 – C38	110.7(1)	C5 – C4 – C7	122.0(1)
O2 – C33 – C34	106.1(1)	C38 – C33 – C34	110.3(1)

Table 22. Selected torsion angles (°) of compound **7dm**.

O1 – C14 – C12 – C11	-7.0(2)	C2 – C1 – C6 – C5	1.9(2)
C19 – C14 – C12 – C11	109.6(1)	C11 – C1 – C6 – C5	-178.0(1)
C15 – C14 – C12 – C11	-128.8(1)	C24 – C25 – C20 – C21	-1.6(2)
O1 – C14 – C12 – C13	175.4(1)	C24 – C25 – C20 – C30	179.0(1)
C19 – C14 – C12 – C13	-68.1(1)	C31 – C30 – C20 – C21	13.8(2)
C15 – C14 – C12 – C13	53.6(1)	C31 – C30 – C20 – C25	-166.8(1)
O1 – C14 – C19 – C18	-65.7(1)	O2 – C33 – C38 – C37	-63.2(1)
C12 – C14 – C19 – C18	175.8(1)	C34 – C33 – C38 – C37	54.0(1)
C15 – C14 – C19 – C18	54.2(1)	C31 – C33 – C38 – C37	175.2(1)
C32 – C31 – C30 – C20	1.0(2)	C5 – C4 – C3 – C2	1.6(2)
C33 – C31 – C30 – C20	-179.4(1)	C7 – C4 – C3 – C2	179.8(1)
C22 – C23 – C26 – C29	14.0(2)	C35 – C36 – C37 – C38	56.8(2)
C24 – C23 – C26 – C29	-167.6(1)	C33 – C38 – C37 – C36	-56.4(2)
C22 – C23 – C26 – C27	-105.9(1)	C20 – C25 – C24 – C23	1.1(2)
C24 – C23 – C26 – C27	72.5(1)	C22 – C23 – C24 – C25	0.0(2)
C22 – C23 – C26 – C28	134.3(1)	C26 – C23 – C24 – C25	-178.6(1)
C24 – C23 – C26 – C28	-47.3(2)	C14 – C19 – C18 – C17	-56.1(1)
C35 – C34 – C33 – O2	67.0(1)	C17 – C16 – C15 – C14	55.9(2)
C35 – C34 – C33 – C38	-52.9(1)	O1 – C14 – C15 – C16	63.3(1)
C35 – C34 – C33 – C31	-174.0(1)	C19 – C14 – C15 – C16	-54.3(1)
C30 – C31 – C33 – O2	-7.8(2)	C12 – C14 – C15 – C16	-175.3(1)
C32 – C31 – C33 – O2	171.8(1)	C37 – C36 – C35 – C34	-56.2(1)
C30 – C31 – C33 – C38	114.2(1)	C33 – C34 – C35 – C36	54.8(1)
C32 – C31 – C33 – C38	-66.2(1)	C4 – C3 – C2 – C1	0.4(2)
C30 – C31 – C33 – C34	-124.4(1)	C6 – C1 – C2 – C3	-2.2(2)
C32 – C31 – C33 – C34	55.3(1)	C11 – C1 – C2 – C3	177.7(1)
C13 – C12 – C11 – C1	-1.4(2)	C1 – C6 – C5 – C4	0.2(2)
C14 – C12 – C11 – C1	-178.9(1)	C3 – C4 – C5 – C6	-1.9(2)
C10 – C7 – C4 – C5	-151.1(1)	C7 – C4 – C5 – C6	179.9(1)
C8 – C7 – C4 – C5	88.9(2)	C24 – C23 – C22 – C21	-0.4(2)
C9 – C7 – C4 – C5	-31.3(2)	C26 – C23 – C22 – C21	178.1(1)
C10 – C7 – C4 – C3	30.8(2)	C19 – C18 – C17 – C16	57.0(2)
C8 – C7 – C4 – C3	-89.2(2)	C15 – C16 – C17 – C18	-56.9(2)
C9 – C7 – C4 – C3	150.7(1)	C23 – C22 – C21 – C20	-0.2(2)
C12 – C11 – C1 – C2	-19.6(2)	C25 – C20 – C21 – C22	1.2(2)
C12 – C11 – C1 – C6	160.3(1)	C30 – C20 – C21 – C22	-179.5(1)

Single crystals of compound **7ee**, suitable for X-ray diffraction, were obtained by slow evaporation of CH_2Cl_2 solution. The crystals were introduced into perfluorinated oil and a suitable single crystal was carefully mounted on the top of a thin glass wire. Data collection was performed with an Oxford Xcalibur 3 diffractometer equipped with a Spellman generator (50 kV, 40 mA) and a Kappa CCD detector, operating with Mo-K_α radiation (λ = 0.71071 Å). Data collection and data reduction were performed with the CrysAlisPro software.[47] Absorption correction using the multiscan method[48] was applied. The structures were solved with SHELXS-97,[49] refined with SHELXL-97[50] and finally checked using PLATON.[51] Details for data collection and structure refinement are summarized in Table 23.

CCDC-2027328 contains supplementary crystallographic data for this compound. These data can be obtained free of charge from The Cambridge Crystallographic Data Centre *via* www.ccdc.cam.ac.uk/data_request/cif.

[47] Program package 'CrysAlisPro 1.171.39.46e (Rigaku OD, 2018)'.
[48] Program package 'CrysAlisPro 1.171.39.46e (Rigaku OD, 2018)'.
[49] Sheldrick, G. M. (1997) SHELXS-97: *Program for Crystal Structure Solution*, University of Göttingen, Germany.
[50] Sheldrick, G. M. (1997) SHELXL-97: *Program for the Refinement of Crystal Structures*, University of Göttingen, Germany.
[51] Spek, A. L. (1999) PLATON: *A Multipurpose Crystallographic Tool*, Utrecht University, Utrecht, The Netherlands.

Table 23. Details for X-ray data collection and structure refinement for compound **7ee**.

	**7ee**
Empirical formula	$C_{16}H_{14}F_3N$
Formula mass	277.28
T[K]	143(2)
Crystal size [mm]	0.40 × 0.05 × 0.05
Crystal description	colorless rod
Crystal system	monoclinic
Space group	*P*21/*c*
a [Å]	5.2514(4)
b [Å]	26.0806(18)
c [Å]	10.2125(8)
α [°]	90.0
β [°]	103.387(8)
γ [°]	90.0
V [$Å^3$]	1360.70(18)
Z	4
$\rho_{calcd.}$ [g cm^{-3}]	1.354
μ [mm^{-1}]	0.108
F(000)	576
Θ range [°]	3.74 – 25.24
Index ranges	$-8 \leq h \leq 8$
	$-32 \leq k \leq 32$
	$-11 \leq l \leq 12$
Reflns. collected	9402
Reflns. obsd.	1848
Reflns. unique	2673 (R_{int} = 0.0664)
R_1, wR_2 (2σ data)	0.0635, 0.1523
R_1, wR_2 (all data)	0.0939, 0.1748
GOOF on F^2	1.030
Peak/hole [e $Å^{-3}$]	0.666 / -0.240

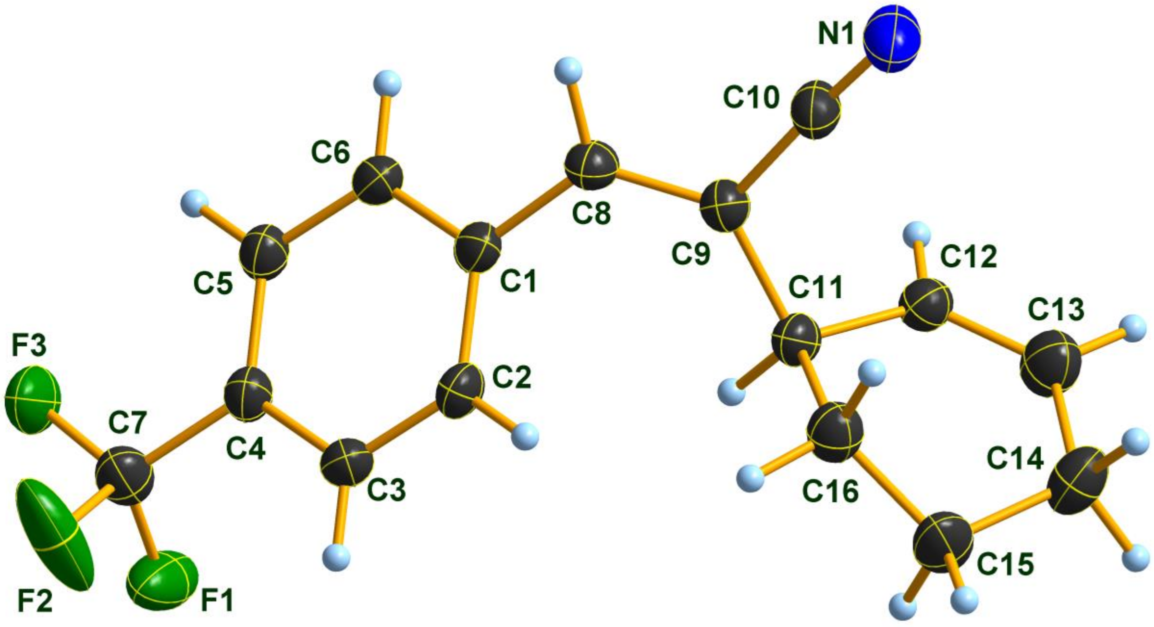

Figure 7. Molecular structure of compound **7ee** in the crystal. DIAMOND[52] representation; thermal ellipsoids are drawn at 50 % probability level.

Table 24. Selected bond lengths (Å) of compound **7ee**.

F1 – C7	1.348(3)	C4 – C3	1.392(4)
C5 – C6	1.385(4)	C4 – C7	1.490(4)
C5 – C4	1.389(4)	C3 – C2	1.374(4)
C8 – C9	1.342(4)	F2 – C7	1.320(3)
C8 – C1	1.472(3)	C12 – C13	1.324(4)
C9 – C10	1.446(4)	C12 – C11	1.515(3)
C9 – C11	1.513(3)	C14 – C15	1.504(4)
F3 – C7	1.330(3)	C14 – C13	1.511(4)
C1 – C6	1.393(3)	C16 – C11	1.538(3)
C1 – C2	1.397(3)	N1 – C10	1.150(3)
C16 – C15	1.514(4)		

Table 25. Selected bond angles (°) of compound **7ee**.

C6 – C5 – C4	119.3(2)	C9 – C11 – C16	111.3(2)
C9 – C8 – C1	127.5(2)	C12 – C11 – C16	110.6(2)
C8 – C9 – C10	117.4(2)	N1 – C10 – C9	176.7(3)
C8 – C9 – C11	128.6(2)	C3 – C2 – C1	120.8(2)

[52] DIAMOND, Crystal Impact GbR., Version 3.2i.

C10 – C9 – C11	114.1(2)	F2 – C7 – F3	107.0(2)
C6 – C1 – C2	118.5(2)	F2 – C7 – F1	106.4(2)
C6 – C1 – C8	119.5(2)	F3 – C7 – F1	104.9(2)
C2 – C1 – C8	121.9(2)	F2 – C7 – C4	112.3(2)
C5 – C6 – C1	121.3(2)	F3 – C7 – C4	113.8(2)
C15 – C16 – C11	112.0(2)	F1 – C7 – C4	111.9(2)
C5 – C4 – C3	120.1(2)	C15 – C14 – C13	111.8(2)
C5 – C4 – C7	121.7(2)	C14 – C15 – C16	112.9(2)
C3 – C4 – C7	118.1(2)	C12 – C13 – C14	122.6(3)
C2 – C3 – C4	120.1(2)	C9 – C11 – C12	109.6(2)
C13 – C12 – C11	125.3(3)		

Table 26. Selected torsion angles (°) of compound **7ee**.

C1 – C8 – C9 – C10	-175.1(2)	C13 – C12 – C11 – C16	11.1(4)
C1 – C8 – C9 – C11	6.3(5)	C15 – C16 – C11 – C9	-162.6(2)
C9 – C8 – C1 – C6	-145.1(3)	C15 – C16 – C11 – C12	-40.5(3)
C9 – C8 – C1 – C2	39.4(4)	C4 – C3 – C2 – C1	0.6(4)
C4 – C5 – C6 – C1	1.4(4)	C6 – C1 – C2 – C3	-0.3(4)
C2 – C1 – C6 – C5	-0.7(4)	C8 – C1 – C2 – C3	175.3(2)
C8 – C1 – C6 – C5	-176.4(2)	C5 – C4 – C7 – F2	-122.8(3)
C6 – C5 – C4 – C3	-1.1(4)	C3 – C4 – C7 – F2	55.1(3)
C6 – C5 – C4 – C7	176.7(2)	C5 – C4 – C7 – F3	-1.0(4)
C5 – C4 – C3 – C2	0.1(4)	C3 – C4 – C7 – F3	176.8(2)
C7 – C4 – C3 – C2	-177.8(2)	C5 – C4 – C7 – F1	117.6(3)
C8 – C9 – C11 – C12	124.2(3)	C3 – C4 – C7 – F1	-64.5(3)
C10 – C9 – C11 – C12	-54.4(3)	C13 – C14 – C15 – C16	-43.9(4)
C8 – C9 – C11 – C16	-113.1(3)	C11 – C16 – C15 – C14	59.1(3)
C10 – C9 – C11 – C16	68.3(3)	C11 – C12 – C13 – C14	2.6(5)
C13 – C12 – C11 – C9	134.2(3)	C15 – C14 – C13 – C12	13.7(4)

Single crystals of compound **7fn**, suitable for X-ray diffraction, were obtained by slow evaporation of CH_2Cl_2 solution. The crystals were introduced into perfluorinated oil and a suitable single crystal was carefully mounted on the top of a thin glass wire. Data collection was performed with an Oxford Xcalibur 3 diffractometer equipped with a Spellman generator (50 kV, 40 mA) and a Kappa CCD detector, operating with Mo-K_α radiation (λ = 0.71071 Å).

Data collection and data reduction were performed with the CrysAlisPro software.[53] Absorption correction using the multiscan method[54] was applied. The structures were solved with SHELXS-97,[55] refined with SHELXL-97[56] and finally checked using PLATON.[57] Details for data collection and structure refinement are summarized in Table 27.

CCDC-1831885 contains supplementary crystallographic data for this compound. These data can be obtained free of charge from The Cambridge Crystallographic Data Centre *via* www.ccdc.cam.ac.uk/data_request/cif.

---

[53] Program package CrysAlisPro 1.171.38.46 (Rigaku OD, 2015).

[54] Program package CrysAlisPro 1.171.38.46 (Rigaku OD, 2015).

[55] Sheldrick, G. M. (1997) SHELXS-97: *Program for Crystal Structure Solution*, University of Göttingen, Germany.

[56] Sheldrick, G. M. (1997) SHELXL-97: *Program for the Refinement of Crystal Structures*, University of Göttingen, Germany.

[57] Spek, A. L. (1999) PLATON: *A Multipurpose Crystallographic Tool*, Utrecht University, Utrecht, The Netherlands.

Table 27. Details for X-ray data collection and structure refinement for compound **7fn**.

	**7fn**
Empirical formula	$C_{12}H_{10}F_3NO_2$
Formula mass	257.21
T[K]	143(2)
Crystal size [mm]	0.45 × 0.12 × 0.06
Crystal description	colorless rod
Crystal system	monoclinic
Space group	*P*21/*c*
a [Å]	12.3101(13)
b [Å]	8.1799(6)
c [Å]	12.7623(11)
α [°]	90
β [°]	112.598(12)
γ [°]	90
V [Å^3]	1186.4(2)
Z	4
$\rho_{calcd.}$ [g cm^{-3}]	1.440
μ [mm^{-1}]	0.129
F(000)	528
Θ range [°]	4.16 – 25.24
Index ranges	$-16 \leq h \leq 16$
	$-10 \leq k \leq 10$
	$-16 \leq l \leq 17$
Reflns. collected	10879
Reflns. obsd.	2130
Reflns. unique	2943 (R_{int} = 0.0425)
R_1, wR_2 (2σ data)	0.0491, 0.1053
R_1, wR_2 (all data)	0.0719, 0.1204
GOOF on F^2	1.044
Peak/hole [e Å^{-3}]	0.275 / -0.284

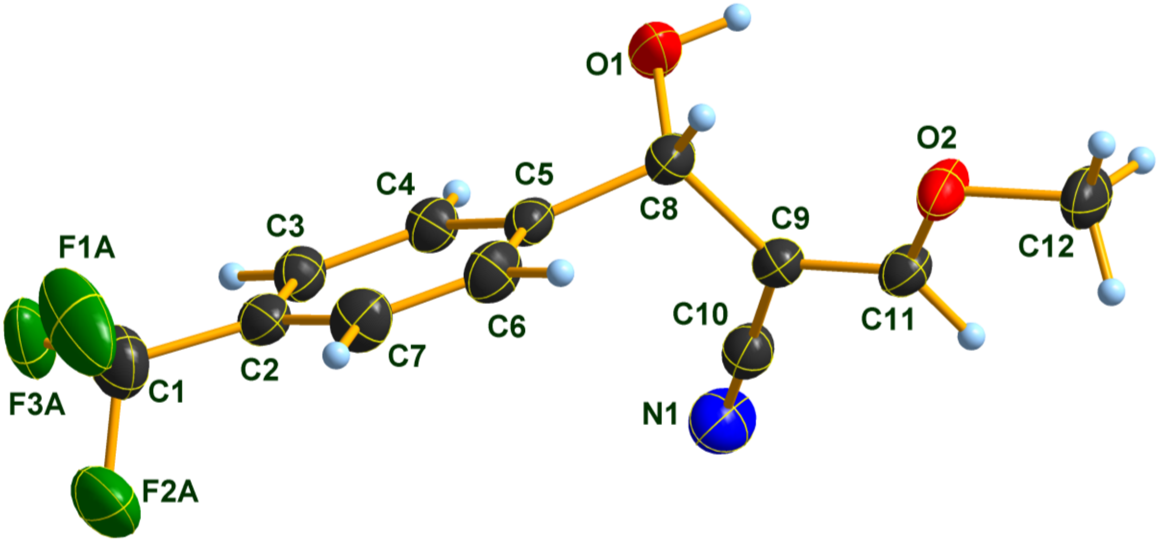

Figure 8. Molecular structure of compound **7fn** in the crystal, DIAMOND[58] representation; thermal ellipsoids are drawn at 50 % probability level. The CF_3 group is disordered over two positions; only one position is shown for clarity.

[58] DIAMOND, Crystal Impact GbR., Version 3.2i.

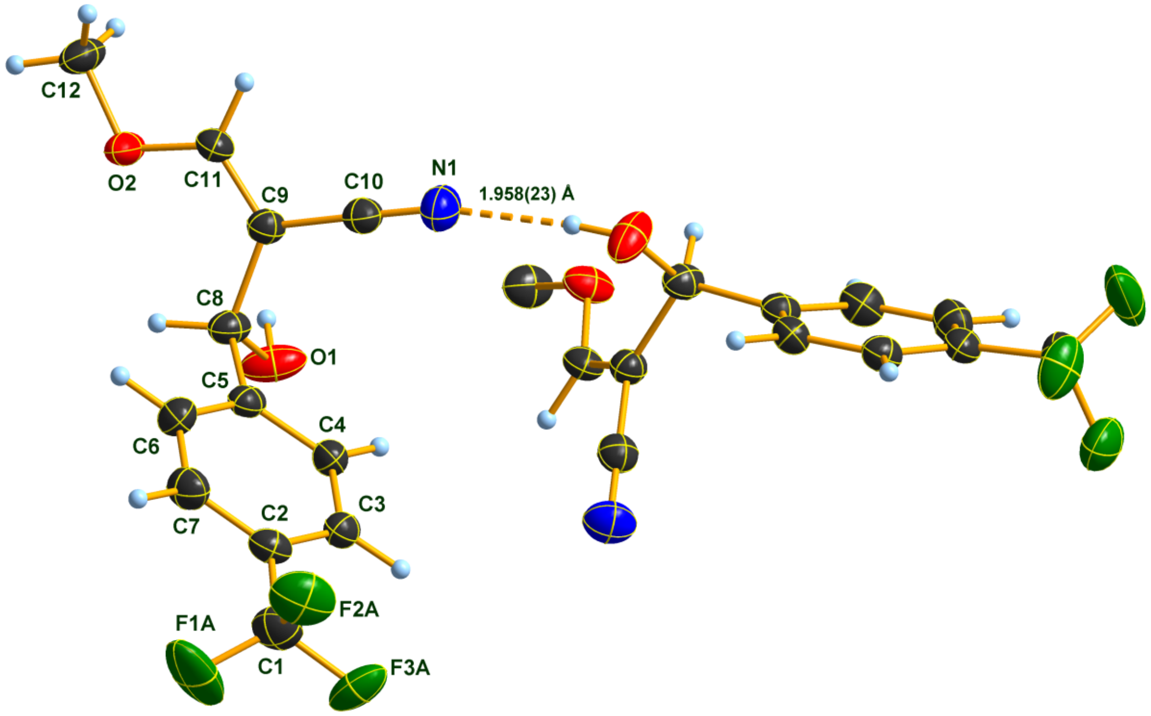

Figure 9. Hydrogen bonding in the crystal of compound **7fn**, DIAMOND[59] representation; thermal ellipsoids are drawn at 50 % probability level. Symmetry code for the second (not labeled) molecule: -x, -0.5+y, 0.5-z.

Table 28. Selected bond lengths (Å) of compound **7fn**.

C9 – C11	1.340(2)	C6 – C7	1.381(2)
C9 – C10	1.426(2)	C1 – F1A	1.312(4)
C9 – C8	1.519(2)	C1 – F2A	1.356(4)
C8 – O1	1.418(2)	C1 – F3A	1.408(5)
C8 – C5	1.512(2)	C10 – N1	1.146(2)
O2 – C11	1.333(2)	C2 – C3	1.383(2)
O2 – C12	1.440(2)	C2 – C7	1.384(2)
C5 – C6	1.386(2)	C2 – C1	1.494(2)
C5 – C4	1.391(2)	C4 – C3	1.386(2)

[59] DIAMOND, Crystal Impact GbR., Version 3.2i.

Table 29. Selected bond angles (°) of compound **7fn**.

C11 – C9 – C10	117.2(1)	C7 – C6 – C5	120.8(2)
C11 – C9 – C8	123.6(1)	C6 – C7 – C2	119.8(2)
C10 – C9 – C8	119.0(1)	F1A – C1 – F2A	102.4(4)
O1 – C8 – C5	107.3(1)	F1A – C1 – F3A	116.8(4)
O1 – C8 – C9	111.3(1)	F2A – C1 – F3A	98.3(3)
C5 – C8 – C9	113.6(1)	F1A – C1 – C2	113.5(2)
C11 – O2 – C12	115.2(1)	F2A – C1 – C2	112.5(2)
C6 – C5 – C4	118.9(2)	F3A – C1 – C2	111.9(2)
C6 – C5 – C8	119.8(1)	C7 – C2 – C1	118.7(2)
C4 – C5 – C8	121.2(2)	C3 – C4 – C5	120.5(2)
N1 – C10 – C9	179.1(2)	O2 – C11 – C9	121.1(1)
C3 – C2 – C7	120.1(2)	C2 – C3 – C4	119.8(2)
C3 – C2 – C1	121.1(2)		

Table 30. Selected torsion angles (°) of compound **7fn**.

C11 – C9 – C8 – O1	104.5(2)	C3 – C2 – C1 – F1A	-140.1(4)
C10 – C9 – C8 – O1	-71.0(2)	C7 – C2 – C1 – F1A	37.1(5)
C11 – C9 – C8 – C5	-134.3(2)	C3 – C2 – C1 – F2A	104.1(3)
C10 – C9 – C8 – C5	50.1(2)	C7 – C2 – C1 – F2A	-78.6(3)
O1 – C8 – C5 – C6	-145.4(2)	C3 – C2 – C1 – F3A	-5.3(3)
C9 – C8 – C5 – C6	91.2(2)	C7 – C2 – C1 – F3A	172.0(2)
O1 – C8 – C5 – C4	31.6(2)	C1 – C2 – C7 – C6	-175.5(2)
C9 – C8 – C5 – C4	-91.8(2)	C1 – C2 – C3 – C4	175.2(2)
C6 – C5 – C4 – C3	1.7(2)	C5 – C4 – C3 – C2	0.2(2)
C8 – C5 – C4 – C3	-175.3(1)	C4 – C5 – C6 – C7	-2.0(2)
C12 – O2 – C11 – C9	-173.1(2)	C8 – C5 – C6 – C7	175.1(2)
C10 – C9 – C11 – O2	178.9(2)	C5 – C6 – C7 – C2	0.3(3)
C8 – C9 – C11 – O2	3.3(2)	C3 – C2 – C7 – C6	1.8(3)
C7 – C2 – C3 – C4	-2.0(2)		

Single crystals of compound **7fd**, suitable for X-ray diffraction, were obtained by slow evaporation of CH_2Cl_2 solution. The crystals were introduced into perfluorinated oil and a suitable single crystal was carefully mounted on the top of a thin glass wire. Data collection was performed with an Oxford Xcalibur 3 diffractometer equipped with a Spellman generator (50 kV, 40 mA) and a Kappa CCD detector, operating with Mo-K_α radiation (λ = 0.71071 Å).

Data collection and data reduction were performed with the CrysAlisPro software.[60] Absorption correction using the multiscan method[61] was applied. The structures were solved with SHELXS-97,[62] refined with SHELXL-97[63] and finally checked using PLATON.[64] Details for data collection and structure refinement are summarized in Table 31.

CCDC-2027324 contains supplementary crystallographic data for this compound. These data can be obtained free of charge from The Cambridge Crystallographic Data Centre *via* www.ccdc.cam.ac.uk/data_request/cif.

---

[60] Program package 'CrysAlisPro 1.171.39.46e (Rigaku OD, 2018)'.
[61] Program package 'CrysAlisPro 1.171.39.46e (Rigaku OD, 2018)'.
[62] Sheldrick, G. M. (1997) SHELXS-97: *Program for Crystal Structure Solution*, University of Göttingen, Germany.
[63] Sheldrick, G. M. (1997) SHELXL-97: *Program for the Refinement of Crystal Structures*, University of Göttingen, Germany.
[64] Spek, A. L. (1999) PLATON: *A Multipurpose Crystallographic Tool*, Utrecht University, Utrecht, The Netherlands.

Table 31. Details for X-ray data collection and structure refinement for compound **7fd**.

	**7fd**
Empirical formula	$C_{12}H_{13}NO_2$
Formula mass	203.23
T[K]	143(2)
Crystal size [mm]	0.20 × 0.15 × 0.03
Crystal description	colorless platelet
Crystal system	monoclinic
Space group	*P*21/*c*
a [Å]	10.7401(17)
b [Å]	11.8914(16)
c [Å]	9.1036(12)
α [°]	90.0
β [°]	111.337(17)
γ [°]	90.0
V [$Å^3$]	1083.0(3)
Z	4
$\rho_{calcd.}$ [g cm^{-3}]	1.246
μ [mm^{-1}]	0.085
F(000)	432
Θ range [°]	4.05 – 25.24
Index ranges	$-13 \leq h \leq 13$
	$-14 \leq k \leq 14$
	$-9 \leq l \leq 11$
Reflns. collected	6315
Reflns. obsd.	1355
Reflns. unique	2005 (R_{int} = 0.0648)
R_1, wR_2 (2σ data)	0.0820, 0.1732
R_1, wR_2 (all data)	0.1231, 0.1953
GOOF on F^2	1.053
Peak/hole [e $Å^{-3}$]	0.349 / -0.259

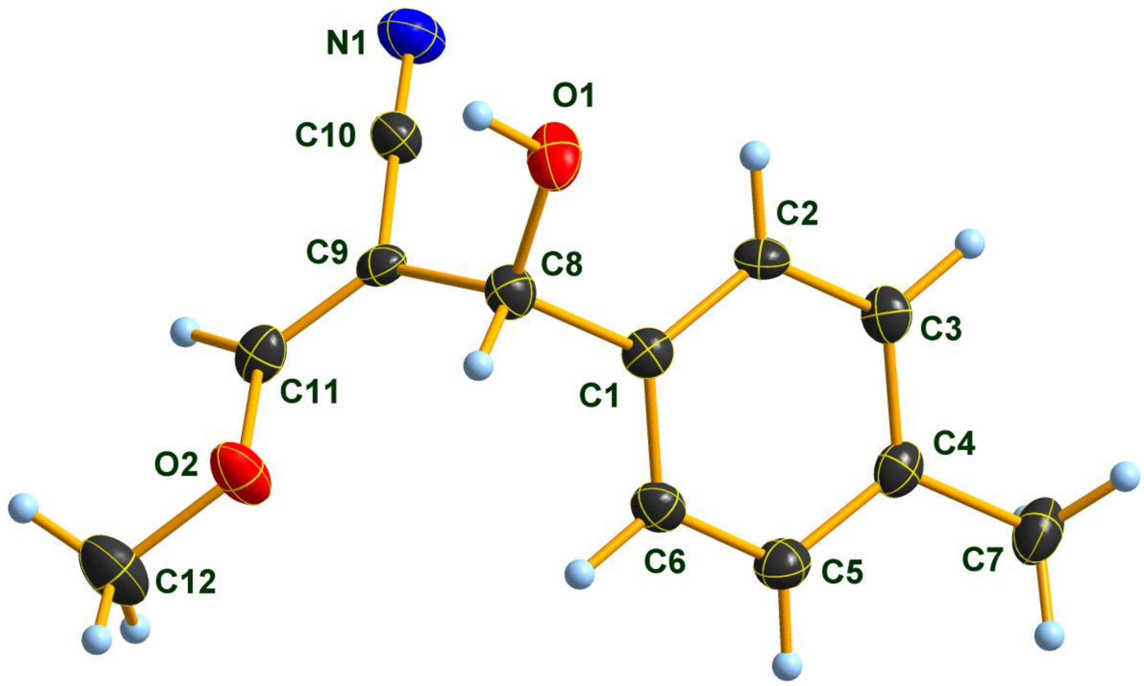

Figure 10. Molecular structure of compound **7fd** in the crystal. DIAMOND[65] representation; thermal ellipsoids are drawn at 50 % probability level.

Table 32. Selected bond lengths (Å) of compound **7fd**.

O1 – C8	1.420(4)	C8 – C9	1.522(5)
O2 – C11	1.335(4)	C4 – C5	1.387(5)
O2 – C12	1.442(5)	C4 – C7	1.513(5)
C1 – C2	1.384(5)	C11 – C9	1.330(5)
C1 – C6	1.391(5)	C10 – C9	1.434(5)
C1 – C8	1.512(5)	C6 – C5	1.388(5)
C2 – C3	1.377(5)	C3 – C4	1.396(5)
N1 – C10	1.137(5)		

Table 33. Selected bond angles (°) of compound **7fd**.

C11 – O2 – C12	116.5(3)	C9 – C11 – O2	120.9(3)
C2 – C1 – C6	118.6(3)	C4 – C5 – C6	120.9(4)
C2 – C1 – C8	121.5(3)	N1 – C10 – C9	178.4(4)
C6 – C1 – C8	119.9(3)	C11 – C9 – C10	118.6(3)
C3 – C2 – C1	121.1(3)	C11 – C9 – C8	124.9(3)
C5 – C6 – C1	120.4(3)	C10 – C9 – C8	116.5(3)

[65] DIAMOND, Crystal Impact GbR., Version 3.2i.

C2 – C3 – C4	120.6(3)	C5 – C4 – C3	118.3(3)
O1 – C8 – C1	107.7(3)	C5 – C4 – C7	121.4(3)
O1 – C8 – C9	111.8(3)	C3 – C4 – C7	120.3(3)
C1 – C8 – C9	110.4(3)		

Table 34. Selected torsion angles (°) of compound **7fd**.

C6 – C1 – C2 – C3	-1.9(5)	C12 – O2 – C11 – C9	-174.6(3)
C8 – C1 – C2 – C3	-179.9(3)	C3 – C4 – C5 – C6	-1.5(5)
C2 – C1 – C6 – C5	0.7(5)	C7 – C4 – C5 – C6	177.0(3)
C8 – C1 – C6 – C5	178.8(3)	C1 – C6 – C5 – C4	1.0(5)
C1 – C2 – C3 – C4	1.4(5)	O2 – C11 – C9 – C10	-178.9(3)
C2 – C1 – C8 – O1	-27.4(4)	O2 – C11 – C9 – C8	3.7(5)
C6 – C1 – C8 – O1	154.7(3)	O1 – C8 – C9 – C11	-136.1(3)
C2 – C1 – C8 – C9	95.0(4)	C1 – C8 – C9 – C11	104.0(4)
C6 – C1 – C8 – C9	-83.0(4)	O1 – C8 – C9 – C10	46.4(4)
C2 – C3 – C4 – C5	0.3(5)	C1 – C8 – C9 – C10	-73.4(4)
C2 – C3 – C4 – C7	-178.2(3)		

Single crystals of compound **7gc**, suitable for X-ray diffraction, were obtained by slow evaporation of CH_2Cl_2 solution. The crystals were introduced into perfluorinated oil and a suitable single crystal was carefully mounted on the top of a thin glass wire. Data collection was performed with an Oxford Xcalibur 3 diffractometer equipped with a Spellman generator (50 kV, 40 mA) and a Kappa CCD detector, operating with Mo-K_α radiation (λ = 0.71071 Å).

Data collection and data reduction were performed with the CrysAlisPro software.[66] Absorption correction using the multiscan method[67] was applied. The structures were solved with SHELXS-97,[68] refined with SHELXL-97[69] and finally checked using PLATON.[70] Details for data collection and structure refinement are summarized in Table 35.

CCDC-2027325 contains supplementary crystallographic data for this compound. These data can be obtained free of charge from The Cambridge Crystallographic Data Centre *via* www.ccdc.cam.ac.uk/data_request/cif.

[66] Program package 'CrysAlisPro 1.171.39.46e (Rigaku OD, 2018)'.
[67] Program package 'CrysAlisPro 1.171.39.46e (Rigaku OD, 2018)'.
[68] Sheldrick, G. M. (1997) SHELXS-97: *Program for Crystal Structure Solution*, University of Göttingen, Germany.
[69] Sheldrick, G. M. (1997) SHELXL-97: *Program for the Refinement of Crystal Structures*, University of Göttingen, Germany.
[70] Spek, A. L. (1999) PLATON: *A Multipurpose Crystallographic Tool*, Utrecht University, Utrecht, The Netherlands.

Table 35. Details for X-ray data collection and structure refinement for compound **7gc**.

	**7gc**
Empirical formula	$C_{12}H_{11}Cl_2NO_2$
Formula mass	272.12
T[K]	143(2)
Crystal size [mm]	0.10 × 0.10 × 0.04
Crystal description	colorless block
Crystal system	triclinic
Space group	*P*-1
a [Å]	8.6835(4)
b [Å]	14.3010(7)
c [Å]	20.7726(11)
α [°]	98.709(4)
β [°]	91.623(4)
γ [°]	92.076(4)
V [Å^3]	2546.7(2)
Z	8
$\rho_{calcd.}$ [g cm^{-3}]	1.419
μ [mm^{-1}]	0.498
F(000)	1120
Θ range [°]	3.27 – 25.24
Index ranges	$-10 \leq h \leq 10$
	$-17 \leq k \leq 17$
	$-25 \leq l \leq 25$
Reflns. collected	20854
Reflns. obsd.	6770
Reflns. unique	10356 (R_{int} = 0.0414)
R_1, wR_2 (2σ data)	0.0523, 0.0937
R_1, wR_2 (all data)	0.0930, 0.1122
GOOF on F^2	1.017
Peak/hole [e Å^{-3}]	0.419 / -0.286

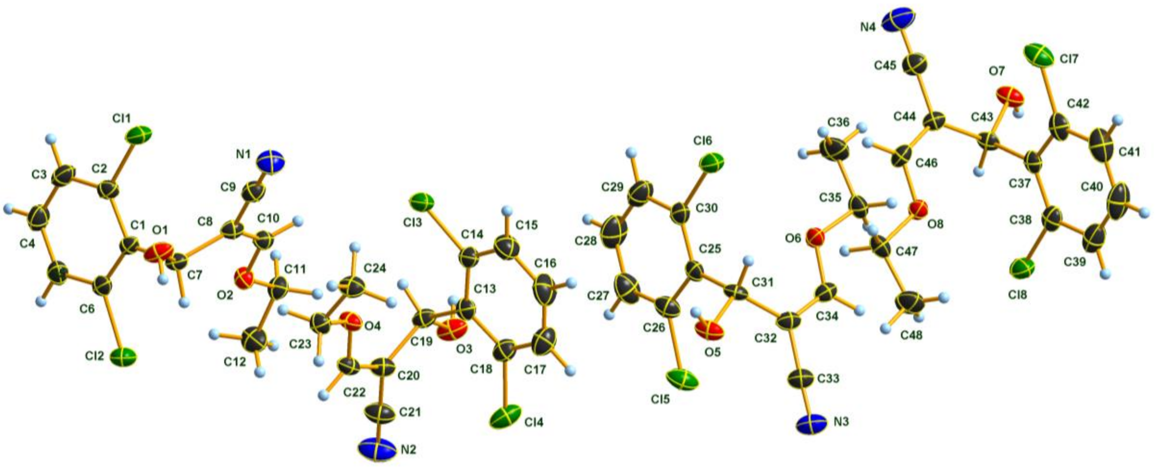

Figure 11. Molecular structure of compound **7gc** in the crystal. DIAMOND[71] representation; thermal ellipsoids are drawn at 50 % probability level.

Table 36. Selected bond lengths (Å) of compound **7gc**.

Cl6 – C30	1.753(3)	C26 – C25	1.387(4)
Cl3 – C14	1.751(3)	C26 – C27	1.390(4)
Cl1 – C2	1.731(3)	C45 – N4	1.137(4)
Cl4 – C18	1.741(3)	C35 – C36	1.491(4)
Cl2 – C6	1.750(3)	C47 – C48	1.500(4)
Cl8 – C38	1.742(3)	C4 – C5	1.375(4)
Cl7 – C42	1.744(3)	C4 – C3	1.387(4)
Cl5 – C26	1.740(3)	C34 – C32	1.335(4)
O5 – C31	1.419(3)	C14 – C15	1.367(4)
O2 – C10	1.331(3)	C30 – C29	1.383(4)
O2 – C11	1.451(3)	C30 – C25	1.398(4)
O4 – C22	1.327(3)	C2 – C3	1.389(4)
O4 – C23	1.455(3)	C6 – C5	1.379(4)
O8 – C46	1.343(3)	C21 – N2	1.142(4)
O8 – C47	1.452(3)	C8 – C9	1.428(4)
O1 – C7	1.423(3)	C17 – C16	1.376(4)
C46 – C44	1.334(4)	C29 – C28	1.379(5)
O3 – C19	1.417(3)	C24 – C23	1.492(4)
C13 – C18	1.397(4)	C27 – C28	1.374(4)
C13 – C14	1.405(4)	C39 – C40	1.365(5)
C13 – C19	1.511(4)	C41 – C40	1.373(5)
O6 – C34	1.337(3)	C15 – C16	1.392(4)

[71] DIAMOND, Crystal Impact GbR., Version 3.2i.

O6 – C35	1.450(3)	C37 – C42	1.396(4)
C20 – C22	1.331(4)	C37 – C38	1.402(4)
C20 – C21	1.427(4)	C37 – C43	1.514(4)
C20 – C19	1.536(4)	C31 – C32	1.521(4)
C11 – C12	1.496(4)	C31 – C25	1.522(4)
C1 – C6	1.403(4)	C18 – C17	1.390(4)
C1 – C2	1.410(4)	C42 – C41	1.383(4)
C1 – C7	1.515(4)	N1 – C9	1.150(4)
C44 – C45	1.433(4)	C38 – C39	1.390(4)
C44 – C43	1.523(4)	C7 – C8	1.534(4)
C10 – C8	1.337(4)	C33 – N3	1.140(3)
O7 – C43	1.426(3)	C33 – C32	1.430(4)

Table 37. Selected bond angles (°) of compound **7gc**.

C10 – O2 – C11	117.7(2)	C25 – C30 – Cl6	120.8(2)
C22 – O4 – C23	118.7(2)	O4 – C22 – C20	118.8(2)
C46 – O8 – C47	117.0(2)	C3 – C2 – C1	122.5(3)
C44 – C46 – O8	119.0(3)	C3 – C2 – Cl1	117.3(2)
C18 – C13 – C14	115.2(3)	C1 – C2 – Cl1	120.2(2)
C18 – C13 – C19	123.0(3)	C5 – C6 – C1	123.4(3)
C14 – C13 – C19	121.8(3)	C5 – C6 – Cl2	116.4(2)
C34 – O6 – C35	116.9(2)	C1 – C6 – Cl2	120.2(2)
C22 – C20 – C21	120.5(3)	N2 – C21 – C20	179.4(4)
C22 – C20 – C19	120.7(3)	O7 – C43 – C37	111.1(2)
C21 – C20 – C19	118.7(2)	O7 – C43 – C44	109.7(2)
O2 – C11 – C12	106.8(2)	C37 – C43 – C44	113.1(2)
C6 – C1 – C2	115.0(3)	C10 – C8 – C9	119.7(3)
C6 – C1 – C7	122.0(2)	C10 – C8 – C7	121.3(3)
C2 – C1 – C7	122.9(3)	C9 – C8 – C7	118.8(2)
C46 – C44 – C45	118.4(3)	C4 – C3 – C2	119.3(3)
C46 – C44 – C43	122.5(2)	C4 – C5 – C6	119.4(3)
C45 – C44 – C43	118.8(2)	N1 – C9 – C8	177.9(3)
O2 – C10 – C8	118.7(3)	C16 – C17 – C18	119.3(3)
C42 – C37 – C38	115.4(3)	C28 – C29 – C30	118.7(3)
C42 – C37 – C43	122.7(3)	C26 – C25 – C30	115.7(3)
C38 – C37 – C43	121.9(3)	C26 – C25 – C31	122.6(2)
O5 – C31 – C32	108.6(2)	C30 – C25 – C31	121.7(3)
O5 – C31 – C25	111.4(2)	C34 – C32 – C33	118.7(3)

C32 – C31 – C25	113.9(2)	C34 – C32 – C31	121.0(2)
C17 – C18 – C13	122.9(3)	C33 – C32 – C31	120.1(2)
C17 – C18 – Cl4	117.2(2)	C28 – C27 – C26	119.0(3)
C13 – C18 – Cl4	120.0(2)	O4 – C23 – C24	106.6(2)
C41 – C42 – C37	123.0(3)	C40 – C39 – C38	119.3(3)
C41 – C42 – Cl7	117.1(3)	C40 – C41 – C42	119.0(3)
C37 – C42 – Cl7	119.9(2)	C39 – C40 – C41	121.0(3)
C39 – C38 – C37	122.3(3)	C27 – C28 – C29	120.8(3)
C39 – C38 – Cl8	117.0(2)	C14 – C15 – C16	119.4(3)
C37 – C38 – Cl8	120.7(2)	C17 – C16 – C15	120.0(3)
O1 – C7 – C1	109.7(2)	C5 – C4 – C3	120.3(3)
O1 – C7 – C8	110.3(2)	C32 – C34 – O6	118.6(3)
C1 – C7 – C8	113.1(2)	O3 – C19 – C13	111.9(2)
N3 – C33 – C32	178.4(3)	O3 – C19 – C20	108.4(2)
C25 – C26 – C27	122.8(3)	C13 – C19 – C20	112.7(2)
C25 – C26 – Cl5	120.9(2)	C15 – C14 – C13	123.2(3)
C27 – C26 – Cl5	116.3(3)	C15 – C14 – Cl3	116.3(2)
N4 – C45 – C44	178.5(4)	C13 – C14 – Cl3	120.5(2)
O6 – C35 – C36	106.9(2)	C29 – C30 – C25	123.0(3)
O8 – C47 – C48	106.6(2)	C29 – C30 – Cl6	116.2(2)

Table 38. Selected torsion angles (°) of compound **7gc**.

C47 – O8 – C46 – C44	-177.9(3)	C45 – C44 – C43 – O7	-1.2(4)
C10 – O2 – C11 – C12	168.9(2)	C46 – C44 – C43 – C37	-62.4(4)
O8 – C46 – C44 – C45	177.1(3)	C45 – C44 – C43 – C37	123.3(3)
O8 – C46 – C44 – C43	2.8(4)	O2 – C10 – C8 – C9	176.1(2)
C11 – O2 – C10 – C8	178.5(3)	O2 – C10 – C8 – C7	1.8(4)
C14 – C13 – C18 – C17	0.2(4)	O1 – C7 – C8 – C10	166.9(3)
C19 – C13 – C18 – C17	178.5(3)	C1 – C7 – C8 – C10	-69.8(3)
C14 – C13 – C18 – Cl4	179.8(2)	O1 – C7 – C8 – C9	-7.5(3)
C19 – C13 – C18 – Cl4	-2.0(4)	C1 – C7 – C8 – C9	115.8(3)
C38 – C37 – C42 – C41	-0.1(4)	C5 – C4 – C3 – C2	-0.1(5)
C43 – C37 – C42 – C41	-178.3(3)	C1 – C2 – C3 – C4	0.2(5)
C38 – C37 – C42 – Cl7	-179.2(2)	Cl1 – C2 – C3 – C4	178.8(2)
C43 – C37 – C42 – Cl7	2.6(4)	C3 – C4 – C5 – C6	0.2(5)
C42 – C37 – C38 – C39	-0.2(4)	C1 – C6 – C5 – C4	-0.4(5)
C43 – C37 – C38 – C39	178.1(3)	Cl2 – C6 – C5 – C4	179.2(2)
C42 – C37 – C38 – Cl8	178.7(2)	C13 – C18 – C17 – C16	0.1(5)

C43 – C37 – C38 – Cl8	-3.1(4)	Cl4 – C18 – C17 – C16	-179.4(2)
C6 – C1 – C7 – O1	-114.4(3)	C25 – C30 – C29 – C28	0.2(5)
C2 – C1 – C7 – O1	63.0(3)	Cl6 – C30 – C29 – C28	-179.7(3)
C6 – C1 – C7 – C8	122.0(3)	C27 – C26 – C25 – C30	-1.7(4)
C2 – C1 – C7 – C8	-60.6(3)	Cl5 – C26 – C25 – C30	178.7(2)
C34 – O6 – C35 – C36	173.7(3)	C27 – C26 – C25 – C31	176.3(3)
C46 – O8 – C47 – C48	-176.1(3)	Cl5 – C26 – C25 – C31	-3.3(4)
C35 – O6 – C34 – C32	175.3(3)	C29 – C30 – C25 – C26	1.2(4)
C18 – C13 – C19 – O3	-60.5(3)	Cl6 – C30 – C25 – C26	-179.0(2)
C14 – C13 – C19 – O3	117.7(3)	C29 – C30 – C25 – C31	-176.8(3)
C18 – C13 – C19 – C20	62.0(3)	Cl6 – C30 – C25 – C31	3.0(4)
C14 – C13 – C19 – C20	-119.9(3)	O5 – C31 – C25 – C26	-61.3(3)
C22 – C20 – C19 – O3	-176.2(3)	C32 – C31 – C25 – C26	61.9(3)
C21 – C20 – C19 – O3	1.6(4)	O5 – C31 – C25 – C30	116.5(3)
C22 – C20 – C19 – C13	59.3(4)	C32 – C31 – C25 – C30	-120.3(3)
C21 – C20 – C19 – C13	-122.9(3)	O6 – C34 – C32 – C33	-177.4(3)
C18 – C13 – C14 – C15	-0.5(4)	O6 – C34 – C32 – C31	-2.1(4)
C19 – C13 – C14 – C15	-178.8(3)	O5 – C31 – C32 – C34	-171.1(3)
C18 – C13 – C14 – Cl3	-178.5(2)	C25 – C31 – C32 – C34	64.2(3)
C19 – C13 – C14 – Cl3	3.2(4)	O5 – C31 – C32 – C33	4.2(4)
C23 – O4 – C22 – C20	-170.4(3)	C25 – C31 – C32 – C33	-120.6(3)
C21 – C20 – C22 – O4	-177.5(3)	C25 – C26 – C27 – C28	0.9(5)
C19 – C20 – C22 – O4	0.3(4)	Cl5 – C26 – C27 – C28	-179.5(3)
C6 – C1 – C2 – C3	-0.4(4)	C22 – O4 – C23 – C24	150.8(2)
C7 – C1 – C2 – C3	-178.0(3)	C37 – C38 – C39 – C40	-0.1(5)
C6 – C1 – C2 – Cl1	-178.9(2)	Cl8 – C38 – C39 – C40	-178.9(2)
C7 – C1 – C2 – Cl1	3.5(4)	C37 – C42 – C41 – C40	0.6(5)
C2 – C1 – C6 – C5	0.5(4)	Cl7 – C42 – C41 – C40	179.7(2)
C7 – C1 – C6 – C5	178.1(3)	C38 – C39 – C40 – C41	0.6(5)
C2 – C1 – C6 – Cl2	-179.1(2)	C42 – C41 – C40 – C39	-0.8(5)
C7 – C1 – C6 – Cl2	-1.5(4)	C26 – C27 – C28 – C29	0.6(5)
C42 – C37 – C43 – O7	58.9(3)	C30 – C29 – C28 – C27	-1.1(5)
C38 – C37 – C43 – O7	-119.3(3)	C13 – C14 – C15 – C16	0.5(5)
C42 – C37 – C43 – C44	-64.9(3)	Cl3 – C14 – C15 – C16	178.6(2)
C38 – C37 – C43 – C44	116.9(3)	C18 – C17 – C16 – C15	-0.2(5)
C46 – C44 – C43 – O7	173.1(3)	C14 – C15 – C16 – C17	-0.1(5)

Single crystals of compound **7ho**, suitable for X-ray diffraction, were obtained by slow evaporation of CH_2Cl_2 solution. The crystals were introduced into perfluorinated oil and a suitable single crystal was carefully mounted on the top of a thin glass wire. Data collection was performed with an Oxford Xcalibur 3 diffractometer equipped with a Spellman generator (50 kV, 40 mA) and a Kappa CCD detector, operating with Mo-K_α radiation (λ = 0.71071 Å).

Data collection and data reduction were performed with the CrysAlisPro software.[72] Absorption correction using the multiscan method[73] was applied. The structures were solved with SHELXS-97,[74] refined with SHELXL-97[75] and finally checked using PLATON.[76] Details for data collection and structure refinement are summarized in Table 39.

CCDC-2027329 contains supplementary crystallographic data for this compound. These data can be obtained free of charge from The Cambridge Crystallographic Data Centre *via* www.ccdc.cam.ac.uk/data_request/cif.

[72] Program package 'CrysAlisPro 1.171.39.46e (Rigaku OD, 2018)'.

[73] Program package 'CrysAlisPro 1.171.39.46e (Rigaku OD, 2018)'.

[74] Sheldrick, G. M. (1997) SHELXS-97: *Program for Crystal Structure Solution*, University of Göttingen, Germany.

[75] Sheldrick, G. M. (1997) SHELXL-97: *Program for the Refinement of Crystal Structures*, University of Göttingen, Germany.

[76] Spek, A. L. (1999) PLATON: *A Multipurpose Crystallographic Tool*, Utrecht University, Utrecht, The Netherlands.

Table 39. Details for X-ray data collection and structure refinement for compound **7ho**.

	**7ho**
Empirical formula	$C_{24}H_{26}OS$
Formula mass	362.51
T[K]	123(2)
Crystal size [mm]	0.40 × 0.30 × 0.08
Crystal description	colorless block
Crystal system	triclinic
Space group	*P*-1
a [Å]	6.5370(3)
b [Å]	11.1601(6)
c [Å]	13.1519(9)
α [°]	102.872(5)
β [°]	95.207(5)
γ [°]	91.589(4)
V [Å^3]	930.37(9)
Z	2
$\rho_{calcd.}$ [g cm^{-3}]	1.294
μ [mm^{-1}]	0.184
F(000)	388
Θ range [°]	3.32 – 25.24
Index ranges	$-9 \leq h \leq 9$
	$-15 \leq k \leq 15$
	$-18 \leq l \leq 18$
Reflns. collected	19026
Reflns. obsd.	4569
Reflns. unique	4569 (R_{int} = 0.0356)
R_1, wR_2 (2σ data)	0.0431, 0.1070
R_1, wR_2 (all data)	0.0575, 0.1194
GOOF on F^2	1.036
Peak/hole [e Å^{-3}]	0.489 / -0.258

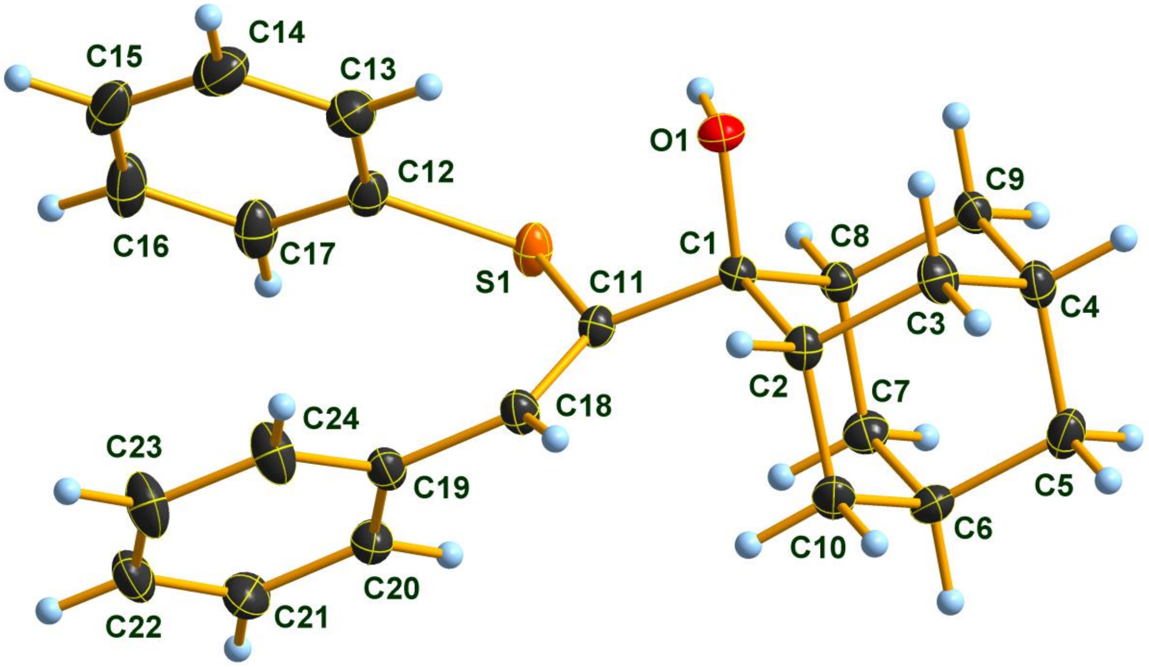

Figure 12. Molecular structure of compound **7ho** in the crystal. DIAMOND[77] representation; thermal ellipsoids are drawn at 50 % probability level.

Table 40. Selected bond lengths (Å) of compound **7ho**.

S1 – C12	1.779(1)	C12 – C13	1.384(2)
S1 – C11	1.789(1)	C12 – C17	1.390(2)
C1 – O1	1.439(1)	C11 – C18	1.340(2)
C1 – C11	1.530(2)	C20 – C21	1.388(2)
C1 – C2	1.543(2)	C20 – C19	1.395(2)
C1 – C8	1.550(2)	C14 – C15	1.385(2)
C2 – C3	1.537(2)	C14 – C13	1.394(2)
C2 – C10	1.539(2)	C16 – C15	1.377(2)
C3 – C4	1.534(2)	C16 – C17	1.392(2)
C4 – C9	1.532(2)	C19 – C24	1.390(2)
C4 – C5	1.534(2)	C19 – C18	1.477(2)
C5 – C6	1.531(2)	C23 – C24	1.388(2)
C10 – C6	1.532(2)	C23 – C22	1.389(2)
C7 – C6	1.534(2)	C21 – C22	1.381(2)
C7 – C8	1.536(2)	C8 – C9	1.532(2)

[77] DIAMOND, Crystal Impact GbR., Version 3.2i.

Table 41. Selected bond angles (°) of compound **7ho**.

C12 – S1 – C11	102.3(1)	C7 – C8 – C1	110.1(1)
O1 – C1 – C11	107.2(1)	C13 – C12 – C17	120.1(1)
O1 – C1 – C2	106.5(1)	C13 – C12 – S1	121.0(1)
C11 – C1 – C2	114.0(1)	C17 – C12 – S1	118.9(1)
O1 – C1 – C8	110.1(1)	C18 – C11 – C1	125.9(1)
C11 – C1 – C8	111.4(1)	C18 – C11 – S1	121.3(1)
C2 – C1 – C8	107.6(1)	C1 – C11 – S1	112.6(1)
C3 – C2 – C10	108.2(1)	C4 – C9 – C8	110.0(1)
C3 – C2 – C1	109.6(1)	C21 – C20 – C19	120.6(1)
C10 – C2 – C1	111.0(1)	C15 – C14 – C13	120.1(1)
C4 – C3 – C2	110.0(1)	C12 – C13 – C14	119.8(1)
C9 – C4 – C5	109.2(1)	C15 – C16 – C17	120.2(1)
C9 – C4 – C3	108.9(1)	C16 – C15 – C14	120.2(1)
C5 – C4 – C3	109.7(1)	C24 – C19 – C20	118.4(1)
C6 – C5 – C4	109.3(1)	C24 – C19 – C18	121.0(1)
C6 – C10 – C2	110.0(1)	C20 – C19 – C18	120.7(1)
C6 – C7 – C8	109.6(1)	C12 – C17 – C16	119.7(1)
C5 – C6 – C10	109.5(1)	C24 – C23 – C22	120.1(1)
C5 – C6 – C7	110.1(1)	C11 – C18 – C19	126.3(1)
C10 – C6 – C7	108.6(1)	C22 – C21 – C20	120.5(1)
C9 – C8 – C7	109.5(1)	C23 – C24 – C19	121.0(1)
C9 – C8 – C1	109.6(1)	C21 – C22 – C23	119.4(1)

Table 42. Selected torsion angles (°) of compound **7ho**.

O1 – C1 – C2 – C3	-57.2(1)	C2 – C1 – C11 – C18	12.0(2)
C11 – C1 – C2 – C3	-175.2(1)	C8 – C1 – C11 – C18	133.9(1)
C8 – C1 – C2 – C3	60.8(1)	O1 – C1 – C11 – S1	68.8(1)
O1 – C1 – C2 – C10	-176.6(1)	C2 – C1 – C11 – S1	-173.7(1)
C11 – C1 – C2 – C10	65.5(1)	C8 – C1 – C11 – S1	-51.7(1)
C8 – C1 – C2 – C10	-58.5(1)	C12 – S1 – C11 – C18	46.0(1)
C10 – C2 – C3 – C4	60.0(1)	C12 – S1 – C11 – C1	-128.7(1)
C1 – C2 – C3 – C4	-61.1(1)	C5 – C4 – C9 – C8	60.3(1)
C2 – C3 – C4 – C9	59.4(1)	C3 – C4 – C9 – C8	-59.5(1)
C2 – C3 – C4 – C5	-60.1(1)	C7 – C8 – C9 – C4	-59.7(1)
C9 – C4 – C5 – C6	-60.0(1)	C1 – C8 – C9 – C4	61.2(1)
C3 – C4 – C5 – C6	59.3(1)	C17 – C12 – C13 – C14	1.8(2)
C3 – C2 – C10 – C6	-60.4(1)	S1 – C12 – C13 – C14	-179.8(1)

C1 – C2 – C10 – C6	59.8(1)	C15 – C14 – C13 – C12	-1.2(2)
C4 – C5 – C6 – C10	-59.5(1)	C17 – C16 – C15 – C14	0.9(2)
C4 – C5 – C6 – C7	59.9(1)	C13 – C14 – C15 – C16	-0.2(2)
C2 – C10 – C6 – C5	60.8(1)	C21 – C20 – C19 – C24	0.9(2)
C2 – C10 – C6 – C7	-59.5(1)	C21 – C20 – C19 – C18	179.8(1)
C8 – C7 – C6 – C5	-59.2(1)	C13 – C12 – C17 – C16	-1.0(2)
C8 – C7 – C6 – C10	60.8(1)	S1 – C12 – C17 – C16	-179.4(1)
C6 – C7 – C8 – C9	58.7(1)	C15 – C16 – C17 – C12	-0.3(2)
C6 – C7 – C8 – C1	-61.9(1)	C1 – C11 – C18 – C19	-177.2(1)
O1 – C1 – C8 – C9	54.7(1)	S1 – C11 – C18 – C19	8.9(2)
C11 – C1 – C8 – C9	173.4(1)	C24 – C19 – C18 – C11	-124.8(2)
C2 – C1 – C8 – C9	-61.0(1)	C20 – C19 – C18 – C11	56.3(2)
O1 – C1 – C8 – C7	175.2(1)	C19 – C20 – C21 – C22	0.8(2)
C11 – C1 – C8 – C7	-66.1(1)	C22 – C23 – C24 – C19	1.4(2)
C2 – C1 – C8 – C7	59.5(1)	C20 – C19 – C24 – C23	-2.0(2)
C11 – S1 – C12 – C13	52.6(1)	C18 – C19 – C24 – C23	179.1(1)
C11 – S1 – C12 – C17	-129.0(1)	C20 – C21 – C22 – C23	-1.4(2)
O1 – C1 – C11 – C18	-105.6(1)	C24 – C23 – C22 – C21	0.3(2)
